# Supplementary material for: Formal group insertion into aryl C‒N bonds through an aromaticity destruction-reconstruction process
Source: Nat Commun. 2018 Aug 24;9:3423. doi: 10.1038/s41467-018-05637-z (PMC6109128; doi:10.1038/s41467-018-05637-z)
Supplement: Supplementary file 1 — Supplementary Information [file 41467_2018_5637_MOESM1_ESM.pdf]

Formal Group Insertion into Aryl C-N Bonds through An Aromaticity Destruction-Reconstruction Process

Han, et al.

Formal Group Insertion into Aryl C-N Bonds through An Aromaticity Destruction-Reconstruction Process

Dandan Han, Qiuqin He, Renhua Fan*

*Corresponding author. Email: rhfan@fudan.edu.cn

Supplementary Information

Supplementary Methods

General Information

All reactions were performed in Schlenk tubes under nitrogen atmosphere. Flash column chromatography was performed using silica gel (60-Å pore size, 32–63 µm, standard grade). Analytical thin–layer chromatography was performed using glass plates pre-coated with 0.25 mm 230–400 mesh silica gel impregnated with a fluorescent indicator (254 nm). Thin layer chromatography plates were visualized by exposure to ultraviolet light. Organic solutions were concentrated on rotary evaporators at ~20 Torr (house vacuum) at 35–40 °C. Commercial reagents and solvents were used as received. Nuclear magnetic resonance (NMR) spectra are recorded in parts per million from internal tetramethylsilane on the δ scale.

Supplementary Table 1. Evaluation of Conditions

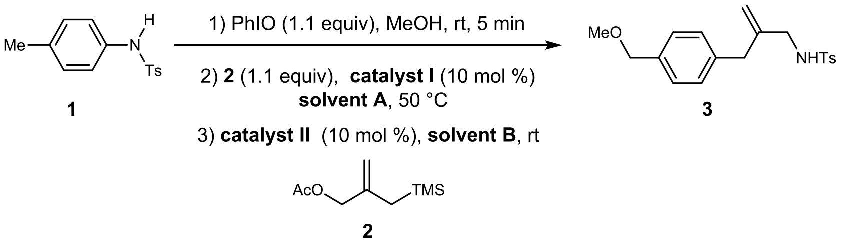

entry	catalyst I	solvent A	catalyst II	solvent B	yield (%)
1	$Pd(PPh_3)_4$	THF	$TsOH·H_2O$	THF	2
2	$Pd(PPh_3)_4$	Toluene	$TsOH·H_2O$	DCE	6
3	$Pd_2(dba)_3$	Toluene	$TsOH·H_2O$	DCE	0
4	$Pd(dba)_2$	Toluene	$TsOH·H_2O$	DCE	0
5	$Pd(dppe)_2$	Toluene	$TsOH·H_2O$	DCE	0
6	$Pd(PPh_3)_2Cl_2$	Toluene	$TsOH·H_2O$	DCE	0
7	$Pd(Pt\text{-}Bu_3)_2$	Toluene	$TsOH·H_2O$	DCE	0
8[a]	$Pd(OAc)_2/PPh_3$	Toluene	$TsOH·H_2O$	DCE	0
9[a]	$Pd(OAc)_2/P(Oi\text{-}Pr)_3$	Toluene	$TsOH·H_2O$	DCE	0
10[a]	$Pd(PPh_3)_2Cl_2/PPh_3$	Toluene	$TsOH·H_2O$	DCE	0
11[a]	$Pd(PPh_3)_4/PPh_3$	Toluene	$TsOH·H_2O$	DCE	0
12[a]	$Pd(PPh_3)_4/PCy_3$	Toluene	$TsOH·H_2O$	DCE	0
13[a]	$Pd(PPh_3)_4$/dppe	Toluene	$TsOH·H_2O$	DCE	0
14[a]	$Pd(PPh_3)_4$/dppb	Toluene	$TsOH·H_2O$	DCE	0
15	$Pd(PPh_3)_4$	Toluene	$Cu(OTf)_2$	DCE	7
16	$Pd(PPh_3)_4$	Toluene	$Sc(OTf)_3$	DCE	7

17	$Pd(PPh_3)_4$	Toluene	$Yb(OTf)_3$	DCE	0
18	$Pd(PPh_3)_4$	Toluene	AgOTf	DCE	0
19	$Pd(PPh_3)_4$	Toluene	$Zn(OTf)_2$	DCE	0
20	$Pd(PPh_3)_4$	Toluene	$Pd(PPh_3)_2Cl_2$	DCE	0
21	$Pd(PPh_3)_4$	Toluene	$Bi(OTf)_3$	DCE	8
22	$Pd(PPh_3)_4$	Toluene	CuI	DCE	0
23	$Pd(PPh_3)_4$	Toluene	$Fe(acac)_3$	DCE	0
24	$Pd(PPh_3)_4$	Toluene	$AuCl_3$	DCE	0
25	$Pd(PPh_3)_4$	Toluene	$In(OTf)_3$	DCE	5
26	$Pd(PPh_3)_4$	Toluene	PhCOOH	DCE	3
27	$Pd(PPh_3)_4$	DCE	$Bi(OTf)_3$	DCE	2
28	$Pd(PPh_3)_4$	1.4-Dioxane	$Bi(OTf)_3$	DCE	13
29	$Pd(PPh_3)_4$	DMF	$Bi(OTf)_3$	DCE	8
30	$Pd(PPh_3)_4$	MeOH	$Bi(OTf)_3$	DCE	0
31	$Pd(PPh_3)_4$	AcOEt	$Bi(OTf)_3$	DCE	15
32	$Pd(PPh_3)_4$	CH_3NO_2	$Bi(OTf)_3$	DCE	0
33	$Pd(PPh_3)_4$	Acetone	$Bi(OTf)_3$	DCE	2
34	$Pd(PPh_3)_4$	MeCN	$Bi(OTf)_3$	DCE	3
35	$Pd(PPh_3)_4$	AcOEt[c]	$Bi(OTf)_3$	DCE	16
36	$Pd(PPh_3)_4$	AcOEt[c,d]	$Bi(OTf)_3$	DCE	19
37	$Pd(PPh_3)_4$	AcOEt[c,e]	$Bi(OTf)_3$	DCE	21
38	$Pd(PPh_3)_4$[b]	AcOEt[c,e]	$Bi(OTf)_3$	DCE	6
39	$Pd(PPh_3)_4$[f]	AcOEt[c,e]	$Bi(OTf)_3$	DCE	<5
40	$Pd(PPh_3)_4$	AcOEt[c,e]	$Bi(OTf)_3$	DCM	18
41	$Pd(PPh_3)_4$	AcOEt[c,e]	$Bi(OTf)_3$	CH_3NO_2	2
42	$Pd(PPh_3)_4$	AcOEt[c,e]	$Bi(OTf)_3$	AcOEt	10
43	$Pd(PPh_3)_4$	AcOEt[c,e]	$Bi(OTf)_3$	MeCN	9
44	$Pd(PPh_3)_4$	AcOEt[c,e]	$Bi(OTf)_3$	MeOH	61
45	$Pd(PPh_3)_4$	AcOEt[c,e]	$Bi(OTf)_3$[g]	MeOH	30
**46**	**$Pd(PPh_3)_4$**	**AcOEt[c,e]**	**$Bi(OTf)_3$[h]**	**MeOH**	**68**
47	$Pd(PPh_3)_4$	AcOEt[c,e]	$Bi(OTf)_3$[i]	MeOH	67

a: Ligand (20 mol %), *b*: $Pd(PPh_3)_4$ (5 mol %), *c*: AcOEt (anhydrous), *d*: reaction temperature (70 ℃), *e*: reaction temperature (80 ℃, reflux), *f*: $Pd(PPh_3)_4$ (1 mol %), *g*: $Bi(OTf)_3$ (5 mol %), *h*: $Bi(OTf)_3$ (12 mol %), *i*: $Bi(OTf)_3$ (15 mol %).

Representative Procedure and Characterization of Products

Representative Procedure

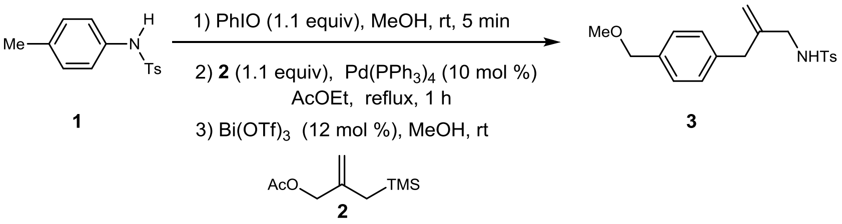

PhIO (0.11 mmol) was added to a solution of compound **1** (0.1 mmol) in MeOH (2.0 mL) at 25 ^{0}C. After 5 min, the reaction mixture was concentrated *in vacuo*, then was passed through a short silica gel column to remove PhI. The resulting product was mixed with a solution of **2** (0.11 mmol) and $Pd(PPh_3)_4$ (0.01 mmol) in anhydrous EtOAc (2.0 mL), and the resulting mixture was stirred at 80 ^{0}C for 1 h. Then the reaction mixture was concentrated *in vacuo*. The resulting crude product was mixed with a solution of $Bi(OTf)_3$ (0.012 mmol) in MeOH (2.0 mL) and stirred at 25 ^{0}C for 12 h. After the substrate was consumed completely (monitored by TLC analysis), the mixture was passed through a short silica gel column and then concentrated under reduced pressure. The residue was purified by flash column chromatography on silica gel (petroleum ether/ethyl acetate = 5/1) to furnish the product **3**.

The procedure for synthesis of compound 44

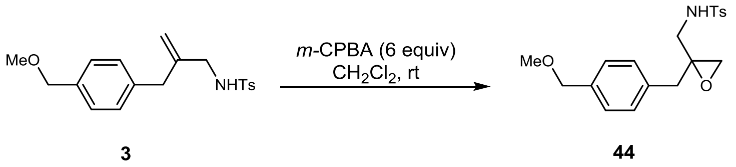

A published epoxidation method was adapted[1]. To a solution of compound **3** (34.5 mg, 0.1 mmol) in CH_2Cl_2 (2 mL) was added *m*-chloroperbenzoic acid (≤77%, 134 mg, 0.6 mmol, 6.0 equivalents) and stirred at room temperature. After compound **3** was consumed completely (monitored by TLC analysis), the mixture was filtered through Celite and washed with CH_2Cl_2. The filtrate was washed with saturated aqueous Na_2SO_3 and the organic layer was separated. The organic layers were washed with saturated aqueous $NaHCO_3$, brine, dried over Na_2SO_4, filtered, and concentrated under reduced pressure. The residue was purified by chromatography on silica gel to afford the desired compound **44**.

The procedure for synthesis of compound 45

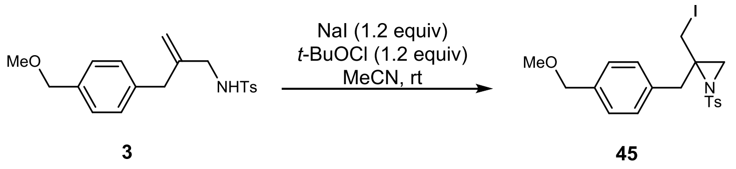

A published iodocyclization method was adapted[2]. To a mixture of **3** (34.5 mg, 0.1 mmol) and NaI (18 mg, 0.12 mmol) in MeCN (0.6 mL) was added *t*-BuOCl (13 mg, 0.12 mmol). The mixture was

allowed to stir in the dark at room temperature for 5 h under an atmosphere of nitrogen. The solvent was evaporated and the residue was purified by chromatography on silica gel to afford the desired compound **45**.

The procedure[3] for synthesis of compound 46

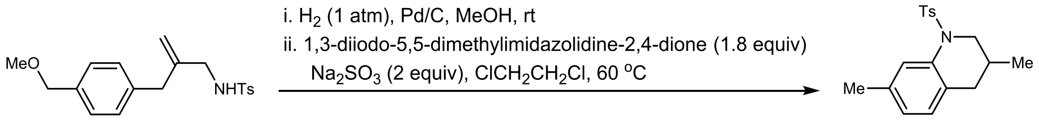

3 **46**

To a mixture of **3** (34.5 mg, 0.1 mmol) in MeOH (2 mL) was added Pd/C (10 mol %). The mixture was allowed to stir at room temperature overnight under an atmosphere of Hydrogen (1 atm). After compound **3** was consumed completely (monitored by TLC analysis), the mixture was filtered through a celite pad and purified by chromatography on silica gel to afford the desired reduction product. A mixture of reduction product and Na_2SO_3 (2.0 equiv), 1,3-Diiodo-5,5-dimethylhydantoin (DIH, 1.8 equiv) was sealed and flushed with N_2. 1,2-DCE (1.5 mL) was added, and the resulting yellow mixture was heated at 60 °C for 2 h, affording a dark red heterogenous solution. The reaction was quenched with saturated solution of Na_2SO_3. The layers were separated and the aqueous layer was extracted with CH_2Cl_2. The combined organic fractions were dried over Na_2SO_4 and concentrated under vacuum. The resulting residue was purified by flash chromatography, providing the pure compound **46**.

The procedure[4] for synthesis of compound 47

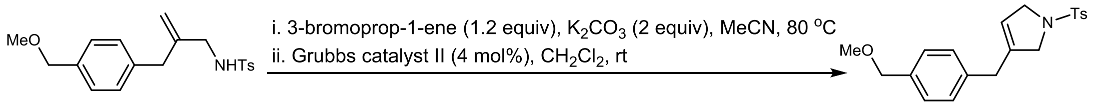

3 **47**

To a solution of **3** (34.5 mg, 0.1 mmol) and K_2CO_3 (27.6 mg, 0.2 mmol) in MeCN (1 mL) was added allyl bromide (14.4 mg, 0.12 mmol), and the mixture was refluxed for 12 h. The solution was filtered through a celite pad and the filtrate was concentrated under reduced pressure. Purification by flush column chromatography on silica gel gave the allylation product. The allylation product and Grubbs 2:nd generation catalyst (4 mol %) was dissolved in dry dichloromethane (5 mL) and the solution stirred at room temperature overnight. When the reaction was complete according to TLC, the solvent was removed and the crude material purified by column chromatography for producing compound **47**.

The procedure[5] for synthesis of compound 48

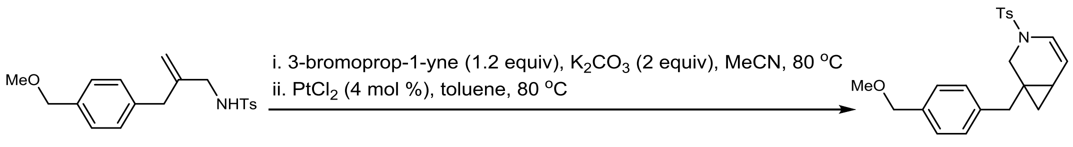

3 **48**

To a solution of **3** (34.5 mg, 0.1 mmol) and K_2CO_3 (27.6 mg, 0.2 mmol) in MeCN (1 mL) was added propargyl bromide (14.4 mg, 0.12 mmol), and the mixture was refluxed for 12 h. The solution was filtered through a celite pad and the filtrate was concentrated under reduced pressure. Purification by flush column chromatography on silica gel gave the propargylation product. Then, $PtCl_2$ (4 mol %)

was added to a solution of the propargylation product in toluene (5 mL) and the resulting mixture was stirred at 80 ^{0}C. When the reaction was complete according to TLC, the solvent was removed and the crude material purified by column chromatography for producing compound **48**.

The procedure[6] for synthesis of compound 49

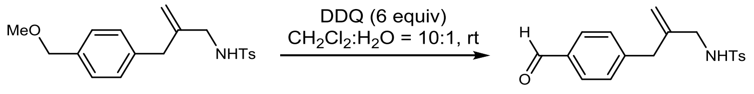

3 49

To a solution of **3** (34.5 mg, 0.1 mmol) in CH_2Cl_2 (6 mL) and water (0.6 mL) at room temperature was added solid DDQ (134 mg, 0.25 mmol). When the reaction was complete according to TLC, the reaction was quenched by addition of saturated aqueous $NaHCO_3$ and extracted with ether. The organic extract was washed with brine, dried over Na_2SO_4, filtered and concentrated. The crude material purified by column chromatography for producing compound **49**.

The procedure for synthesis of compound 50

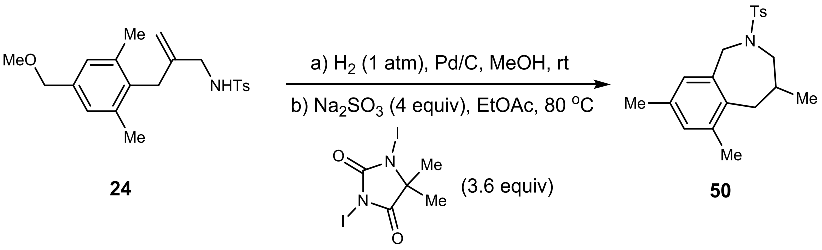

24 50

To a mixture of compound **24** (37.3 mg, 0.1 mmol) in MeOH (2 mL) was added Pd/C (10 mol %). The mixture was allowed to stir at room temperature overnight under an atmosphere of Hydrogen (1 atm). After compound **24** was consumed completely (monitored by TLC analysis), the mixture was filtered through a celite pad and purified by chromatography on silica gel to afford the desired reduction product. A mixture of reduction product and Na_2SO_3 (4 equiv), 1,3-Diiodo-5,5-dimethylhydantoin (DIH, 3.6 equiv) was sealed and flushed with N_2. EtOAc (1.5 mL) was added, and the resulting yellow mixture was heated at 80 °C. When the reaction was complete according to TLC, the reaction was quenched with saturated solution of Na_2SO_3. The layers were separated and the aqueous layer was extracted with CH_2Cl_2. The combined organic fractions were dried over Na_2SO_4 and concentrated under vacuum. The resulting residue was purified by flash chromatography, providing the pure compound **50**.

The procedure[7,8] for synthesis of compound 51

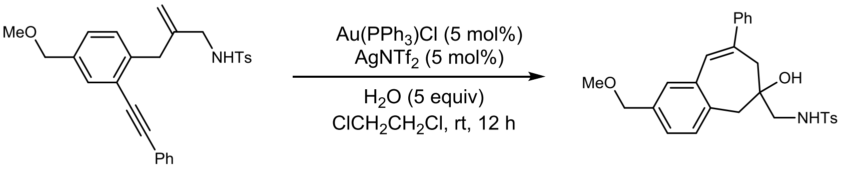

13 51

Au(PPh_3)Cl (0.005 mmol), $AgNTf_2$ (0.005 mmol) and H_2O (0.5 mmol) was added to a solution of compound **13** (0.1 mmol) in dichloroethane (2.0 mL) under N_2. The mixture was stirred at 25 °C for 12 h. After the substrate was completely consumed (monitored by TLC analysis), the mixture was

passed through a short silica gel column and then concentrated under reduced pressure. The residue was purified by flash column chromatography on silica gel to furnish compound **51**.

Characterization of Products

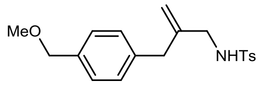

N-(2-(4-(methoxymethyl)benzyl)allyl)-4-methylbenzenesulfonamide **3** (23 mg, 68%). White solid; mp: 80-81 °C; ^{1}H NMR (400 MHz, $CDCl_3$): δ 7.70 (d, J = 8.2 Hz, 2 H), 7.28 (d, J = 8.1 Hz, 2 H), 7.22 (d, J = 7.9 Hz, 2 H), 7.06 (d, J = 7.9 Hz, 2 H), 4.99 (s, 1 H), 4.85 (s, 1 H), 4.65 (t, J = 6.3 Hz, 1 H), 4.41 (s, 2 H), 3.45 (d, J = 6.4 Hz, 2 H), 3.38 (s, 3 H), 3.30 (s, 2 H), 2.42 (s, 3 H); ^{13}C NMR (100 MHz, $CDCl_3$): δ 143.8, 143.4, 137.8, 136.8, 136.3, 129.6, 128.9, 127.9, 127.1, 114.2, 74.4, 58.1, 47.2, 40.0, 21.5; HRMS (m/z): [M+H]+ calcd. for $C_{19}H_{23}NO_3S$, 346.1471; found, 346.1478.

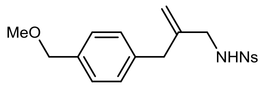

N-(2-(4-(methoxymethyl)benzyl)allyl)-4-nitrobenzenesulfonamide **3b** (17 mg, 45%). Yellow liquid; ^{1}H NMR (400 MHz, $CDCl_3$): δ 8.31 (d, J = 8.4 Hz, 2 H), 7.96 (d, J = 8.5 Hz, 2 H), 7.22 (d, J = 7.6 Hz, 2 H), 7.06 (d, J = 7.7 Hz, 2 H), 5.08 (t, J = 5.7 Hz, 1 H), 4.99 (s, 1 H), 4.91 (s, 1 H), 4.41 (s, 2 H), 3.52 (d, J = 5.9 Hz, 2 H), 3.40 (s, 3 H), 3.30 (s, 2 H); ^{13}C NMR (100 MHz, $CDCl_3$): δ 149.9, 145.8, 143.1, 137.4, 136.5, 128.8, 128.2, 128.0, 124.3, 114.6, 74.4, 58.2, 47.0, 40.1; HRMS (m/z): $[M+Na]^{+}$ calcd. for $C_{18}H_{20}N_2O_5S$, 399.0985; found, 399.0989.

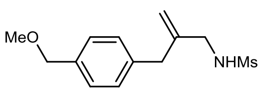

N-(2-(4-(methoxymethyl)benzyl)allyl)methanesulfonamide **3c** (11 mg, 40%). Yellow liquid; ^{1}H NMR (400 MHz, $CDCl_3$): δ 7.28 (d, J = 7.5 Hz, 2 H), 7.17 (d, J = 7.3 Hz, 2 H), 5.14 (s, 1 H), 4.99 (s, 1 H), 4.59 (s, 1 H), 4.43 (s, 2 H), 3.64 (d, J = 5.9 Hz, 2 H), 3.41 (s, 2 H), 3.39 (s, 3 H), 2.90 (s, 3 H); ^{13}C NMR (100 MHz, $CDCl_3$): δ 144.1, 137.6, 136.5, 128.9, 128.1, 114.1, 74.4, 58.1, 47.0, 40.6, 40.2; HRMS (m/z): $[M+Na]^{+}$ calcd. for $C_{13}H_{19}NO_3S$, 292.0978; found, 292.0987.

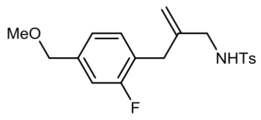

N-(2-(2-fluoro-4-(methoxymethyl)benzyl)allyl)-4-methylbenzenesulfonamide **4** (30 mg, 83%). Yellow liquid; ^{1}H NMR (400 MHz, $CDCl_3$): δ 7.73 (d, J = 7.7 Hz, 2 H), 7.29 (d, J = 7.8 Hz, 2 H), 7.07 (t, J = 7.6 Hz, 1 H), 7.01-6.98 (m, 2 H), 4.99 (s, 1 H), 4.78 (s, 1 H), 4.73 (t, J = 6.2 Hz, 1 H), 4.40 (s, 2 H), 3.51 (d, J = 6.3 Hz, 2 H), 3.39 (s, 3 H), 3.31 (s, 2 H), 2.42 (s, 3 H); ^{13}C NMR (100 MHz, $CDCl_3$) δ 161.0 (d, J (C,F) = 244.6 Hz), 143.4, 142.4, 139.4 (d, J (C,F) = 7.2 Hz), 136.8, 131.0 (d, J (C,F) = 4.7 Hz), 129.6, 127.1, 124.5 (d, J (C,F) = 15.9 Hz), 123.1 (d, J (C,F) = 3.2 Hz), 114.4 (d, J (C,F) = 22.5 Hz), 114.2, 73.7, 58.2, 47.5, 32.8, 21.5; HRMS (m/z): $[M+Na]^{+}$ calcd. for $C_{19}H_{22}FNO_3S$, 386.1197; found, 386.1212.

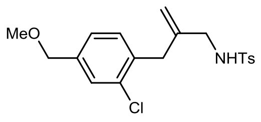

N-(2-(2-chloro-4-(methoxymethyl)benzyl)allyl)-4-methylbenzenesulfonamide **5** (27 mg, 71%). Yellow liquid; ^{1}H NMR (400 MHz, $CDCl_3$): δ 7.74 (d, J = 8.0 Hz, 2 H), 7.32 (s, 1 H), 7.30 (d, J = 8.0 Hz, 2 H), 7.14 (d, J = 8.0 Hz, 1 H), 7.10 (d, J = 7.8 Hz, 1 H), 5.01 (s, 1 H), 4.71-4.68 (m, 2 H), 4.39 (s, 2 H), 3.53 (d, J = 6.4 Hz, 2 H), 3.40 (s, 2 H), 3.39 (s,

3 H), 2.43 (s, 3 H); ^{13}C NMR (100 MHz, CDCl$_3$): δ 143.5, 142.1, 138.4, 136.8, 135.3, 134.3, 131.0, 129.7, 128.6, 127.1, 126.0, 114.5, 73.6, 58.3, 47.8, 37.1, 21.5; HRMS (m/z): [M+Na]$^+$ calcd. for $C_{19}H_{22}ClNO_3S$, 402.0901; found, 402.0880.

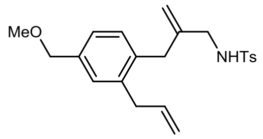

N-(2-(2-allyl-4-(methoxymethyl)benzyl)allyl)-4-methylbenzenesulfonamide **6** (27 mg, 70%). Yellow liquid; ^{1}H NMR (400 MHz, CDCl$_3$): δ 7.73 (d, *J* = 8.0 Hz, 2 H), 7.30 (d, *J* = 7.9 Hz, 2 H), 7.12-6.99 (m, 2 H), 7.00 (d, *J* = 7.5 Hz, 1 H), 5.93-5.83 (m, 1 H), 5.03 (d, *J* = 10.0 Hz, 1 H), 4.99 (s, 1 H), 4.95 (d, *J* = 18.0 Hz, 1 H), 4.62 (s, 1 H), 4.55 (t, *J* = 6.2 Hz, 1 H), 4.40 (s, 2 H), 3.51 (d, *J* = 6.3 Hz, 2 H), 3.39 (s, 3 H), 3.29 (s, 2 H), 3.27 (d, *J* = 6.4 Hz, 2 H), 2.43 (s, 3 H); ^{13}C NMR (100 MHz, CDCl$_3$): δ 143.5, 143.4, 138.4, 136.8, 136.6, 135.6, 130.2, 129.7, 129.2, 127.1, 125.9, 115.9, 114.1, 74.5, 58.2, 47.9, 36.9, 36.8, 21.5; HRMS (m/z): [M+Na]$^+$ calcd. for $C_{22}H_{27}NO_3S$, 408.1604; found, 408.1600.

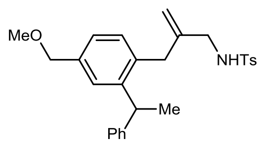

N-(2-(4-(methoxymethyl)-2-(1-phenylethyl)benzyl)allyl)-4-methylbenzenesulfonamide **7** (28 mg, 63%). Yellow liquid; ^{1}H NMR (400 MHz, CDCl$_3$): δ 7.70 (d, *J* = 7.9 Hz, 2 H), 7.29-7.20 (m, 5 H), 7.16-7.07 (m, 4 H), 6.99 (d, *J* = 7.7 Hz, 1 H), 4.96 (s, 1 H), 4.60 (s, 1 H), 4.44-4.42 (m, 3 H), 4.21 (q, *J* = 7.0 Hz, 1 H), 3.46-3.35 (m, 5 H), 3.26 (d, *J* = 16.5 Hz, 1 H), 3.16 (d, *J* = 16.4 Hz, 1 H), 2.41 (s, 3 H), 1.54 (d, *J* = 7.1 Hz, 3 H); ^{13}C NMR (100 MHz, CDCl$_3$): δ 146.2, 144.1, 143.6, 143.4, 136.9, 136.6, 135.3, 130.5, 129.6, 128.3, 127.5, 127.1, 126.8, 125.9, 125.8, 114.2, 74.7, 58.1, 47.7, 40.2, 36.8, 22.4, 21.5; HRMS (m/z): [M+Na]$^+$ calcd. for $C_{27}H_{31}NO_3S$, 472.1917; found, 472.1920.

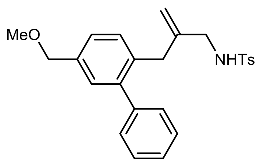

N-(2-((5-(methoxymethyl)-[1,1'-biphenyl]-2-yl)methyl)allyl)-4-methylbenzenesulfonamide **8** (33 mg, 78%). Pale yellow liquid; ^{1}H NMR (400 MHz, CDCl$_3$): δ 7.63 (d, *J* = 8.2 Hz, 2 H), 7.39-7.32 (m, 3 H), 7.26-7.19 (m, 6 H), 7.13 (d, *J* = 7.8 Hz, 1 H), 4.92 (s, 1 H), 4.55 (s, 1 H), 4.45 (s, 2 H), 4.29 (t, *J* = 6.3 Hz, 1 H), 3.41 (s, 3 H), 3.34 (d, *J* = 6.3 Hz, 2 H), 3.18 (s, 2 H), 2.41 (s, 3 H); ^{13}C NMR (100 MHz, CDCl$_3$): δ 144.1, 143.3, 142.2, 141.2, 136.8, 136.4, 135.0, 130.3, 129.5, 129.0, 128.0, 127.1, 127.0, 126.8, 114.3, 74.4, 58.2, 47.6, 37.0, 21.5; HRMS (m/z): [M+H]$^+$ calcd. for $C_{25}H_{27}NO_3S$, 422.1784; found, 422.1775.

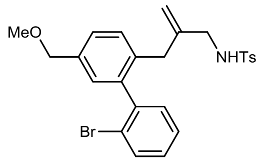

N-(2-((2'-bromo-5-(methoxymethyl)-[1,1'-biphenyl]-2-yl)methyl)allyl)-4-methylbenzenesulfonamide **9** (27 mg, 55%). Yellow liquid; ^{1}H NMR (400 MHz, CDCl$_3$): δ 7.62 (d, *J* = 7.9 Hz, 3 H), 7.33-7.20 (m, 5 H), 7.14 (t, *J* = 8.1 Hz, 2 H), 7.09 (s, 1 H), 4.89 (s, 1 H), 4.53 (s, 1 H), 4.45 (s, 2 H), 4.25 (t, *J* = 6.2 Hz, 1 H), 3.40 (s, 3 H), 3.35-3.22 (m, 2 H), 3.13 (d, *J* = 15.8 Hz, 1 H), 2.99 (d, *J* = 15.8 Hz, 1 H), 2.42 (s, 3 H); ^{13}C NMR (100 MHz, CDCl$_3$): δ 143.3, 143.0, 141.6, 141.0, 136.8, 136.3, 135.5, 132.5, 131.2, 129.7, 129.6, 129.3, 129.0, 127.4, 127.1, 127.0, 123.8, 114.7, 74.2, 58.2, 47.3, 37.2, 21.5; HRMS (m/z): [M+Na]$^+$ calcd. for $C_{25}H_{26}BrNO_3S$, 522.0709; found, 522.0715.

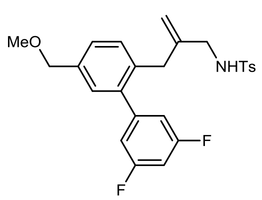

N-(2-((3',5'-difluoro-5-(methoxymethyl)-[1,1'-biphenyl]-2-yl)methyl)allyl)-4-methylbenzenesulfonamide **10** (28 mg, 62%). Yellow liquid; ^{1}H NMR (400 MHz, $CDCl_3$): δ 7.67 (d, J = 7.9 Hz, 2 H), 7.28 (d, J = 8.1 Hz, 3 H), 7.17 (s, 1 H), 7.14 (d, J = 7.8 Hz, 1 H), 6.80-6.74 (m, 3 H), 4.97 (s, 1 H), 4.56-4.54 (m, 2 H), 4.45 (s, 2 H), 3.42 (s, 3 H), 3.39 (d, J = 6.3 Hz, 2 H), 3.20 (s, 2 H), 2.42 (s, 3 H); ^{13}C NMR (100 MHz, $CDCl_3$): δ 162.6 (d, J (C,F) = 247.4 Hz), 162.4 (d, J (C,F) = 247.4 Hz), 144.4 (t, J (C,F) =9.4 Hz), 144.0, 143.5, 140.0, 136.7, 134.7, 130.6, 129.6, 129.0, 127.5, 127.0, 114.6, 112.0 (dd, J (C,F) = 18.2 Hz, 6.8 Hz), 102.5 (t, J (C,F) = 25.0 Hz), 74.2, 58.3, 47.7, 37.0, 21.4; HRMS (m/z): $[M+Na]^+$ calcd. for $C_{25}H_{25}F_2NO_3S$, 480.1415; found, 480.1423.

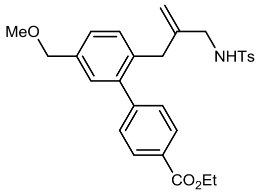

Ethyl 5'-(methoxymethyl)-2'-(2-(((4-methylphenyl)sulfonamido)methyl)allyl)-[1,1'-biphenyl]-4-carboxylate **11** (35 mg, 72%). Yellow liquid; ^{1}H NMR (400 MHz, $CDCl_3$): δ 8.04 (d, J = 8.1 Hz, 2 H), 7.65 (d, J = 8.2 Hz, 2 H), 7.30-7.26 (m, 5 H), 7.19 (s, 1 H), 7.15 (d, J = 7.8 Hz, 1 H), 4.94 (s, 1 H), 4.53 (s, 1 H), 4.47-4.45 (m, 3 H), 4.41 (q, J = 7.1 Hz, 2 H), 3.42 (s, 3 H), 3.34 (d, J = 6.4 Hz, 2 H), 3.20 (s, 2 H), 2.42 (s, 3 H), 1.42 (t, J = 7.1 Hz, 3 H); ^{13}C NMR (100 MHz, $CDCl_3$): δ 166.4, 145.9, 143.9, 143.4, 141.3, 136.7, 136.6, 134.8, 130.4, 129.6, 129.3, 129.2, 129.1, 129.0, 127.3, 127.0, 114.4, 74.3, 61.0, 58.3, 47.6, 37.0, 21.5, 14.3; HRMS (m/z): $[M+Na]^+$ calcd. for $C_{28}H_{31}NO_5S$, 516.1815; found, 516.1828.

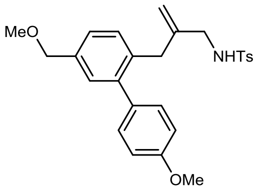

N-(2-((4'-methoxy-5-(methoxymethyl)-[1,1'-biphenyl]-2-yl)methyl)allyl)-4-methylbenzenesulfonamide **12** (32 mg, 70%). Pale yellow solid; mp: 95-96 °C; ^{1}H NMR (400 MHz, $CDCl_3$): δ 7.64 (d, J = 8.1 Hz, 2 H), 7.26 (d, J = 7.9 Hz, 2 H), 7.23-7.10 (m, 5 H), 6.91 (d, J = 8.5 Hz, 2 H), 4.92 (s, 1 H), 4.56 (s, 1 H), 4.44 (s, 2 H), 4.25 (t, J = 6.2 Hz, 1 H), 3.86 (s, 3 H), 3.41 (s, 3 H), 3.35 (d, J = 6.2 Hz, 2 H), 3.19 (s, 2 H), 2.42 (s, 3 H); ^{13}C NMR (100 MHz, $CDCl_3$): δ 158.7, 144.2, 143.3, 141.9, 136.8, 136.3, 135.2, 133.5, 130.3, 130.1, 129.7, 129.6, 127.0, 126.6, 114.3, 113.5, 74.4, 58.2, 55.3, 47.7, 37.0, 21.5; HRMS (m/z): $[M+Na]^+$ calcd. for $C_{26}H_{29}NO_4S$, 474.1710; found, 474.1691.

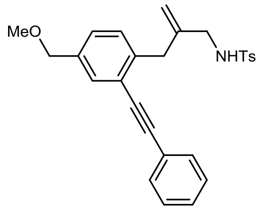

N-(2-(4-(methoxymethyl)-2-(phenylethynyl)benzyl)allyl)-4-methylbenzenesulfonamide **13** (32 mg, 73%). Yellow solid; mp: 71-72 °C; ^{1}H NMR (400 MHz, $CDCl_3$): δ 7.67 (d, J = 8.2 Hz, 2 H), 7.50-7.47 (m, 3 H), 7.35-7.33 (m, 3 H), 7.21-7.18 (m, 3 H), 7.12 (d, J = 7.9 Hz, 1 H), 5.01-4.99 (m, 2 H), 4.82 (s, 1 H), 4.40 (s, 2 H), 3.54 (s, 2 H), 3.52 (d, J = 6.5 Hz, 2 H), 3.37 (s, 3 H), 2.35 (s, 3 H); ^{13}C NMR (100 MHz, $CDCl_3$): δ 143.2, 142.9, 139.6, 136.7, 136.4, 131.5, 131.4, 129.5, 129.4, 128.3, 128.2, 127.8, 126.9, 123.0, 122.9, 114.0, 93.2, 87.8, 73.8, 58.0, 47.4, 38.3, 21.3; HRMS (m/z): $[M+H]^+$ calcd. for $C_{27}H_{27}NO_3S$, 446.1784; found, 446.1774.

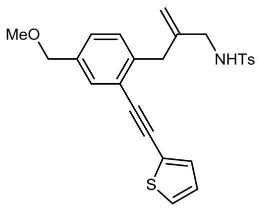

N-(2-(4-(methoxymethyl)-2-(thiophen-2-ylethynyl)benzyl)allyl)-4-methylbenzenesulfonamide **14** (32 mg, 70%). Yellow solid; mp: 78-79 °C; ^{1}H NMR (400 MHz, $CDCl_3$): δ 7.68 (d, J = 8.2 Hz, 2 H), 7.45 (s, 1 H), 7.29 (d, J = 5.1 Hz, 1 H), 7.26-7.20 (m, 4 H), 7.12 (d, J = 7.9 Hz, 1 H), 7.01 (dd, J = 5.1 Hz, 3.7 Hz, 1 H), 5.01 (s, 1 H), 4.83 (s, 1 H), 4.78 (t, J = 6.4 Hz, 1 H), 4.40 (s, 2 H), 3.52 (d, J = 6.4 Hz, 2 H), 3.50 (s, 2 H), 3.38 (s, 3 H), 2.38 (s, 3 H); ^{13}C NMR (100 MHz, $CDCl_3$): δ 143.3, 142.8, 139.4, 136.8, 136.6, 131.8, 131.3, 129.6, 128.0, 127.4, 127.2, 127.0, 122.9, 122.7, 114.3, 91.6, 86.4, 73.8, 58.1, 47.4, 38.4, 21.4; HRMS (m/z): $[M+H]^+$ calcd. for $C_{25}H_{25}NO_3S_2$, 452.1349; found, 452.1347.

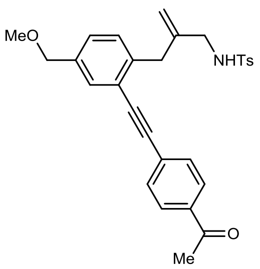

N-(2-(2-((4-acetylphenyl)ethynyl)-4-(methoxymethyl)benzyl)allyl)-4-methylbenzenesulfonamide **15** (36 mg, 74%). Yellow solid; mp: 101-102 °C; ^{1}H NMR (400 MHz, $CDCl_3$): δ 7.94 (d, J = 8.2 Hz, 2 H), 7.68 (d, J = 8.2 Hz, 2 H), 7.58 (d, J = 8.2 Hz, 2 H), 7.51 (s, 1 H), 7.27-7.22 (m, 3 H), 7.16 (d, J = 7.9 Hz, 1 H), 5.03 (s, 1 H), 4.83-4.80 (m, 2 H), 4.43 (s, 2 H), 3.58 (s, 2 H), 3.54 (d, J = 6.4 Hz, 2 H), 3.40 (s, 3 H), 2.61 (s, 3 H), 2.38 (s, 3 H); ^{13}C NMR (100 MHz, $CDCl_3$): δ 197.3, 143.4, 143.0, 139.8, 136.8, 136.7, 136.2, 131.7, 131.6, 129.6, 128.4, 128.3, 127.9, 127.0, 122.5, 114.3, 92.4, 91.2, 73.8, 58.2, 47.5, 38.4, 26.6, 21.4; HRMS (m/z): $[M+H]^+$ calcd. for $C_{29}H_{29}NO_4S$, 488.1890; found, 488.1887.

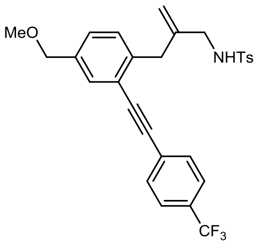

N-(2-(4-(methoxymethyl)-2-((4-(trifluoromethyl)phenyl)ethynyl)benzyl)allyl)-4-methylbenzenesulfonamide **16** (38 mg, 75%). Yellow solid; mp: 89-90 °C; ^{1}H NMR (400 MHz, $CDCl_3$): δ 7.68 (d, J = 8.2 Hz, 2 H), 7.62-7.58 (m, 4 H), 7.51 (s, 1 H), 7.26-7.21 (m, 3 H), 7.15 (d, J = 7.9 Hz, 1 H), 5.02 (s, 1 H), 4.81-4.78 (m, 2 H), 4.43 (s, 2 H), 3.57 (s, 2 H), 3.53 (d, J = 6.4 Hz, 2 H), 3.40 (s, 3 H), 2.38 (s, 3 H); ^{13}C NMR (100 MHz, $CDCl_3$): δ 143.4, 143.0, 139.8, 136.7, 131.7, 130.0 (q, J (C,F) = 32.5 Hz), 129.6, 128.4, 127.0, 126.9, 125.4, 125.3, 125.2, 123.9 (q, J (C,F) = 270.6 Hz), 122.4, 114.3, 91.7, 90.2, 73.8, 58.2, 47.6, 38.4, 21.4; HRMS (m/z): $[M+H]^+$ calcd. for $C_{28}H_{26}F_3NO_3S$, 514.1658; found, 514.1676.

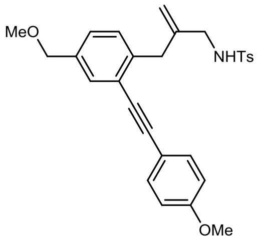

N-(2-(4-(methoxymethyl)-2-((4-methoxyphenyl)ethynyl)benzyl)allyl)-4-methylbenzenesulfonamide **17** (31 mg, 66%). Yellow solid; mp: 94-95 °C; ^{1}H NMR (400 MHz, $CDCl_3$): δ 7.66 (d, J = 8.2 Hz, 2 H), 7.46-7.42 (m, 3 H), 7.22-7.18 (m, 3 H), 7.11 (d, J = 7.9 Hz, 1 H), 6.88 (d, J = 8.8 Hz, 2 H), 5.00 (s, 1 H), 4.84 (s, 1 H), 4.73 (t, J = 6.4 Hz, 1 H), 4.41 (s, 2 H), 3.82 (s, 3 H), 3.54-3.52 (m, 4 H), 3.38 (s, 3 H), 2.37 (s, 3 H); ^{13}C NMR (100 MHz, $CDCl_3$): δ 159.7, 143.2, 143.1, 139.3, 136.8, 136.5, 132.9, 131.4, 129.6, 129.4, 127.5, 127.0, 123.4, 115.1, 114.1, 114.0, 93.3, 86.6, 73.9, 58.1, 55.3, 47.5, 38.4, 21.4; HRMS (m/z): $[M+H]^+$ calcd. for $C_{28}H_{29}NO_4S$, 476.1890; found, 476.1878.

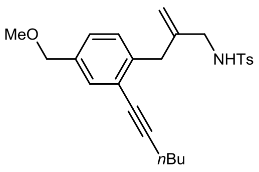

N-(2-(2-(hex-1-yn-1-yl)-4-(methoxymethyl)benzyl)allyl)-4-methylbenzenesulfonamide **18** (30 mg, 71%). Yellow oil; ^{1}H NMR (400 MHz, $CDCl_3$): δ 7.71 (d, J = 8.2 Hz, 2 H), 7.33 (s, 1 H), 7.27 (d, J = 8.0 Hz, 2 H), 7.13 (d, J = 7.8 Hz, 1 H), 7.05 (d, J = 7.8 Hz, 1 H), 4.97 (s, 1 H), 4.88 (t, J = 6.3 Hz, 1 H), 4.78 (s, 1 H), 4.37 (s, 2 H), 3.48 (d, J = 6.3 Hz, 2 H), 3.44 (s, 2 H), 3.36 (s, 3 H), 2.44-2.41 (m, 5 H), 1.61-1.54 (m, 2 H), 1.50-1.41 (m, 2 H), 0.94 (t, J = 7.3 Hz, 3 H); ^{13}C NMR (100 MHz, $CDCl_3$): δ 143.2, 143.1, 139.3, 136.9, 136.3, 131.6, 129.5, 129.2, 127.1, 127.0, 123.8, 114.0, 94.5, 79.1, 73.9, 58.0, 47.4, 38.2, 30.8, 22.0, 21.4, 19.1, 13.5; HRMS (m/z): $[M+H]^+$ calcd. for $C_{25}H_{31}NO_3S$, 426.2097; found, 426.2093.

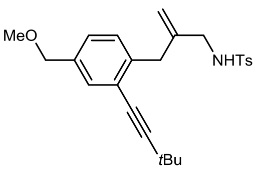

N-(2-(2-(3,3-dimethylbut-1-yn-1-yl)-4-(methoxymethyl)benzyl)allyl)-4-methylbenzene sulfonamide **19** (28 mg, 67%). Yellow solid; mp: 63-64 °C; ^{1}H NMR (400 MHz, $CDCl_3$): δ 7.70 (d, J = 8.2 Hz, 2 H), 7.33 (s, 1 H), 7.27 (d, J = 8.0 Hz, 2 H), 7.13 (dd, J = 7.8 Hz, 1.2 Hz, 1 H), 7.04 (d, J = 7.8 Hz, 1 H), 4.99 (s, 1 H), 4.83 (s, 1 H), 4.74 (t, J = 6.3 Hz, 1 H), 4.37 (s, 2 H), 3.48 (d, J = 6.4 Hz, 2 H), 3.45 (s, 2 H), 3.36 (s, 3 H), 2.41 (s, 3 H), 1.29 (s, 9 H); ^{13}C NMR (100 MHz, $CDCl_3$): δ 143.3, 143.1, 139.1, 136.9, 136.3, 131.6, 129.5, 129.1, 127.1, 123.8, 113.8, 102.6, 77.5, 74.0, 58.0, 47.2, 38.3, 31.0, 21.4; HRMS (m/z): $[M+H]^+$ calcd. for $C_{25}H_{31}NO_3S$, 426.2097; found, 426.2095.

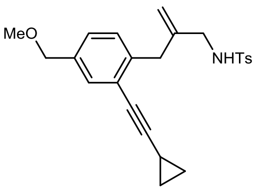

N-(2-(2-(cyclopropylethynyl)-4-(methoxymethyl)benzyl)allyl)-4-methylbenzenesulfona mide **20** (26 mg, 63%). Yellow solid; mp: 67-68 °C; ^{1}H NMR (400 MHz, $CDCl_3$): δ 7.72 (d, J = 8.2 Hz, 2 H), 7.31 (s, 1 H), 7.28 (d, J = 8.0 Hz, 2 H), 7.13 (d, J = 7.8 Hz, 1 H), 7.04 (d, J = 7.9 Hz, 1 H), 4.97 (s, 1 H), 4.82-4.78 (m, 2 H), 4.36 (s, 2 H), 3.48 (d, J = 6.3 Hz, 2 H), 3.42 (s, 2 H), 3.36 (s, 3 H), 2.42 (s, 3 H), 1.50-1.44 (m, 1 H), 0.91-0.86 (m, 2 H), 0.80-0.76 (m, 2 H); ^{13}C NMR (100 MHz, $CDCl_3$): δ 143.3, 143.1, 139.4, 136.9, 136.3, 131.6, 129.6, 129.2, 127.1, 127.0, 123.7, 114.1, 97.6, 74.3, 73.9, 58.0, 47.4, 38.2, 21.4,
8.7, 0.2; HRMS (m/z): $[M+H]^+$ calcd. for $C_{24}H_{27}NO_3S$, 410.1784; found, 410.1784.

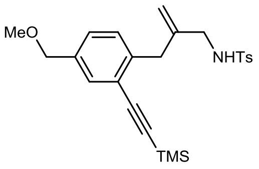

N-(2-(4-(methoxymethyl)-2-((trimethylsilyl)ethynyl)benzyl)allyl)-4-methylbenzenesulf onamide **21** (30 mg, 69%). Yellow solid; mp: 62-63 °C; ^{1}H NMR (400 MHz, $CDCl_3$): δ 7.71 (d, J = 8.2 Hz, 2 H), 7.41 (d, J = 1.3 Hz, 1 H), 7.27 (d, J = 7.8 Hz, 2 H), 7.19 (dd, J = 7.9 Hz, 1.6 Hz, 1 H), 7.08 (d, J = 7.8 Hz, 1 H), 4.99 (s, 1 H), 4.83 (s, 1 H), 4.79 (t, J = 6.4 Hz, 1 H), 4.38 (s, 2 H), 3.48-3.47 (m, 4 H), 3.36 (s, 3 H), 2.41 (s, 3 H), 0.24 (s, 9 H); ^{13}C NMR (100 MHz, $CDCl_3$): δ 143.3, 142.8, 139.9, 136.9, 136.4, 131.9, 129.6, 129.3, 128.0, 127.1, 122.9, 114.2, 103.6, 98.4, 73.8, 58.1, 47.2, 38.2, 21.4, -0.1; HRMS (m/z): $[M+H]^+$ calcd. for $C_{24}H_{31}NO_3SSi$, 442.1867; found, 442.1871.

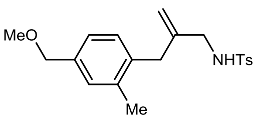

N-(2-(4-(methoxymethyl)-2-methylbenzyl)allyl)-4-methylbenzenesulfonamide **23** (25 mg, 71%). Pale yellow liquid; ^{1}H NMR (400 MHz, $CDCl_3$): δ 7.73 (d, J = 8.2 Hz, 2 H),

7.30 (d, *J* = 8.1 Hz, 2 H), 7.11 (s, 1 H), 7.07 (d, *J* = 7.8 Hz, 1 H), 6.98 (d, *J* = 7.7 Hz, 1 H), 4.97 (s, 1 H), 4.61 (s, 1 H), 4.57 (t, *J* = 6.3 Hz, 1 H), 4.39 (s, 2 H), 3.52 (d, *J* = 6.4 Hz, 2 H), 3.39 (s, 3 H), 3.28 (s, 2 H), 2.43 (s, 3 H), 2.18 (s, 3 H); ^{13}C NMR (100 MHz, $CDCl_3$): δ 143.5, 142.9, 136.9, 136.8, 136.4, 135.8, 129.9, 129.8, 129.7, 127.1, 125.5, 113.9, 74.5, 58.1, 47.9, 37.5, 21.5, 19.2; HRMS (m/z): $[M+H]^+$ calcd. for $C_{20}H_{25}NO_3S$, 360.1628; found, 360.1626.

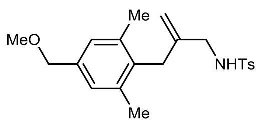

N-(2-(4-(methoxymethyl)-2,6-dimethylbenzyl)allyl)-4-methylbenzenesulfonamide **24** (24 mg, 64%). White solid; mp: 102-103 °C; ^{1}H NMR (400 MHz, $CDCl_3$): δ 7.78 (d, *J* = 8.2 Hz, 2 H), 7.31 (d, *J* = 8.1 Hz, 2 H), 6.97 (s, 2 H), 4.83 (s, 1 H), 4.77 (t, *J* = 6.5 Hz, 1 H), 4.36 (s, 2 H), 4.28 (s, 1 H), 3.62 (d, *J* = 6.5 Hz, 2 H), 3.39 (s, 3 H), 3.25 (s, 2 H), 2.43 (s, 3 H), 2.15 (s, 6 H); ^{13}C NMR (100 MHz, $CDCl_3$): δ 143.5, 141.8, 137.1, 136.8, 135.9, 134.6, 129.7, 127.5, 127.1, 112.2, 74.6, 58.1, 48.8, 33.3, 21.5, 19.7; HRMS (m/z): $[M+H]^+$ calcd. for $C_{21}H_{27}NO_3S$, 374.1784; found, 374.1788.

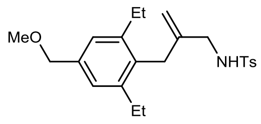

N-(2-(2,6-diethyl-4-(methoxymethyl)benzyl)allyl)-4-methylbenzenesulfonamide **25** (18 mg, 45%). Pale yellow liquid; ^{1}H NMR (400 MHz, $CDCl_3$): δ 7.79 (d, *J* = 8.3 Hz, 2 H), 7.32 (d, *J* = 8.0 Hz, 2 H), 7.01 (s, 2 H), 4.84 (s, 1 H), 4.69 (t, *J* = 6.5 Hz, 1 H), 4.40 (s, 2 H), 4.25 (s, 1 H), 3.64 (d, *J* = 6.5 Hz, 2 H), 3.40 (s, 3 H), 3.27 (s, 2 H), 2.48-2.42 (m, 7 H), 1.14 (t, *J* = 7.6 Hz, 6 H); ^{13}C NMR (100 MHz, $CDCl_3$): δ 143.5, 143.2, 143.0, 136.8, 136.2, 133.0, 129.7, 127.1, 125.6, 112.7, 74.9, 58.2, 48.9, 32.0, 25.9, 21.5, 15.2; HRMS (m/z): $[M+Na]^+$ calcd. for $C_{23}H_{31}NO_3S$, 424.1917; found, 424.1927.

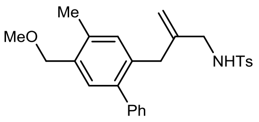

N-(2-((5-(methoxymethyl)-4-methyl-[1,1'-biphenyl]-2-yl)methyl)allyl)-4-methylbenzenesulfonamide **26** (29 mg, 66%). White solid; mp: 93-94 °C; ^{1}H NMR (400 MHz, $CDCl_3$): δ 7.62 (d, *J* = 8.0 Hz, 2 H), 7.38-7.33 (m, 3 H), 7.26-7.24 (m, 2 H), 7.21 (s, 1 H), 7.18 (d, *J* = 7.8 Hz, 2 H), 6.96 (s, 1 H), 4.91 (s, 1 H), 4.58 (s, 1 H), 4.44 (s, 2 H), 4.24 (t, *J* = 6.2 Hz, 1 H), 3.41 (s, 3 H), 3.34 (d, *J* = 6.2 Hz, 2 H), 3.15 (s, 2 H), 2.41 (s, 3 H), 2.33 (s, 3 H); ^{13}C NMR (100 MHz, $CDCl_3$): δ 144.1, 143.3, 141.2, 139.7, 136.9, 135.8, 134.9, 134.3, 132.0, 130.4, 129.5, 129.1, 128.0, 127.0, 126.9, 114.3, 72.7, 58.3, 47.6, 36.9, 21.5, 18.3; HRMS (m/z): $[M+Na]^+$ calcd. for $C_{26}H_{29}NO_3S$, 458.1760; found, 458.1775.

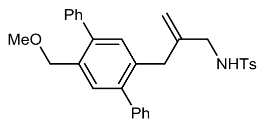

N-(2-((5'-(methoxymethyl)-[1,1':4',1''-terphenyl]-2'-yl)methyl)allyl)-4-methylbenzenesulfonamide **27** (28 mg, 57%). White solid; mp: 135-136 °C; ^{1}H NMR (400 MHz, $CDCl_3$): δ 7.60 (d, *J* = 8.1 Hz, 2 H), 7.45-7.36 (m, 9 H), 7.28 (s, 1 H), 7.26 (d, *J* = 8.1 Hz, 1 H), 7.20 (d, *J* = 7.9 Hz, 2 H), 7.08 (s, 1 H), 4.91 (s, 1 H), 4.61 (s, 1 H), 4.33 (s, 2 H), 4.25 (t, *J* = 6.1 Hz, 1 H), 3.37 (d, *J* = 6.3 Hz, 2 H), 3.34 (s, 3 H), 3.22 (s, 2 H), 2.36 (s, 3 H); ^{13}C NMR (100 MHz, $CDCl_3$): δ 144.0, 143.3, 141.4, 140.9, 140.3, 136.9, 135.1, 133.5, 131.7, 131.2, 129.5, 129.2, 129.1, 128.1, 127.2, 127.1, 127.0, 114.4, 72.2, 58.2, 47.7, 37.0, 21.4; HRMS (m/z): $[M+Na]^+$ calcd. for $C_{31}H_{31}NO_3S$, 520.1917; found, 520.1916.

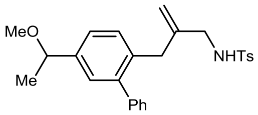

N-(2-((5-(1-methoxyethyl)-[1,1'-biphenyl]-2-yl)methyl)allyl)-4-methylbenzenesulfonamide **28** (33 mg, 77%). Yellow liquid; ^{1}H NMR (400 MHz, $CDCl_3$): δ 7.64 (d, J = 8.2 Hz, 2 H), 7.40-7.34 (m, 3 H), 7.27-7.21 (m, 5 H), 7.16 (d, J = 1.6 Hz, 1 H), 7.13 (d, J = 7.9 Hz, 1 H), 4.94 (s, 1 H), 4.57 (s, 1 H), 4.35-4.27 (m, 2 H), 3.36 (d, J = 6.3 Hz, 2 H), 3.25 (s, 3 H), 3.19 (s, 2 H), 2.41 (s, 3 H), 1.45 (d, J = 6.4 Hz, 3 H); ^{13}C NMR (100 MHz, $CDCl_3$): δ 144.1, 143.3, 142.2, 141.7, 141.4, 136.9, 134.7, 130.3, 129.6, 129.1, 128.1, 128.0, 127.1, 127.0, 125.2, 114.4, 79.2, 56.8, 47.7, 37.0, 23.6, 21.5; HRMS (m/z): $[M+Na]^+$ calcd. for $C_{26}H_{29}NO_3S$, 458.1760; found, 458.1764.

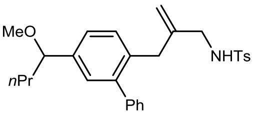

N-(2-((5-(1-methoxybutyl)-[1,1'-biphenyl]-2-yl)methyl)allyl)-4-methylbenzenesulfonamide **29** (33 mg, 72%). Yellow liquid; ^{1}H NMR (400 MHz, $CDCl_3$): δ 7.64 (d, J = 8.0 Hz, 2 H), 7.39-7.33 (m, 3 H), 7.27-7.19 (m, 5 H), 7.13-7.11 (m, 2 H), 4.94 (s, 1 H), 4.57 (s, 1 H), 4.32 (t, J = 6.1 Hz, 1 H), 4.09 (t, J = 6.5 Hz, 1 H), 3.36 (d, J = 6.2 Hz, 2 H), 3.23 (s, 3 H), 3.20 (s, 2 H), 2.42 (s, 3 H), 1.81-1.77 (m, 1 H), 1.64-1.58 (m, 1 H), 1.42-1.26 (m, 2 H), 0.90 (t, J = 7.3 Hz, 3 H); ^{13}C NMR (100 MHz, $CDCl_3$): δ 144.2, 143.3, 142.1, 141.3, 140.8, 136.8, 134.6, 130.2, 129.6, 129.0, 128.5, 128.1, 127.0, 125.6, 114.3, 83.5, 56.6, 47.7, 40.1, 37.0, 21.5, 19.0, 14.0; HRMS (m/z): $[M+Na]^+$ calcd. for $C_{28}H_{33}NO_3S$, 486.2073; found, 486.2075.

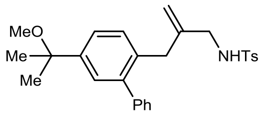

N-(2-((5-(2-methoxypropan-2-yl)-[1,1'-biphenyl]-2-yl)methyl)allyl)-4-methylbenzenesulfonamide **30** (34 mg, 77%). Yellow liquid; ^{1}H NMR (400 MHz, $CDCl_3$): δ 7.64 (d, J = 8.3 Hz, 2 H), 7.40-7.35 (m, 3 H), 7.32 (dd, J = 8.0 Hz, 2.0 Hz, 1 H), 7.27-7.22 (m, 5 H), 7.12 (d, J = 8.0 Hz, 1 H), 4.94 (s, 1 H), 4.58 (s, 1 H), 4.30 (t, J = 6.3 Hz, 1 H), 3.36 (d, J = 6.3 Hz, 2 H), 3.19 (s, 2 H), 3.10 (s, 3 H), 2.41 (s, 3 H), 1.53 (s, 6 H); ^{13}C NMR (100 MHz, $CDCl_3$): δ 144.2, 144.1, 143.3, 142.0, 141.6, 136.9, 134.0, 130.0, 129.6, 129.1, 128.1, 127.6, 127.1, 124.9, 114.3, 76.6, 50.6, 47.7, 37.0, 27.8, 21.5; HRMS (m/z): $[M+Na]^+$ calcd. for $C_{27}H_{31}NO_3S$, 472.1917; found, 472.1935.

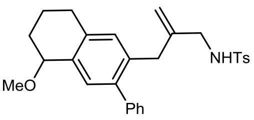

N-(2-((5-methoxy-3-phenyl-5,6,7,8-tetrahydronaphthalen-2-yl)methyl)allyl)-4-methylbenzenesulfonamide **31** (24 mg, 52%). Yellow liquid; ^{1}H NMR (400 MHz, $CDCl_3$): δ 7.62 (d, J = 7.9 Hz, 2 H), 7.37-7.31 (m, 3 H), 7.26-7.21 (m, 5 H), 6.89 (s, 1 H), 4.90 (s, 1 H), 4.58 (s, 1 H), 4.29 (t, J = 4.2 Hz, 1 H), 4.20 (t, J = 6.2 Hz, 1 H), 3.43 (s, 3 H), 3.33 (d, J = 6.2 Hz, 2 H), 3.14 (s, 2 H), 2.84-2.77 (m, 1 H), 2.73-2.65 (m, 1 H), 2.41 (s, 3 H), 2.07-1.86 (m, 3 H), 1.79-1.72 (m, 1 H); ^{13}C NMR (100 MHz, $CDCl_3$): δ 144.0, 143.3, 141.3, 139.8, 136.9, 136.6, 134.8, 131.0, 130.5, 129.5, 129.2, 128.0, 127.0, 126.9, 114.4, 76.4, 56.3, 47.6, 36.9, 28.7, 27.3, 21.5, 18.6; HRMS (m/z): $[M+Na]^+$ calcd. for $C_{28}H_{31}NO_3S$, 484.1917; found, 484.1924.

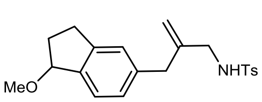

N-(2-((1-methoxy-2,3-dihydro-1H-inden-5-yl)methyl)allyl)-4-methylbenzenesulfonami

de **32** (23 mg, 61%). Yellow solid; mp: 97-98 °C; ^{1}H NMR (400 MHz, $CDCl_3$): δ 7.71 (d, J = 8.1 Hz, 2 H), 7.30-7.26 (m, 3 H), 6.97 (s, 1 H), 6.93 (d, J = 7.7 Hz, 1 H), 4.99 (s, 1 H), 4.85 (s, 1 H), 4.79-4.76 (m, 1 H), 4.65 (t, J = 6.3 Hz, 1 H), 3.45 (d, J = 6.3 Hz, 2 H), 3.40 (s, 3 H), 3.29 (s, 2 H), 3.05-2.98 (m, 1 H), 2.79-2.71 (m, 1 H), 2.42 (s, 3 H), 2.36-2.27 (m, 1 H), 2.11-2.03 (m, 1 H); ^{13}C NMR (100 MHz, $CDCl_3$): δ 144.4, 144.0, 143.4, 140.8, 138.6, 136.8, 129.6, 127.1, 127.0, 125.4, 125.0, 114.1, 84.2, 56.0, 47.2, 40.3, 32.0, 30.0, 21.5; HRMS (m/z): $[M+Na]^+$ calcd. for $C_{21}H_{25}NO_3S$, 394.1447; found, 394.1462.

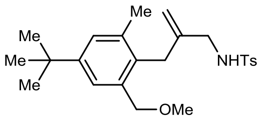

N-(2-(4-(*tert*-butyl)-2-(methoxymethyl)-6-methylbenzyl)allyl)-4-methylbenzenesulfonamide **33** (29 mg, 71%). Pale yellow liquid; ^{1}H NMR (400 MHz, $CDCl_3$): δ 7.75 (d, J = 8.3 Hz, 2 H), 7.29 (d, J = 8.0 Hz, 2 H), 7.15 (d, J = 1.9 Hz, 1 H), 7.11 (d, J = 1.8 Hz, 1 H), 5.23 (t, J = 6.0 Hz, 1 H), 4.89 (s, 1 H), 4.41 (s, 1 H), 4.35 (s, 2 H), 3.54 (d, J = 6.1 Hz, 2 H), 3.33 (s, 3 H), 3.30 (s, 2 H), 2.42 (s, 3 H), 2.16 (s, 3 H), 1.30 (s, 9 H); ^{13}C NMR (100 MHz, $CDCl_3$): δ 149.1, 143.3, 142.3, 137.3, 137.0, 135.5, 132.6, 129.6, 127.4, 127.1, 124.7, 113.4, 73.9, 57.9, 48.5, 34.2, 32.7, 31.3, 21.5 19.9; HRMS (m/z): $[M+Na]^+$ calcd. for $C_{24}H_{33}NO_3S$, 438.2073; found, 438.2079.

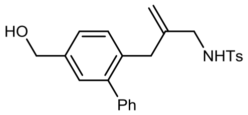

N-(2-((5-(hydroxymethyl)-[1,1'-biphenyl]-2-yl)methyl)allyl)-4-methylbenzenesulfonamide **34** (23 mg, 56%). Pale yellow liquid; ^{1}H NMR (400 MHz, $CDCl_3$): δ 7.62 (d, J = 8.2 Hz, 2 H), 7.40-7.34 (m, 3 H), 7.28-7.24 (m, 3 H), 7.21-7.20 (m, 3 H), 7.14 (d, J = 7.8 Hz, 1 H), 4.92 (s, 1 H), 4.68 (s, 2 H), 4.55 (s, 1 H), 4.31 (t, J = 6.3 Hz, 1 H), 3.33 (d, J = 6.3 Hz, 2 H), 3.19 (s, 2 H), 2.41 (s, 3 H), 1.94 (brs, 1 H); ^{13}C NMR (100 MHz, $CDCl_3$): δ 144.1, 143.3, 142.4, 141.2, 139.1, 136.8, 135.0, 130.4, 129.6, 129.0, 128.8, 128.1, 127.1, 127.0, 126.1, 114.4, 64.9, 47.7, 37.0, 21.5; HRMS (m/z): $[M+Na]^+$ calcd. for $C_{24}H_{25}NO_3S$, 430.1447; found, 430.1467.

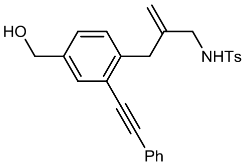

N-(2-(4-(hydroxymethyl)-2-(phenylethynyl)benzyl)allyl)-4-methylbenzenesulfonamide **35** (25 mg, 58%). Yellow solid; mp: 82-83 °C; ^{1}H NMR (400 MHz, $CDCl_3$): δ 7.65 (d, J = 8.2 Hz, 2 H), 7.50-7.48 (m, 3 H), 7.36-7.35 (m, 3 H), 7.26-7.20 (m, 3 H), 7.14 (d, J = 7.9 Hz, 1 H), 5.00 (s, 1 H), 4.84 (s, 1 H), 4.65-4.63 (m, 3 H), 3.56 (s, 2 H), 3.53 (d, J = 6.4 Hz, 2 H), 2.37 (s, 3 H); ^{13}C NMR (100 MHz, $CDCl_3$): δ 143.3, 143.0, 139.5, 139.3, 136.8, 131.5, 130.8, 129.6, 128.4, 127.2, 127.0, 123.2, 123.0, 114.3, 93.4, 87.8, 65.6, 47.5, 38.5, 21.4; HRMS (m/z): $[M+H]^+$ calcd. for $C_{26}H_{25}NO_3S$, 432.1628; found, 432.1635.

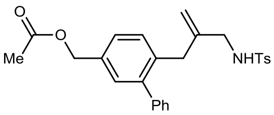

(6-(2-(((4-Methylphenyl)sulfonamido)methyl)allyl)-[1,1'-biphenyl]-3-yl)methyl acetate **36** (25 mg, 55%). Yellow liquid; ^{1}H NMR (400 MHz, $CDCl_3$): δ 7.63 (d, J = 8.3 Hz, 2 H), 7.41-7.33 (m, 3 H), 7.27-7.25 (m, 3 H), 7.23-7.20 (m, 3 H), 7.15 (d, J = 7.8 Hz, 1 H), 5.10 (s, 2 H), 4.94 (s, 1 H), 4.56 (s, 1 H), 4.25 (t, J = 6.3 Hz, 1 H), 3.35 (d, J = 6.3 Hz, 2

H), 3.20 (s, 2 H), 2.42 (s, 3 H), 2.10 (s, 3 H); ^{13}C NMR (100 MHz, $CDCl_3$): δ 170.9, 144.0, 143.4, 142.5, 140.9, 136.8, 135.8, 134.1, 130.5, 130.0, 129.6, 129.0, 128.2, 127.3, 127.1, 114.5, 65.9, 47.7, 37.0, 21.5, 21.0; HRMS (m/z): $[M+Na]^+$ calcd. for $C_{26}H_{27}NO_4S$, 472.1553; found, 472.1554.

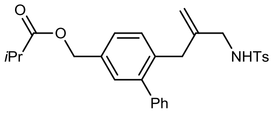

(6-(2-(((4-Methylphenyl)sulfonamido)methyl)allyl)-[1,1'-biphenyl]-3-yl)methyl isobutyrate **37** (22 mg, 46%). Yellow liquid; ^{1}H NMR (400 MHz, $CDCl_3$): δ 7.63 (d, J = 8.2 Hz, 2 H), 7.41-7.34 (m, 3 H), 7.27-7.20 (m, 6 H), 7.15 (d, J = 7.8 Hz, 1 H), 5.11 (s, 2 H), 4.93 (s, 1 H), 4.56 (s, 1 H), 4.22 (t, J = 6.3 Hz, 1 H), 3.35 (d, J = 6.3 Hz, 2 H), 3.20 (s, 2 H), 2.66-2.55 (m, 1 H), 2.42 (s, 3 H), 1.19 (d, J = 7.0 Hz, 6 H); ^{13}C NMR (100 MHz, $CDCl_3$): δ 177.0, 144.0, 143.4, 142.4, 141.0, 136.8, 135.5, 134.5, 130.4, 129.7, 129.6, 129.0, 128.1, 127.2, 127.0, 126.9, 114.5, 65.6, 47.7, 37.0, 34.0, 21.5, 19.0; HRMS (m/z): $[M+Na]^+$ calcd. for $C_{28}H_{31}NO_4S$, 500.1866; found, 500.1873.

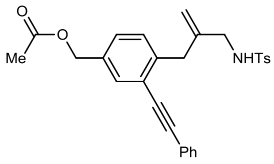

4-(2-(((4-Methylphenyl)sulfonamido)methyl)allyl)-3-(phenylethynyl)benzyl acetate **38** (29 mg, 62%). Yellow solid; mp: 93-94 °C; ^{1}H NMR (400 MHz, $CDCl_3$): δ 7.67 (d, J = 8.2 Hz, 2 H), 7.51-7.49 (m, 3 H), 7.37-7.35 (m, 3 H), 7.26-7.20 (m, 3 H), 7.16 (d, J = 7.9 Hz, 1 H), 5.06 (s, 2 H), 5.03 (s, 1 H), 4.85 (s, 1 H), 4.73 (t, J = 6.4 Hz, 1 H), 3.56 (s, 2 H), 3.54 (d, J = 6.5 Hz, 2 H), 2.38 (s, 3 H), 2.11 (s, 3 H); ^{13}C NMR (100 MHz, $CDCl_3$): δ 170.8, 143.3, 142.9, 140.2, 136.8, 134.3, 132.1, 131.5, 129.6, 129.4, 128.5, 128.4, 128.3, 127.0, 123.4, 122.9, 114.4, 93.6, 87.5, 65.5, 47.5, 38.4, 21.4, 20.9; HRMS (m/z): $[M+H]^+$ calcd. for $C_{28}H_{27}NO_4S$, 474.1734; found, 474.1740.

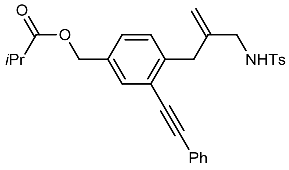

4-(2-(((4-Methylphenyl)sulfonamido)methyl)allyl)-3-(phenylethynyl)benzyl isobutyrate **39** (26 mg, 51%). Yellow solid; mp: 92-93 °C; ^{1}H NMR (400 MHz, $CDCl_3$): δ 7.67 (d, J = 8.2 Hz, 2 H), 7.51-7.49 (m, 3 H), 7.37-7.35 (m, 3 H), 7.26-7.20 (m, 3 H), 7.15 (d, J = 7.9 Hz, 1 H), 5.07 (s, 2 H), 5.03 (s, 1 H), 4.85 (s, 1 H), 4.71 (t, J = 6.3 Hz, 1 H), 3.56 (s, 2 H), 3.54 (d, J = 6.7 Hz, 2 H), 2.65-2.58 (m, 1 H), 2.38 (s, 3 H), 1.20 (d, J = 7.0 Hz, 6 H); ^{13}C NMR (100 MHz, $CDCl_3$): δ 176.9, 143.3, 142.9, 140.1, 136.8, 134.6, 131.9, 131.5, 129.6, 128.5, 128.4, 128.2, 127.0, 123.3, 122.9, 114.4, 93.5, 87.6, 65.3, 47.5, 38.4, 33.9, 21.4, 18.9; HRMS (m/z): $[M+H]^+$ calcd. for $C_{30}H_{31}NO_4S$, 502.2047; found, 502.2065.

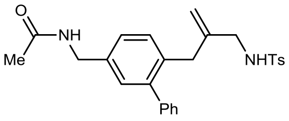

N-((6-(2-(((4-methylphenyl)sulfonamido)methyl)allyl)-[1,1'-biphenyl]-3-yl)methyl)acetamide **40** (21 mg, 47%). Yellow liquid; ^{1}H NMR (400 MHz, $CDCl_3$): δ 7.62 (d, J = 8.1 Hz, 2 H), 7.36-7.32 (m, 3 H), 7.25 (d, J = 8.3 Hz, 2 H), 7.20-7.10 (m, 5 H), 6.08 (brs, 1 H), 4.92 (s, 1 H), 4.54 (s, 1 H), 4.51 (t, J = 6.3 Hz, 1 H), 4.41 (d, J = 5.6 Hz, 2 H), 3.32 (d, J = 6.2 Hz, 2 H), 3.18 (s, 2 H), 2.41 (s, 3 H), 1.99 (s, 3 H); ^{13}C NMR (100 MHz, $CDCl_3$): δ 170.1, 144.0, 143.3, 142.5, 141.0, 136.8, 136.4, 134.8, 130.5, 129.6, 129.4,

128.9, 128.1, 127.2, 127.0, 126.8, 114.4, 47.6, 43.2, 36.9, 23.2, 21.5; HRMS (m/z): $[M+Na]^+$ calcd. for $C_{26}H_{28}N_2O_3S$, 471.1713; found, 471.1714.

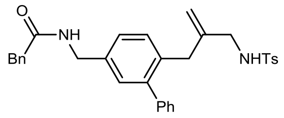

N-((6-(2-(((4-methylphenyl)sulfonamido)methyl)allyl)-[1,1'-biphenyl]-3-yl)methyl)-2-phenylacetamide **41** (24 mg, 46%). Yellow liquid; ^{1}H NMR (400 MHz, $CDCl_3$): δ 7.62 (d, J = 8.1 Hz, 2 H), 7.37-7.24 (m, 10 H), 7.16-7.14 (m, 2 H), 7.10-7.06 (m, 2 H), 7.00 (s, 1 H), 5.81 (brs, 1 H), 4.90 (s, 1 H), 4.52 (s, 1 H), 4.41 (d, J = 5.8 Hz, 2 H), 4.28 (t, J = 6.2 Hz, 1 H), 3.61 (s, 2 H), 3.32 (d, J = 6.2 Hz, 2 H), 3.16 (s, 2 H), 2.41 (s, 3 H); ^{13}C NMR (100 MHz, $CDCl_3$): δ 170.9, 144.0, 143.3, 142.5, 141.0, 136.9, 136.4, 134.8, 130.5, 129.6, 129.4, 129.1, 129.0, 128.9, 128.1, 127.3, 127.2, 127.0, 126.4, 114.4, 47.6, 43.8, 43.0, 36.9, 21.5; HRMS (m/z): $[M+Na]^+$ calcd. for $C_{32}H_{32}N_2O_3S$, 547.2026; found, 547.2044.

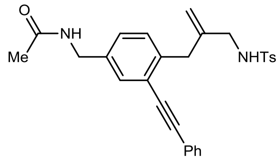

N-(4-(2-(((4-methylphenyl)sulfonamido)methyl)allyl)-3-(phenylethynyl)benzyl)acetamide **42** (24 mg, 50%). Yellow solid; mp: 87-88 °C; ^{1}H NMR (400 MHz, $CDCl_3$): δ 7.64 (d, J = 8.1 Hz, 2 H), 7.49-7.46 (m, 2 H), 7.40 (s, 1 H), 7.36-7.34 (m, 3 H), 7.20 (d, J = 8.0 Hz, 2 H), 7.16-7.10 (m, 2 H), 6.18 (s, 1 H), 5.00 (s, 1 H), 4.88 (t, J = 6.4 Hz, 1 H), 4.84 (s, 1 H), 4.36 (d, J = 5.7 Hz, 2 H), 3.54 (s, 2 H), 3.51 (d, J = 6.3 Hz, 2 H), 2.36 (s, 3 H), 2.00 (s, 3 H); ^{13}C NMR (100 MHz, $CDCl_3$): δ 170.2, 143.3, 142.9, 139.4, 136.7, 131.5, 131.4, 129.7, 129.6, 128.4, 128.0, 127.0, 123.3, 122.9, 114.2, 93.5, 87.7, 47.4, 42.9, 38.4, 23.1, 21.4; HRMS (m/z): $[M+H]^+$ calcd. for $C_{28}H_{28}N_2O_3S$, 473.1893; found, 473.1897.

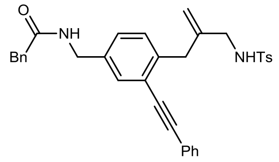

N-(4-(2-(((4-methylphenyl)sulfonamido)methyl)allyl)-3-(phenylethynyl)benzyl)-2-phenylacetamide **43** (24 mg, 43%). Yellow solid; mp: 91-92 °C; ^{1}H NMR (400 MHz, $CDCl_3$): δ 7.66- 7.62 (m, 3 H), 7.54-7.43 (m, 4 H), 7.36-7.26 (m, 7 H), 7.20 (d, J = 8.1 Hz, 2 H), 7.07 (s, 1 H), 5.99 (s, 1 H), 5.00 (s, 1 H), 4.81-4.77 (m, 2 H), 4.35 (d, J = 5.9 Hz, 2 H), 3.61 (s, 2 H), 3.52-3.50 (m, 4 H), 2.36 (s, 3 H); ^{13}C NMR (100 MHz, $CDCl_3$): δ 171.0, 143.3, 143.0, 139.4, 136.6, 134.8, 132.1, 132.0, 131.4, 131.3, 129.7, 129.6, 129.4, 129.0, 128.4, 127.7, 127.3, 127.0, 123.3, 122.9, 114.2, 93.4, 87.7, 47.5, 43.7, 42.8, 38.3, 21.4; HRMS (m/z): $[M+H]^+$ calcd. for $C_{34}H_{32}N_2O_3S$, 549.2206; found, 549.2217.

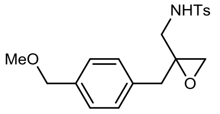

N-((2-(4-(methoxymethyl)benzyl)oxiran-2-yl)methyl)-4-methylbenzenesulfonamide **44** (21 mg, 58%). Yellow liquid; ^{1}H NMR (400 MHz, $CDCl_3$): δ 7.68 (d, J = 7.8 Hz, 2 H), 7.29 (d, J = 7.9 Hz, 2 H), 7.25 (d, J = 8.1 Hz, 2 H), 7.13 (d, J = 7.6 Hz, 2 H), 4.71 (t, J = 6.2 Hz, 1 H), 4.42 (s, 2 H), 3.39 (s, 3 H), 3.12-3.05 (m, 2 H), 3.00 (d, J = 14.4 Hz, 1 H), 2.82 (d, J = 14.4 Hz, 1 H), 2.77 (d, J = 4.3 Hz, 1 H), 2.59 (d, J = 4.3 Hz, 1 H), 2.42 (s, 3 H); ^{13}C NMR (100 MHz, $CDCl_3$): δ 143.6, 136.9, 136.6, 134.8, 129.7, 129.6, 127.9, 127.0, 74.4, 58.3, 58.2, 50.0, 45.2, 38.5, 21.5; HRMS (m/z): $[M+Na]^+$ calcd. for $C_{19}H_{23}NO_4S$, 384.1240; found, 384.1240.

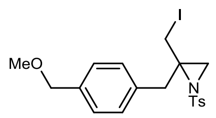

2-(Iodomethyl)-2-(4-(methoxymethyl)benzyl)-1-tosylaziridine **45** (24 mg, 50%). Yellow solid; mp: 84-85 °C; ^{1}H NMR (400 MHz, $CDCl_3$): δ 7.84 (d, J = 8.0 Hz, 2 H), 7.34-7.26 (m, 6 H), 4.43 (s, 2 H), 3.66 (d, J = 10.2 Hz, 1 H), 3.45 (d, J = 14.4 Hz, 1 H), 3.40 (s, 3 H), 3.15-3.09 (m, 2 H), 2.76 (s, 1 H), 2.68 (s, 1 H), 2.45 (s, 3 H); ^{13}C NMR (100 MHz, $CDCl_3$): δ 144.4, 137.2, 136.9, 135.5, 129.6, 129.4, 128.0, 127.6, 74.4, 58.2, 53.2, 41.6, 38.7, 21.6, 7.5; HRMS (m/z): $[M+Na]^+$ calcd. for $C_{19}H_{22}INO_3S$, 494.0257; found, 494.0253.

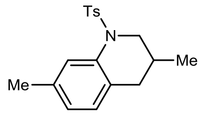

3,7-Dimethyl-1-tosyl-1,2,3,4-tetrahydroquinoline **46** (25 mg, overall yield 79%). Yellow solid; mp: 108-109 °C; ^{1}H NMR (400 MHz, $CDCl_3$): δ 7.62 (s, 1 H), 7.49 (d, J = 8.0 Hz, 2 H), 7.19 (d, J = 7.9 Hz, 2 H), 6.87 (s, 2 H), 4.10 (dd, J = 13.2 Hz, 3.7 Hz, 1 H), 3.03-2.97 (m, 1 H), 2.51 (dd, J = 16.3 Hz, 5.0 Hz, 1 H), 2.37 (s, 3 H), 2.33 (s, 3 H), 2.01 (dd, J = 16.1 Hz, 10.9 Hz, 1 H), 1.70-1.63 (m, 1 H), 0.91 (d, J = 6.5 Hz, 3 H); ^{13}C NMR (100 MHz, $CDCl_3$): δ 143.4, 136.7, 136.2, 136.1, 129.5, 128.8, 127.0, 125.6, 124.5, 52.9, 35.1, 27.2, 21.5, 21.3, 19.0; HRMS (m/z): $[M+Na]^+$ calcd. for $C_{18}H_{21}NO_2S$, 338.1185; found, 338.1192.

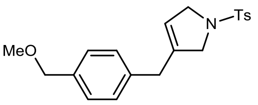

3-(4-(Methoxymethyl)benzyl)-1-tosyl-2,5-dihydro-1H-pyrrole **47** (22 mg, overall yield 61%). Pale yellow liquid; ^{1}H NMR (400 MHz, $CDCl_3$): δ 7.66 (d, J = 7.9 Hz, 2 H), 7.29 (d, J = 7.9 Hz, 2 H), 7.23 (d, J = 7.7 Hz, 2 H), 7.04 (d, J = 7.7 Hz, 2 H), 5.24 (s, 1 H), 4.42 (s, 2 H), 4.08 (s, 2 H), 3.96 (s, 2 H), 3.38 (s, 3 H), 3.30 (s, 2 H), 2.43 (s, 3 H); ^{13}C NMR (100 MHz, $CDCl_3$): δ 143.3, 138.7, 136.9, 136.5, 134.1, 129.7, 128.6, 128.0, 127.3, 119.9, 74.3, 58.1, 56.2, 55.0, 35.0, 21.5; HRMS (m/z): $[M+Na]^+$ calcd. for $C_{20}H_{23}NO_3S$, 380.1291; found, 380.1293.

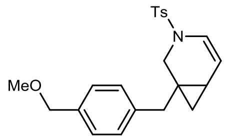

1-(4-(Methoxymethyl)benzyl)-3-tosyl-3-azabicyclo[4.1.0]hept-4-ene **48** (21 mg, overall yield 54%). Yellow solid; mp: 81-82 °C; ^{1}H NMR (400 MHz, $CDCl_3$): δ 7.59 (d, J = 7.9 Hz, 2 H), 7.29 (d, J = 7.9 Hz, 2 H), 7.25 (d, J = 8.3 Hz, 2 H), 7.10 (d, J = 7.7 Hz, 2 H), 6.29 (d, J = 7.8 Hz, 1 H), 5.44-5.40 (m, 1 H), 4.43 (s, 2 H), 3.78 (d, J = 11.5 Hz, 1 H), 3.39 (s, 3 H), 2.81-2.76 (m, 2 H), 2.58 (d, J = 14.5 Hz, 1 H), 2.42 (s, 3 H), 1.09-1.05 (m, 1 H), 0.79 (dd, J = 8.1 Hz, 4.6 Hz, 1 H), 0.54 (t, J = 4.2 Hz, 1 H); ^{13}C NMR (100 MHz, $CDCl_3$): δ 143.6, 137.3, 136.4, 134.9, 129.7, 129.0, 127.8, 126.9, 120.8, 112.5, 74.4, 58.1, 44.5, 41.4, 30.1, 21.5, 18.7, 14.2; HRMS (m/z): $[M+Na]^+$ calcd. for $C_{22}H_{25}NO_3S$, 406.1447; found, 406.1450.

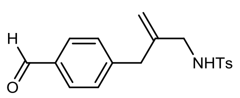

N-(2-(4-formylbenzyl)allyl)-4-methylbenzenesulfonamide **49** (26 mg, 78%). Yellow solid; m.p. 87-88 °C; ^{1}H NMR (400 MHz, $CDCl_3$): δ 9.97 (s, 1 H), 7.77 (d, J = 7.6 Hz, 2 H), 7.71 (d, J = 7.8 Hz, 2 H), 7.30-7.26 (m, 4 H), 5.06 (s, 1 H), 4.89 (t, J = 6.4 Hz, 1

H), 4.86 (s, 1 H), 3.46 (d, *J* = 6.3 Hz, 2 H), 3.42 (s, 2 H), 2.43 (s, 3 H); ^{13}C NMR (100 MHz, $CDCl_3$): δ 191.9, 145.7, 143.6, 142.9, 136.7, 134.8, 129.9, 129.7, 129.6, 127.0, 115.3, 47.3, 40.2, 21.5; HRMS (m/z): $[M+Na]^+$ calcd. for $C_{18}H_{19}NO_3S$, 352.0978; found, 352.0981.

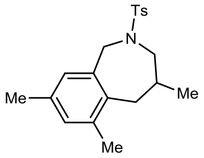

4,6,8-Trimethyl-2-tosyl-2,3,4,5-tetrahydro-1*H*-benzo[*c*]azepine **50** (14 mg, overall yield 42%). Yellow solid; mp: 118-119 °C; ^{1}H NMR (400 MHz, $CDCl_3$): δ 7.58 (d, *J* = 8.2 Hz, 2 H), 7.23 (d, *J* = 8.1 Hz, 2 H), 6.89 (s, 1 H), 6.88 (s, 1 H), 4.41 (d, *J* = 14.5 Hz, 1 H), 4.20 (d, *J* = 14.5 Hz, 1 H), 3.58 (d, *J* = 11.9 Hz, 1 H), 2.99-2.94 (m, 1 H), 2.80 (dd, *J* = 14.7 Hz, 1.8 Hz, 1 H), 2.59 (dd, *J* = 14.7 Hz, 9.0 Hz, 1 H), 2.40 (s, 3 H), 2.25 (s, 3 H), 2.22 (s, 3 H), 1.84-1.82 (m, 1 H), 0.89 (d, *J* = 6.8 Hz, 3 H); ^{13}C NMR (100 MHz, $CDCl_3$): δ 142.9, 136.7, 136.5, 135.8, 135.4, 134.9, 130.7, 129.4, 127.8, 127.1, 57.0, 53.1, 35.2, 31.7, 21.4, 20.7, 20.2, 19.0; HRMS (m/z): $[M+H]^+$ calcd. for $C_{20}H_{25}NO_2S$, 344.1679; found, 344.1688.

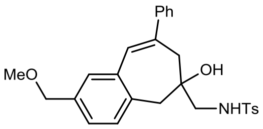

N-((6-hydroxy-2-(methoxymethyl)-8-phenyl-6,7-dihydro-5H-benzo[7]annulen-6-yl)methyl)-4-methylbenzenesulfonamide **51** (29 mg, 63%). Yellow solid; mp: 118-119 °C; ^{1}H NMR (400 MHz, $CDCl_3$): δ 7.71 (d, *J* = 8.3 Hz, 2 H), 7.57-7.54 (m, 2 H), 7.37-7.28 (m, 5 H), 7.21 (s, 1 H), 7.19-7.14 (m, 2 H), 7.01 (s, 1 H), 5.10 (t, *J* = 5.9 Hz, 1 H), 4.44 (s, 2 H), 3.40 (s, 3 H), 3.04 (dd, *J* = 12.5 Hz, 7.5 Hz, 1 H), 2.94-2.87 (m, 2 H), 2.64 (d, *J* = 13.3 Hz, 1 H), 2.50 (d, *J* = 14.3 Hz, 1 H), 2.42 (s, 3 H), 2.34 (d, *J* = 14.4 Hz, 1 H); ^{13}C NMR (100 MHz, $CDCl_3$): δ 143.5, 141.9, 140.2, 138.5, 136.8, 136.5, 135.6, 130.2, 129.8, 128.7, 128.6, 128.2, 127.6, 127.1, 126.6, 126.0, 84.6, 74.4, 58.2, 50.2, 43.2, 40.5, 21.5; HRMS (m/z): $[M+Na]^+$ calcd. for $C_{27}H_{29}NO_4S$, 486.1710; found, 486.1703.

Supplementary Figures

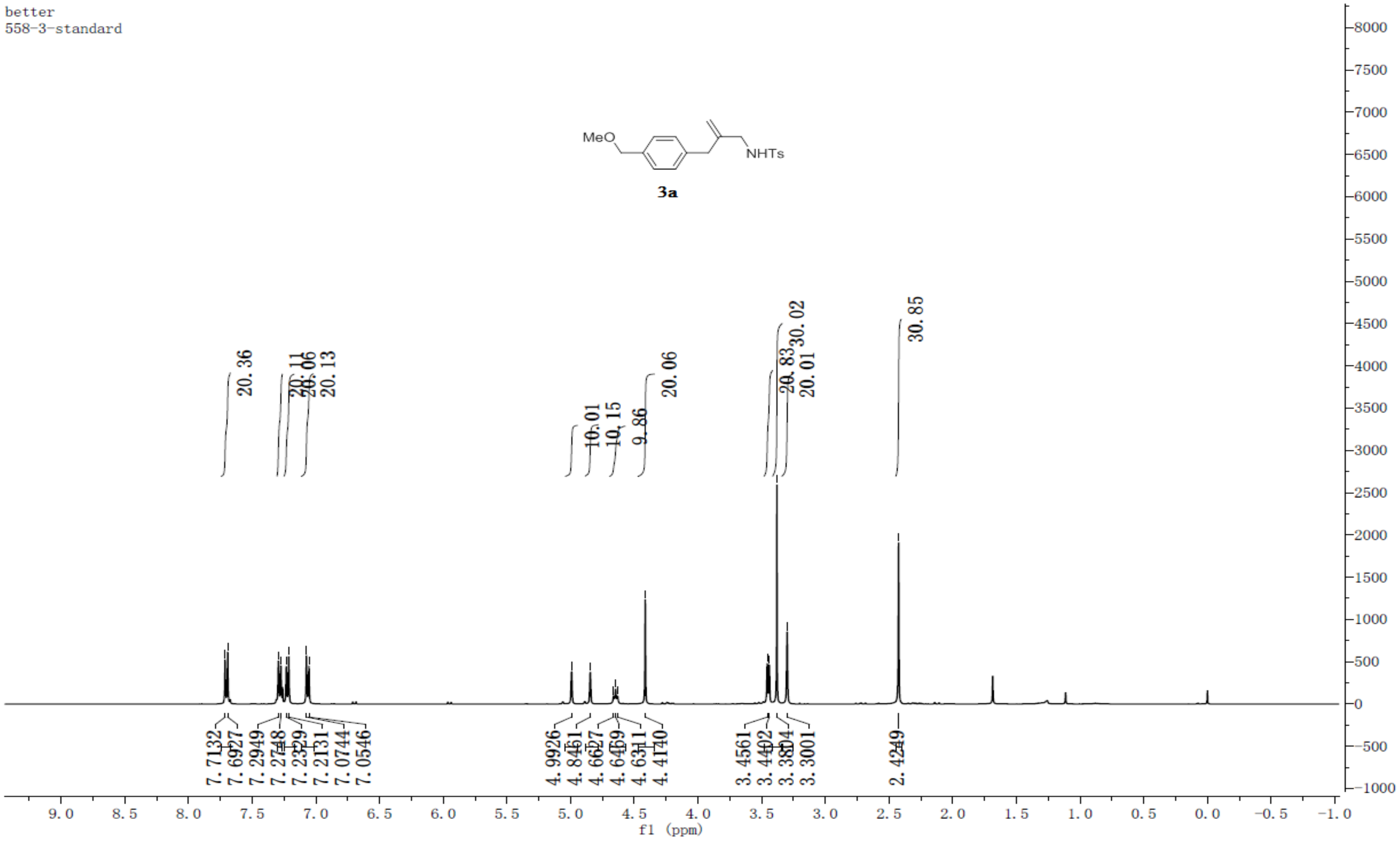

Supplementary Figure 1. ^{1}H NMR Spectrum of substrate 3

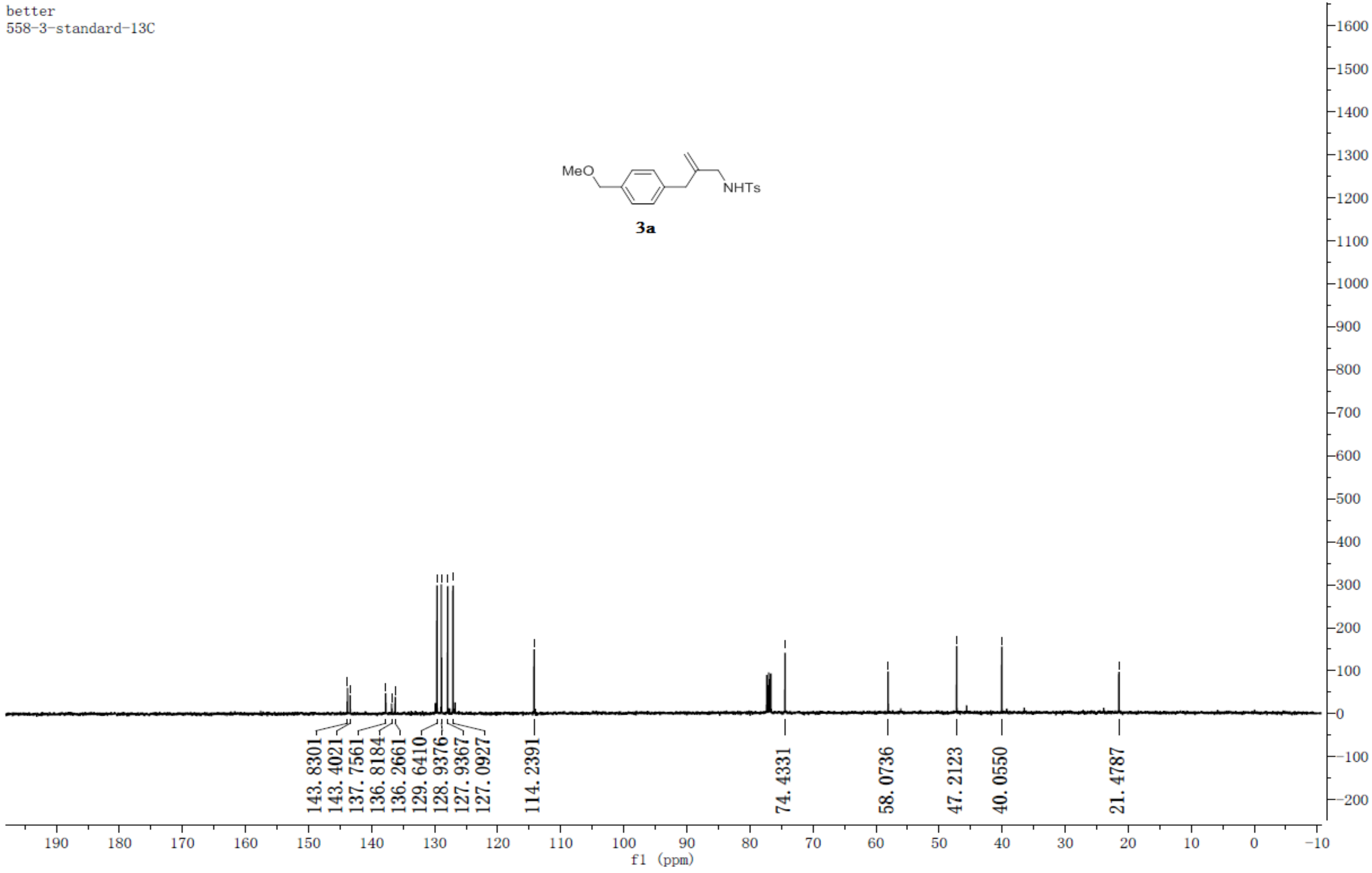

Supplementary Figure 2. ^{13}C NMR Spectrum of substrate 3

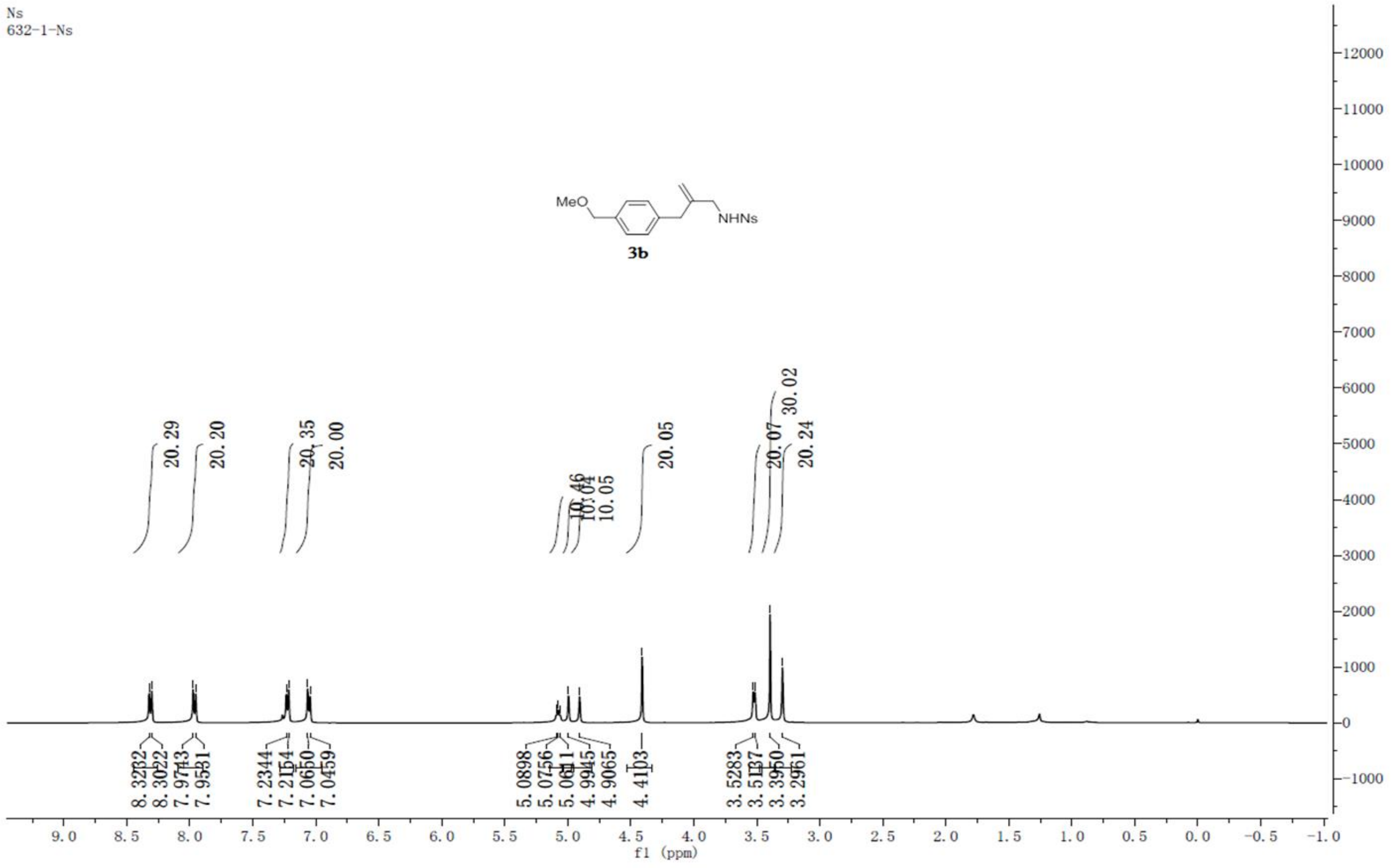

Supplementary Figure 3. ^{1}H NMR Spectrum of substrate 3b

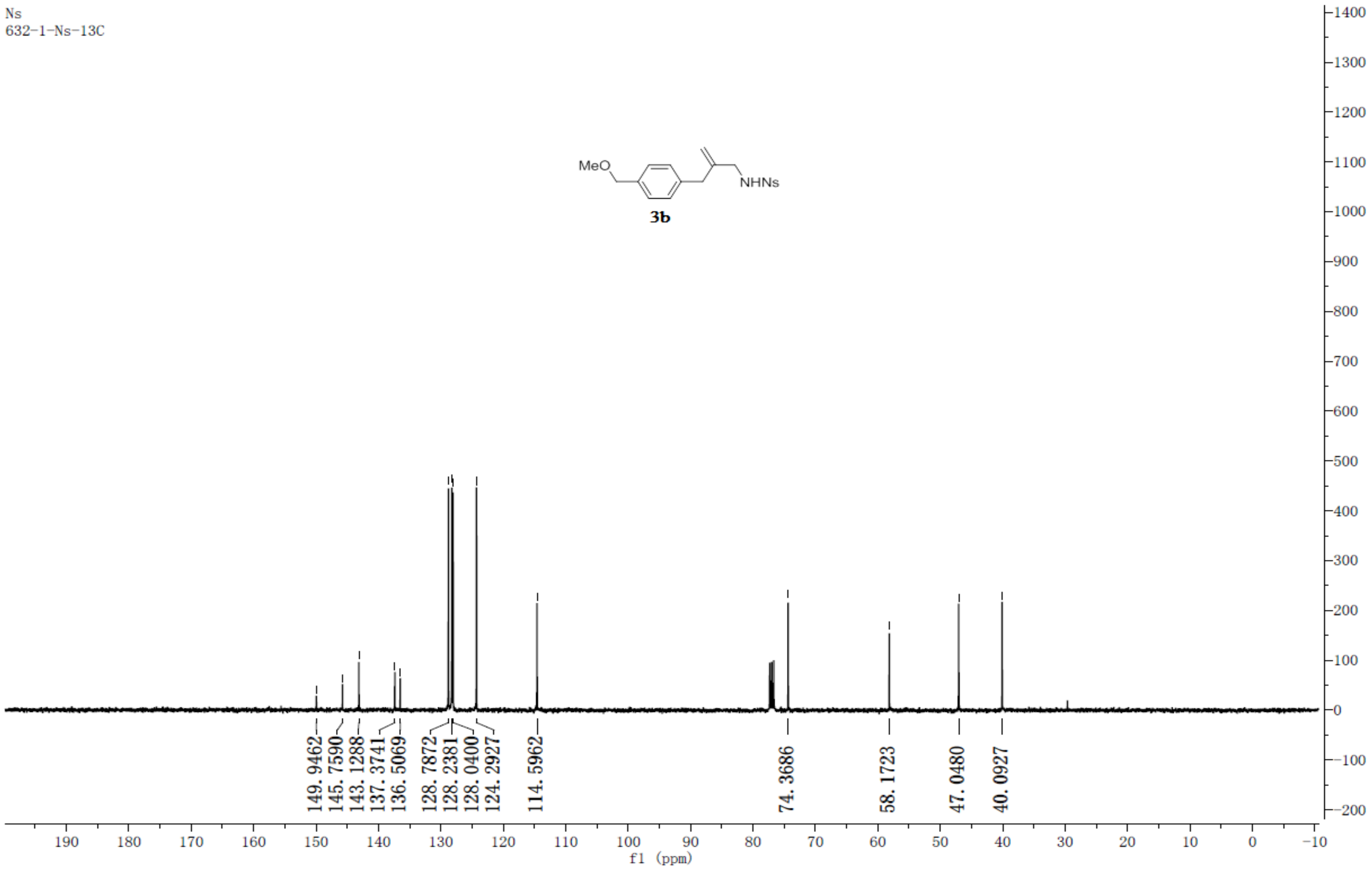

Supplementary Figure 4. ^{13}C NMR Spectrum of substrate 3b

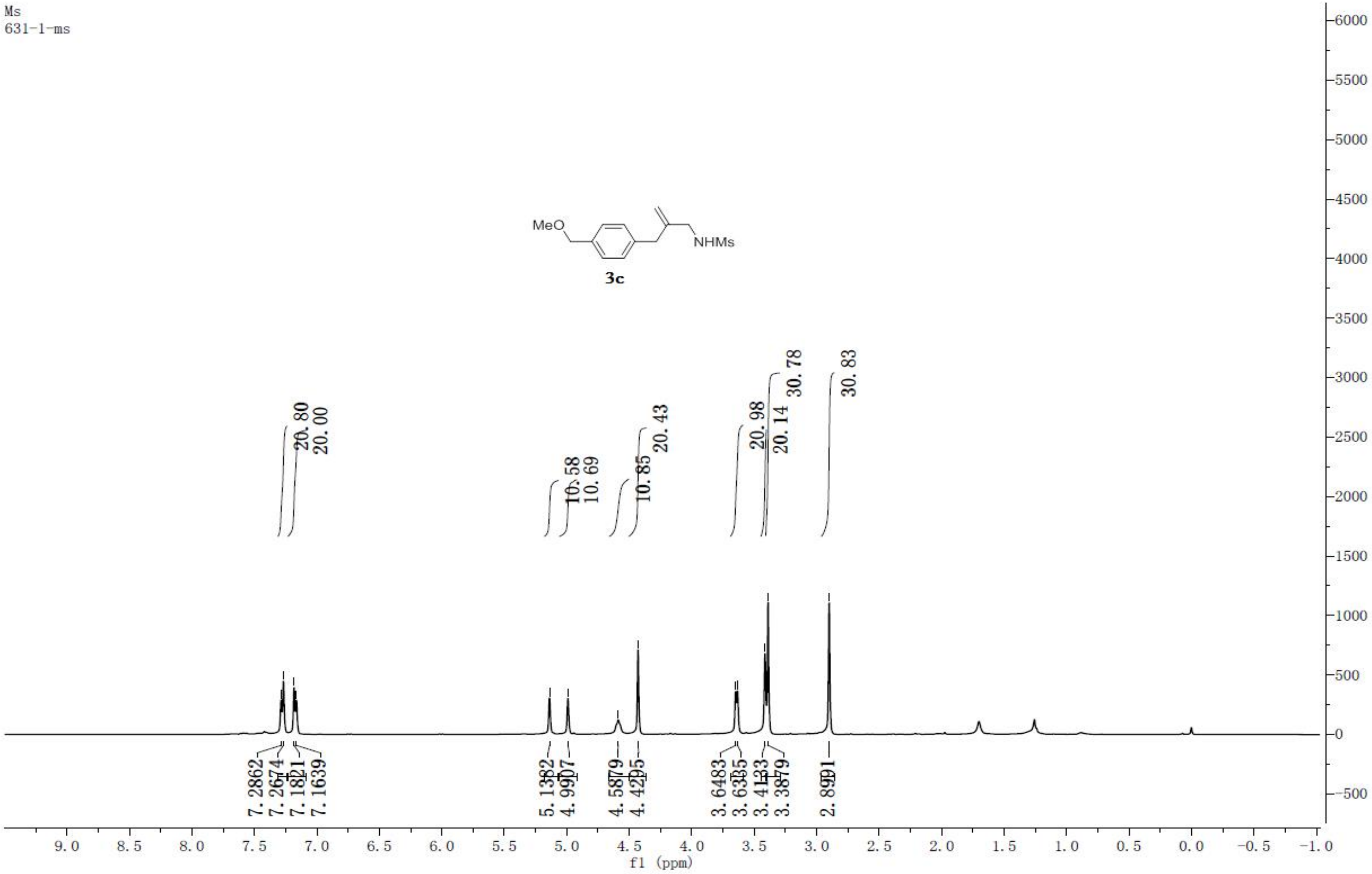

Supplementary Figure 5. 1H NMR Spectrum of substrate 3c

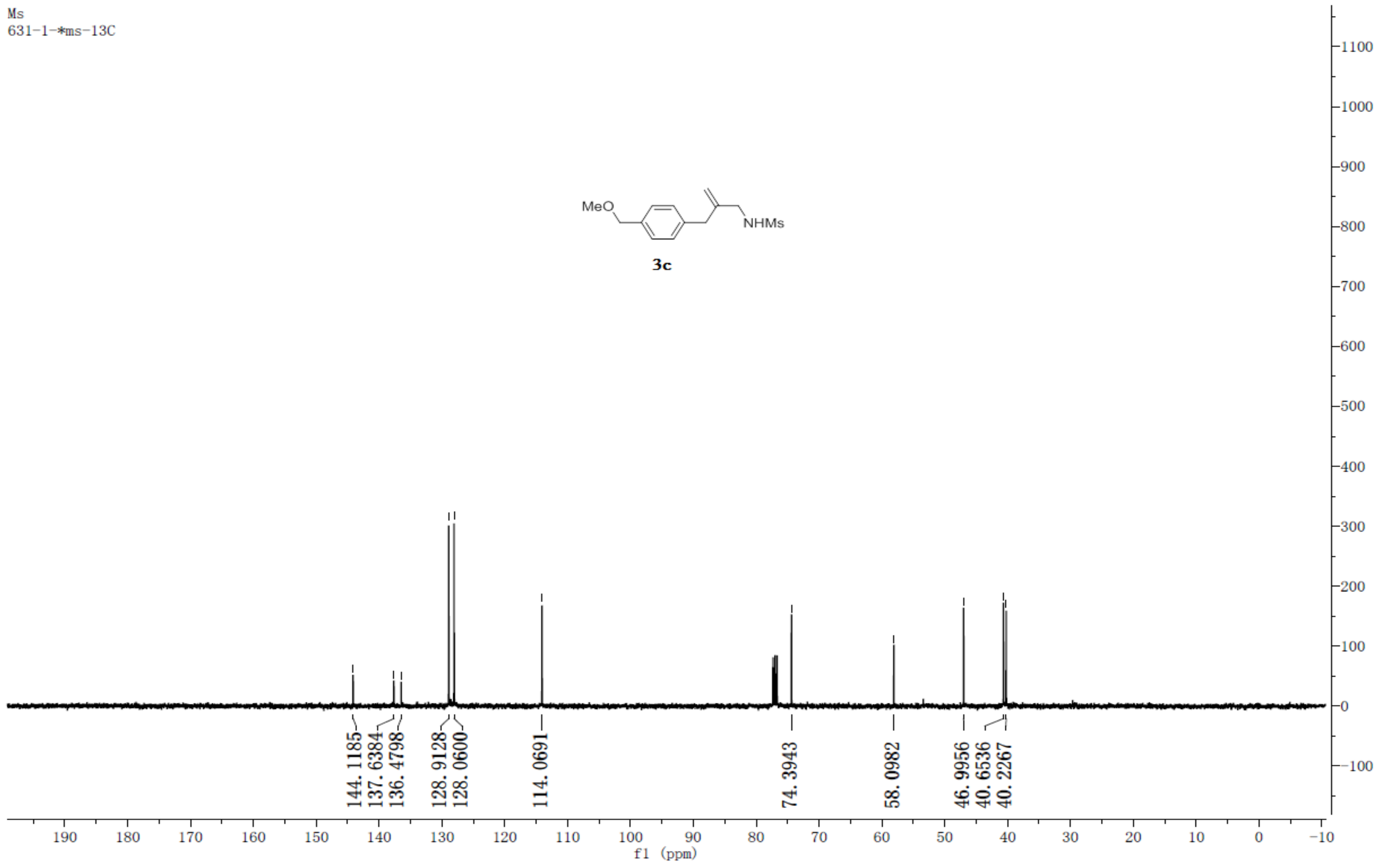

Supplementary Figure 6. ^{13}C NMR Spectrum of substrate 3c

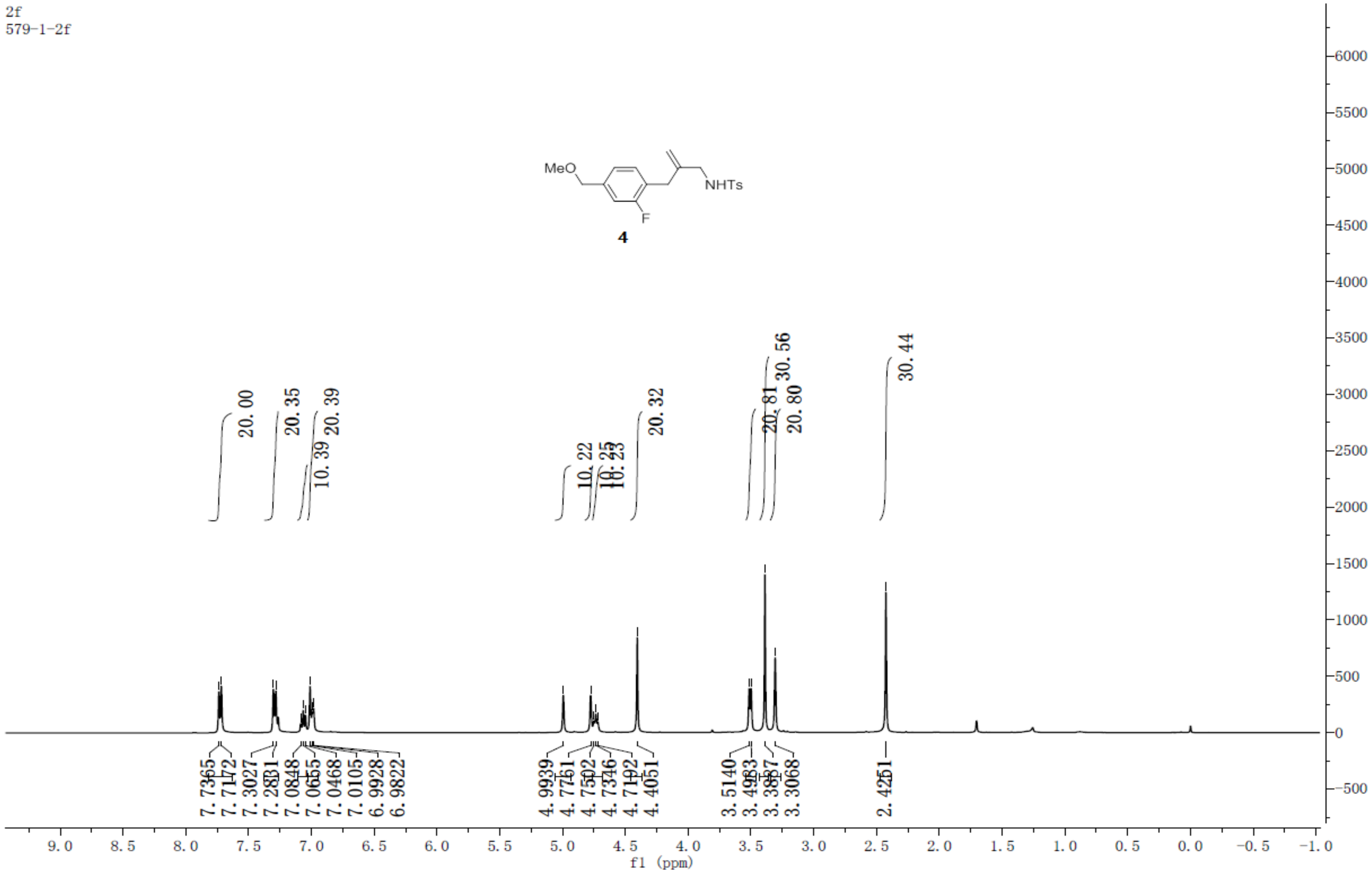

Supplementary Figure 7. ^{1}H NMR Spectrum of substrate 4

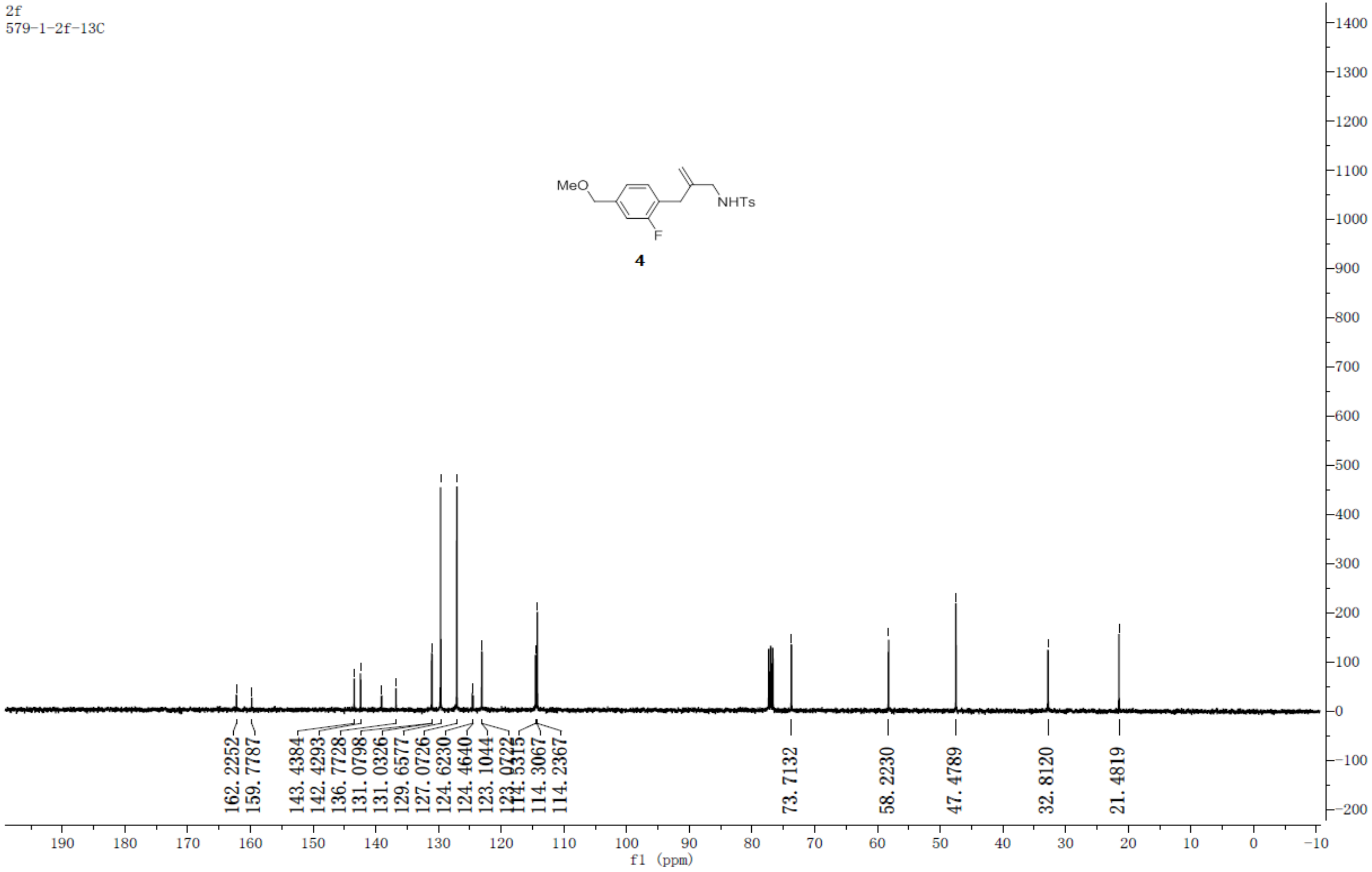

Supplementary Figure 8. ^{13}C NMR Spectrum of substrate 4

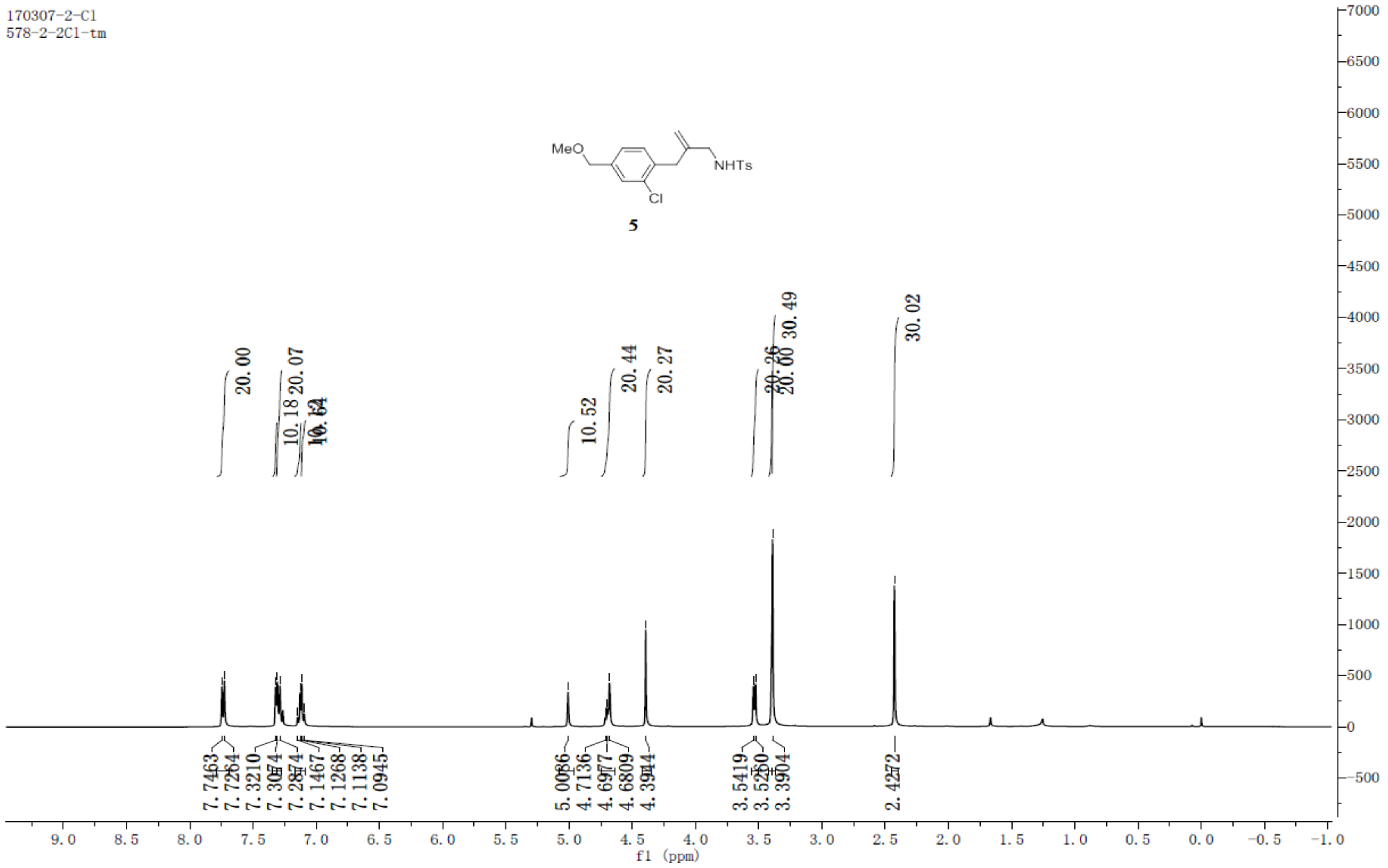

Supplementary Figure 9. ¹H NMR Spectrum of substrate 5

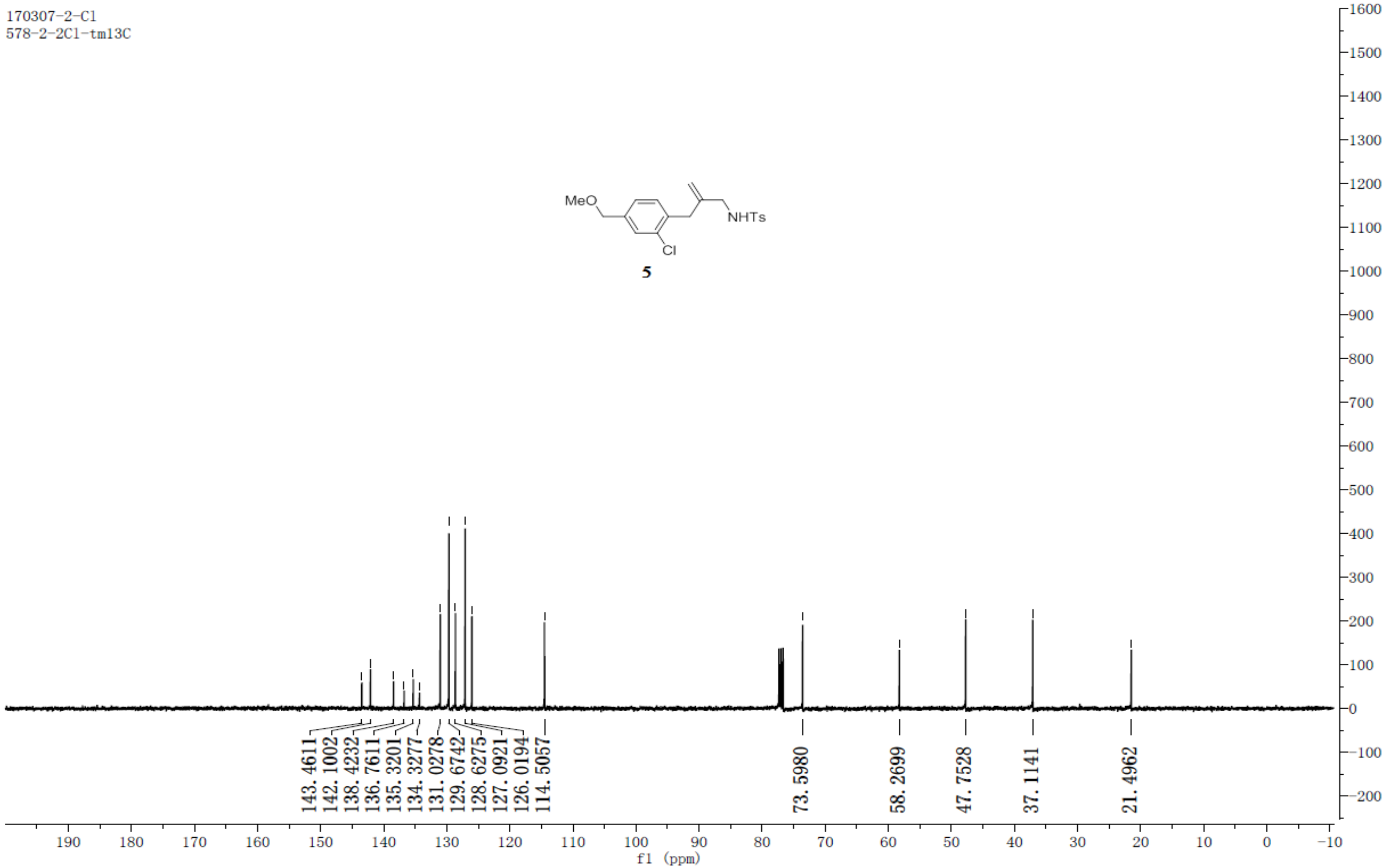

Supplementary Figure 10. ^{13}C NMR Spectrum of substrate 5

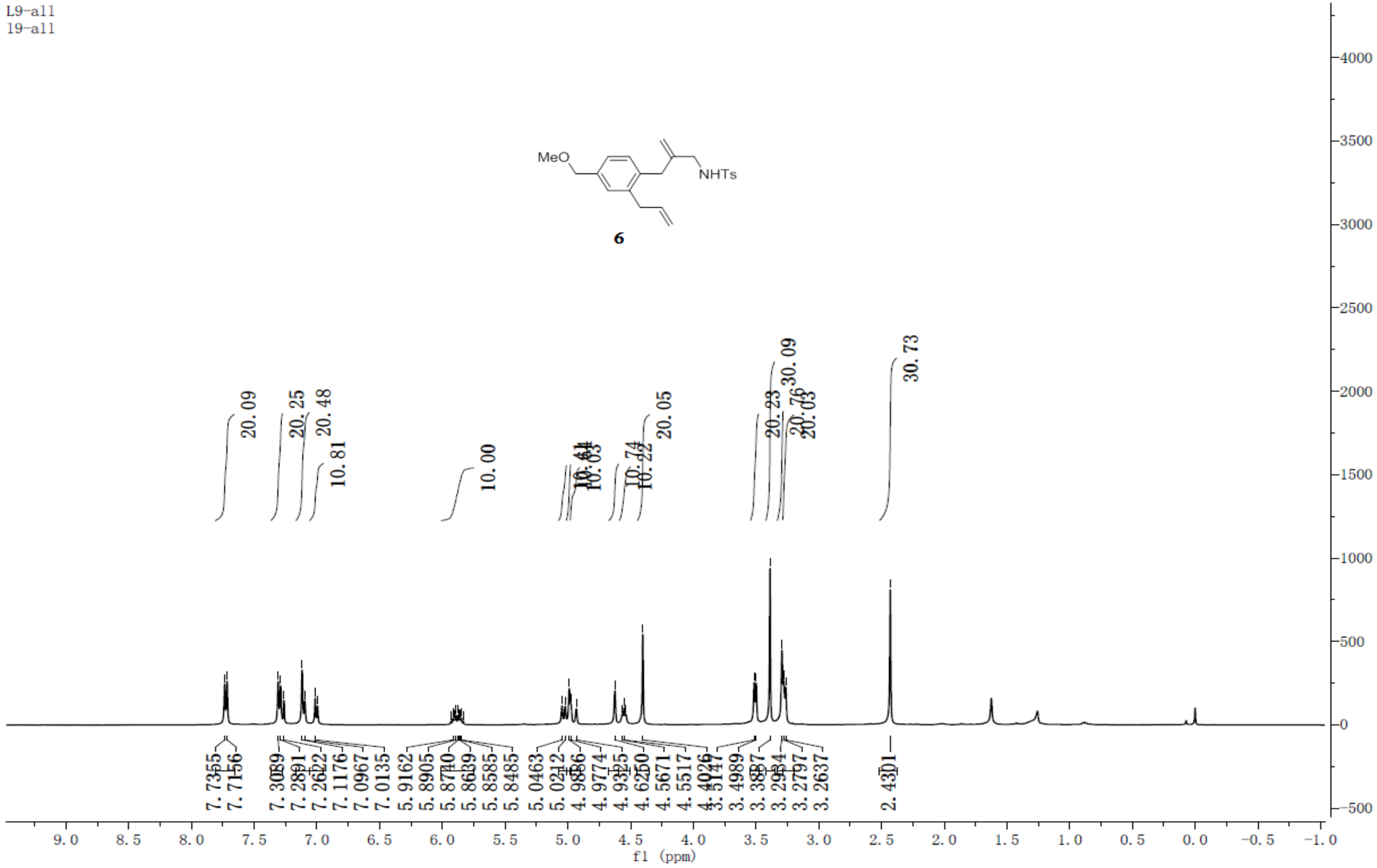

Supplementary Figure 11. ^{1}H NMR Spectrum of substrate 6

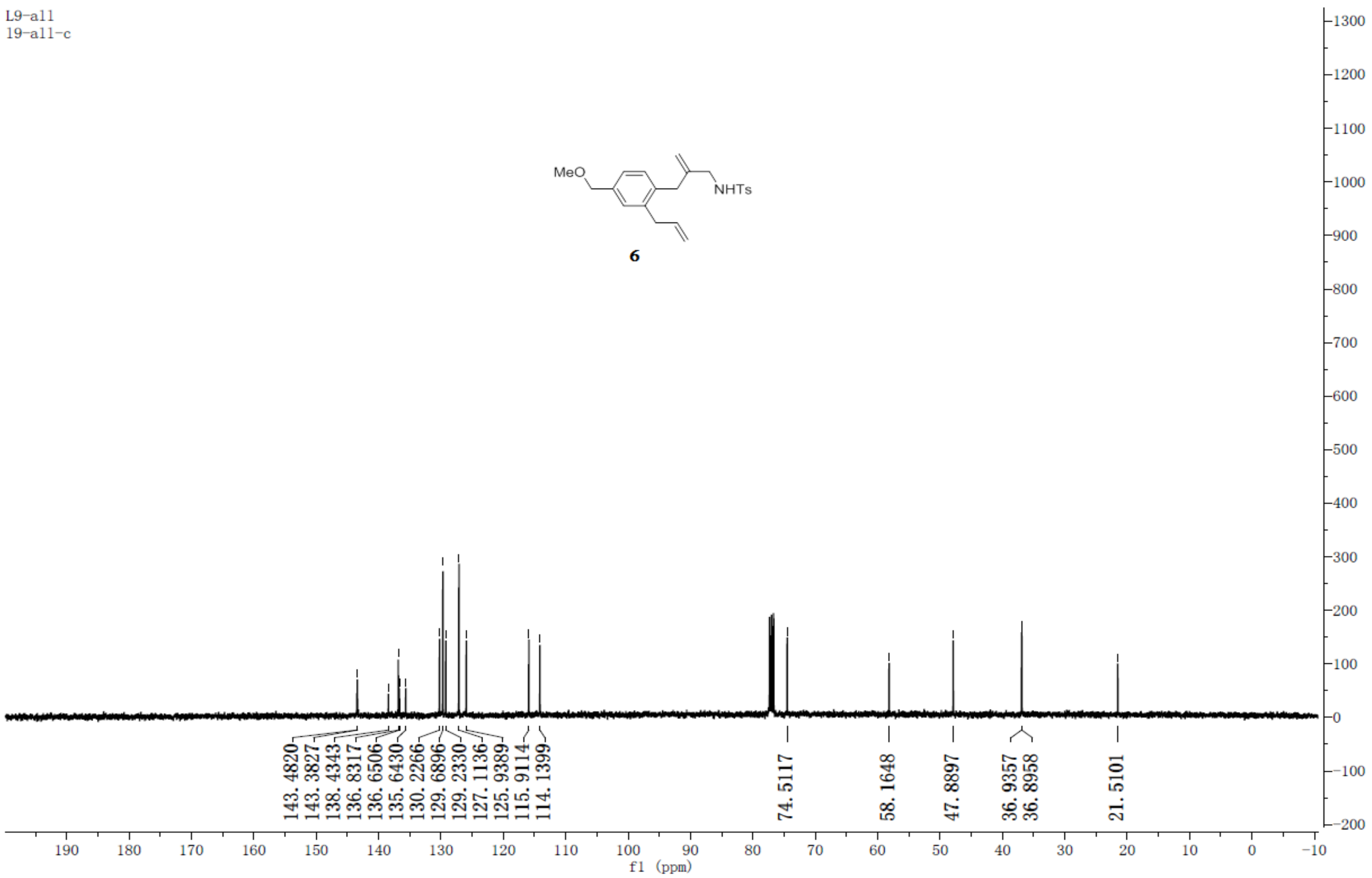

Supplementary Figure 12. ^{13}C NMR Spectrum of substrate 6

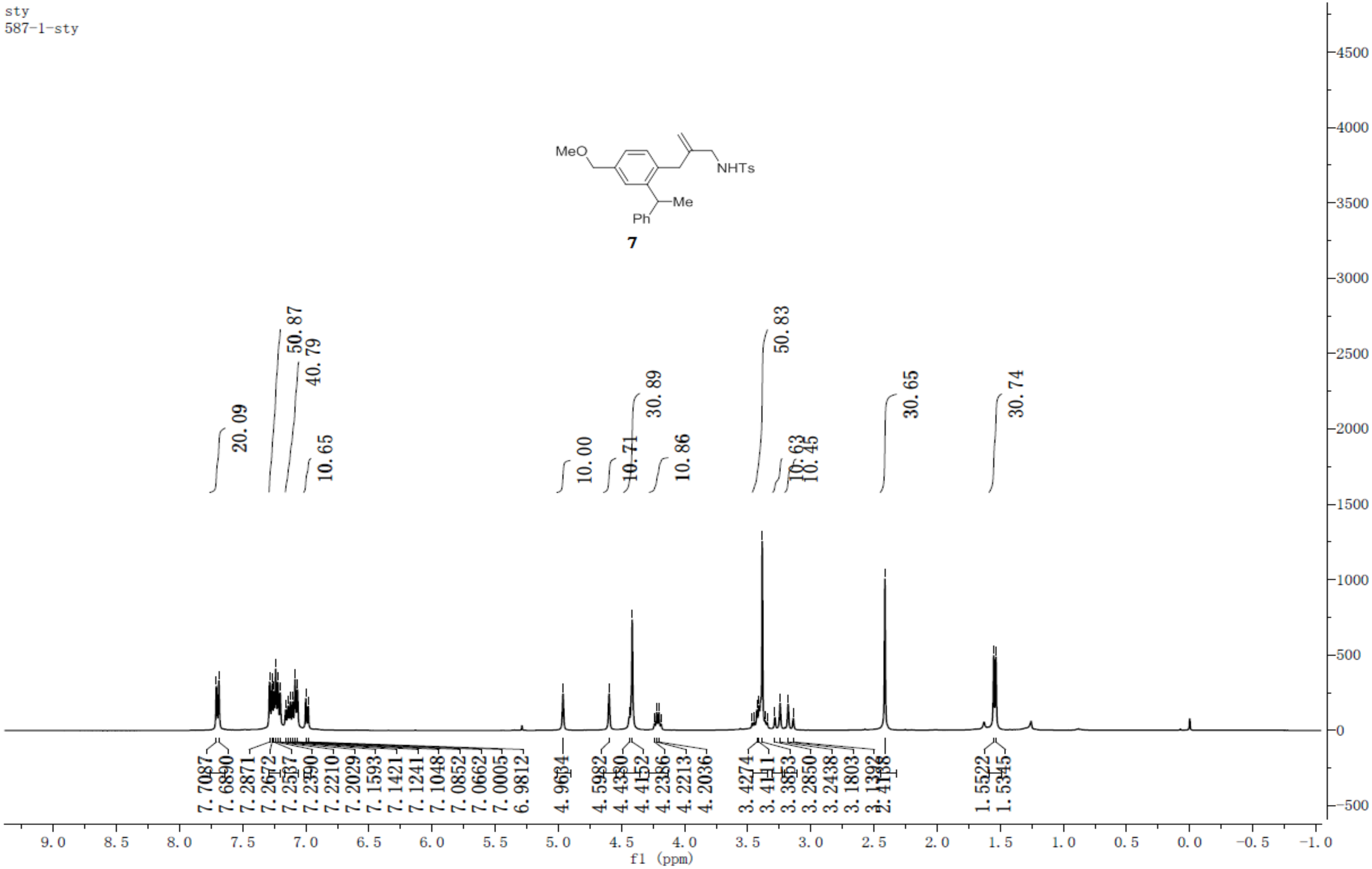

Supplementary Figure 13. ^{1}H NMR Spectrum of substrate 7

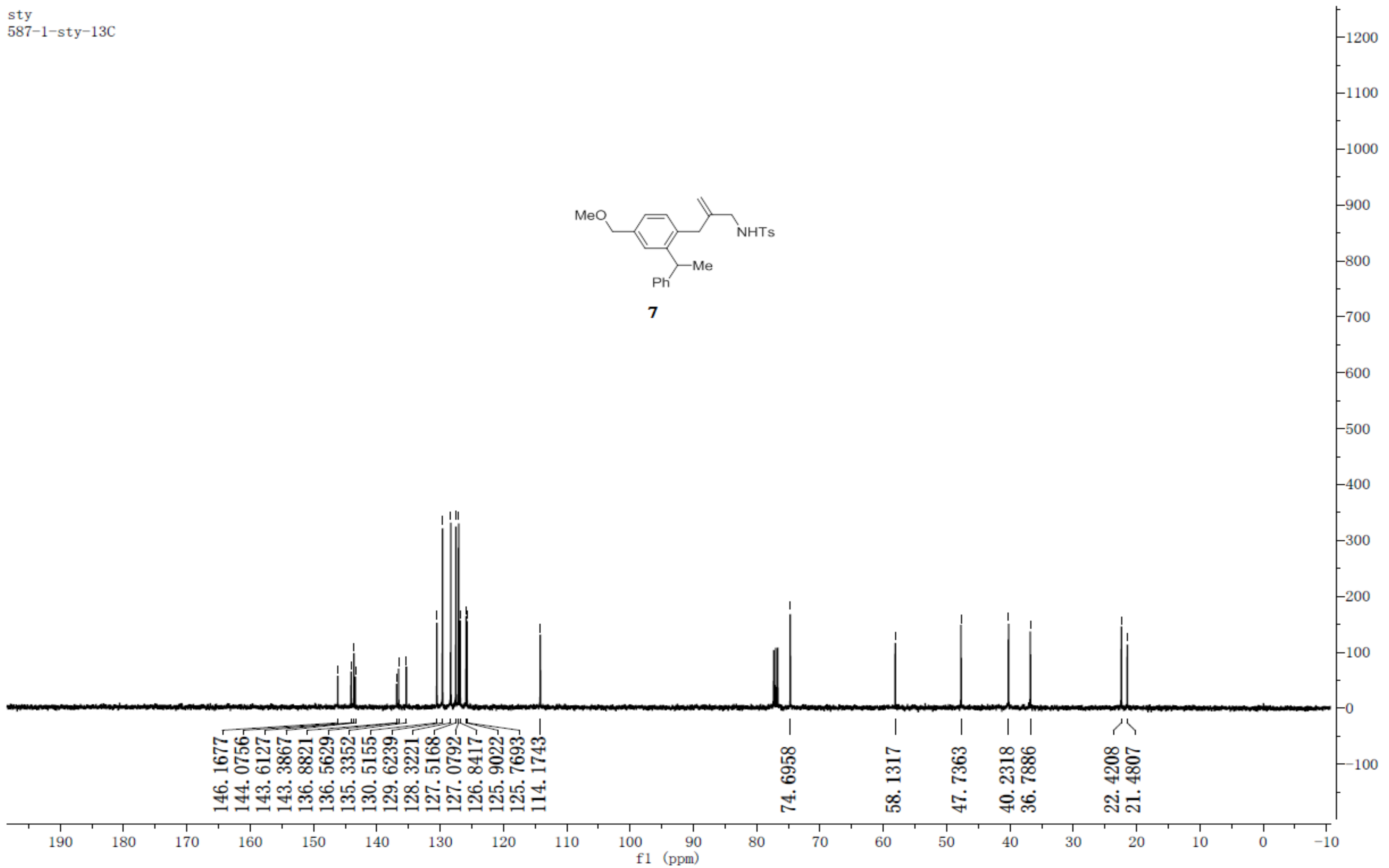

Supplementary Figure 14. ^{13}C NMR Spectrum of substrate 7

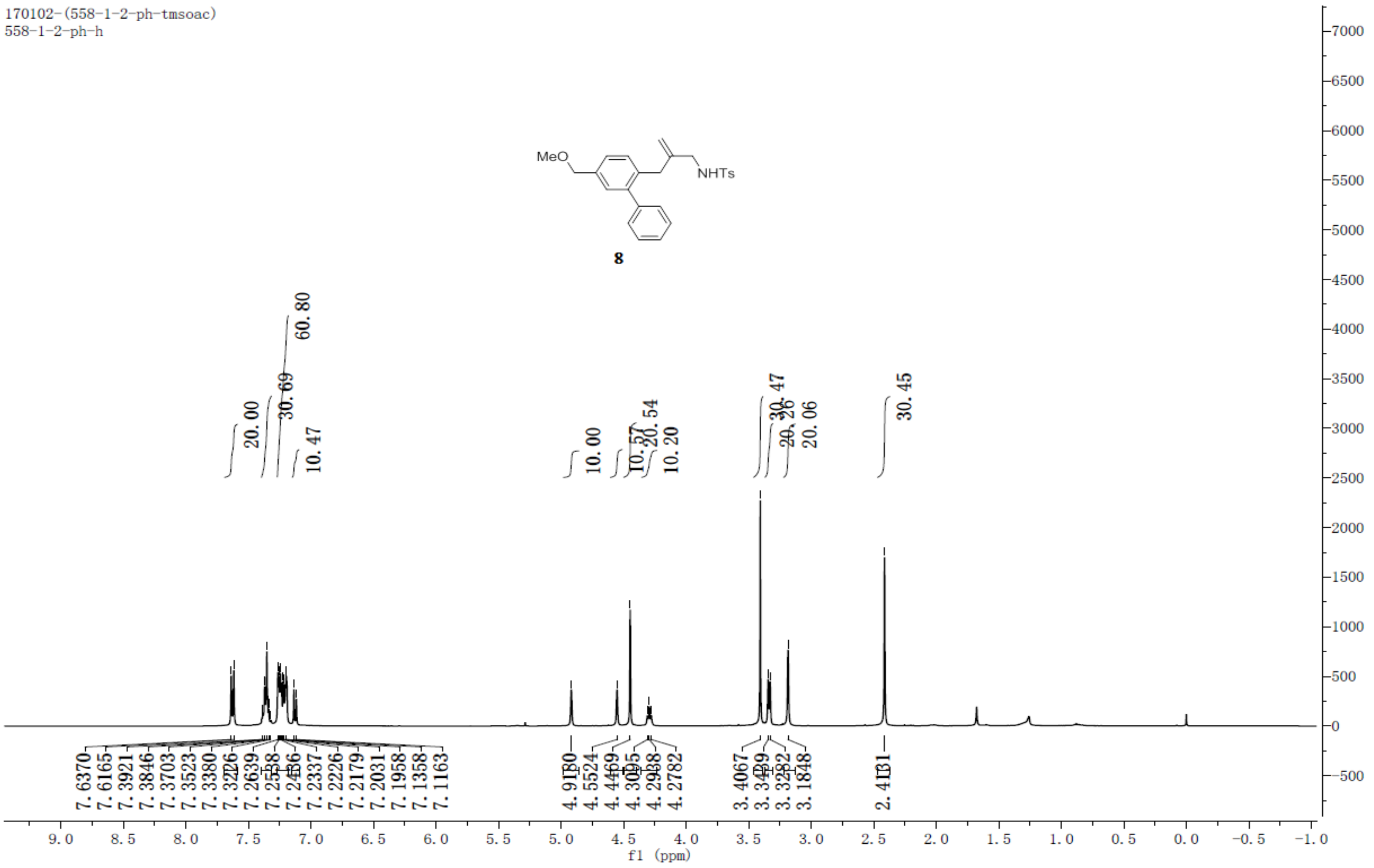

Supplementary Figure 15. ^{1}H NMR Spectrum of substrate 8

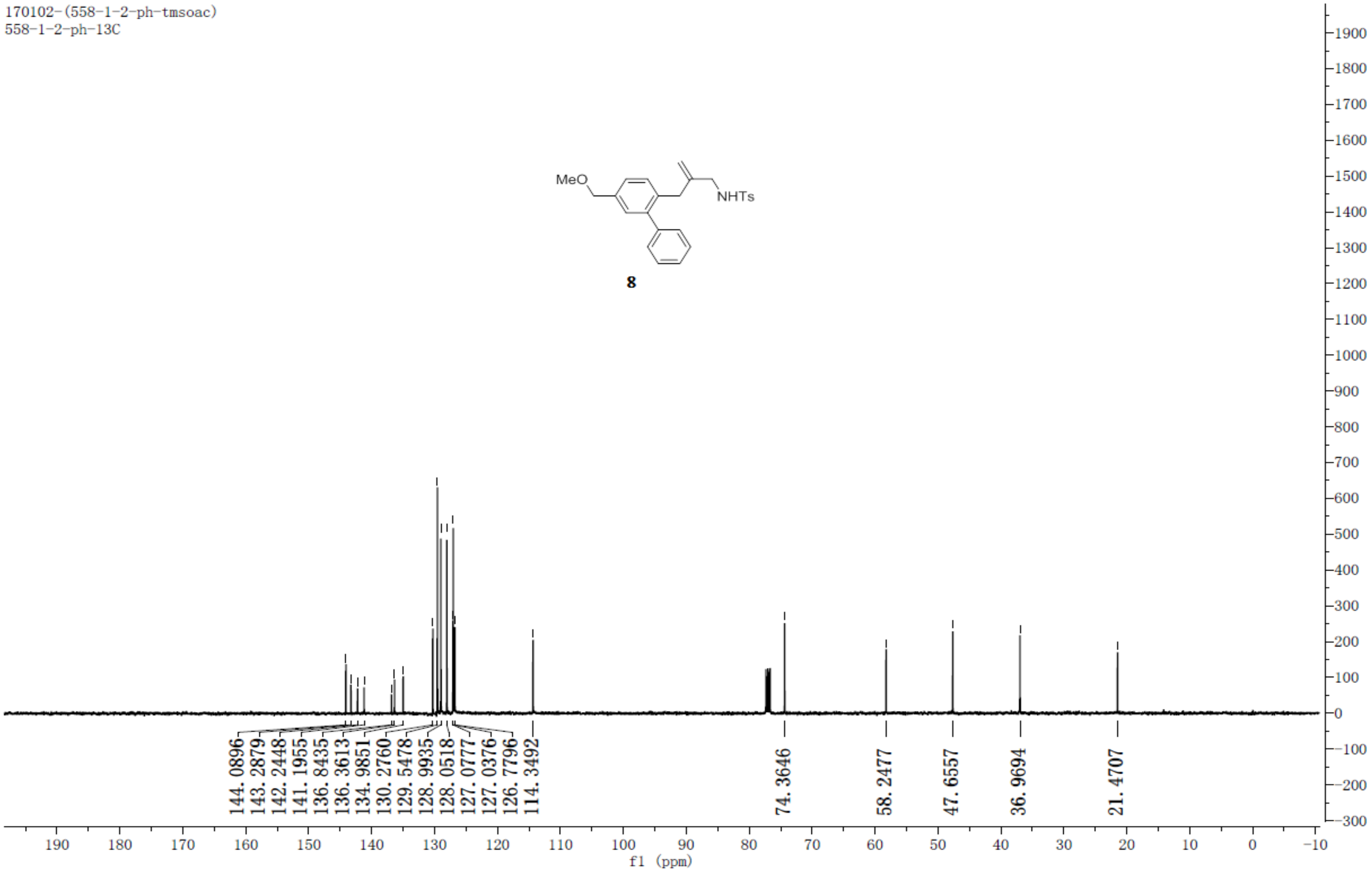

Supplementary Figure 16. ^{13}C NMR Spectrum of substrate 8

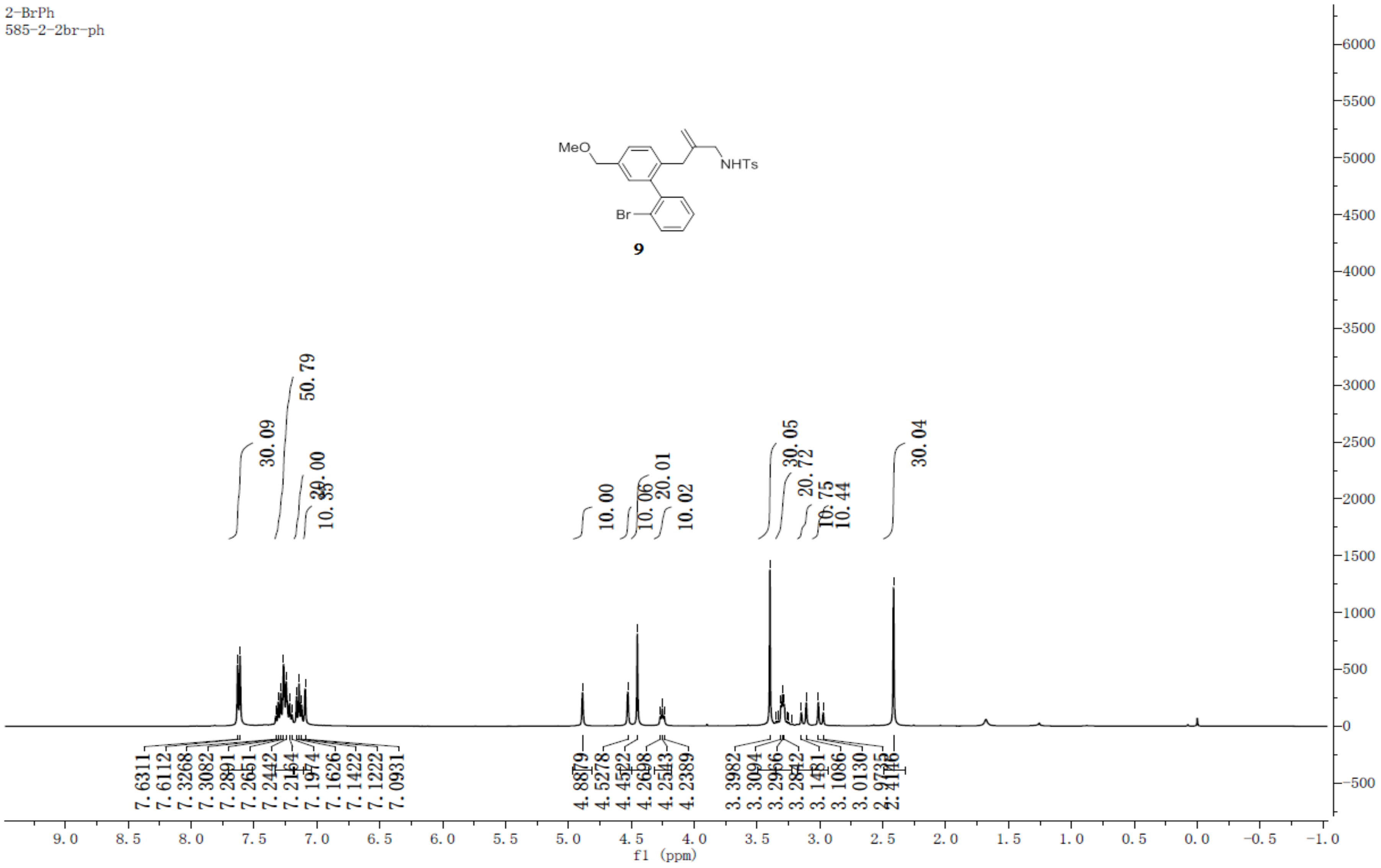

Supplementary Figure 17. ^{1}H NMR Spectrum of substrate 9

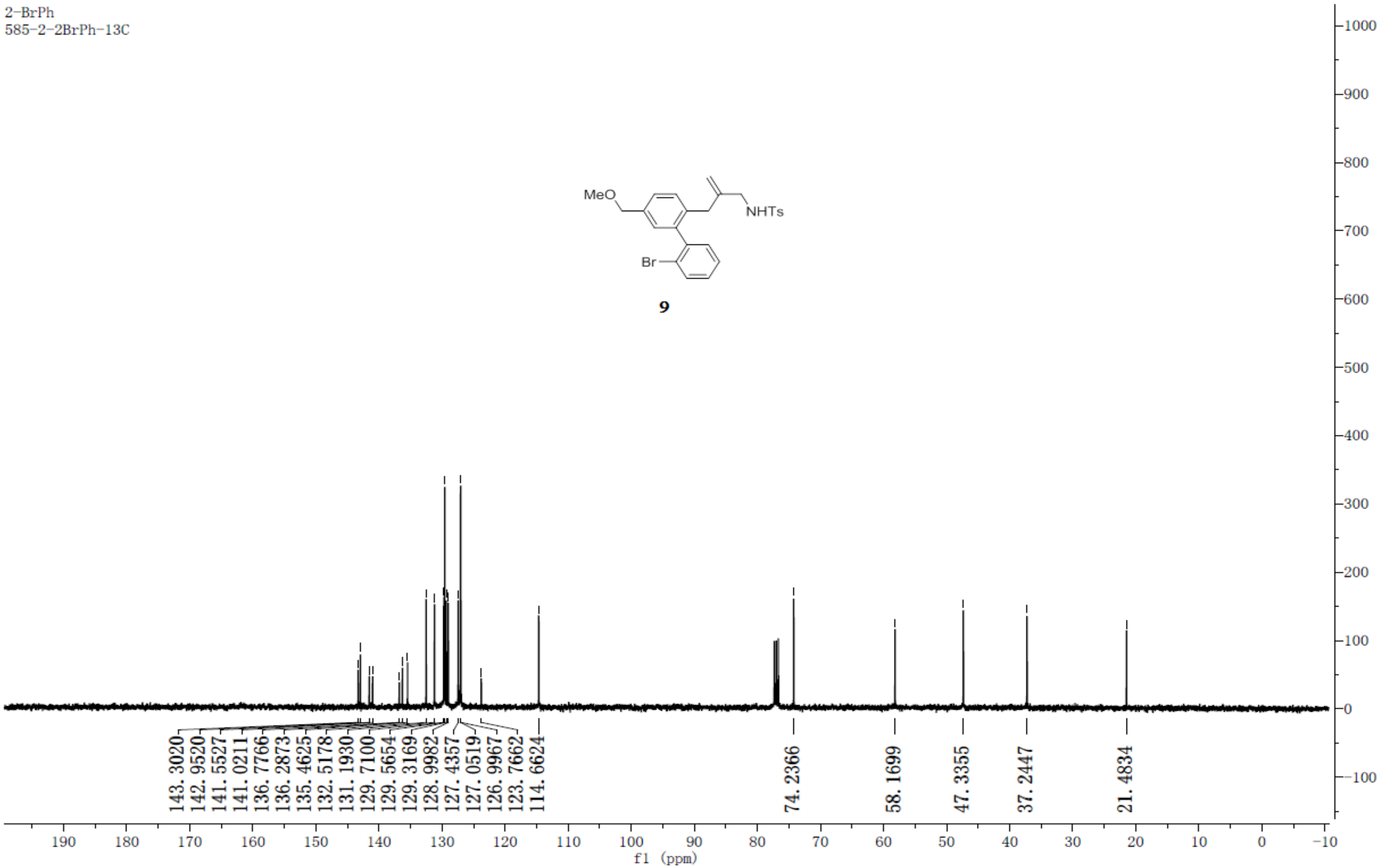

Supplementary Figure 18. ^{13}C NMR Spectrum of substrate 9

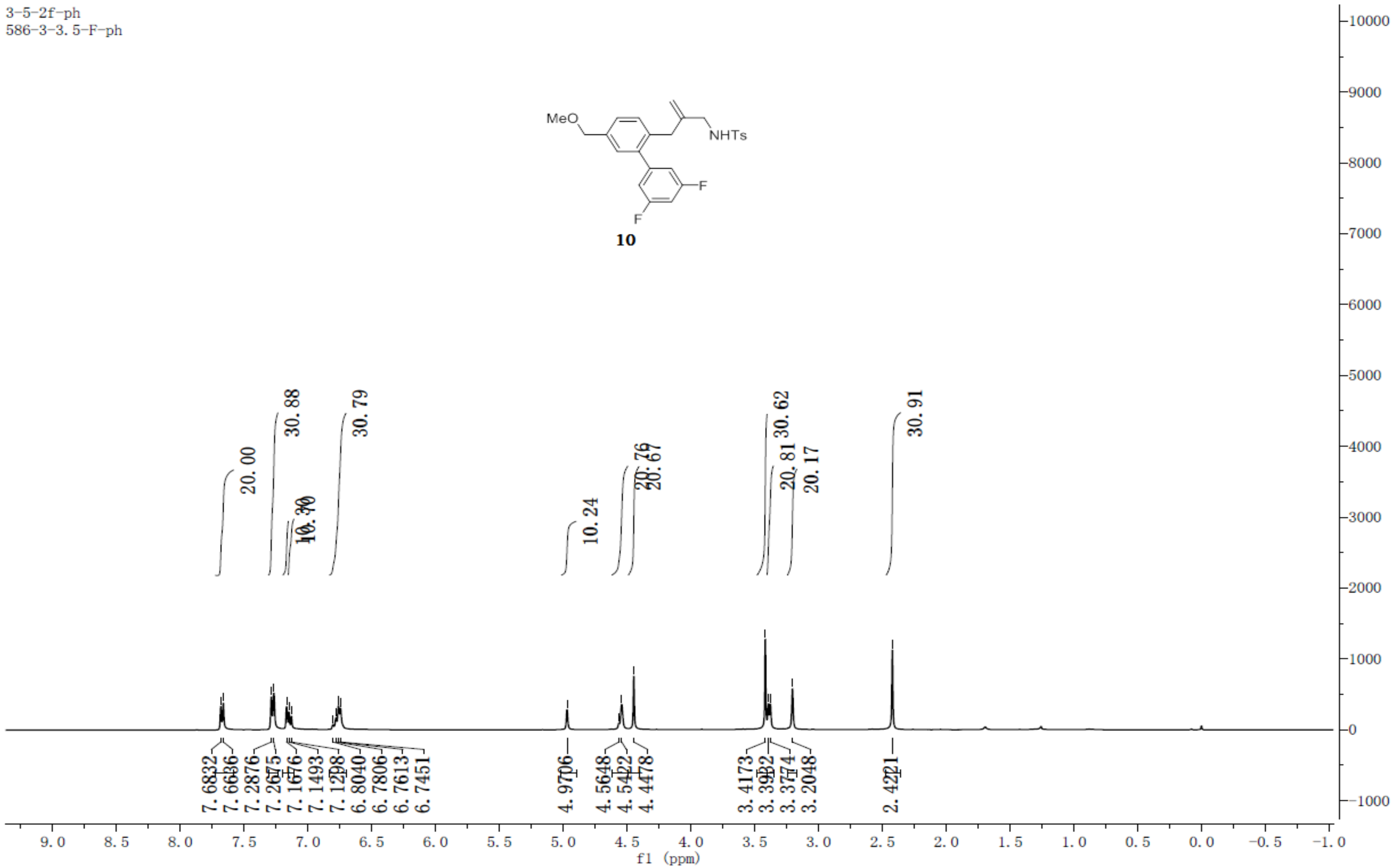

Supplementary Figure 19. ^{1}H NMR Spectrum of substrate 10

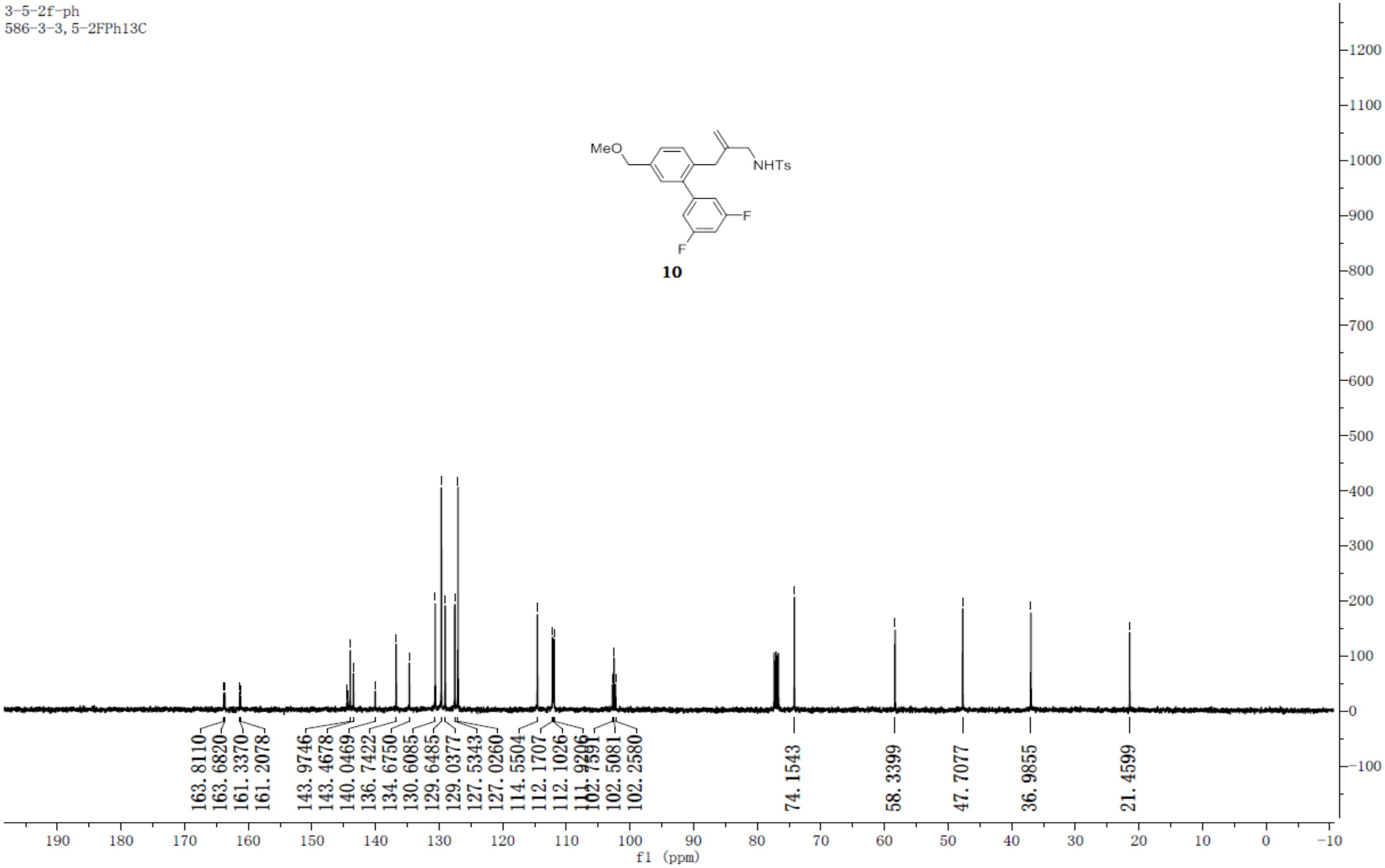

Supplementary Figure 20. ^{13}C NMR Spectrum of substrate 10

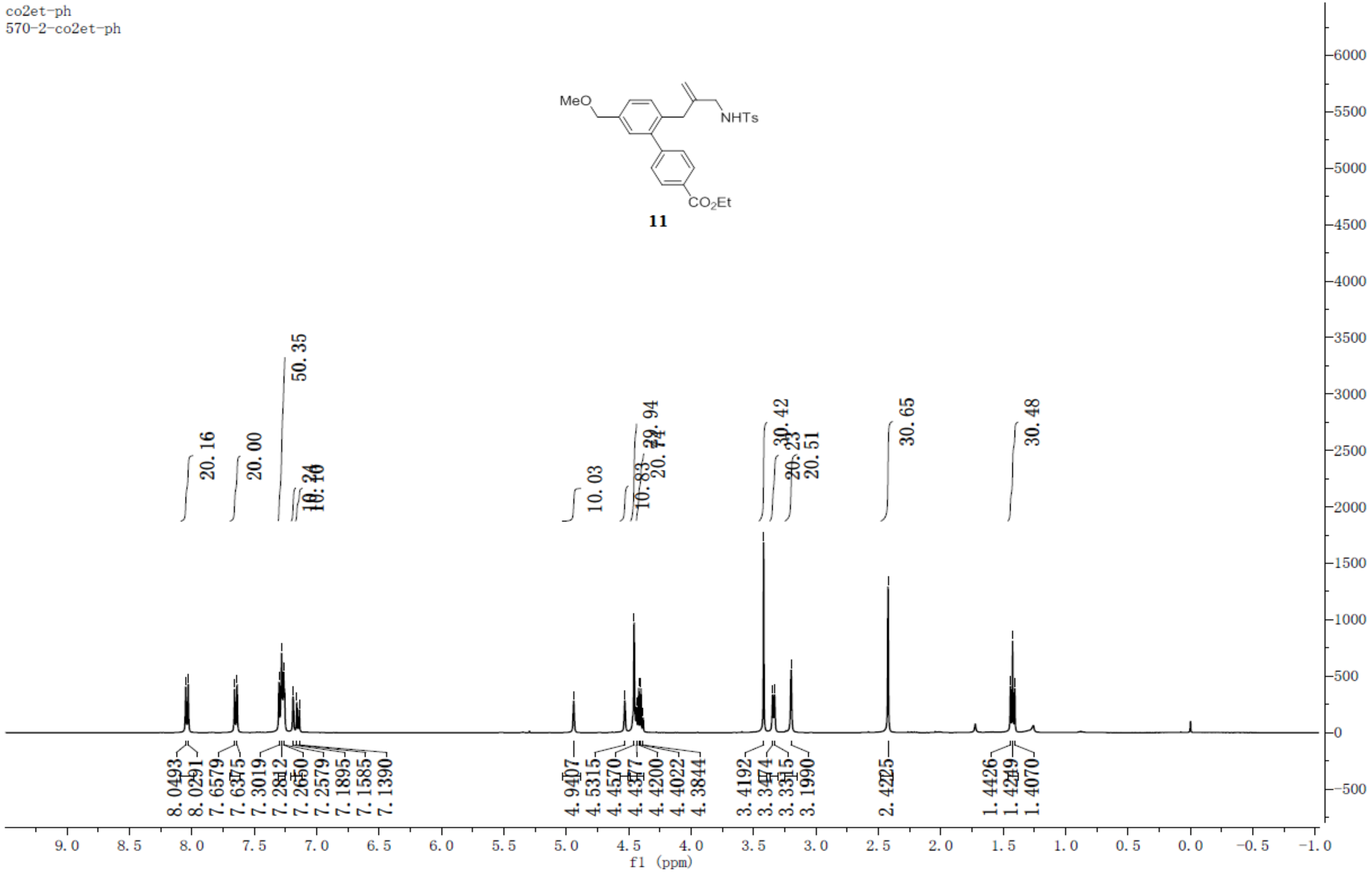

Supplementary Figure 21. ^{1}H NMR Spectrum of substrate 11

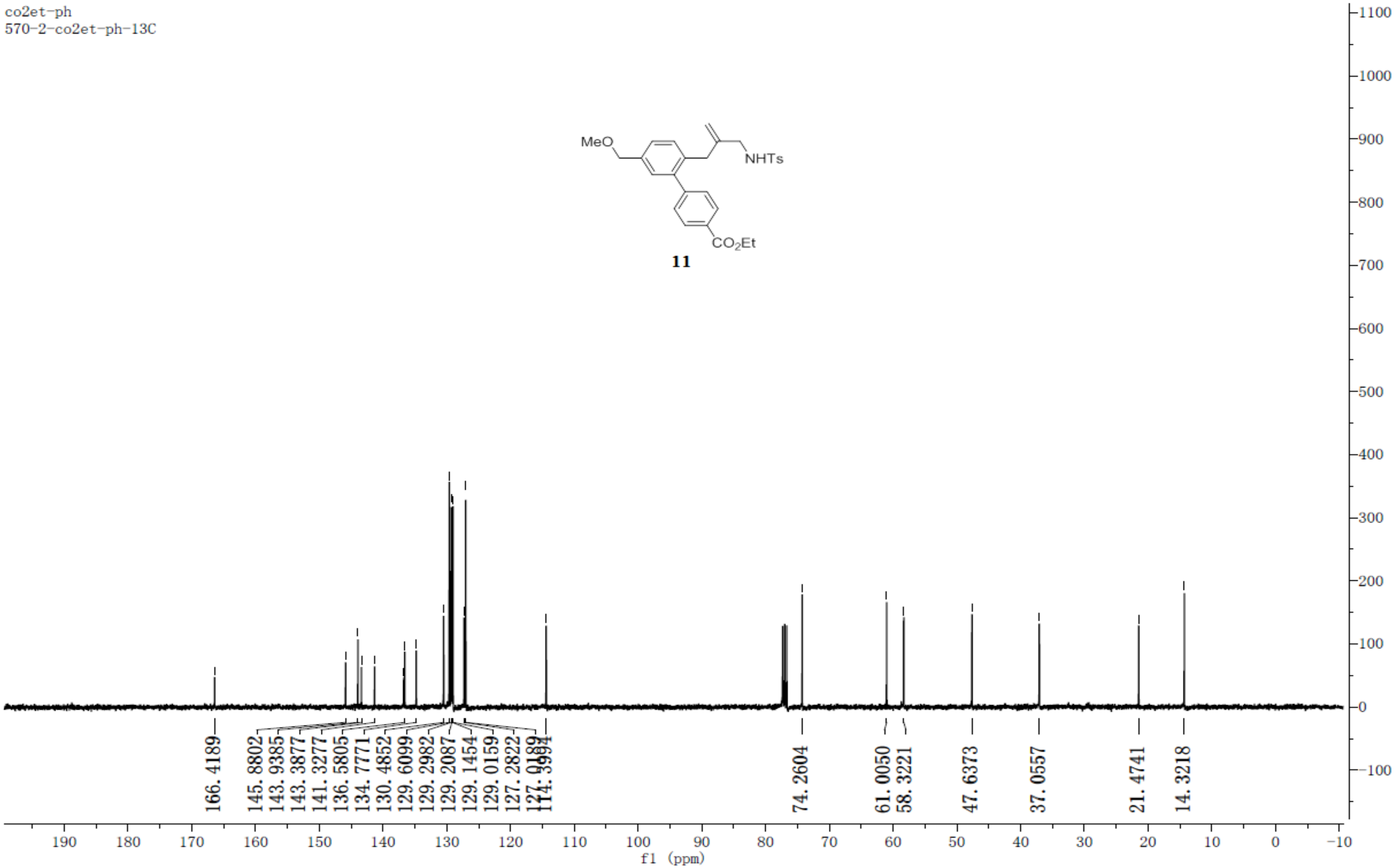

Supplementary Figure 22. ^{13}C NMR Spectrum of substrate 11

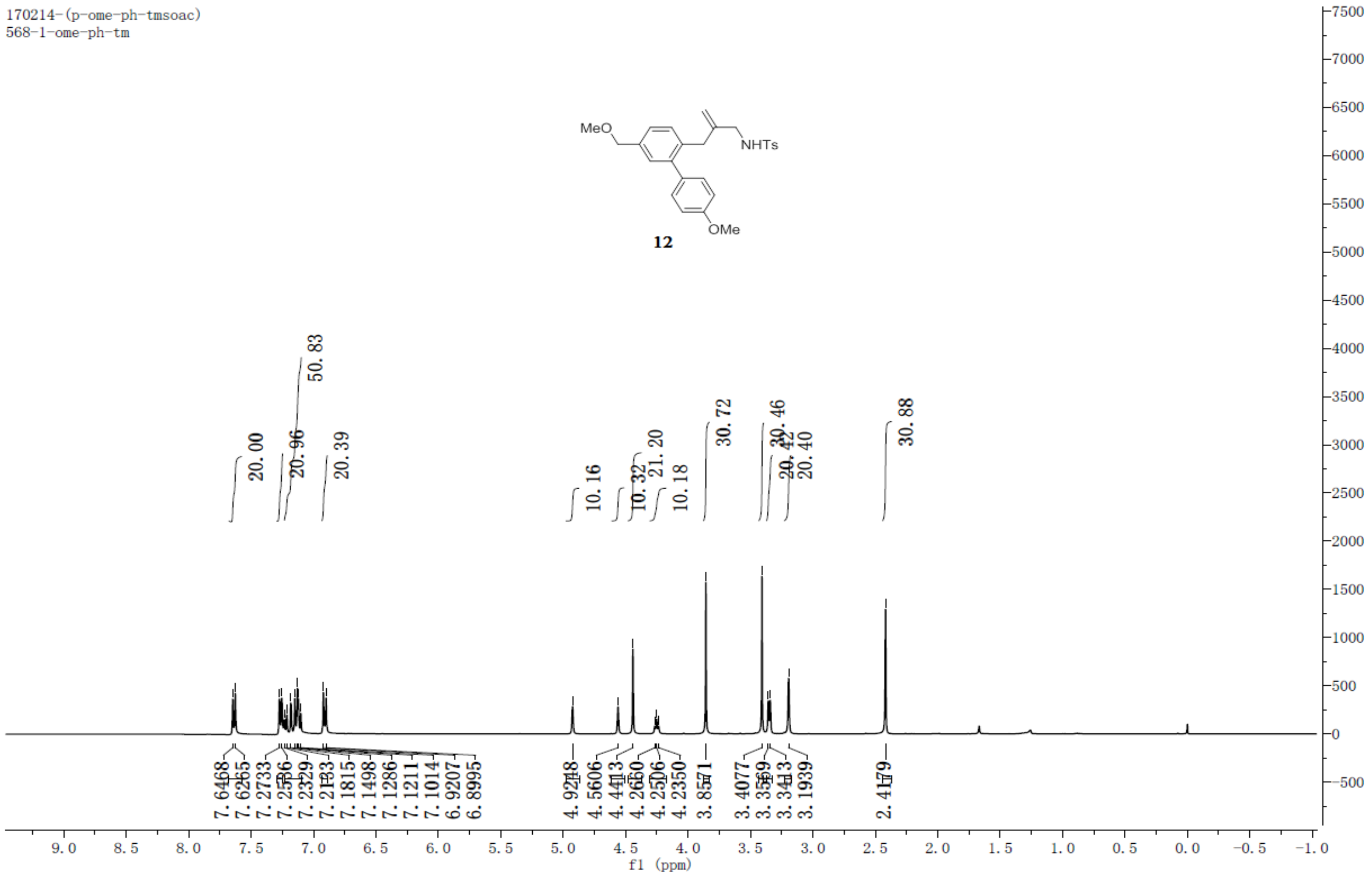

Supplementary Figure 23. ^{1}H NMR Spectrum of substrate 12

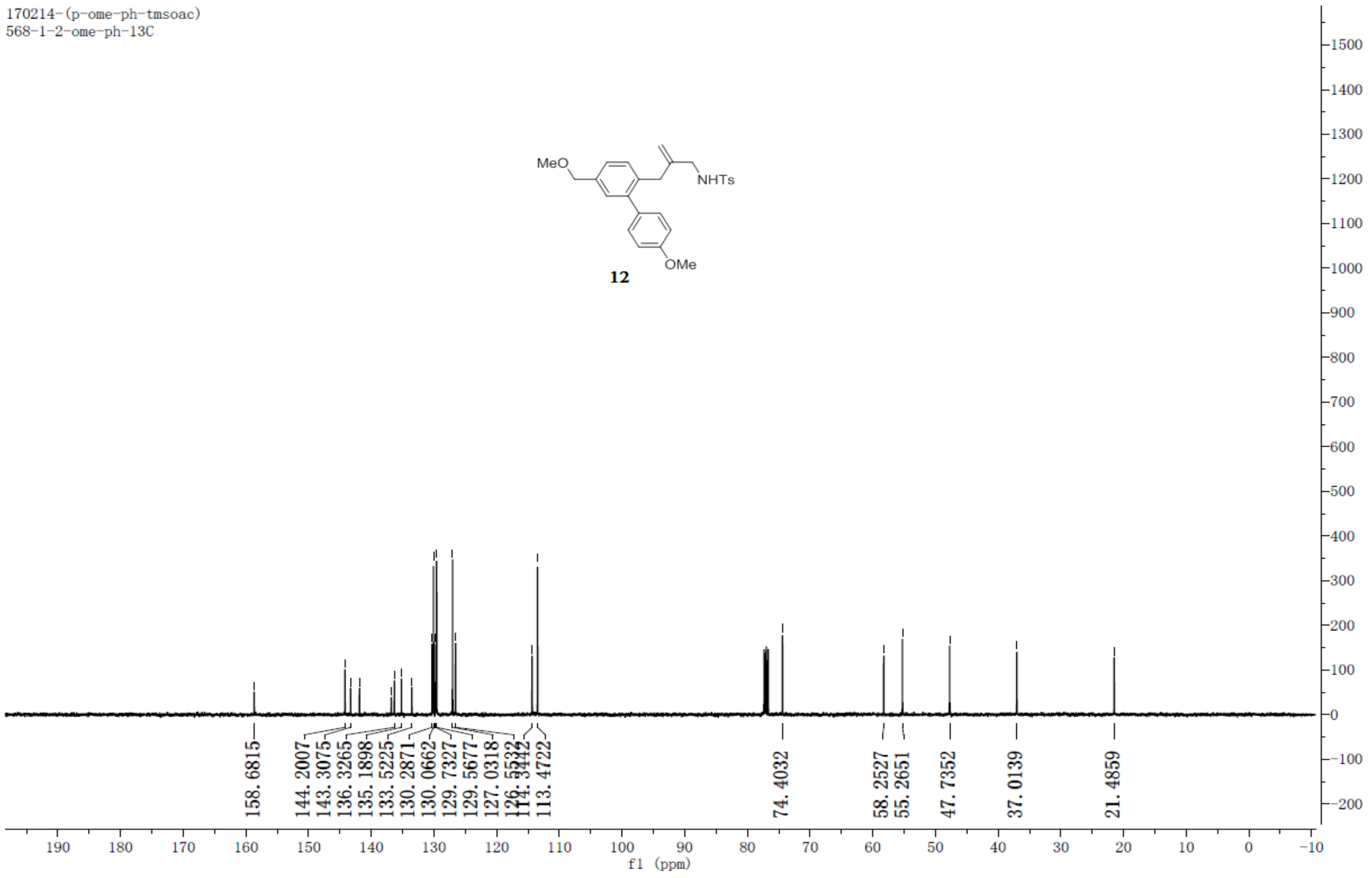

Supplementary Figure 24. ^{13}C NMR Spectrum of substrate 12

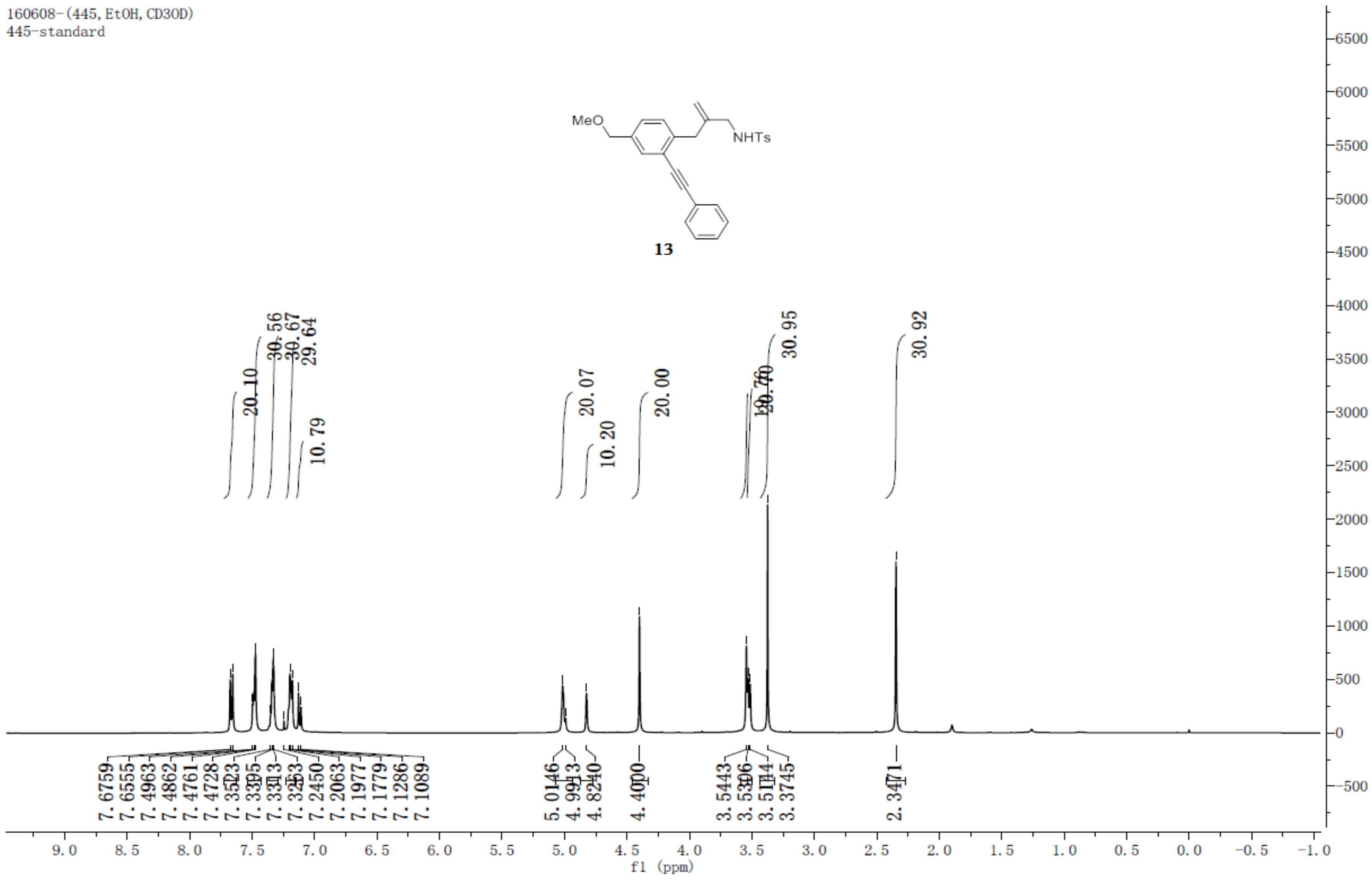

Supplementary Figure 25. ^{1}H NMR Spectrum of substrate 13

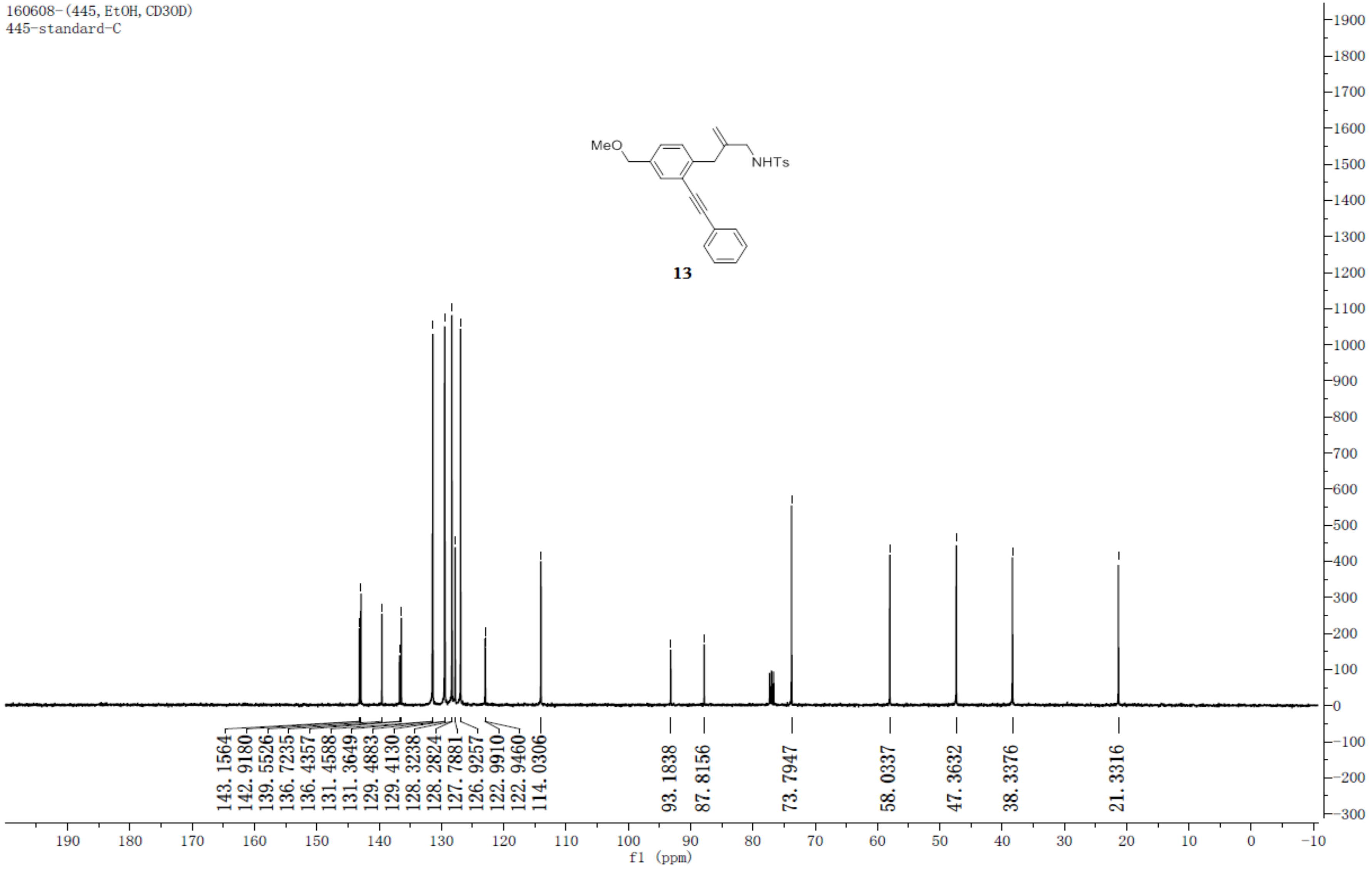

Supplementary Figure 26. ^{13}C NMR Spectrum of substrate 13

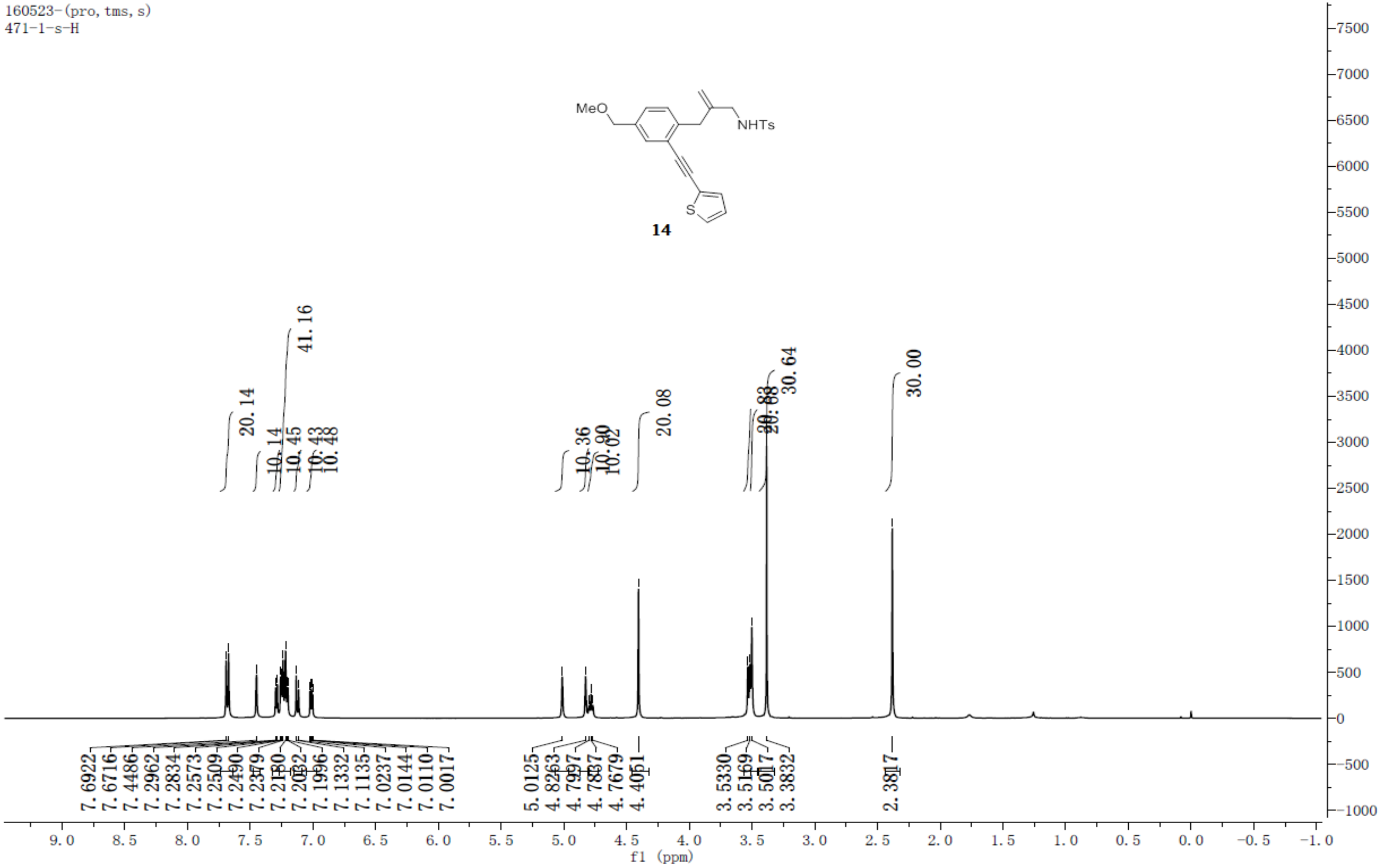

Supplementary Figure 27. ¹H NMR Spectrum of substrate 14

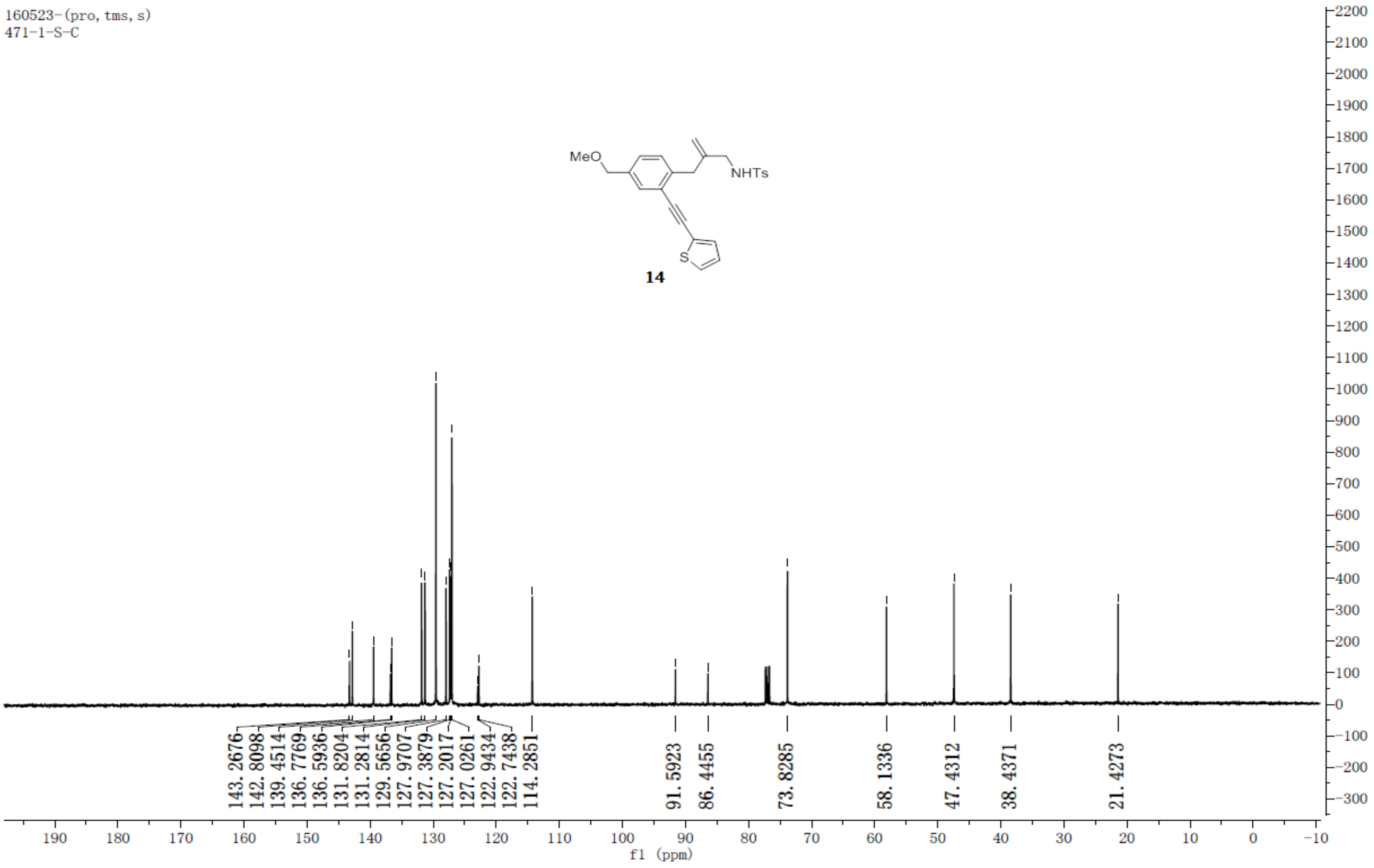

Supplementary Figure 28. ^{13}C NMR Spectrum of substrate 14

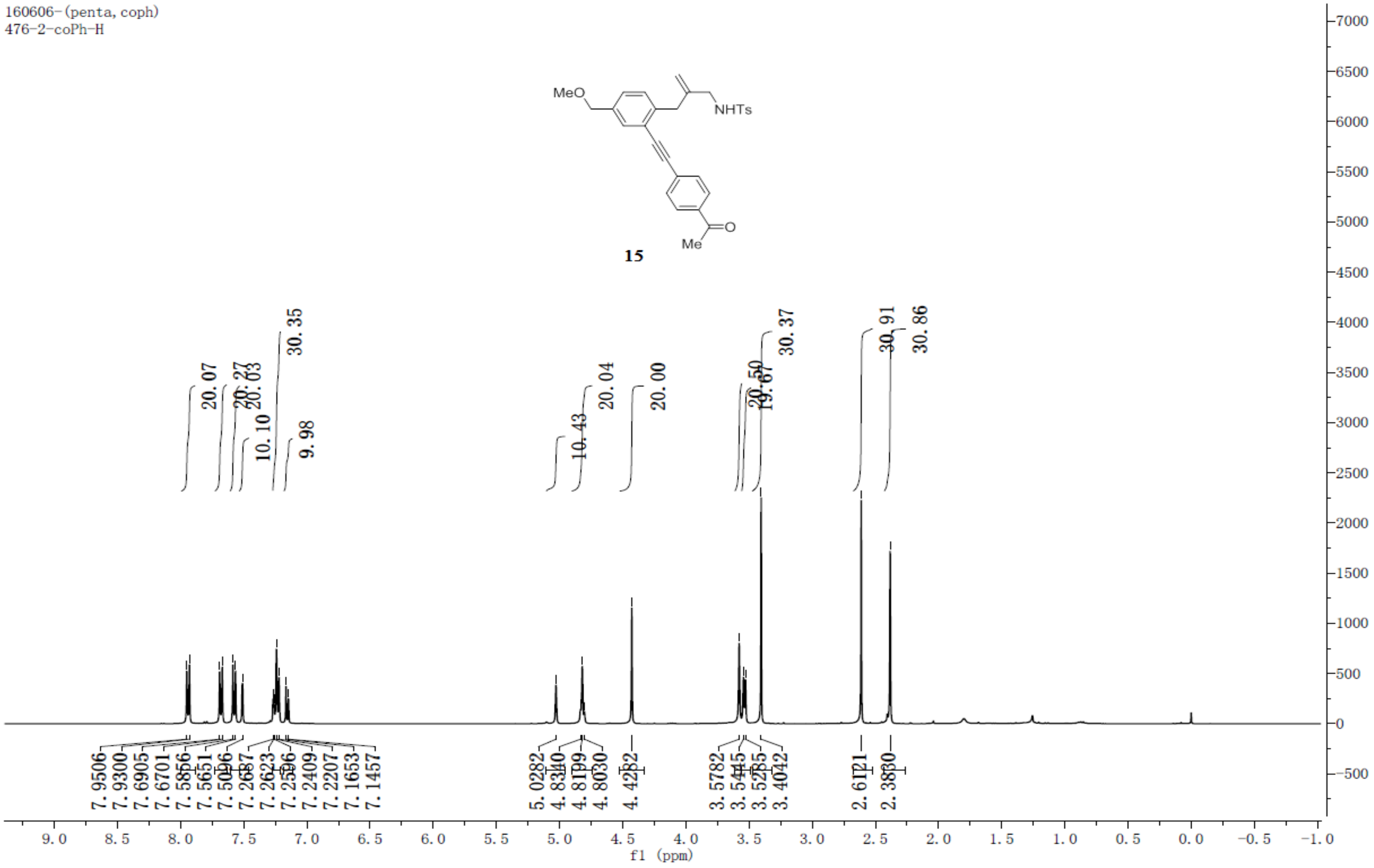

Supplementary Figure 29. ^{1}H NMR Spectrum of substrate 15

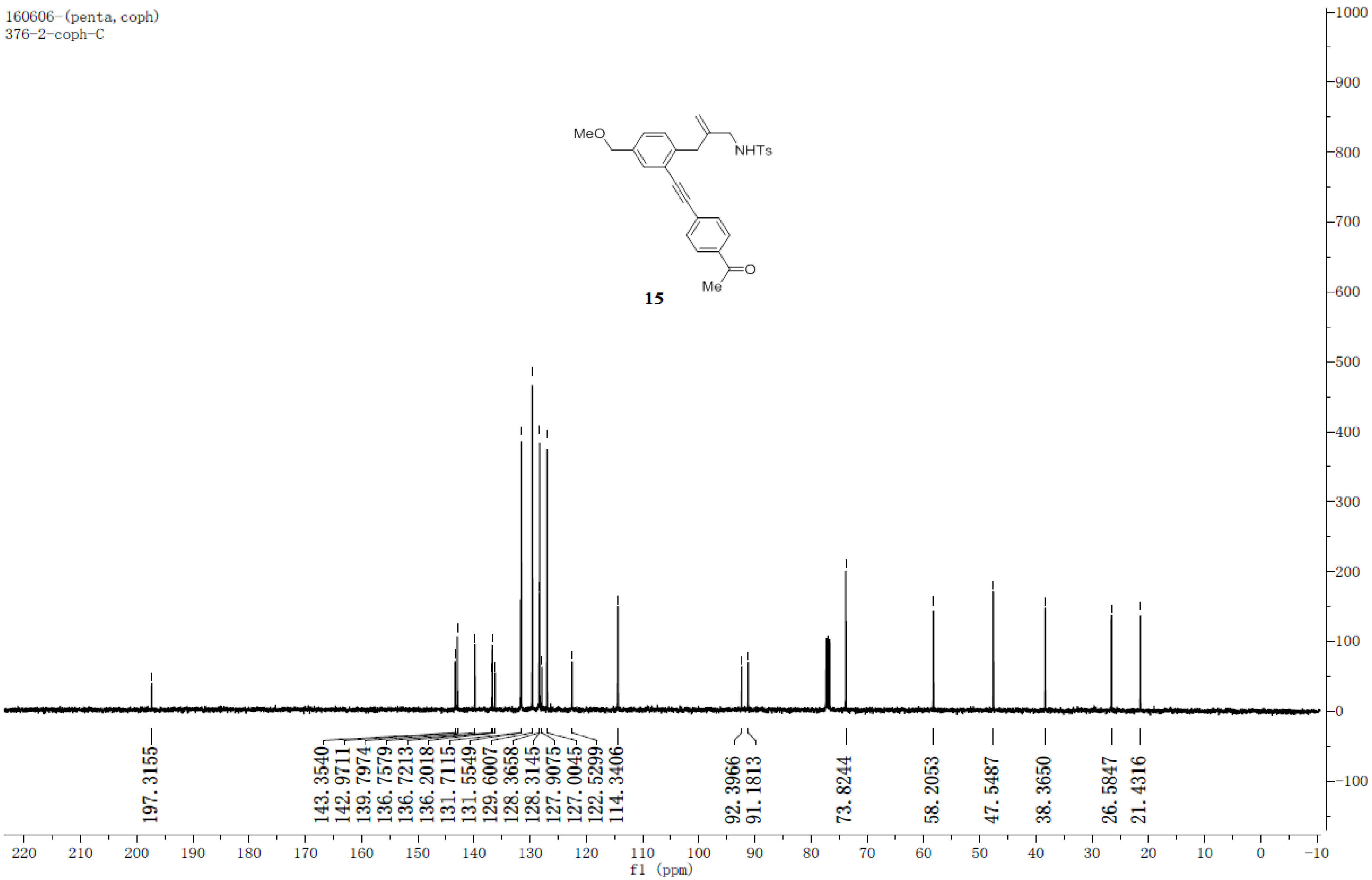

Supplementary Figure 30. ^{13}C NMR Spectrum of substrate 15

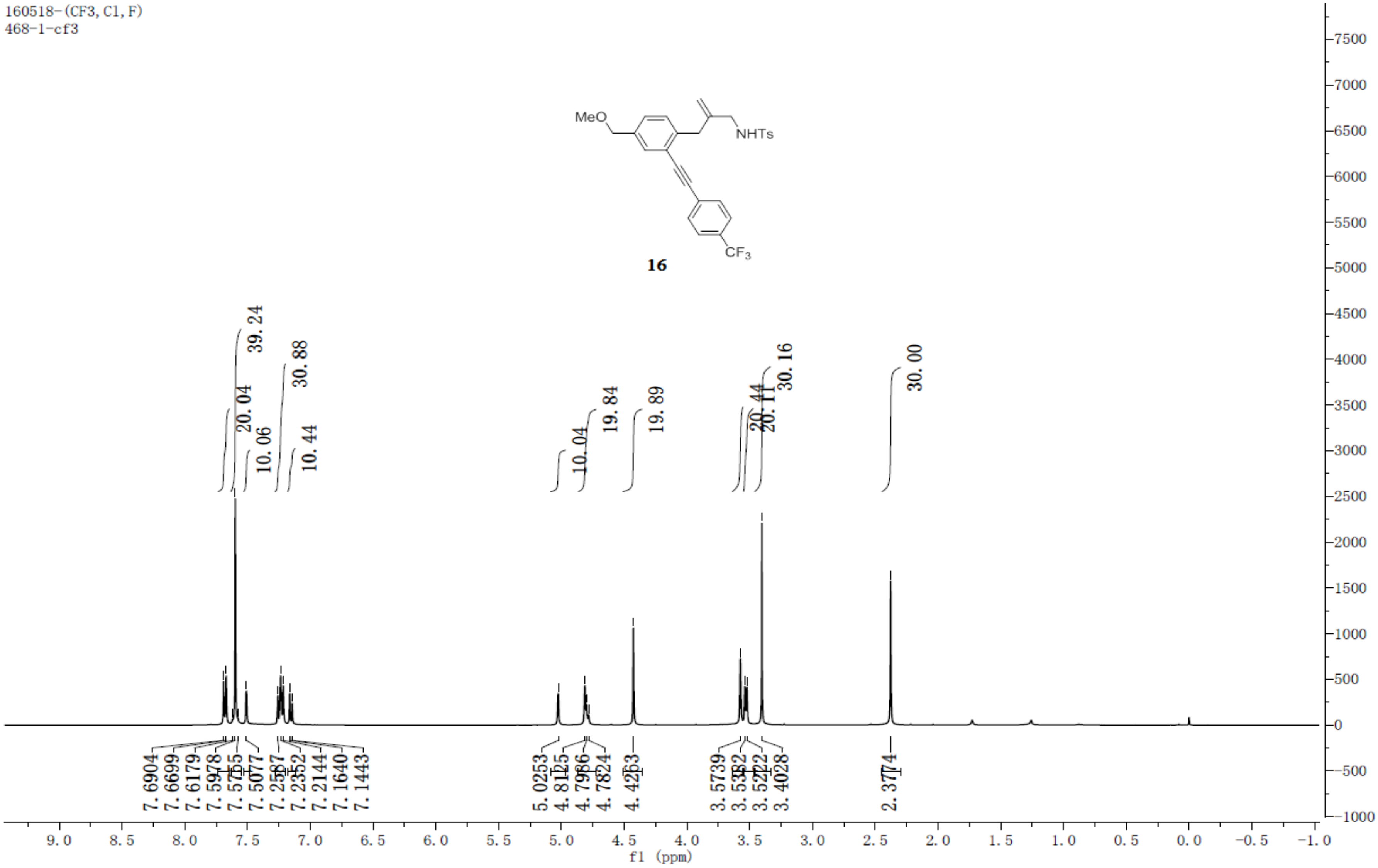

Supplementary Figure 31. ^{1}H NMR Spectrum of substrate 16

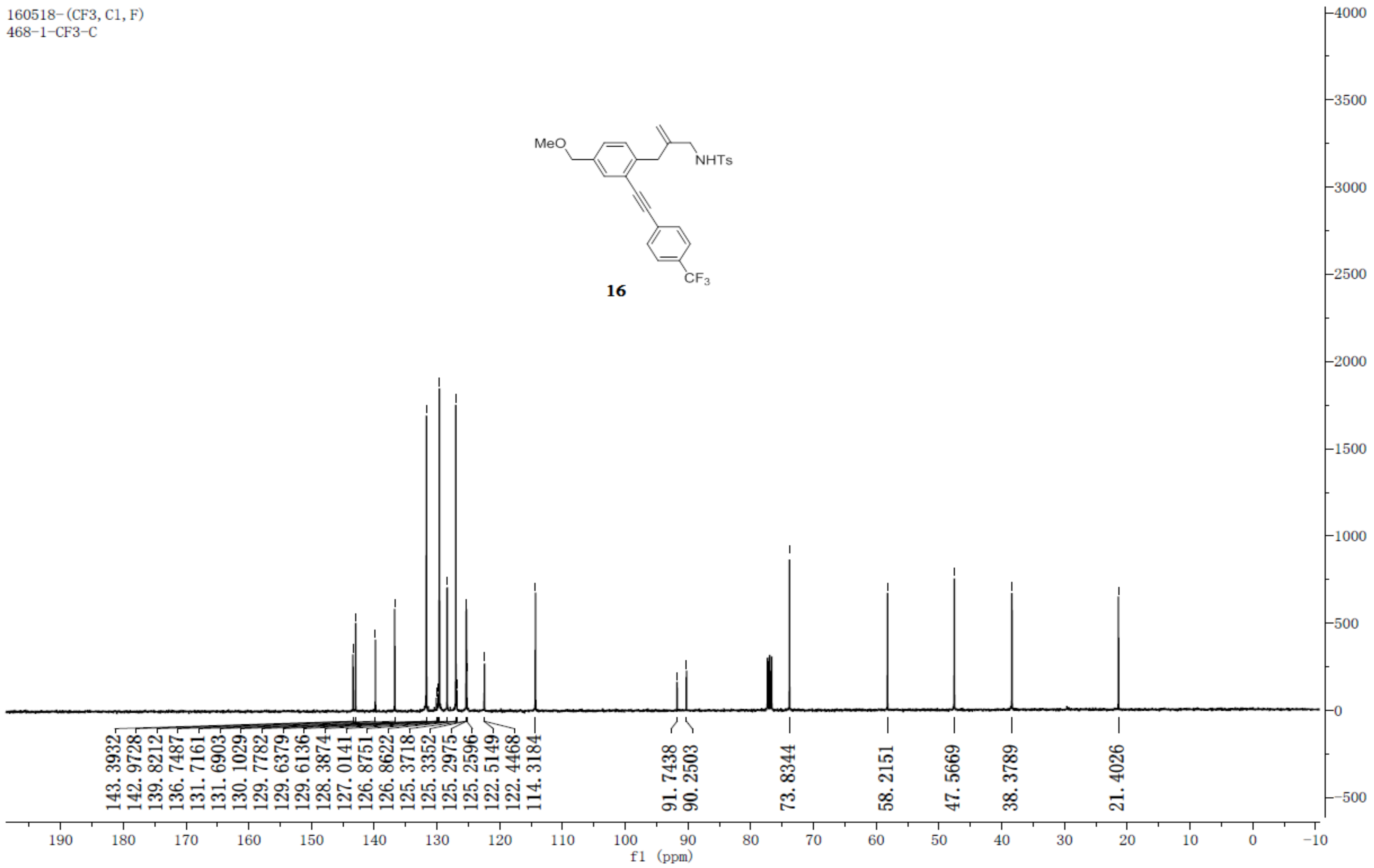

Supplementary Figure 32. ^{13}C NMR Spectrum of substrate 16

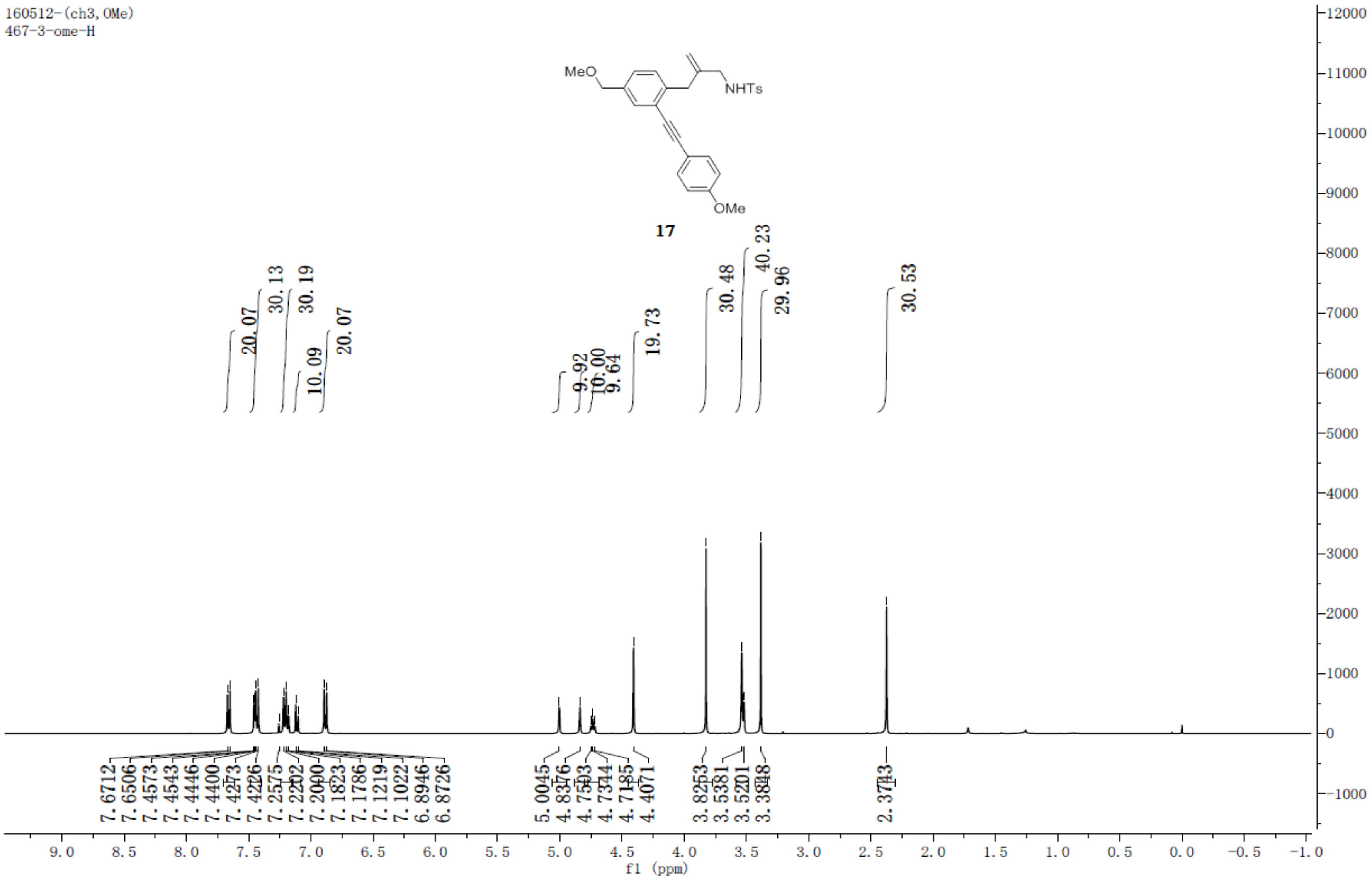

Supplementary Figure 33. ^{1}H NMR Spectrum of substrate 17

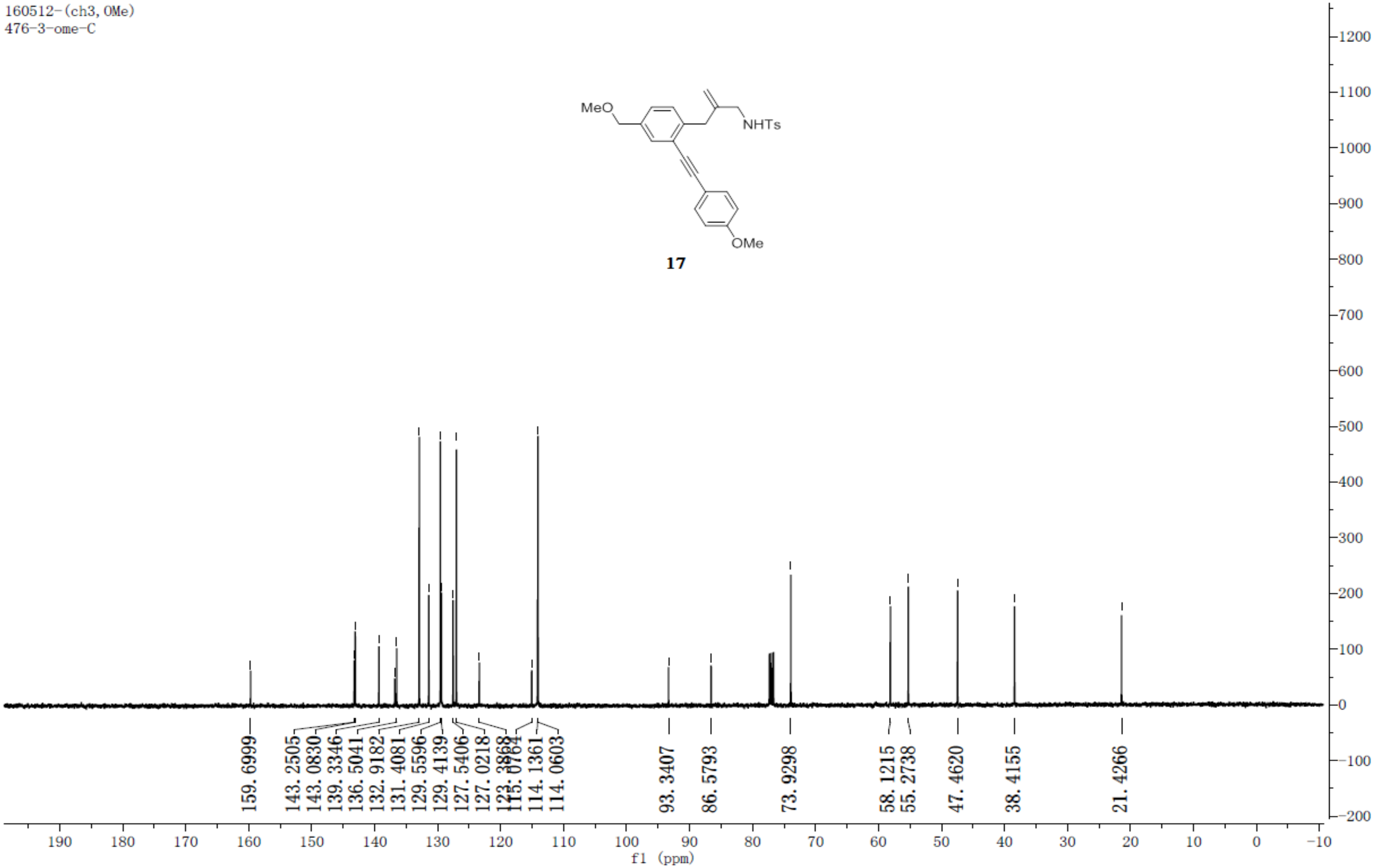

Supplementary Figure 34. ^{13}C NMR Spectrum of substrate 17

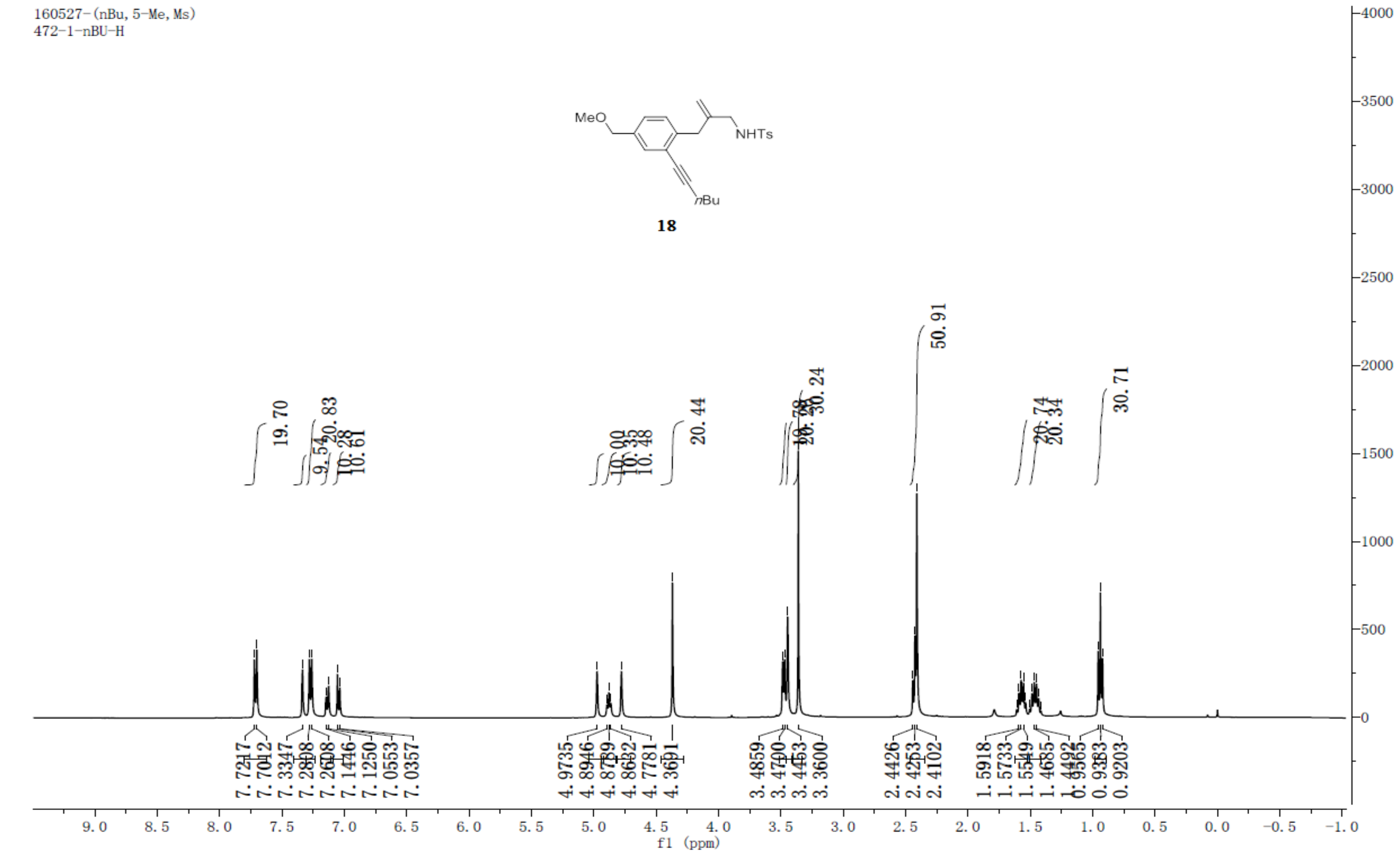

Supplementary Figure 35. ¹H NMR Spectrum of substrate 18

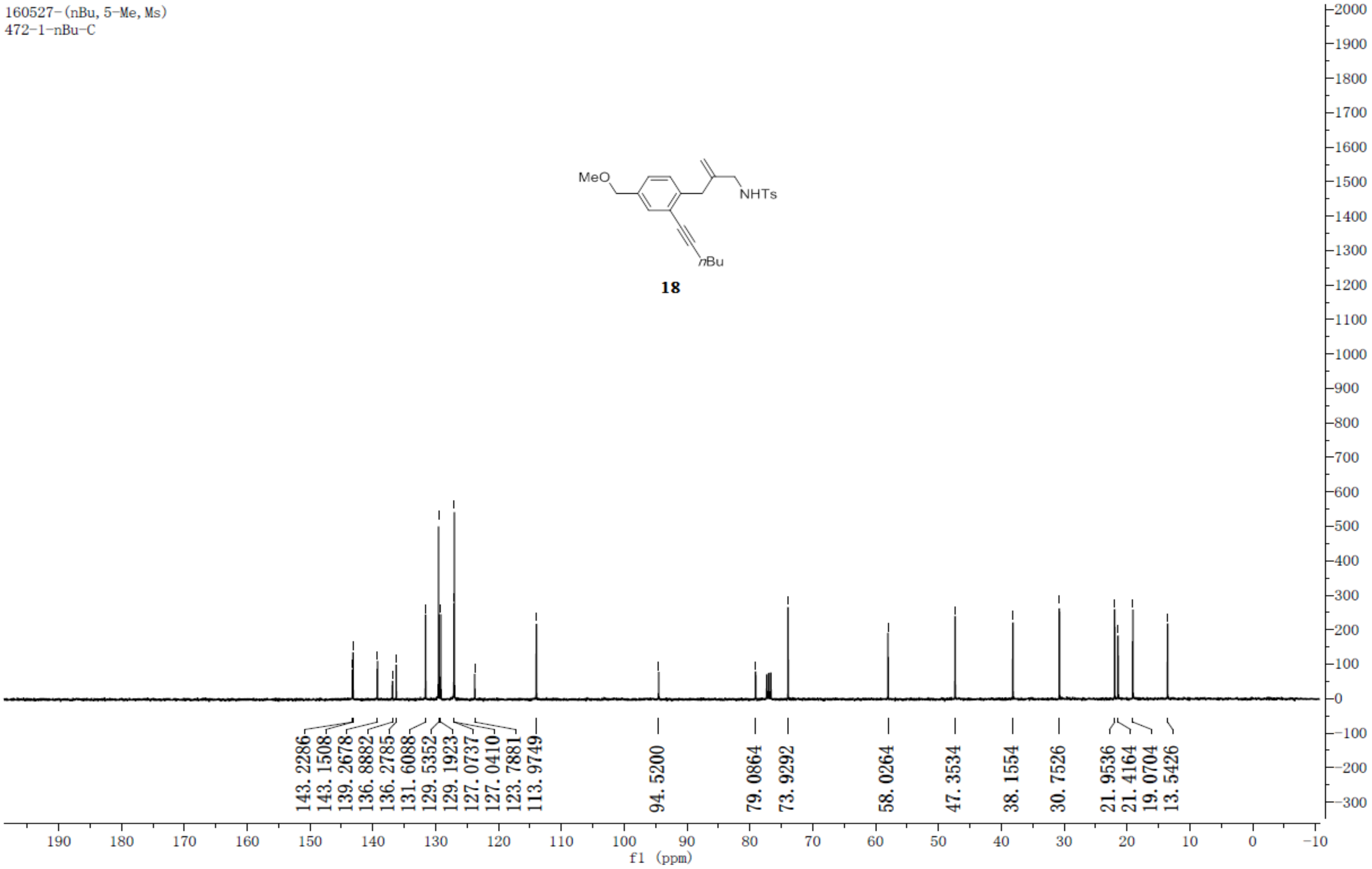

Supplementary Figure 36. ^{13}C NMR Spectrum of substrate 18

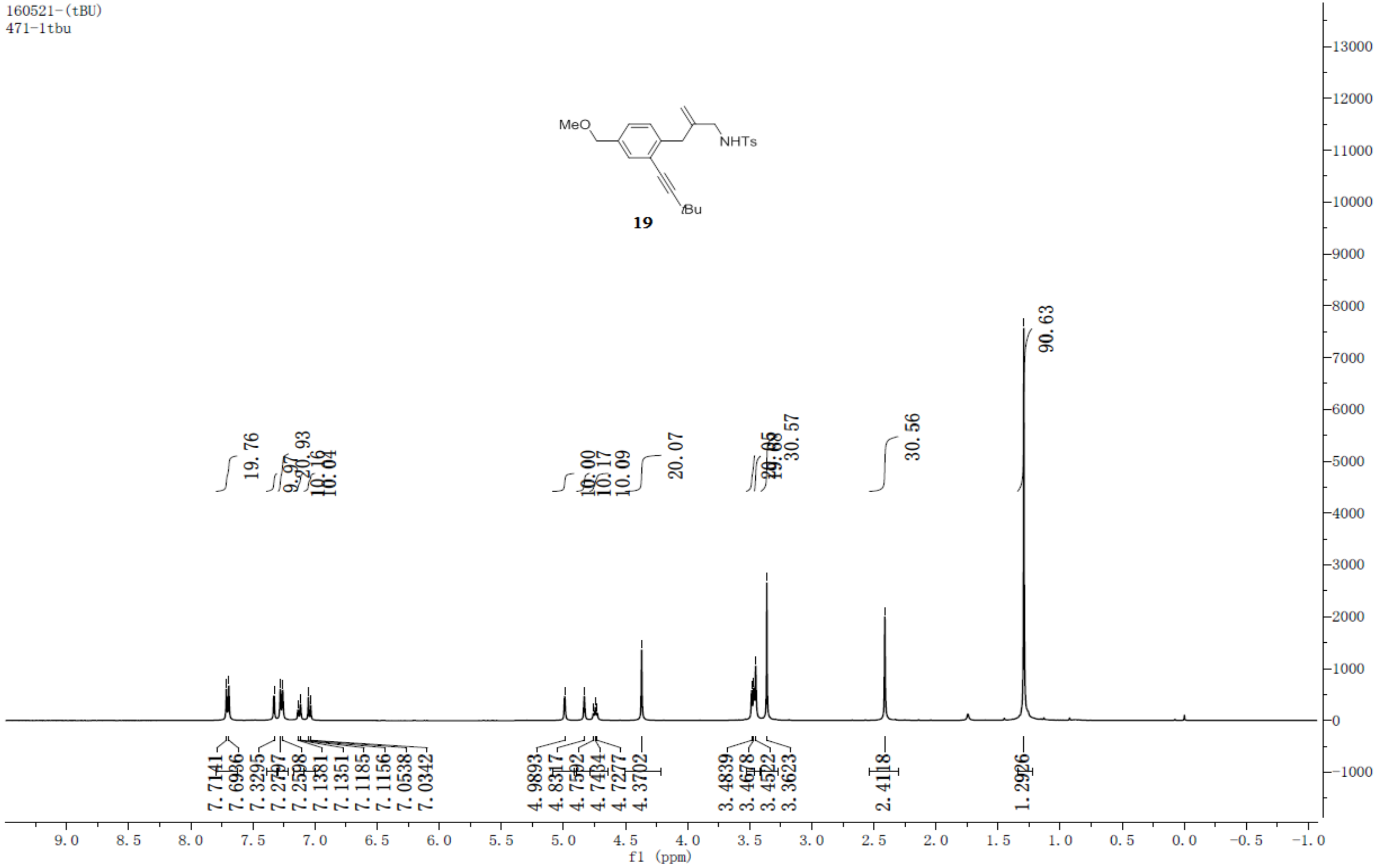

Supplementary Figure 37. ^{1}H NMR Spectrum of substrate 19

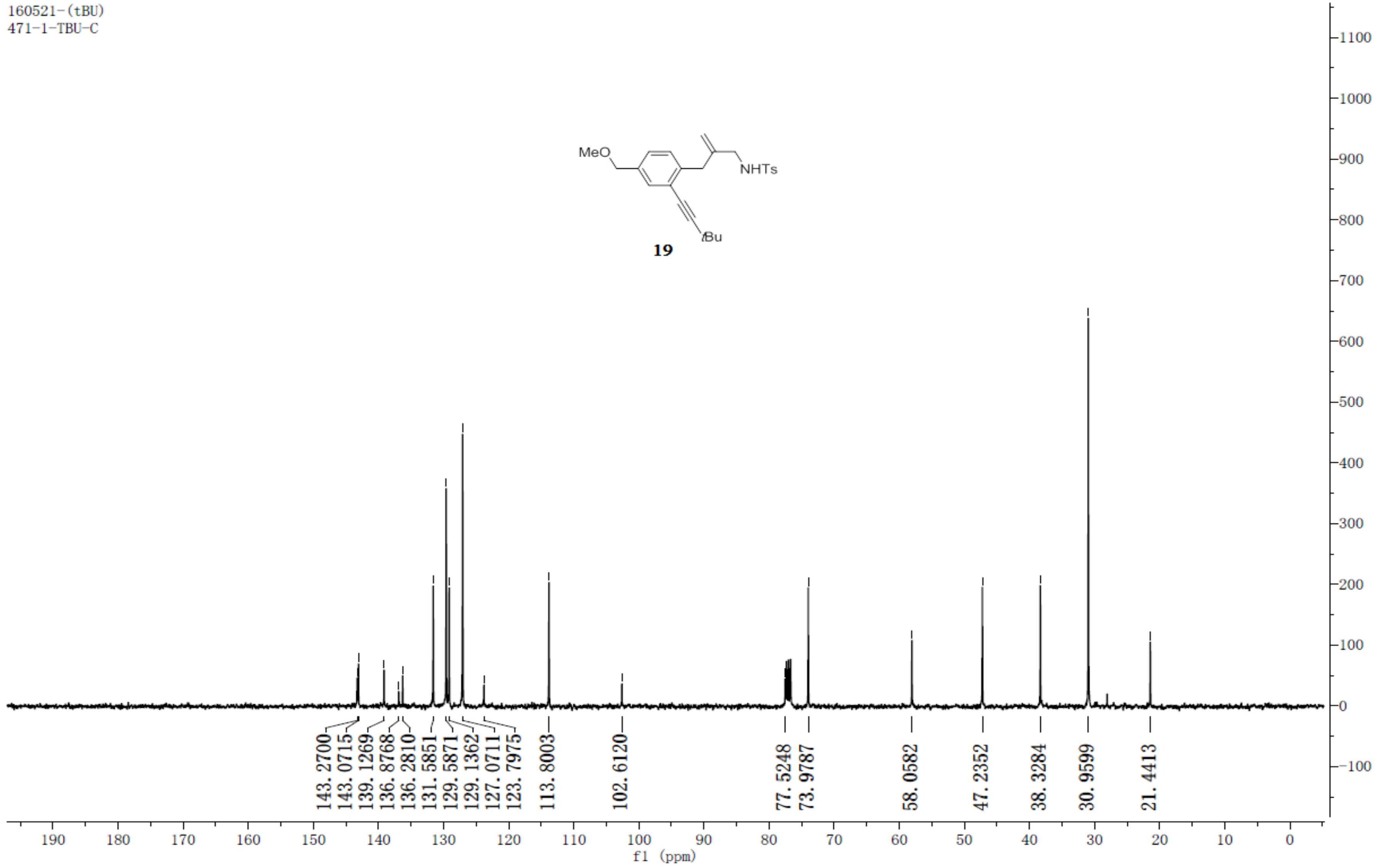

Supplementary Figure 38. ^{13}C NMR Spectrum of substrate 19

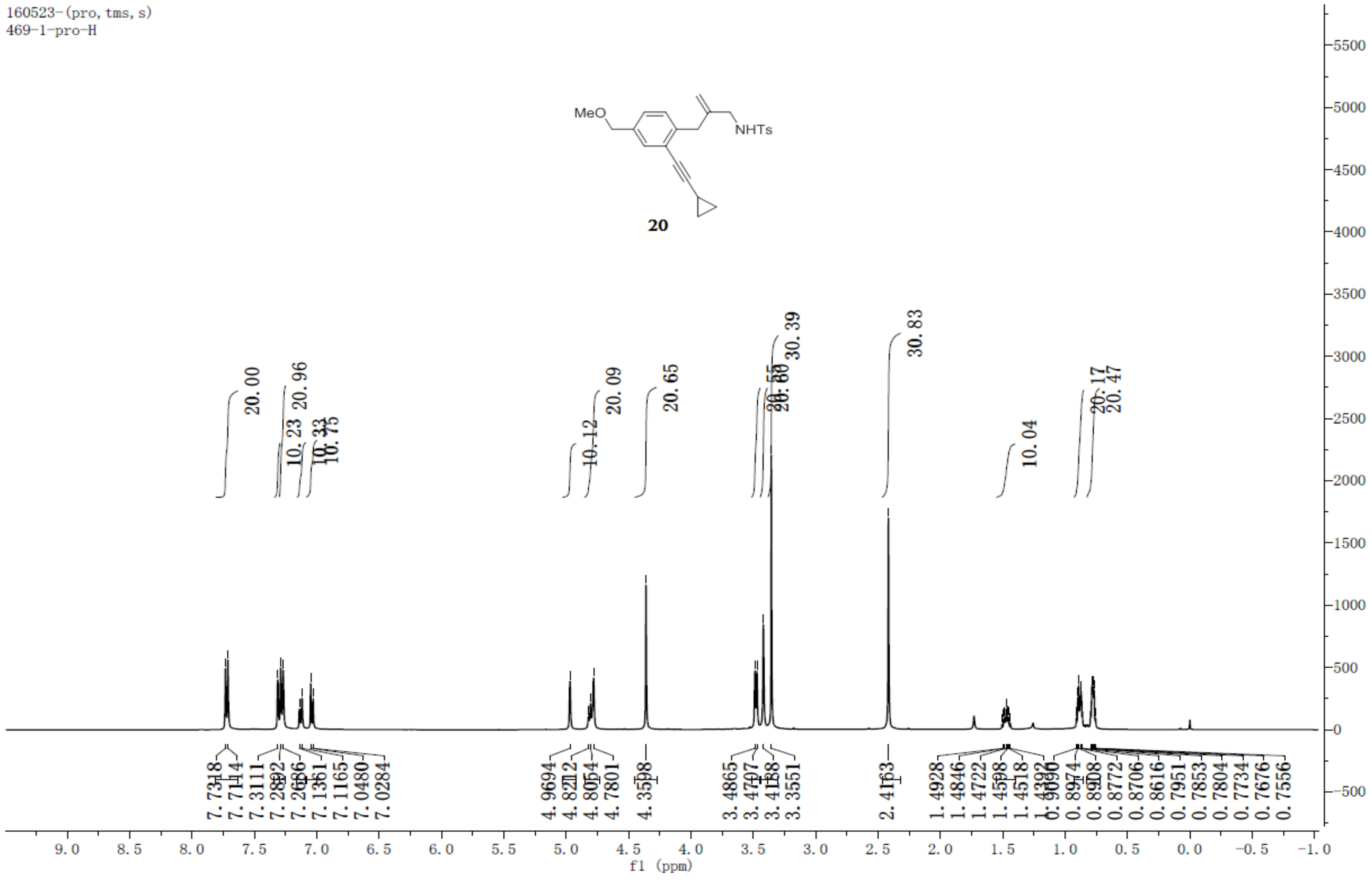

Supplementary Figure 39. ^{1}H NMR Spectrum of substrate 20

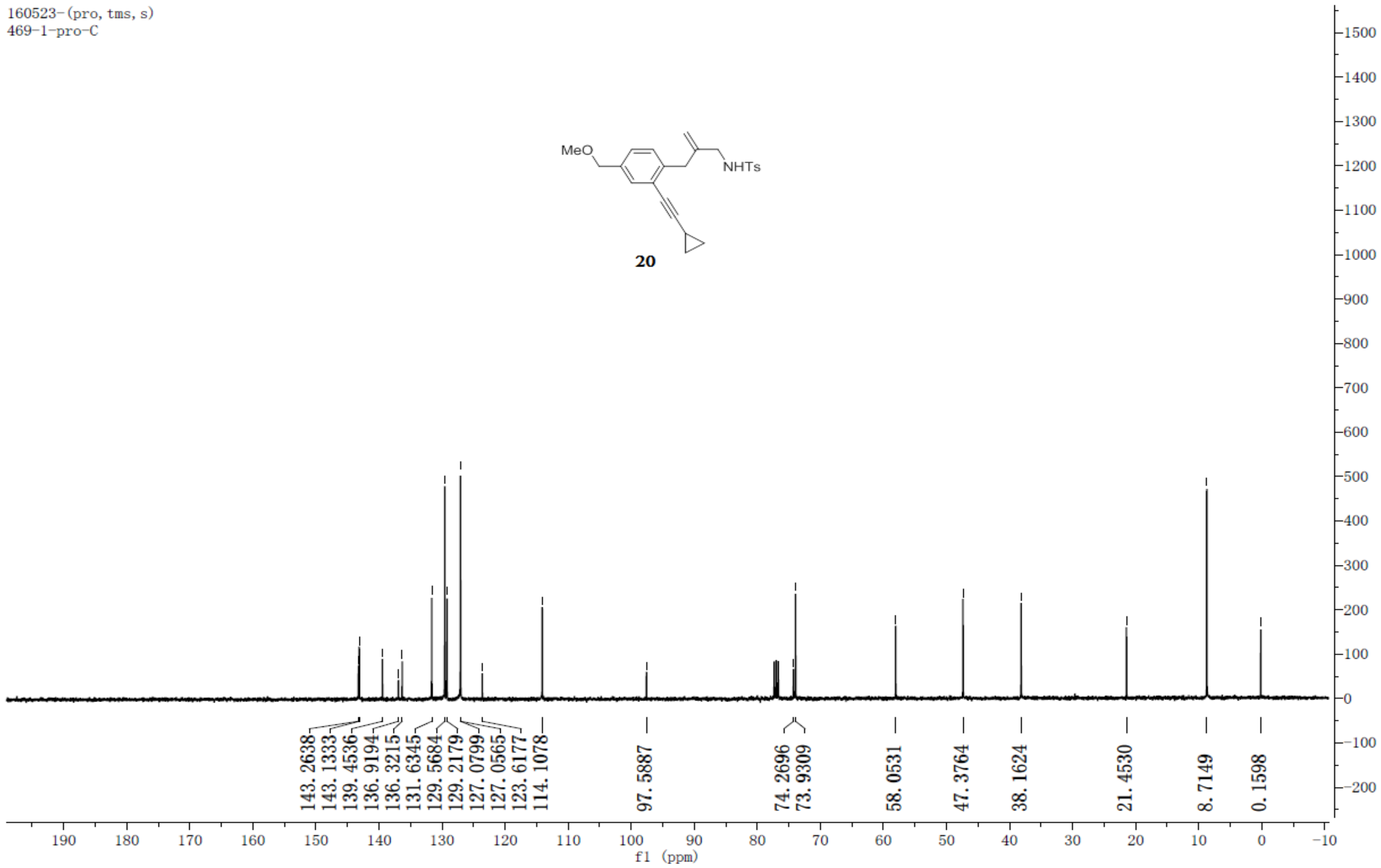

Supplementary Figure 40. ^{13}C NMR Spectrum of substrate 20

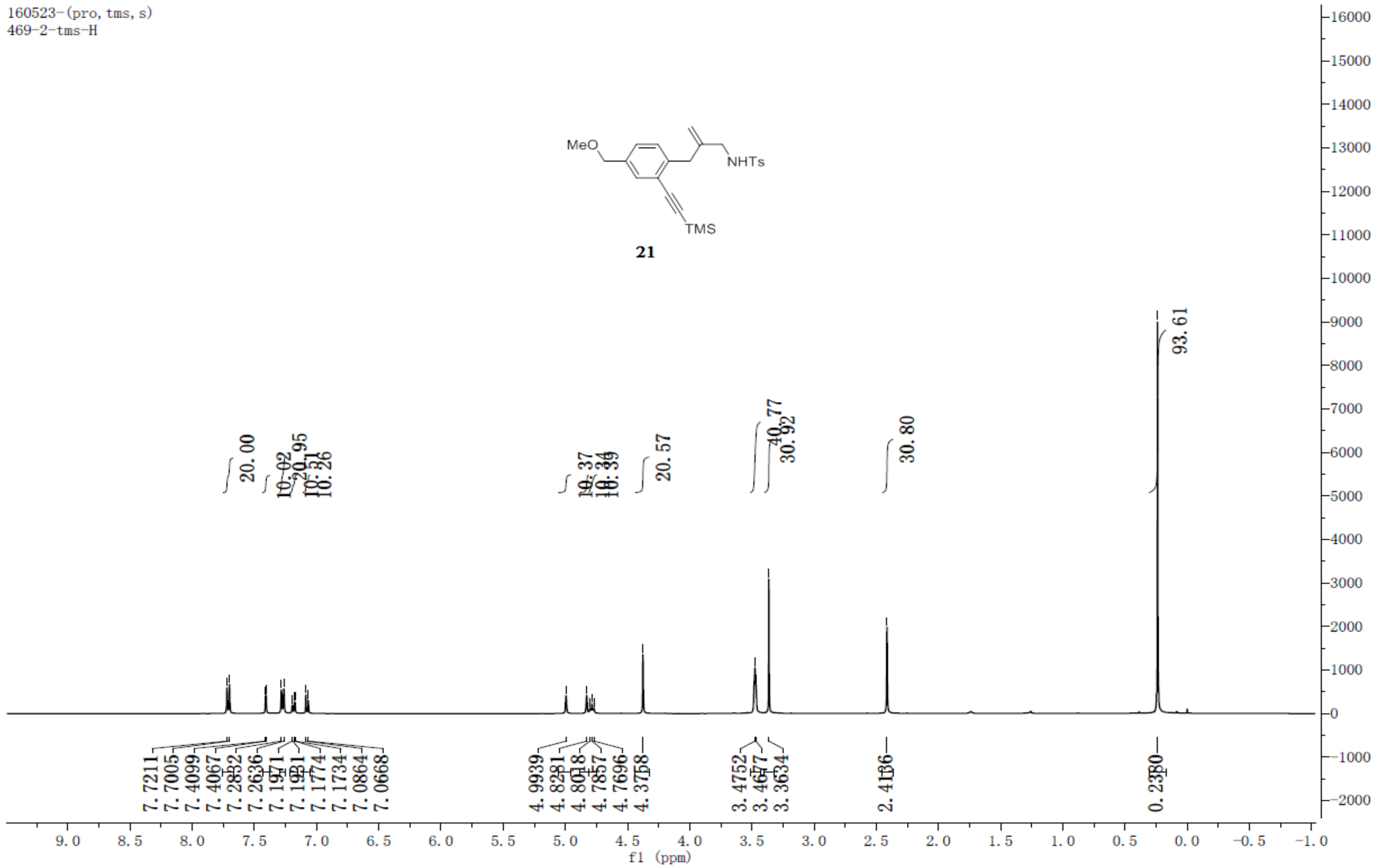

Supplementary Figure 41. ^{1}H NMR Spectrum of substrate 21

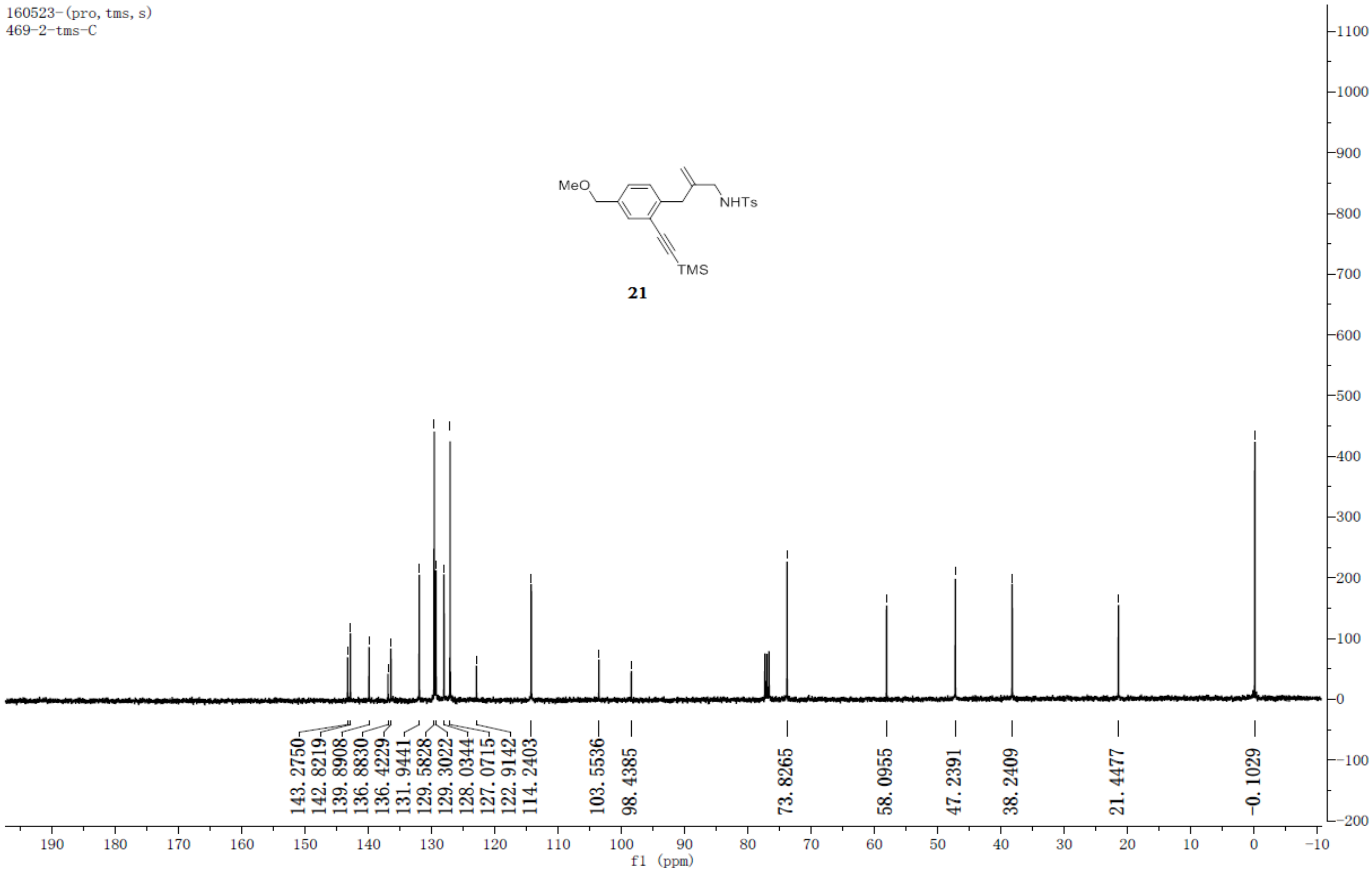

Supplementary Figure 42. ^{13}C NMR Spectrum of substrate 21

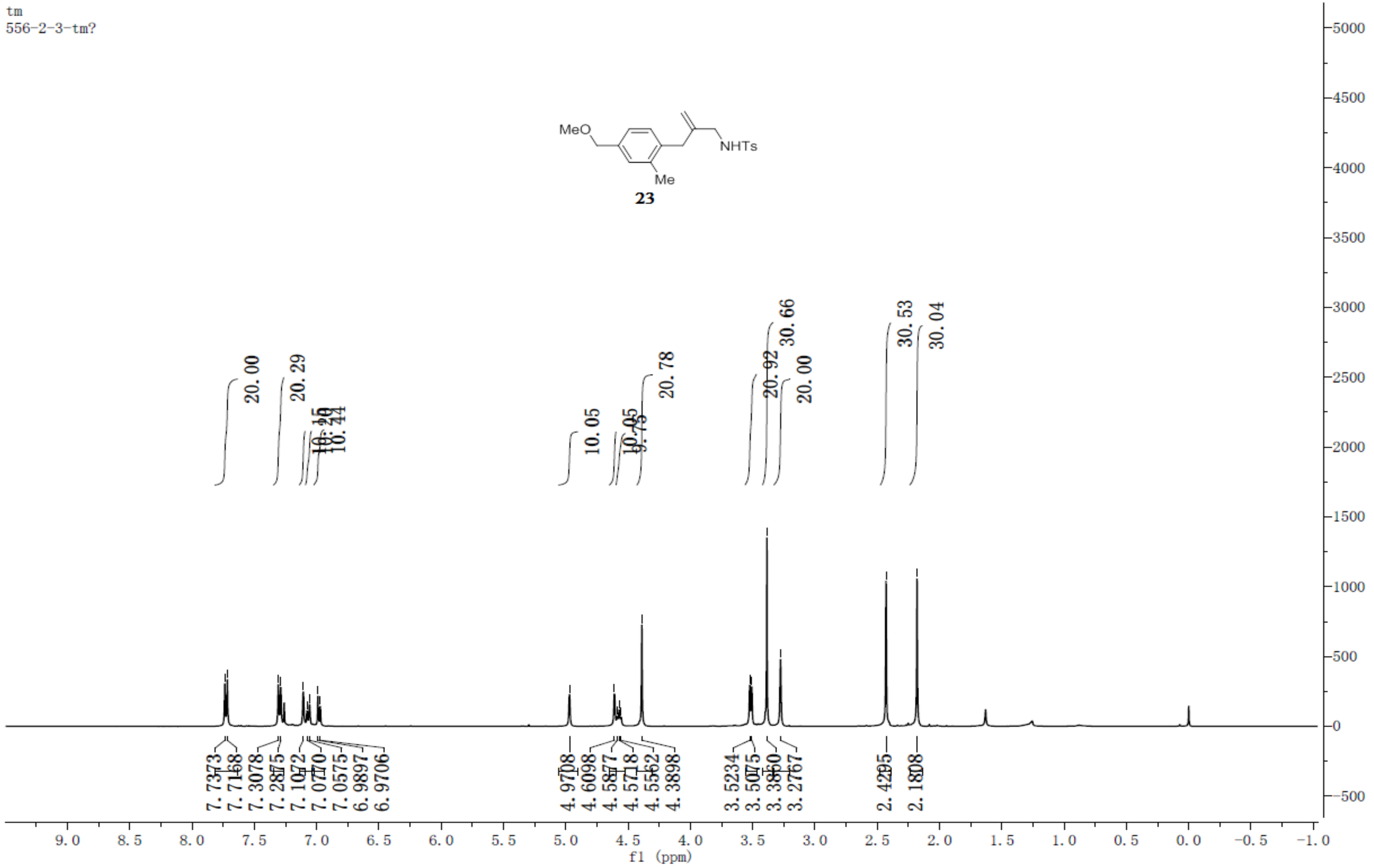

Supplementary Figure 43. ^{1}H NMR Spectrum of substrate 23

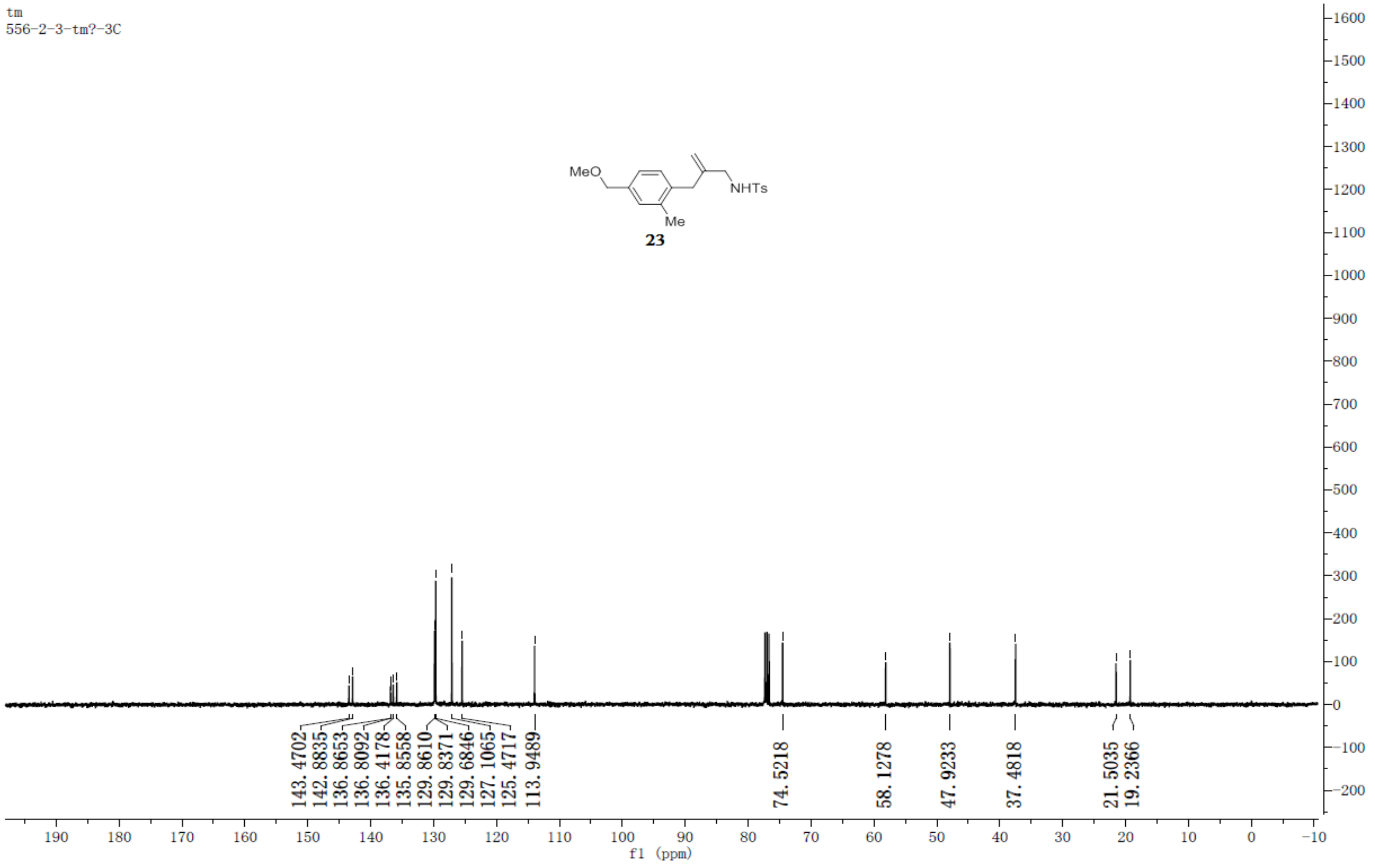

Supplementary Figure 44. ^{13}C NMR Spectrum of substrate 23

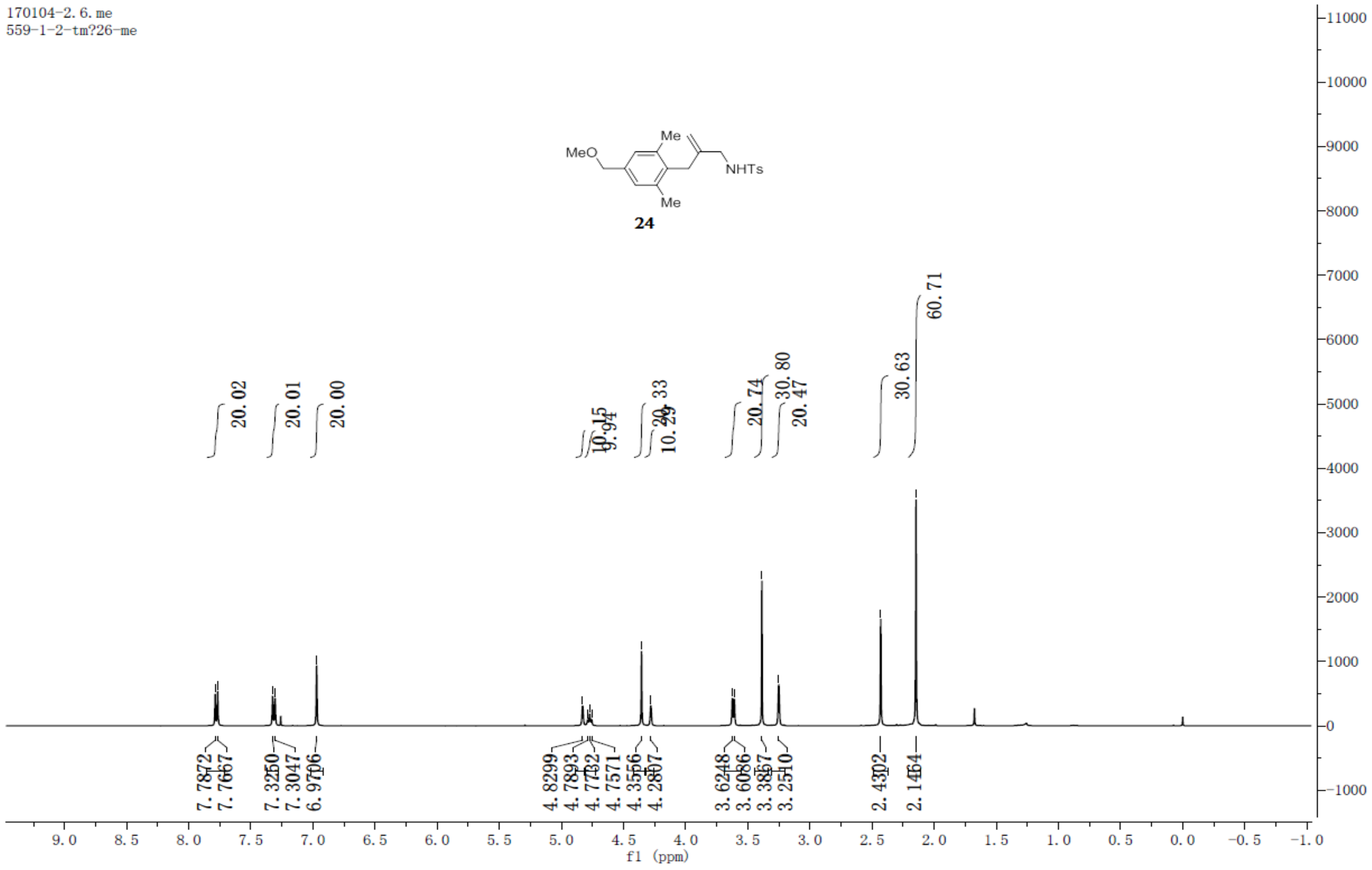

Supplementary Figure 45. ^{1}H NMR Spectrum of substrate 24

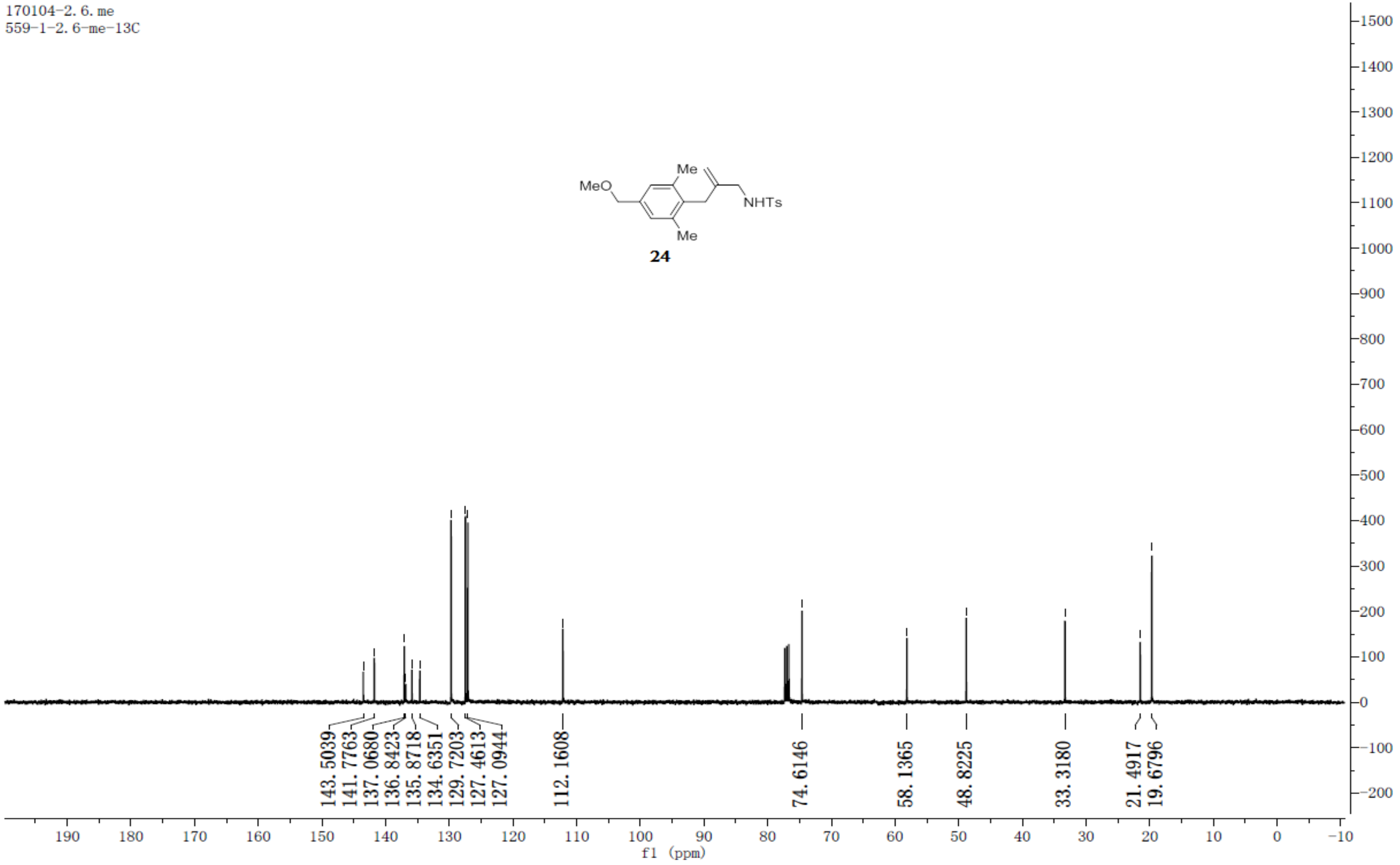

Supplementary Figure 46. ^{13}C NMR Spectrum of substrate 24

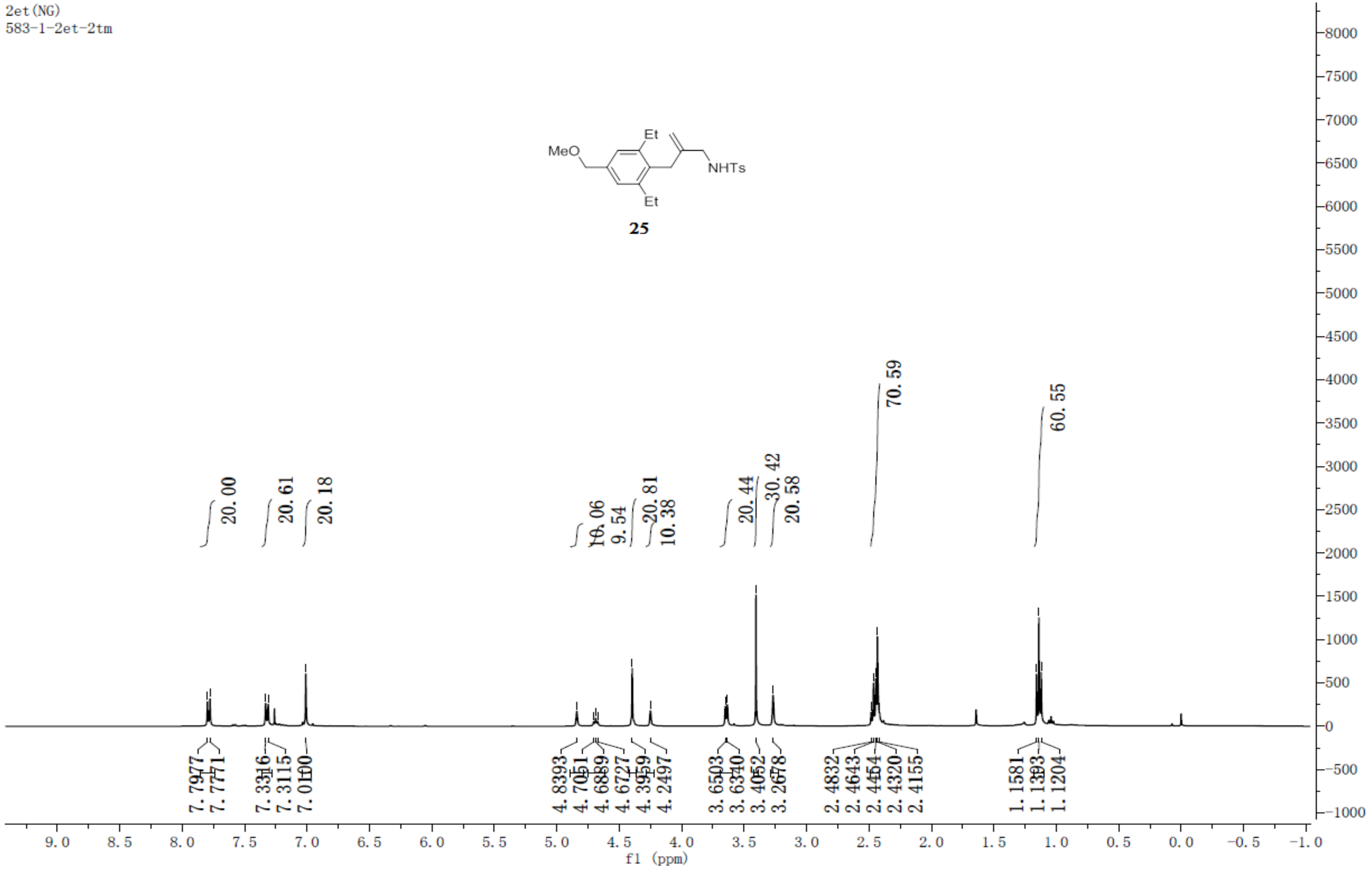

Supplementary Figure 47. 1H NMR Spectrum of substrate 25

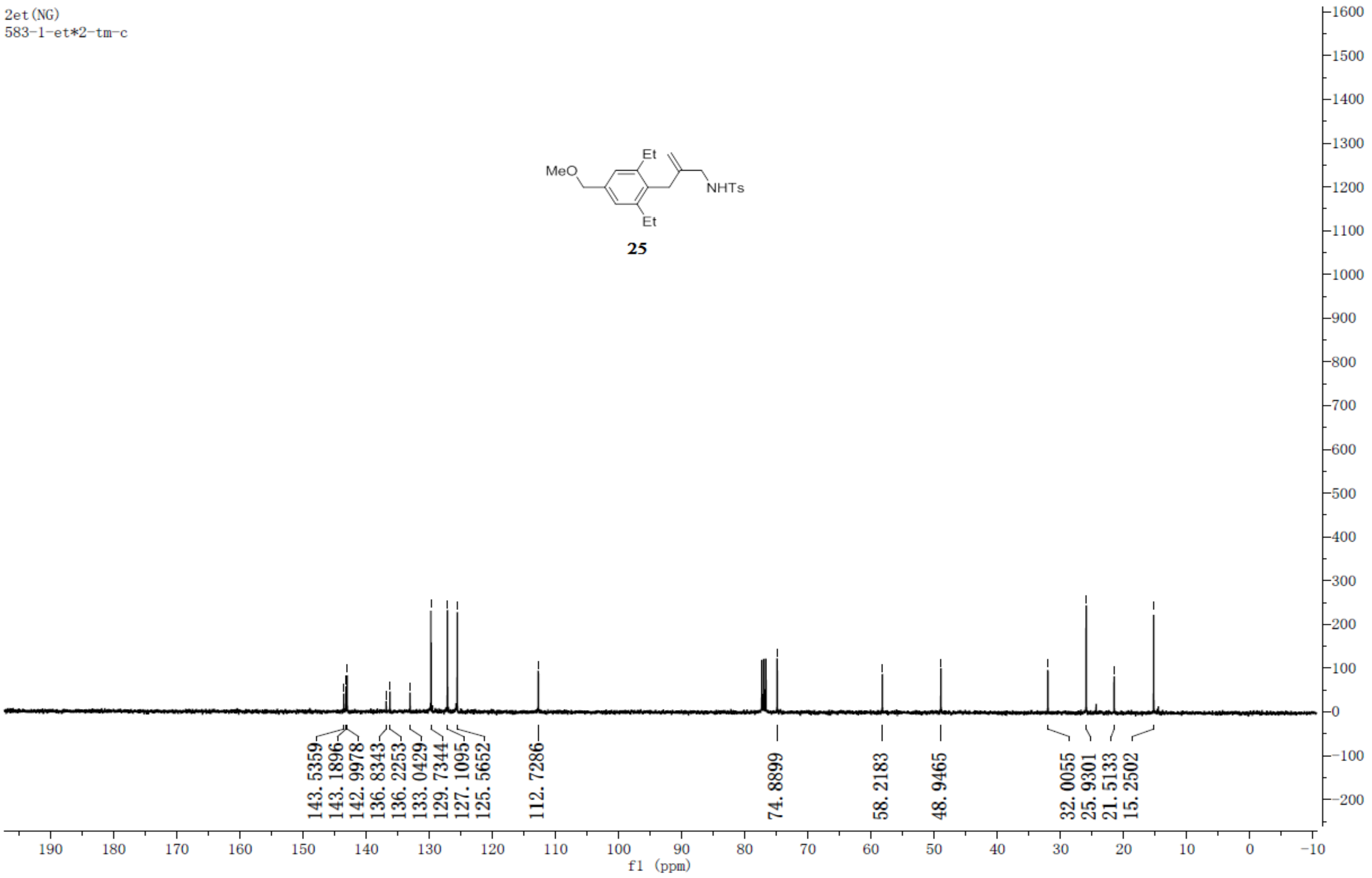

Supplementary Figure 48. ^{13}C NMR Spectrum of substrate 25

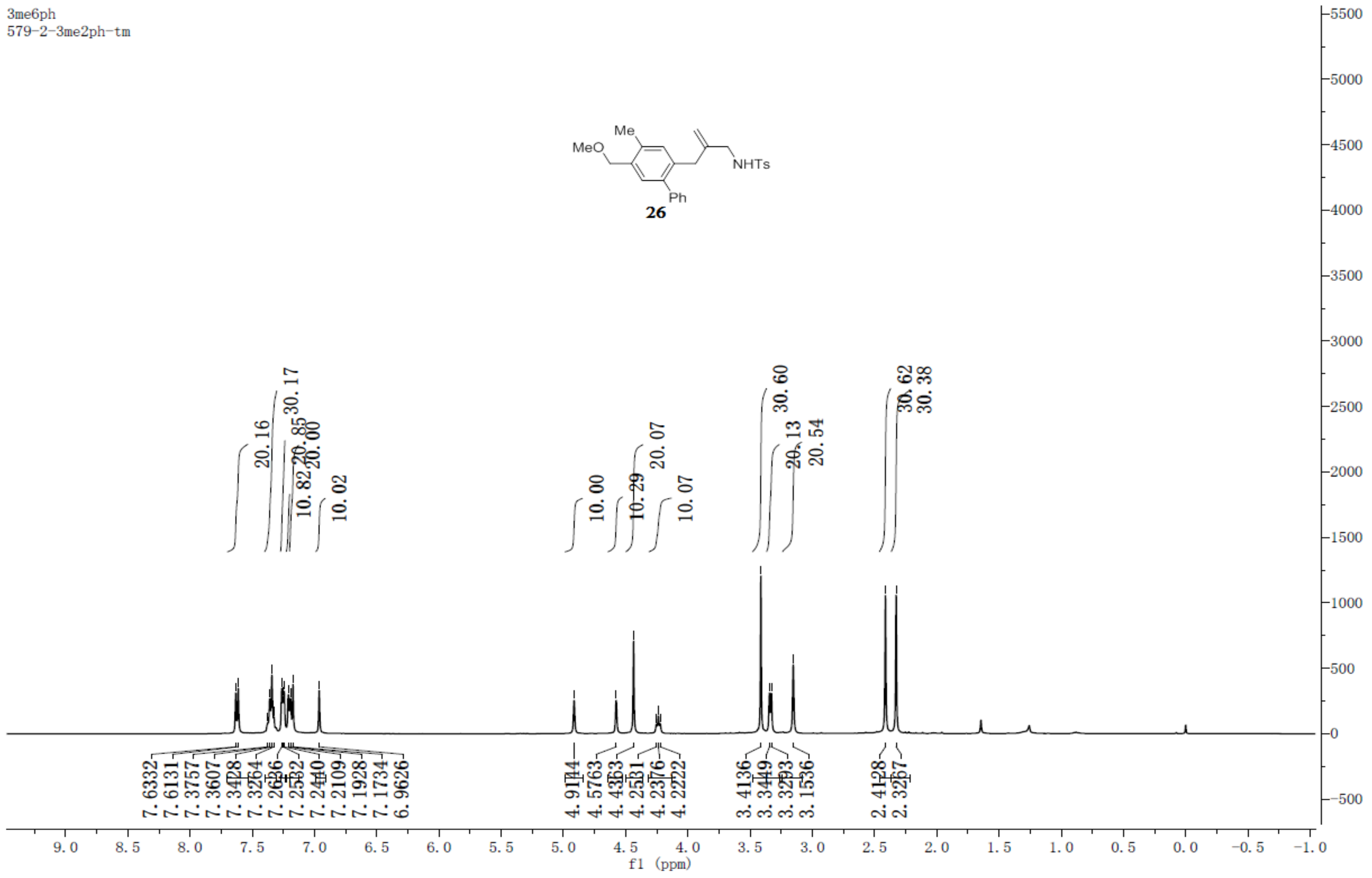

Supplementary Figure 49. ^{1}H NMR Spectrum of substrate 26

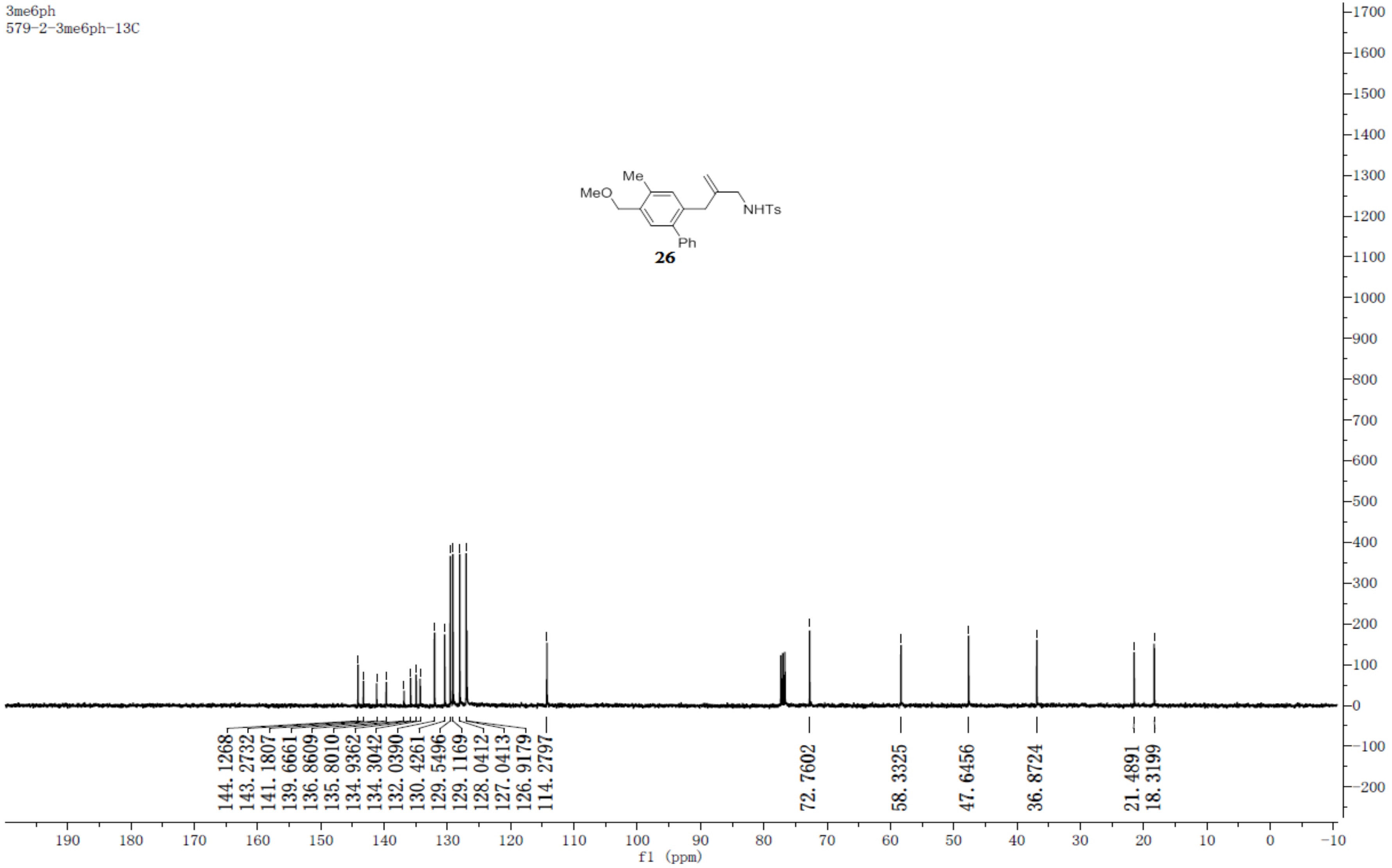

Supplementary Figure 50. ^{13}C NMR Spectrum of substrate 26

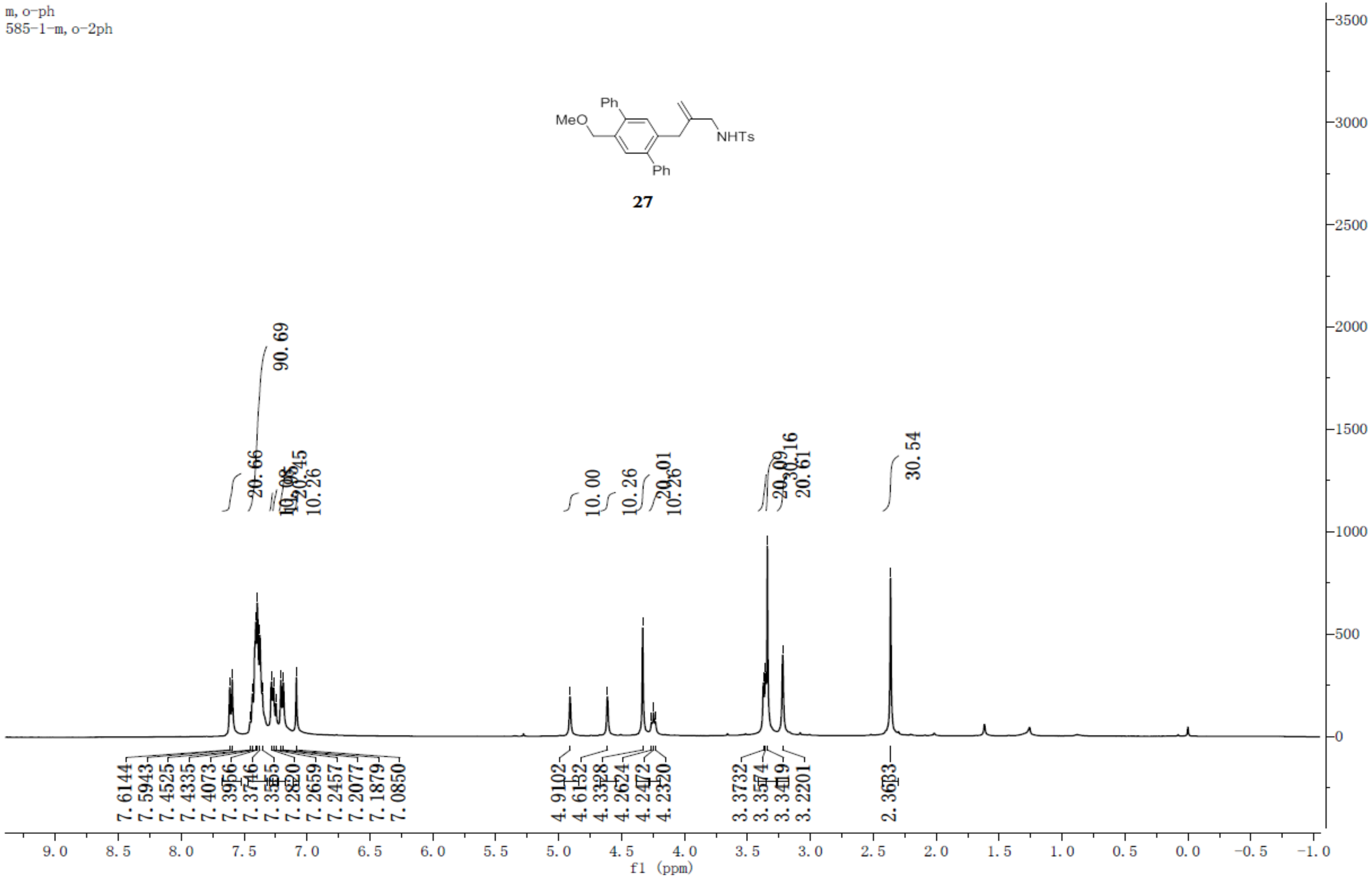

Supplementary Figure 51. ^{1}H NMR Spectrum of substrate 27

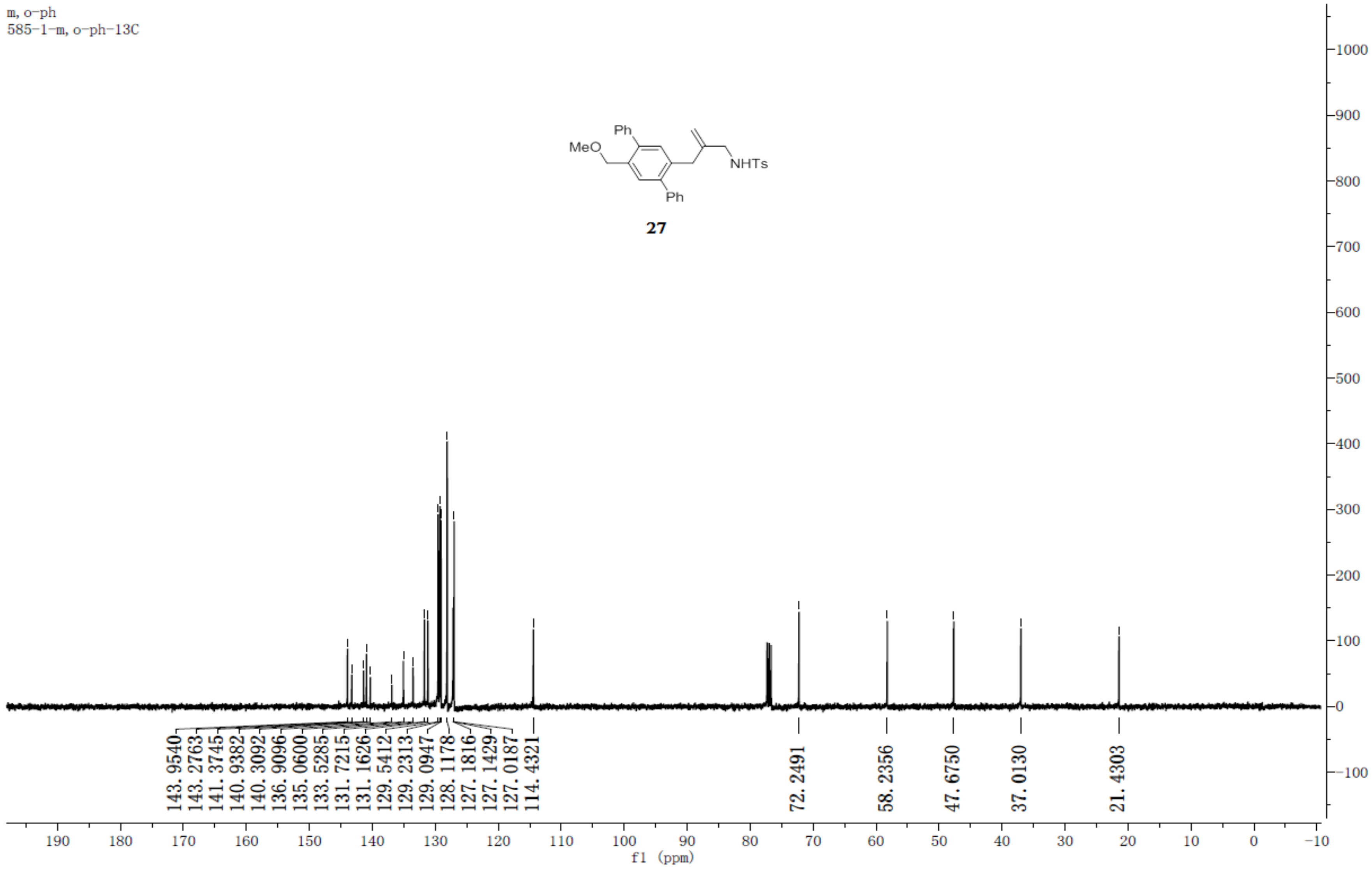

Supplementary Figure 52. ¹³C NMR Spectrum of substrate 27

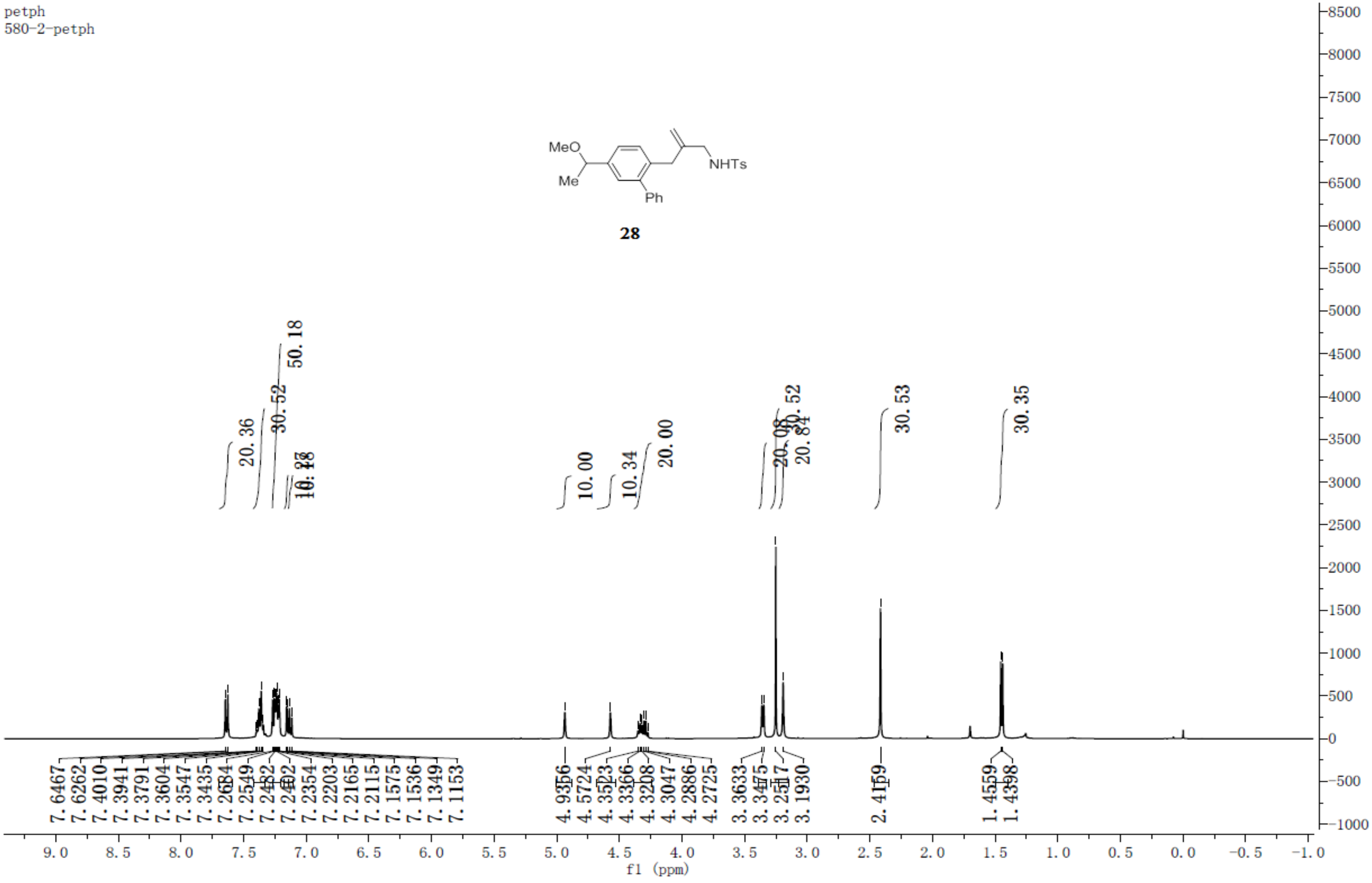

Supplementary Figure 53. ^{1}H NMR Spectrum of substrate 28

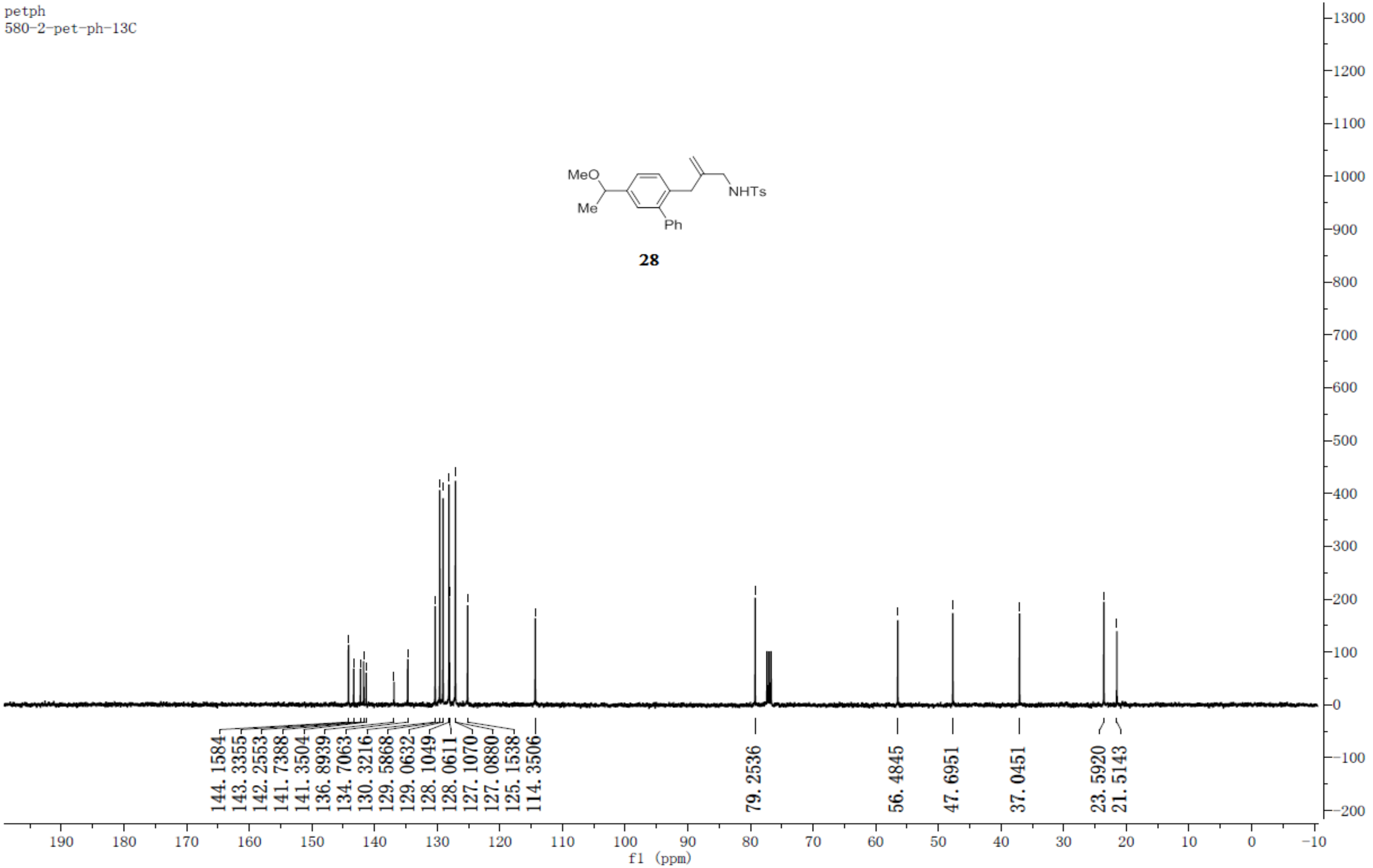

Supplementary Figure 54. ^{13}C NMR Spectrum of substrate 28

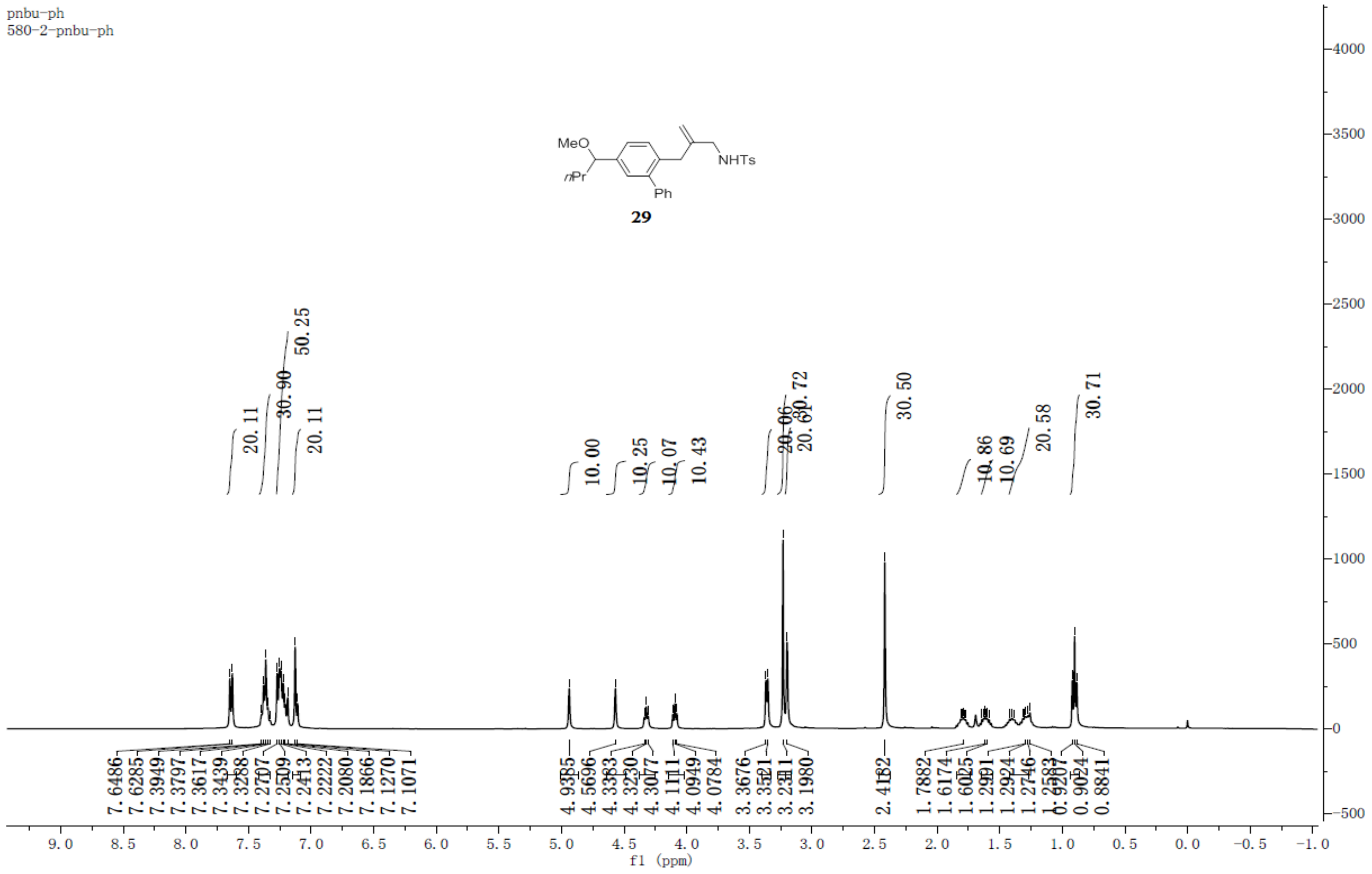

Supplementary Figure 55. ^{1}H NMR Spectrum of substrate 29

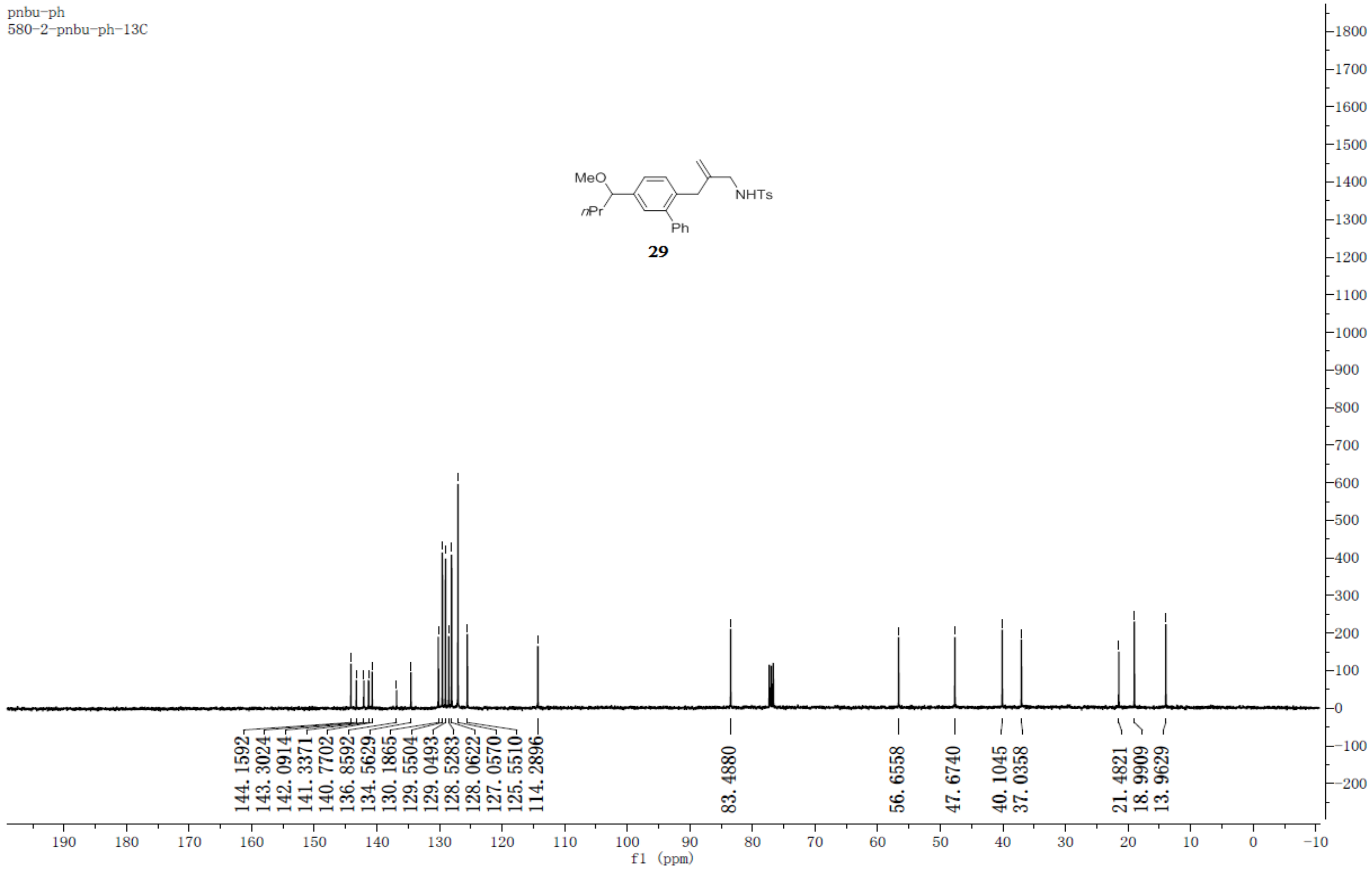

Supplementary Figure 56. ^{13}C NMR Spectrum of substrate 29

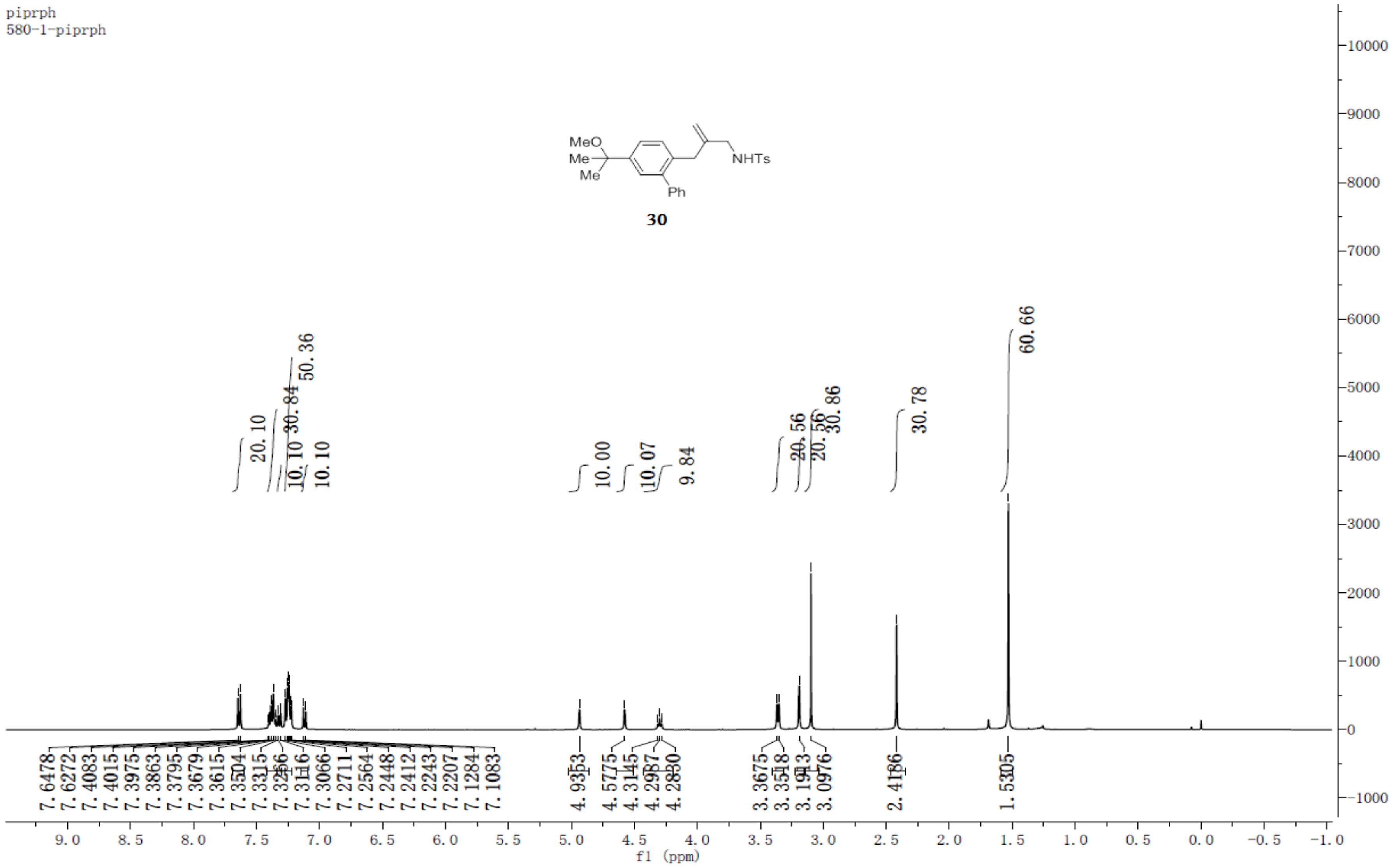

Supplementary Figure 57. ^{1}H NMR Spectrum of substrate 30

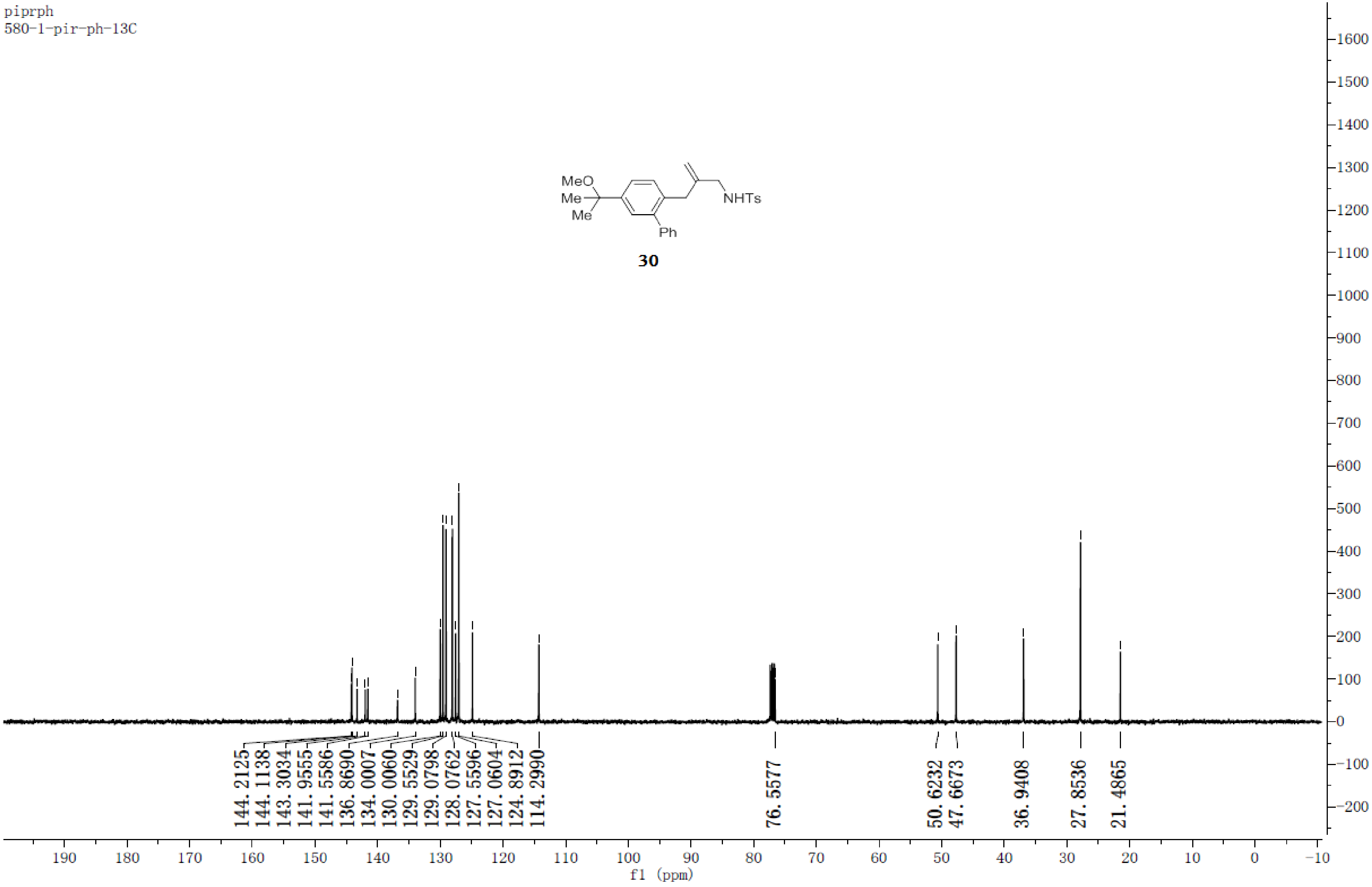

Supplementary Figure 58. ^{13}C NMR Spectrum of substrate 30

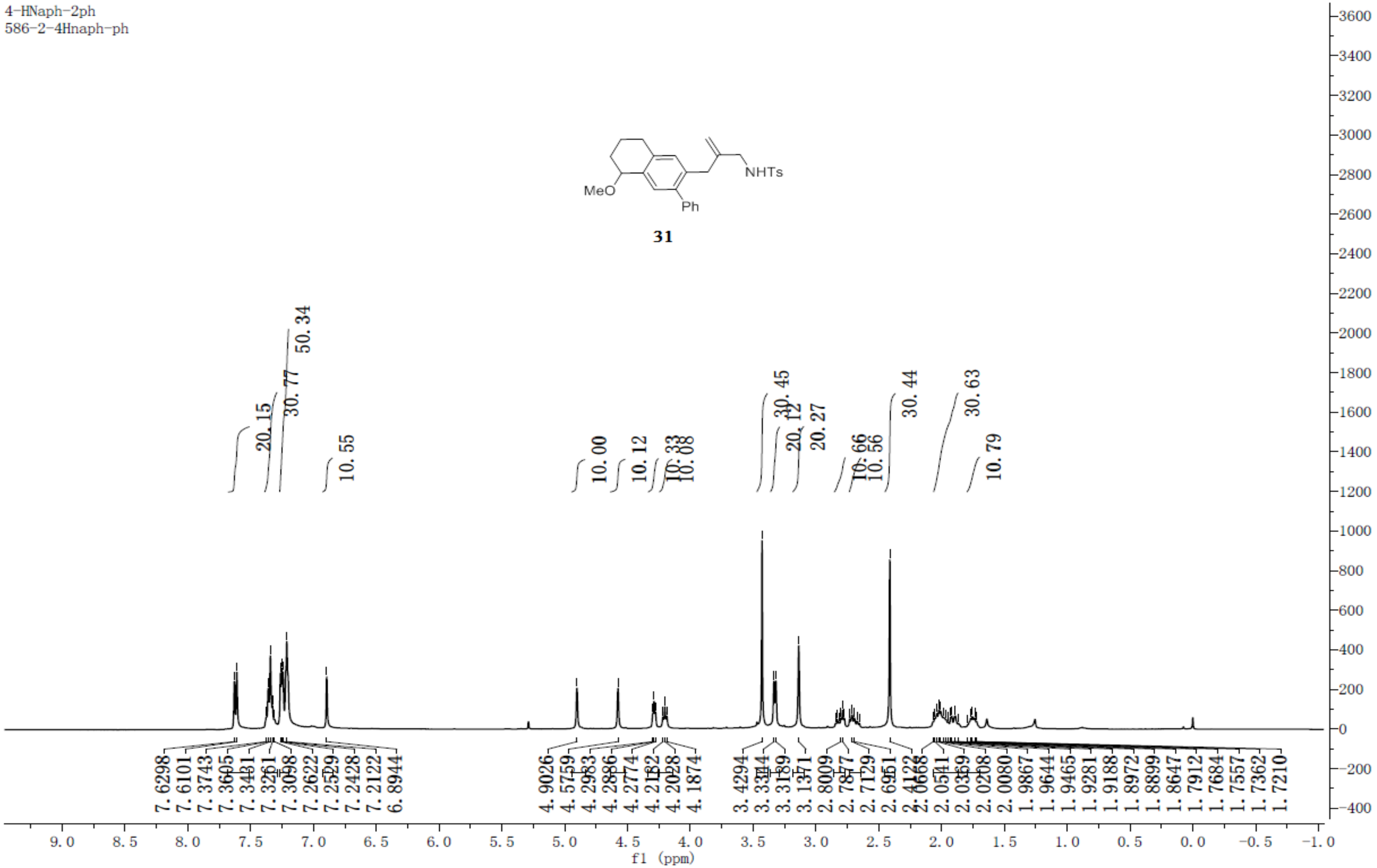

Supplementary Figure 59. ^{1}H NMR Spectrum of substrate 31

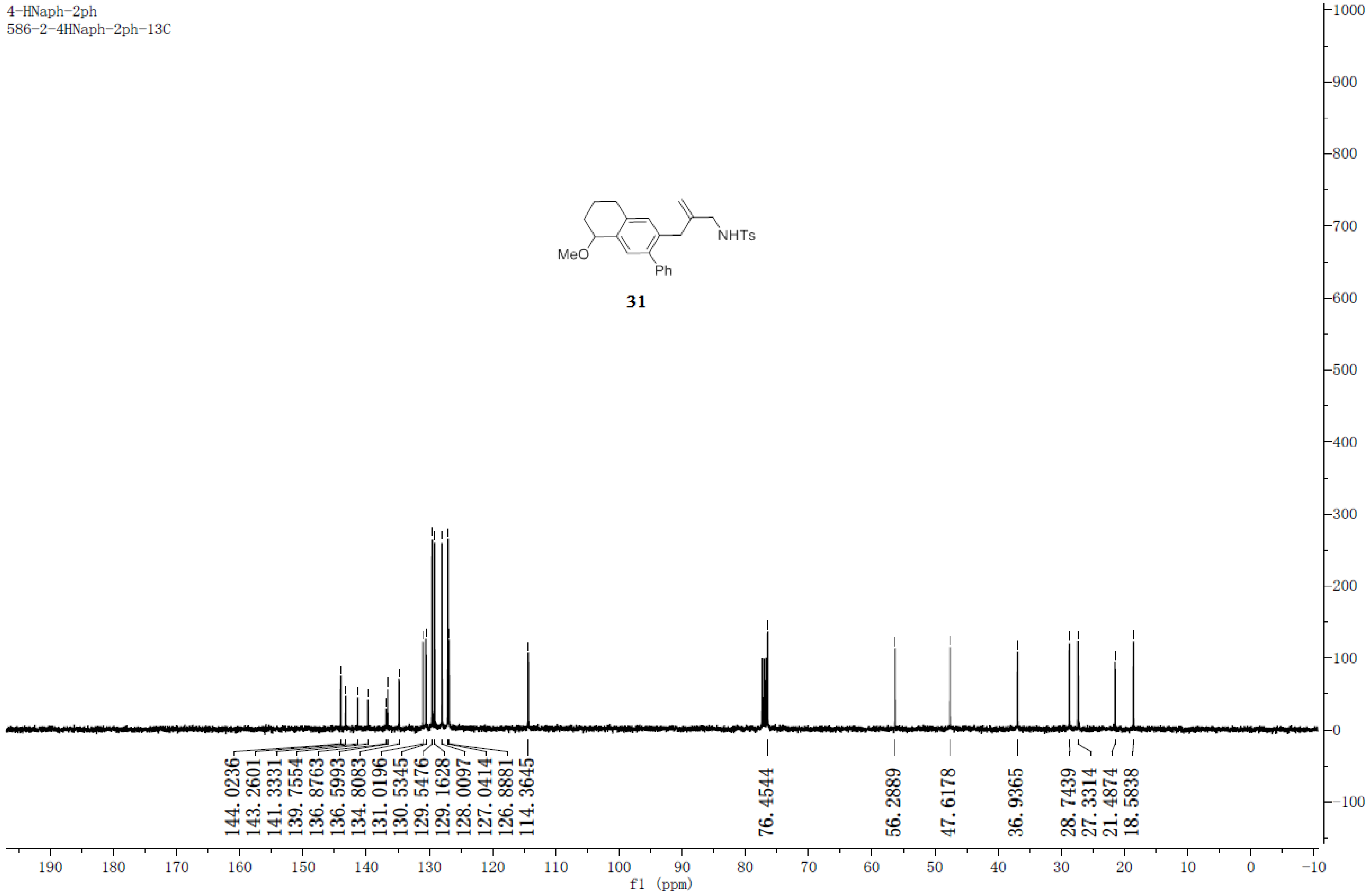

Supplementary Figure 60. ^{13}C NMR Spectrum of substrate 31

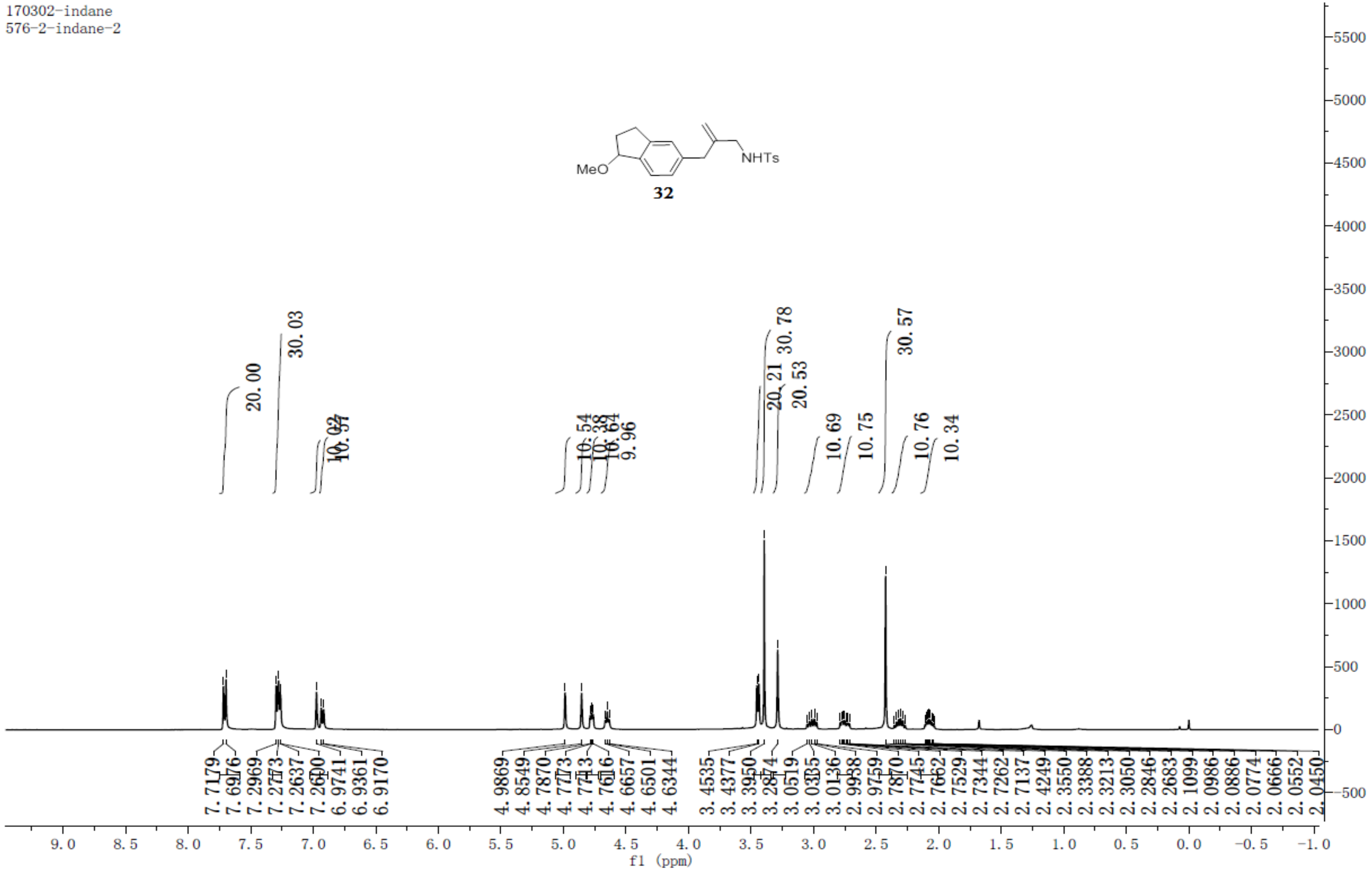

Supplementary Figure 61. ^{1}H NMR Spectrum of substrate 32

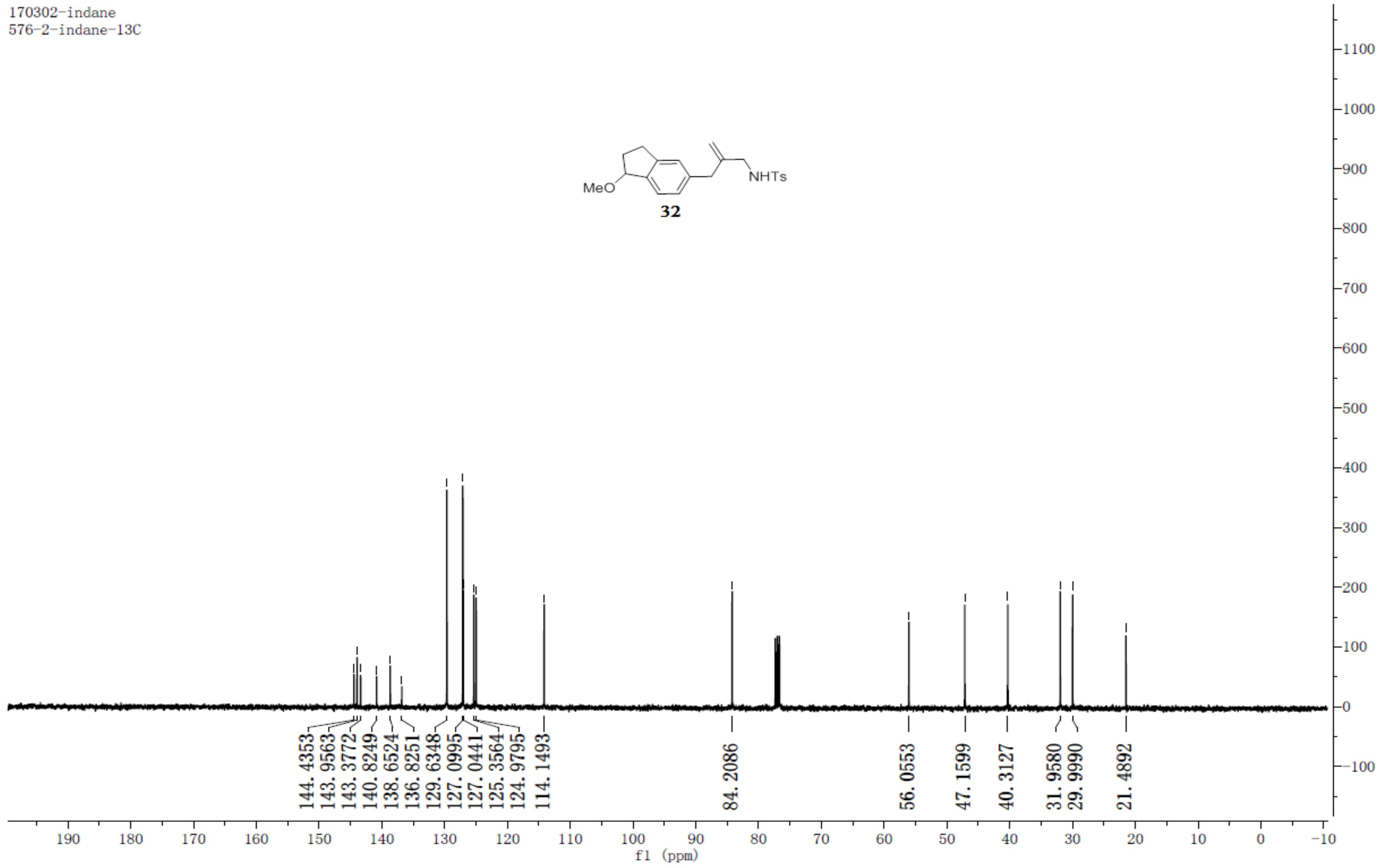

Supplementary Figure 62. ^{13}C NMR Spectrum of substrate 32

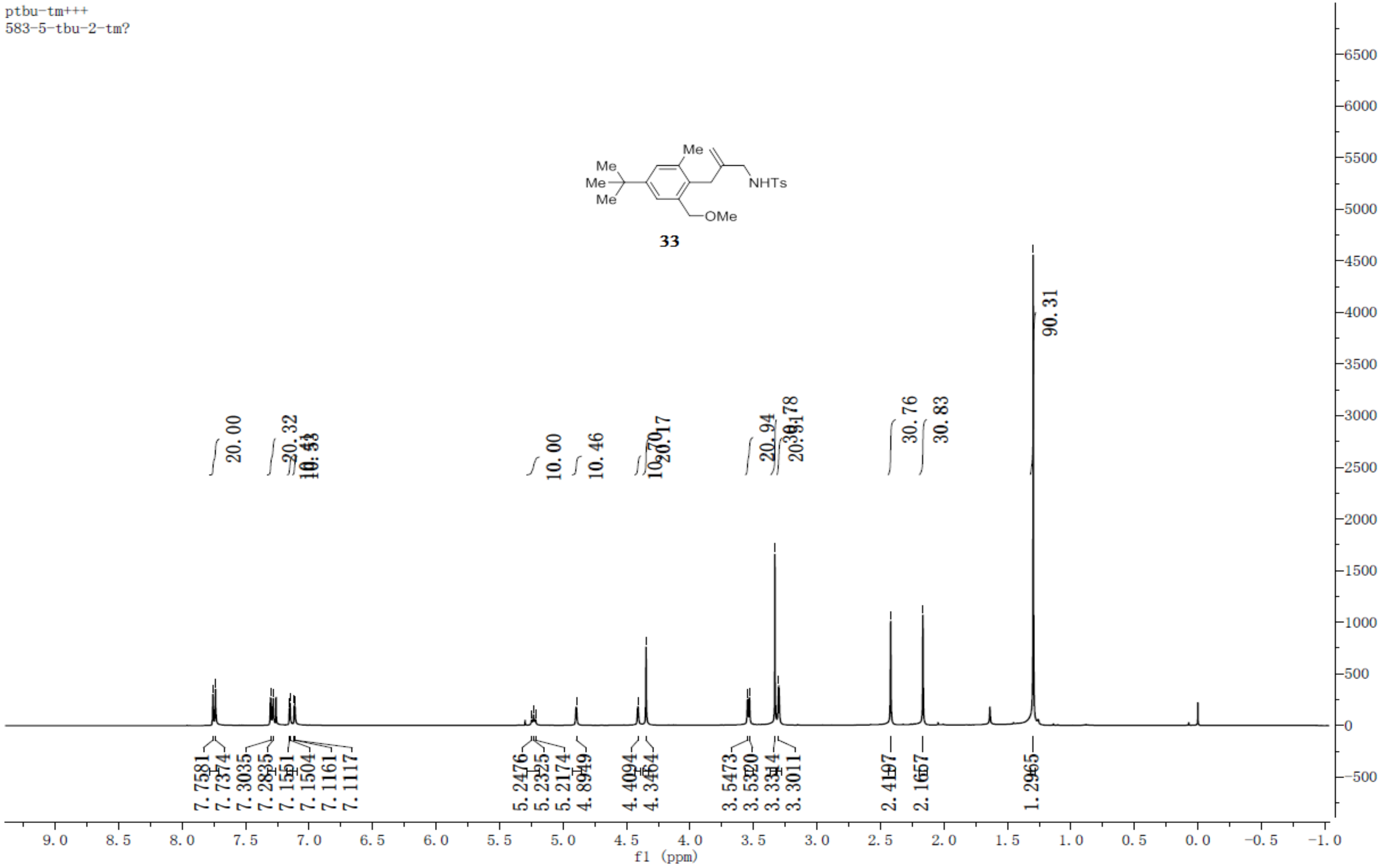

Supplementary Figure 63. ^{1}H NMR Spectrum of substrate 33

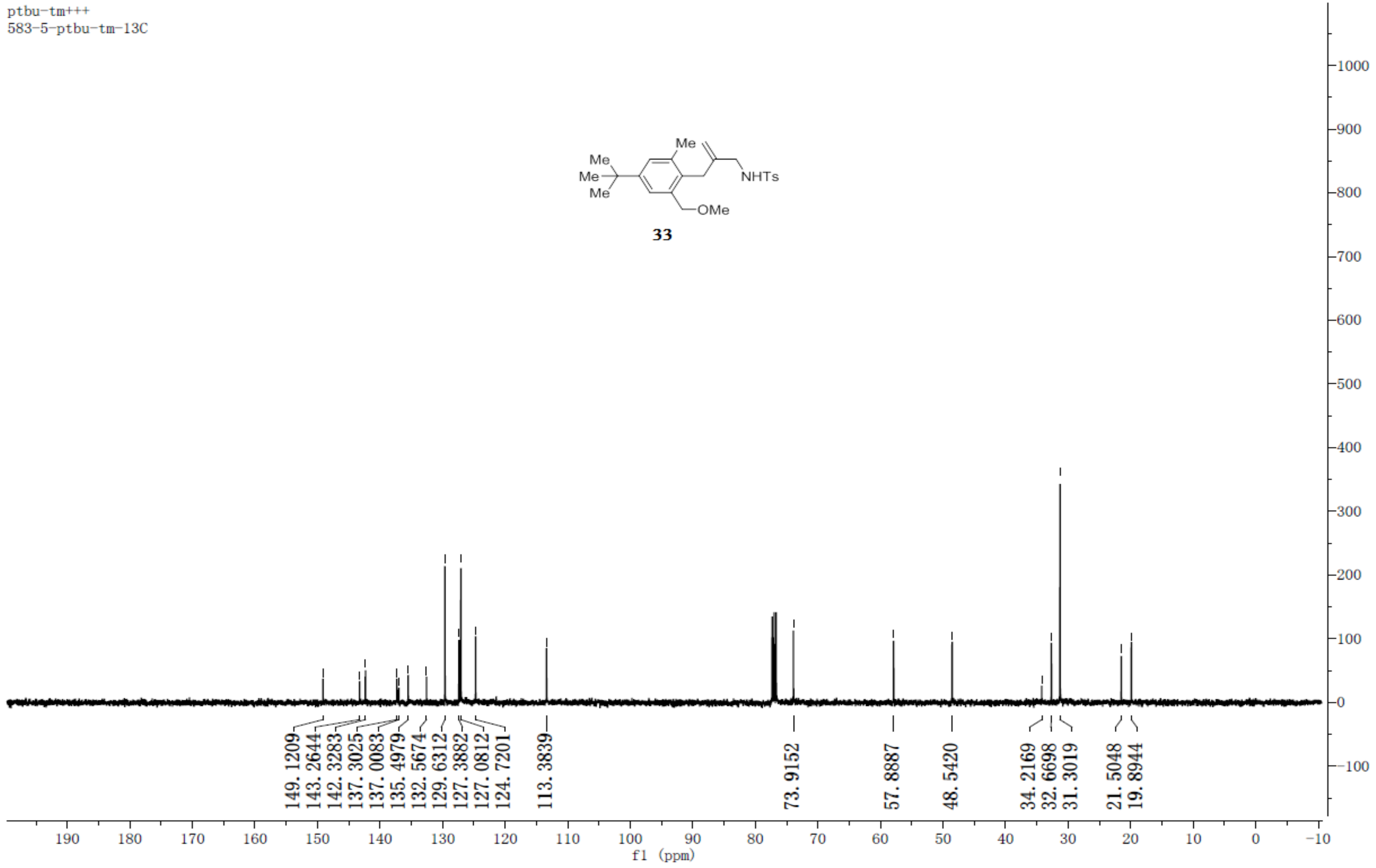

Supplementary Figure 64. ^{13}C NMR Spectrum of substrate 33

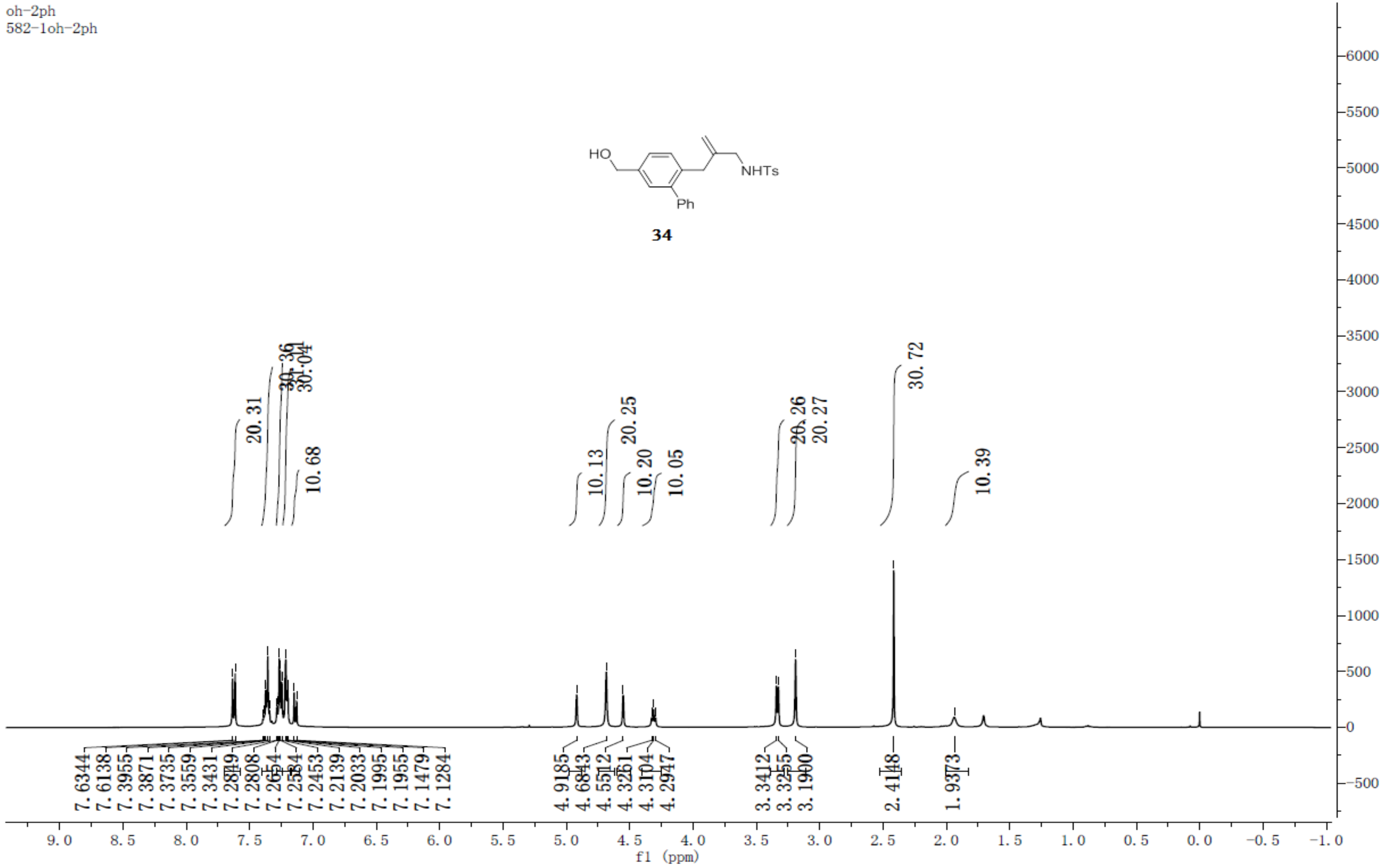

Supplementary Figure 65. ¹H NMR Spectrum of substrate 34

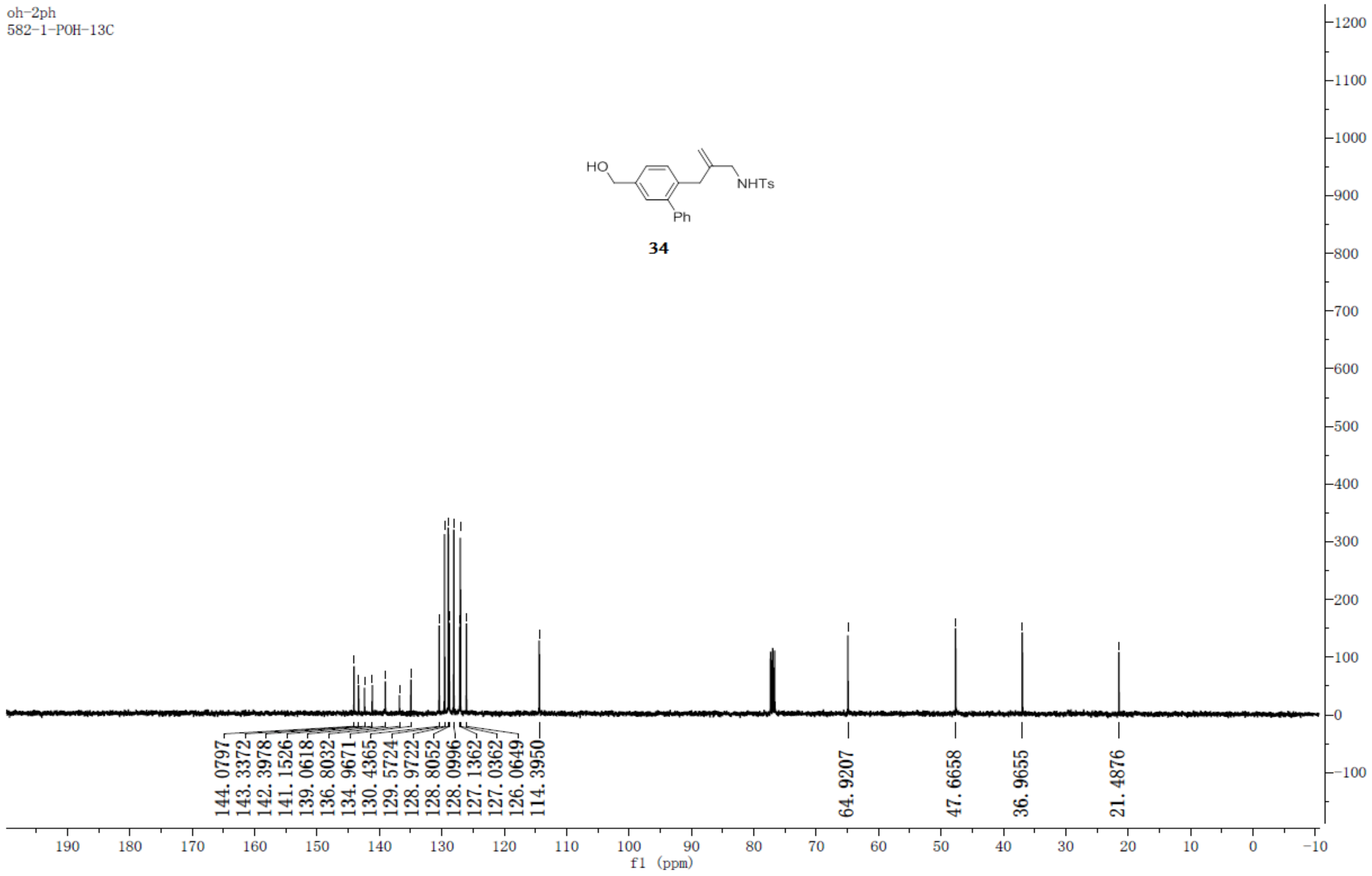

Supplementary Figure 66. 13C NMR Spectrum of substrate 34

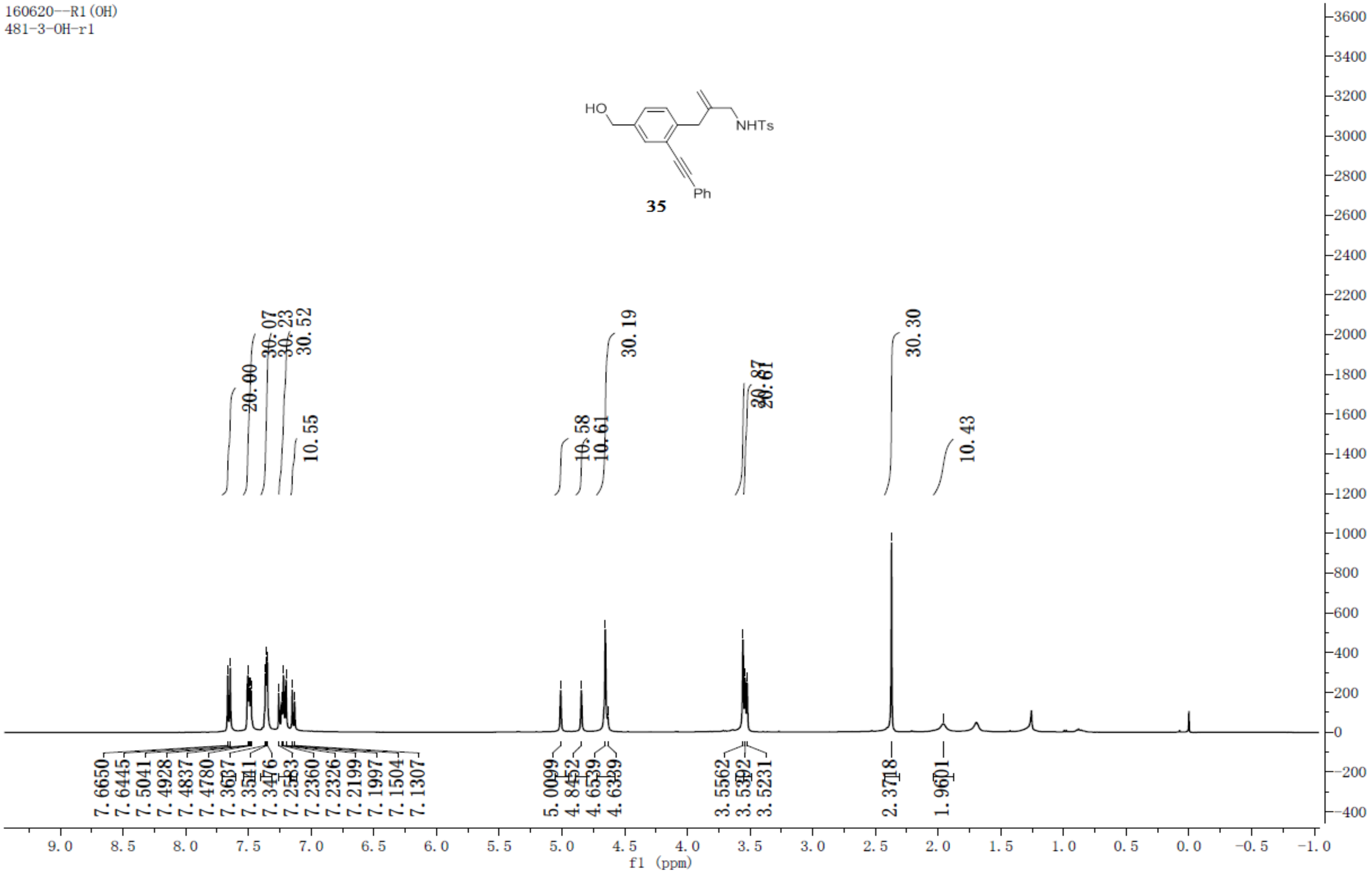

Supplementary Figure 67. ^{1}H NMR Spectrum of substrate 35

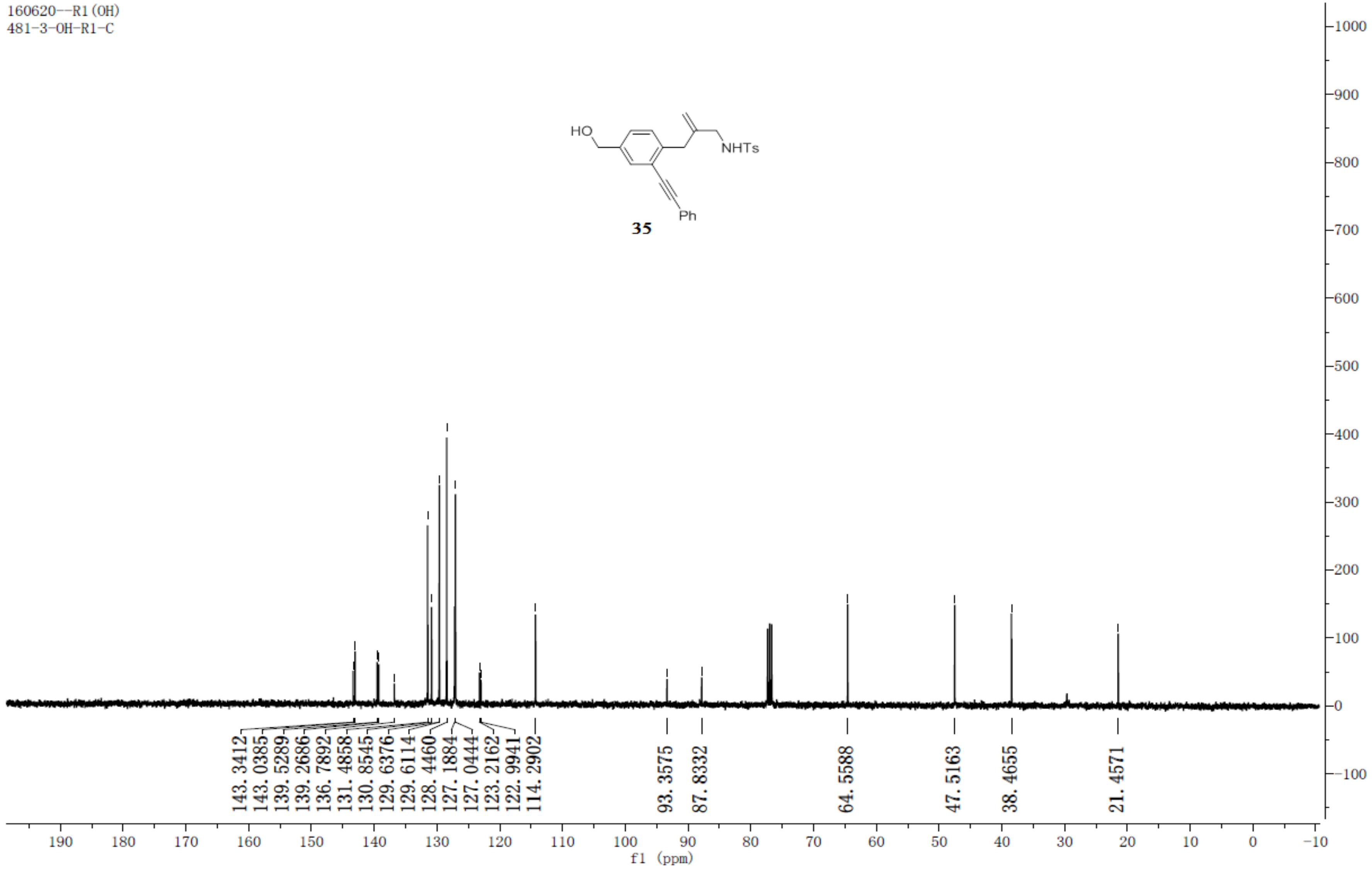

Supplementary Figure 68. ^{13}C NMR Spectrum of substrate 35

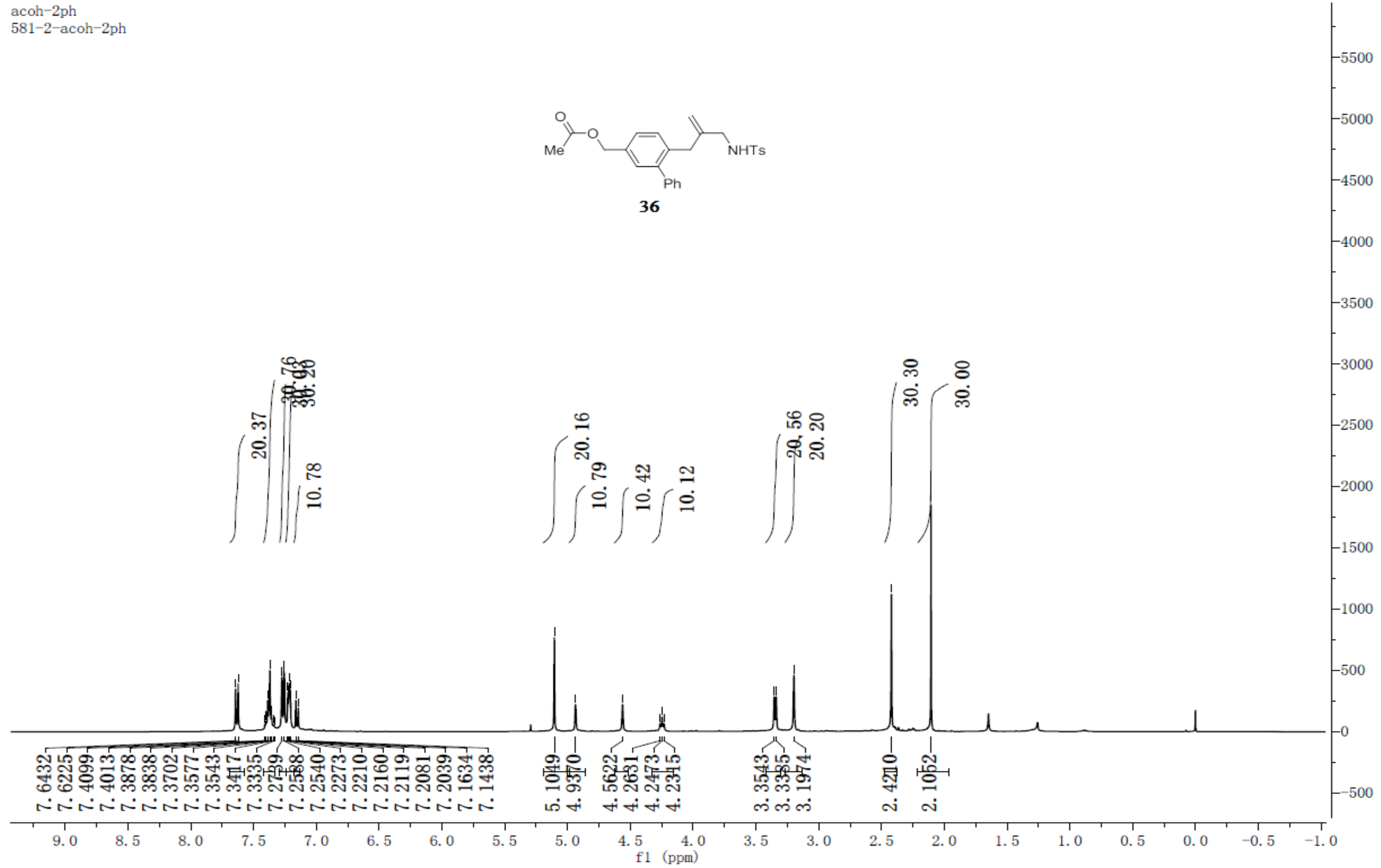

Supplementary Figure 69. ^{1}H NMR Spectrum of substrate 36

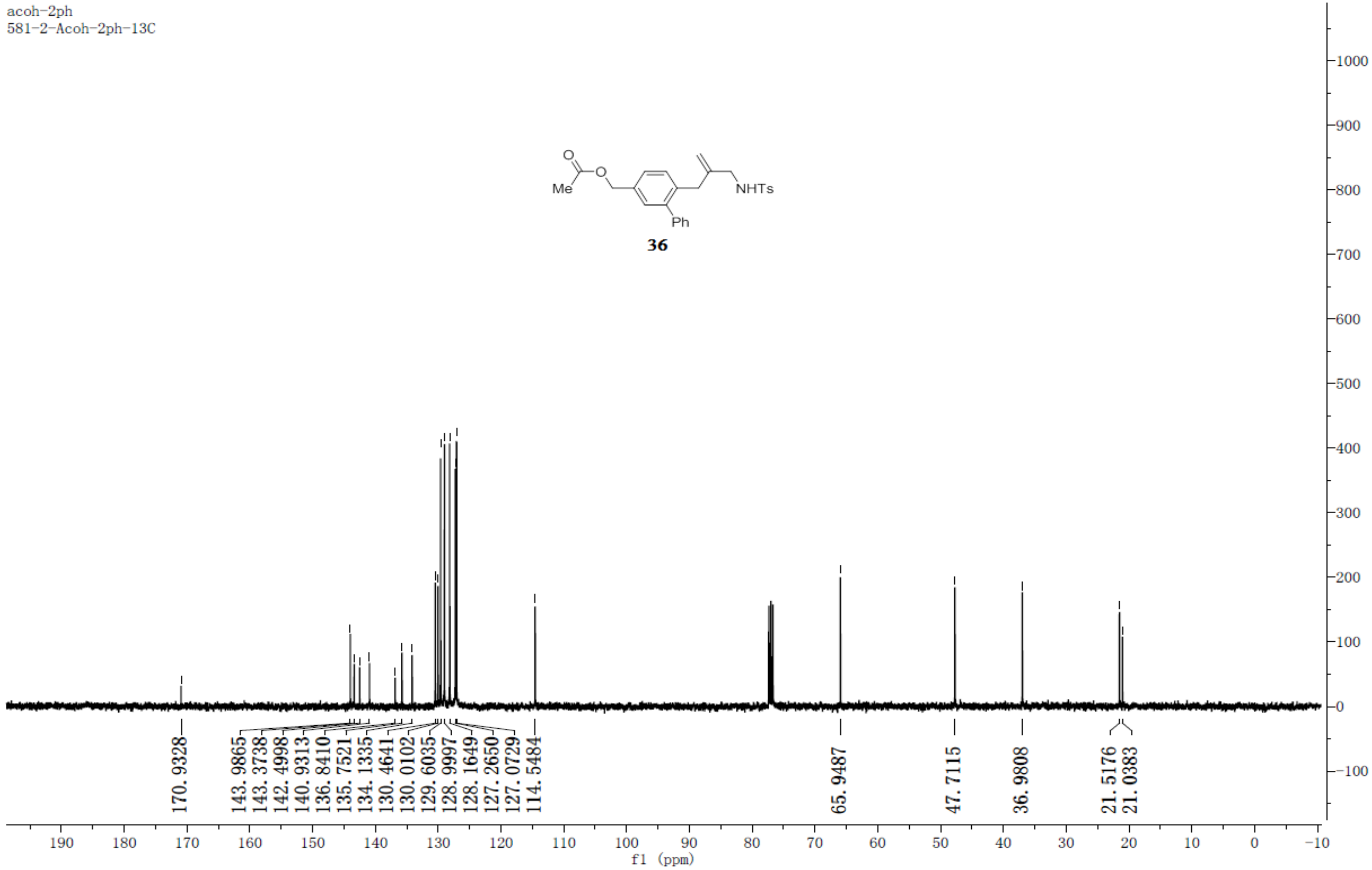

Supplementary Figure 70. ^{13}C NMR Spectrum of substrate 36

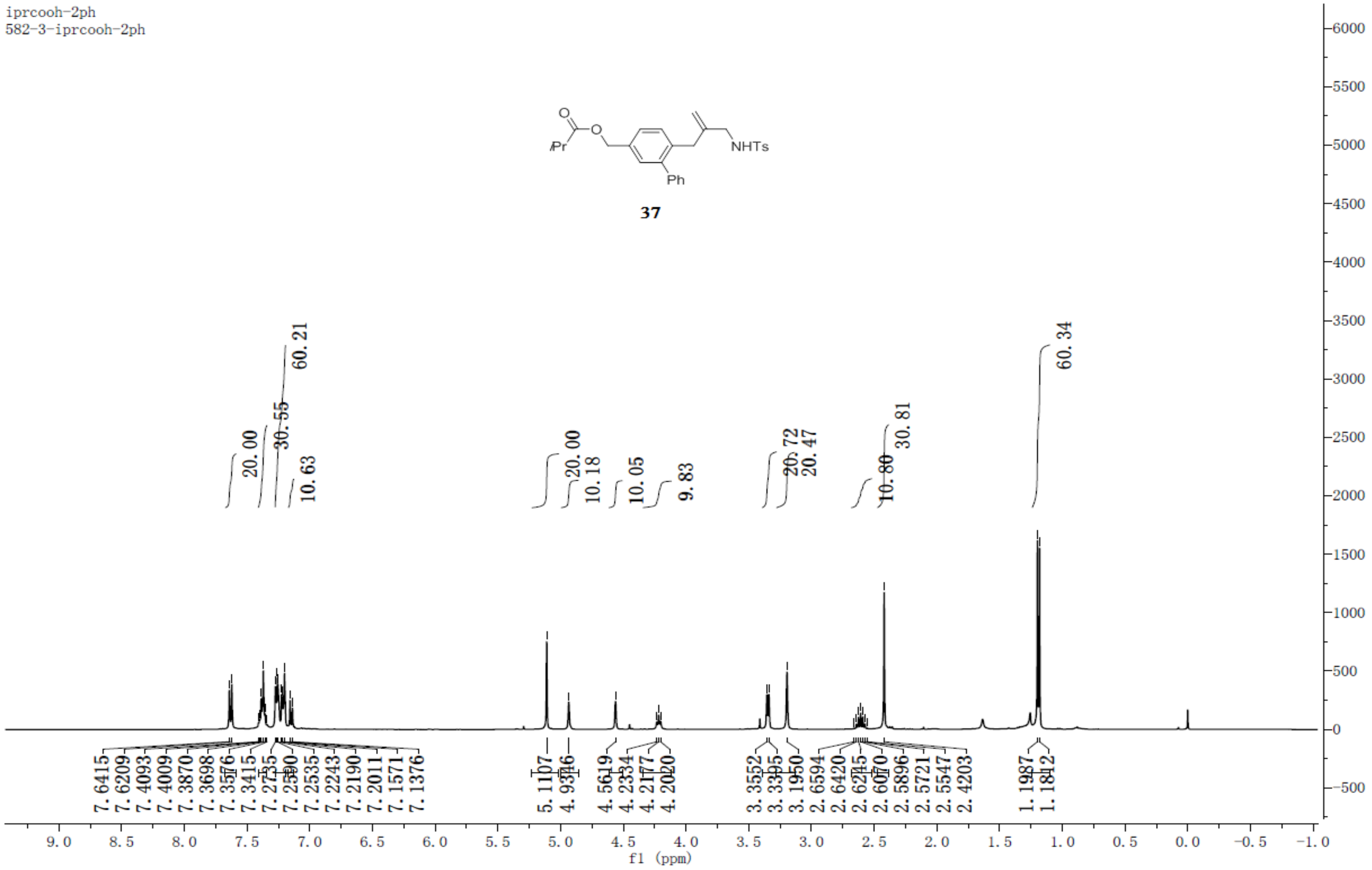

Supplementary Figure 71. ¹H NMR Spectrum of substrate 37

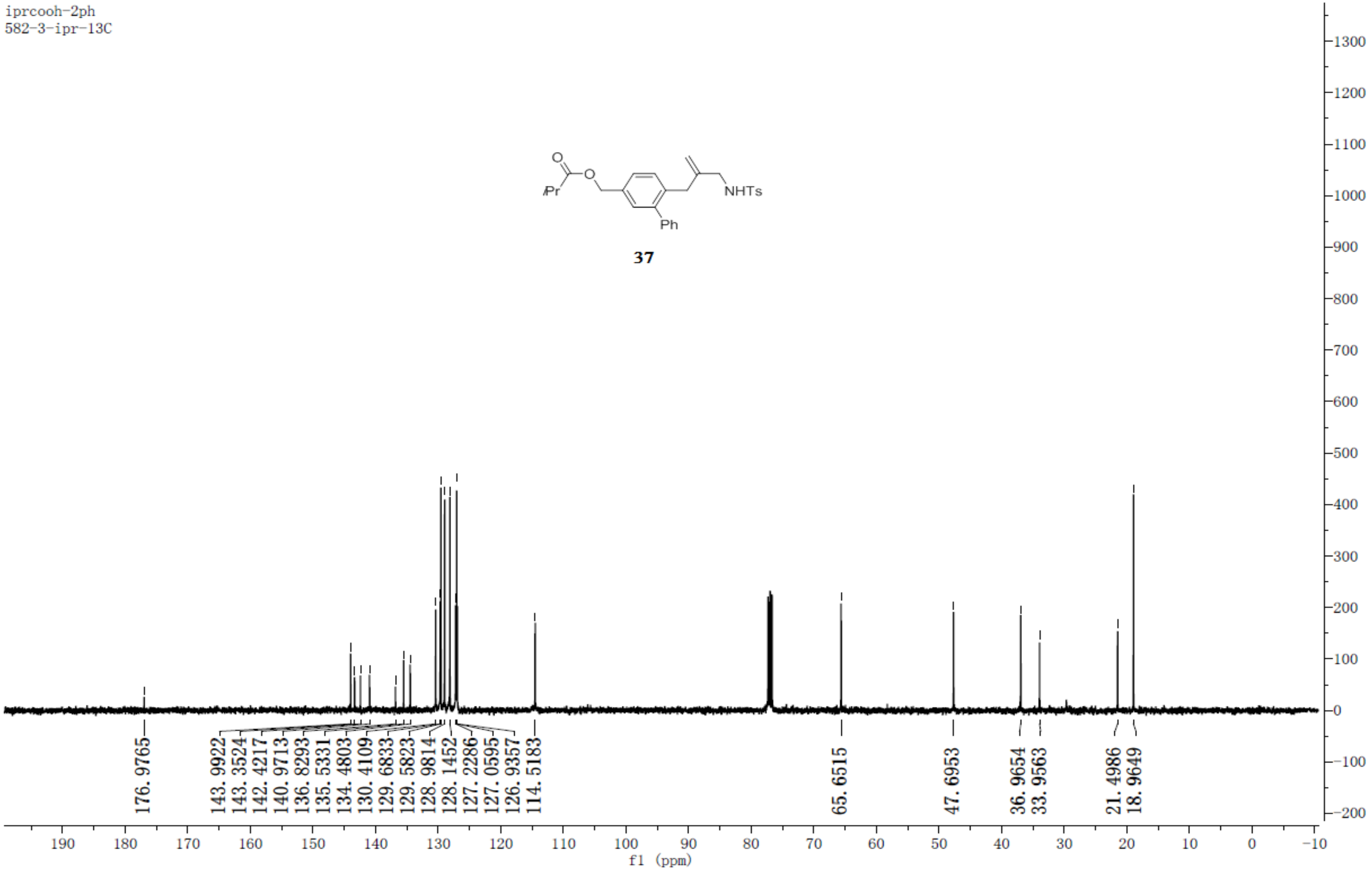

Supplementary Figure 72. ^{13}C NMR Spectrum of substrate 37

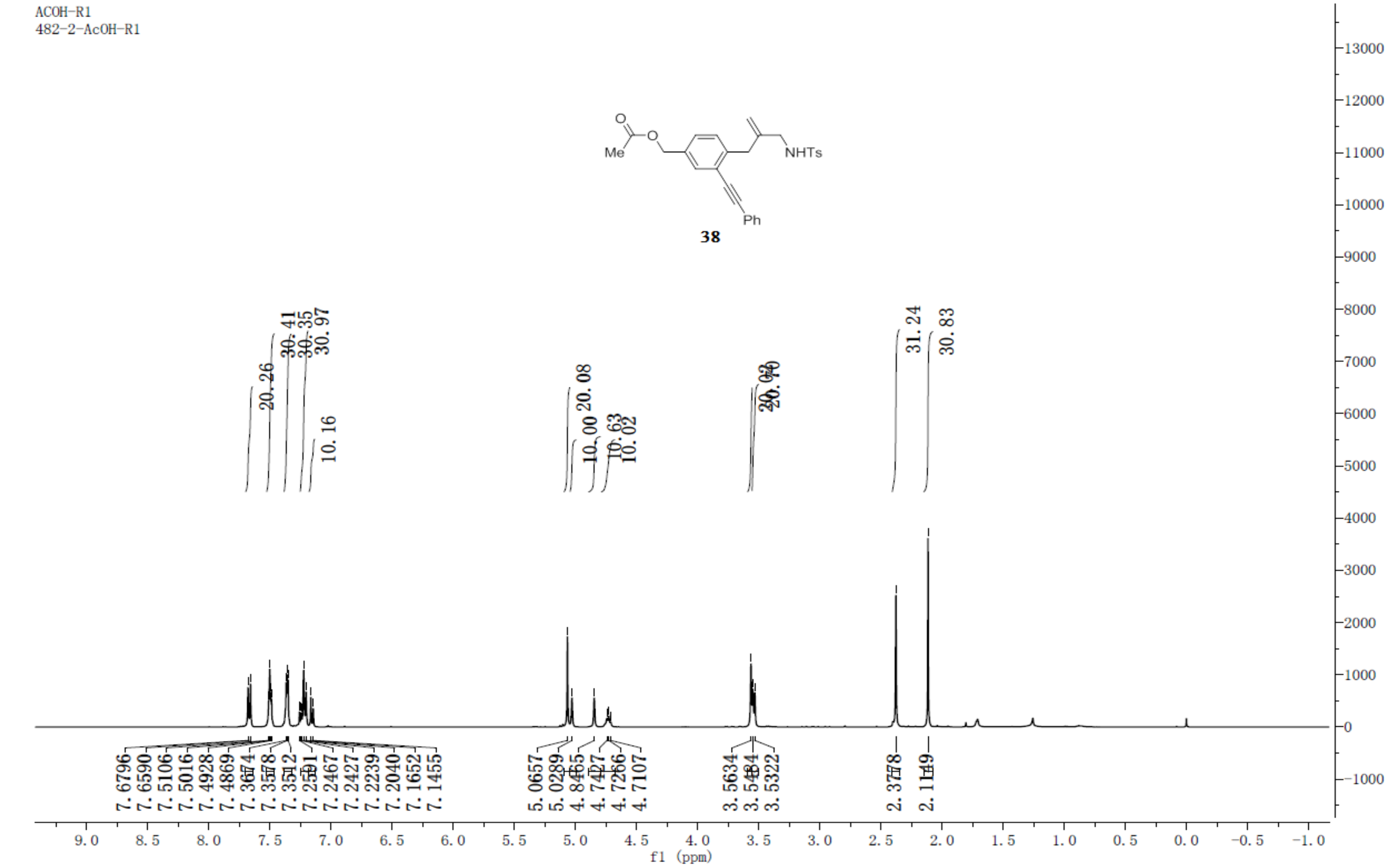

Supplementary Figure 73. ^{1}H NMR Spectrum of substrate 38

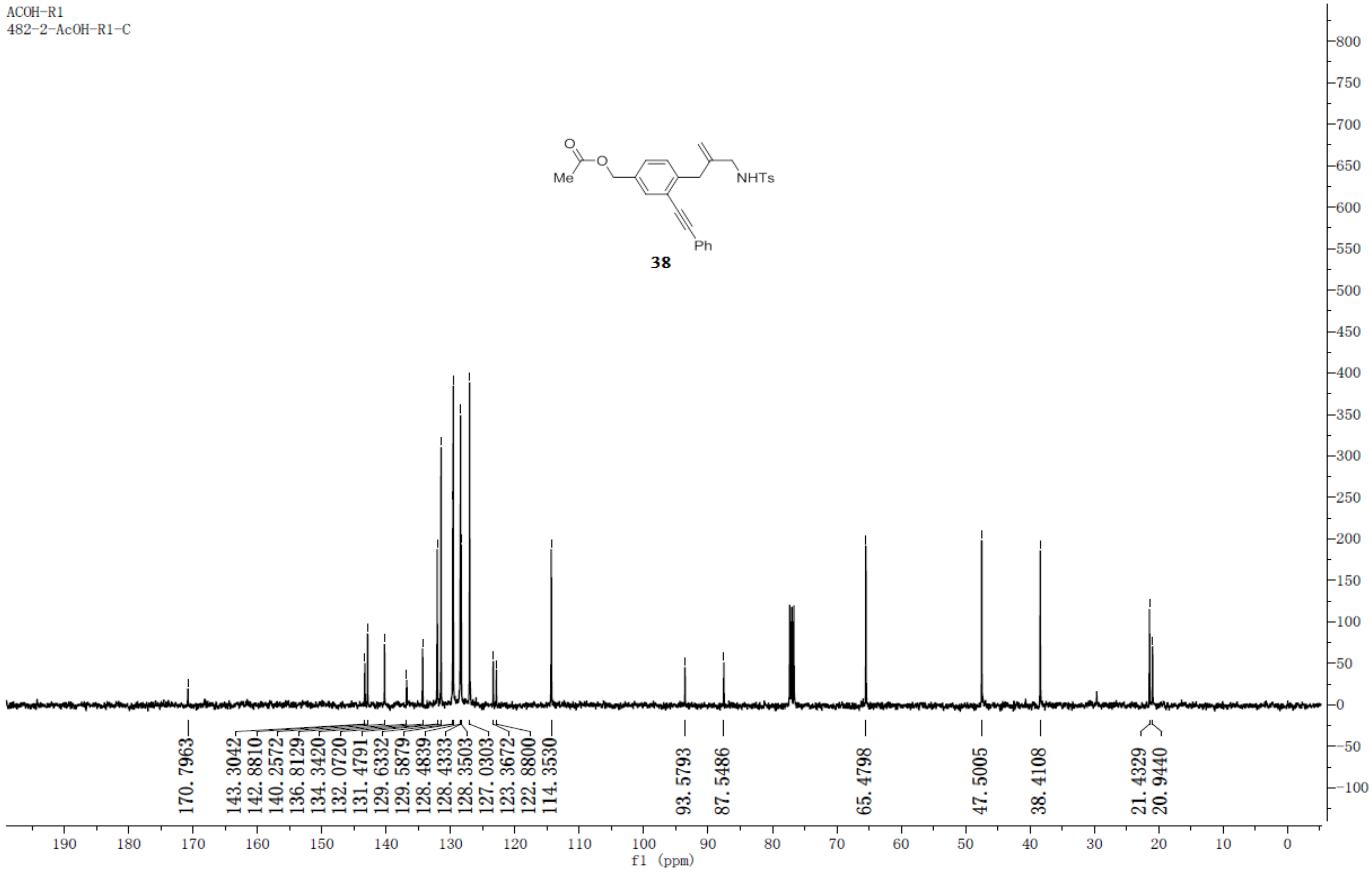

Supplementary Figure 74. ^{13}C NMR Spectrum of substrate 38

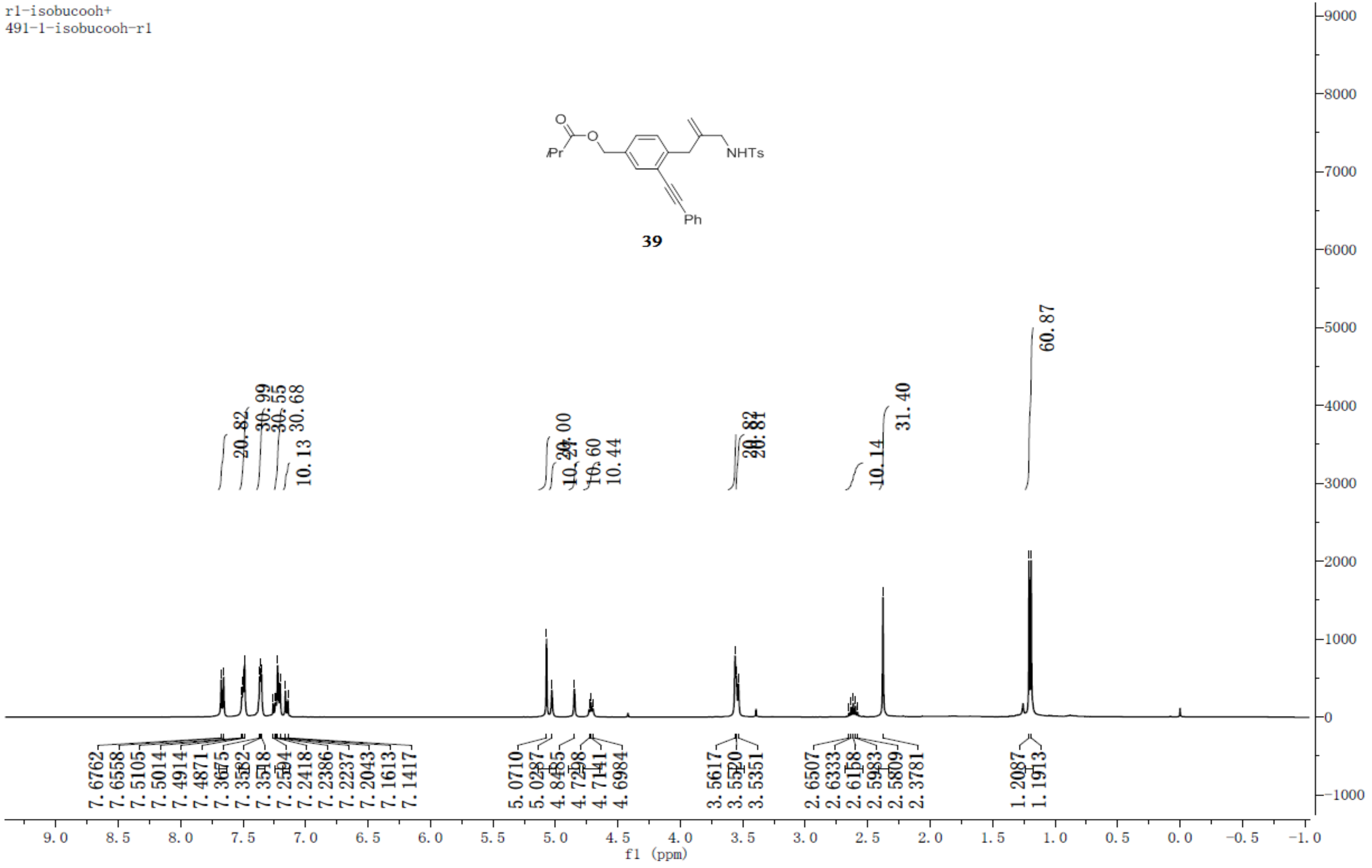

Supplementary Figure 75. ^{1}H NMR Spectrum of substrate 39

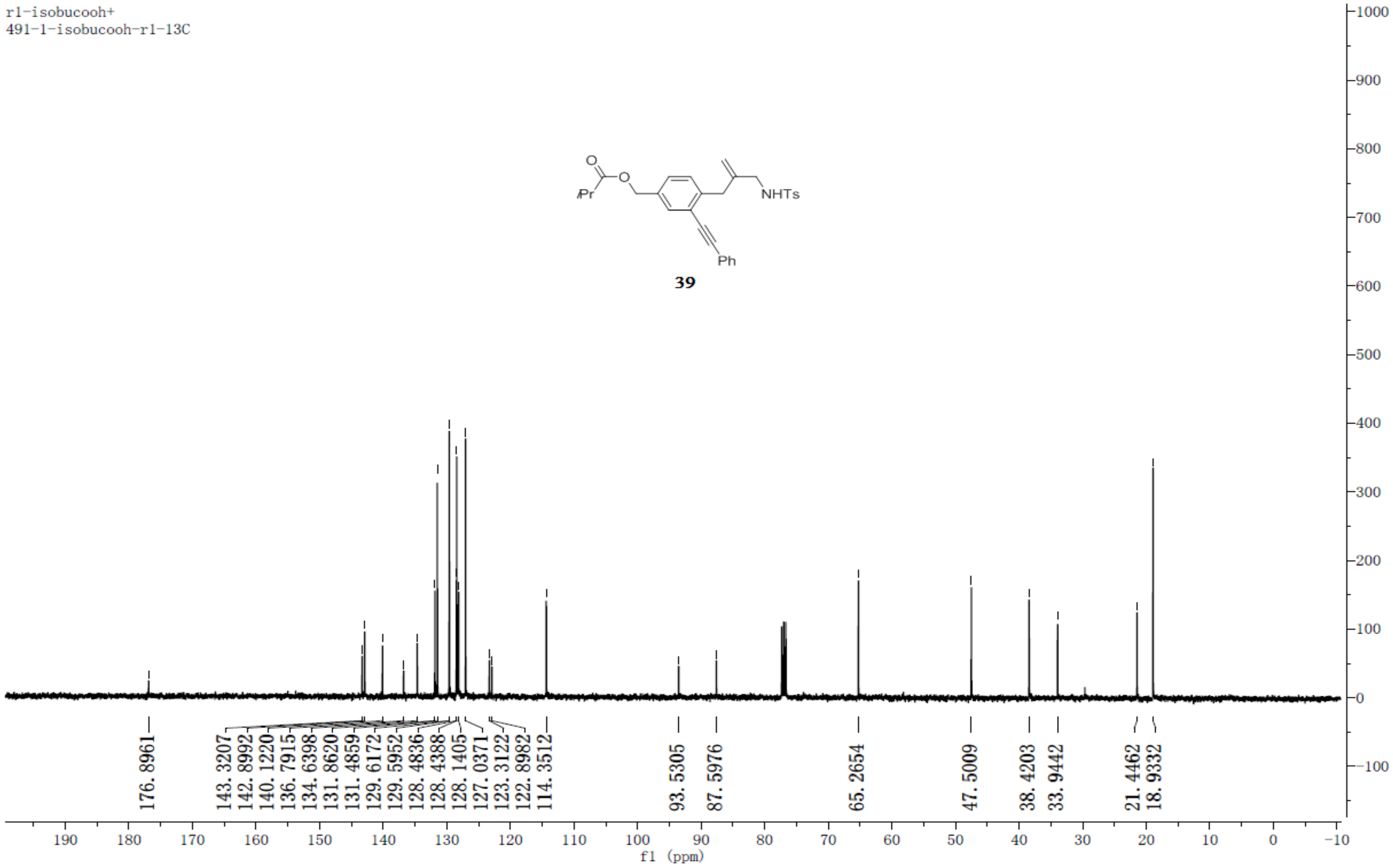

Supplementary Figure 76. ^{13}C NMR Spectrum of substrate 39

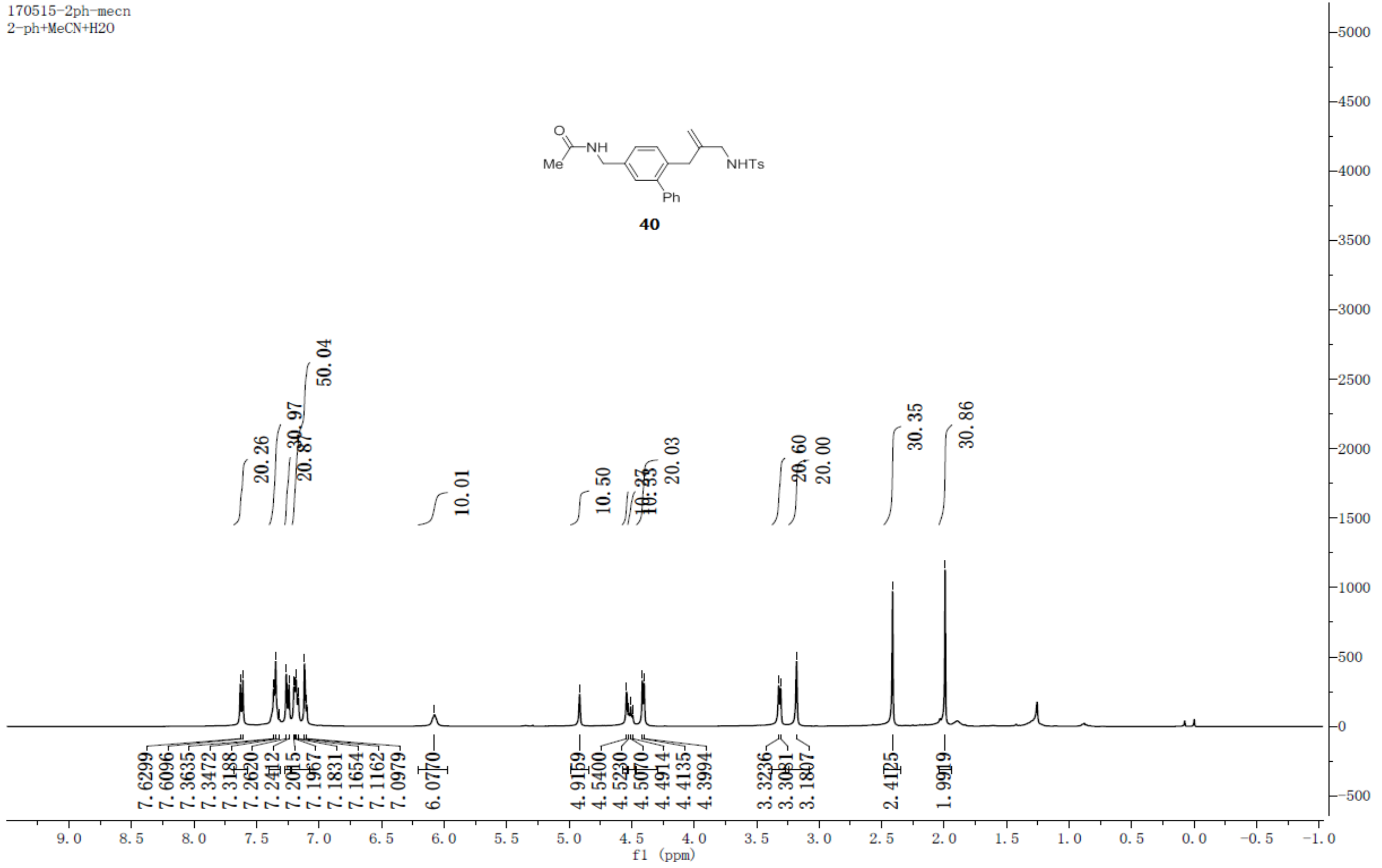

Supplementary Figure 77. ^{1}H NMR Spectrum of substrate 40

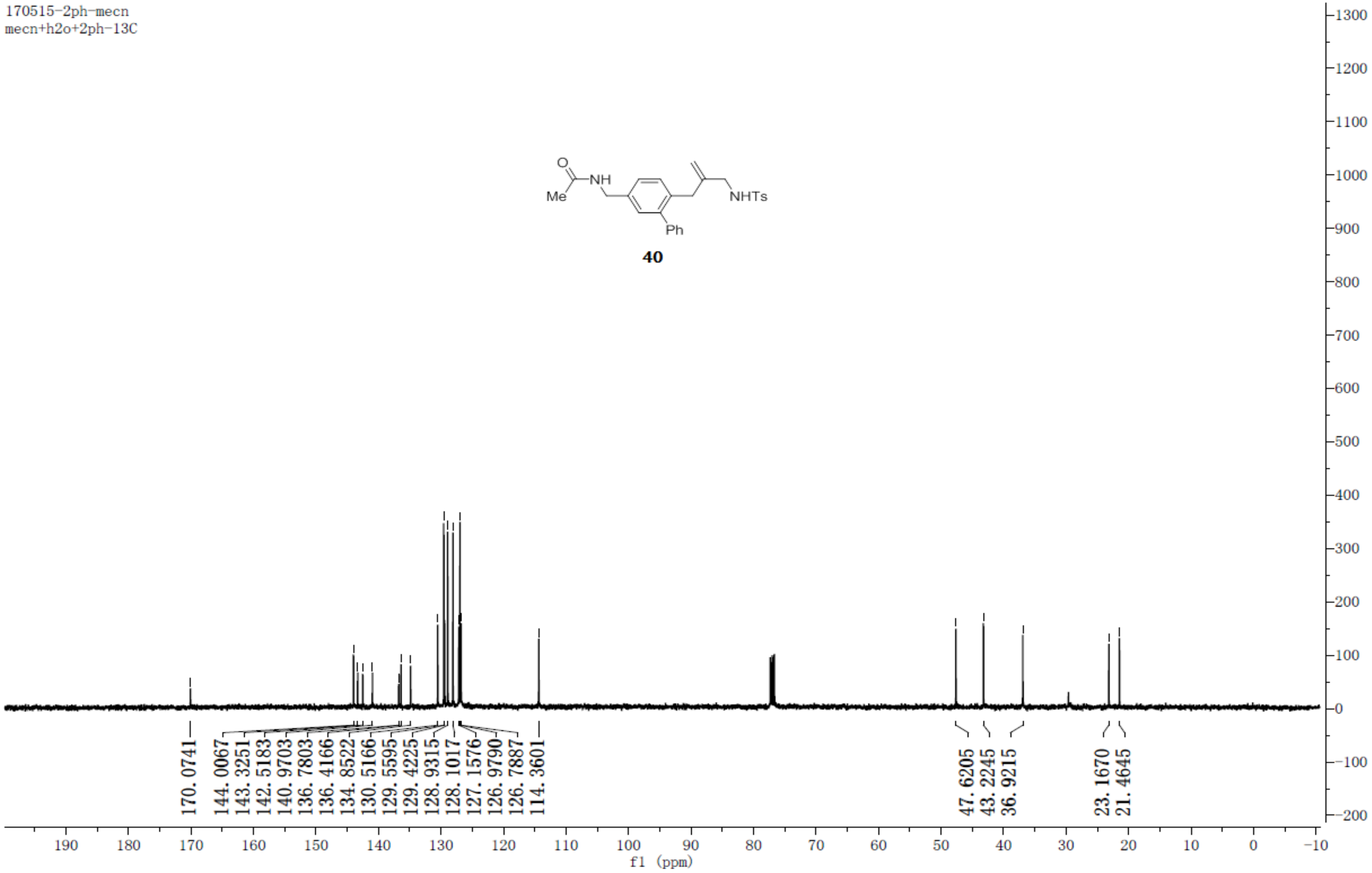

Supplementary Figure 78. ^{13}C NMR Spectrum of substrate 40

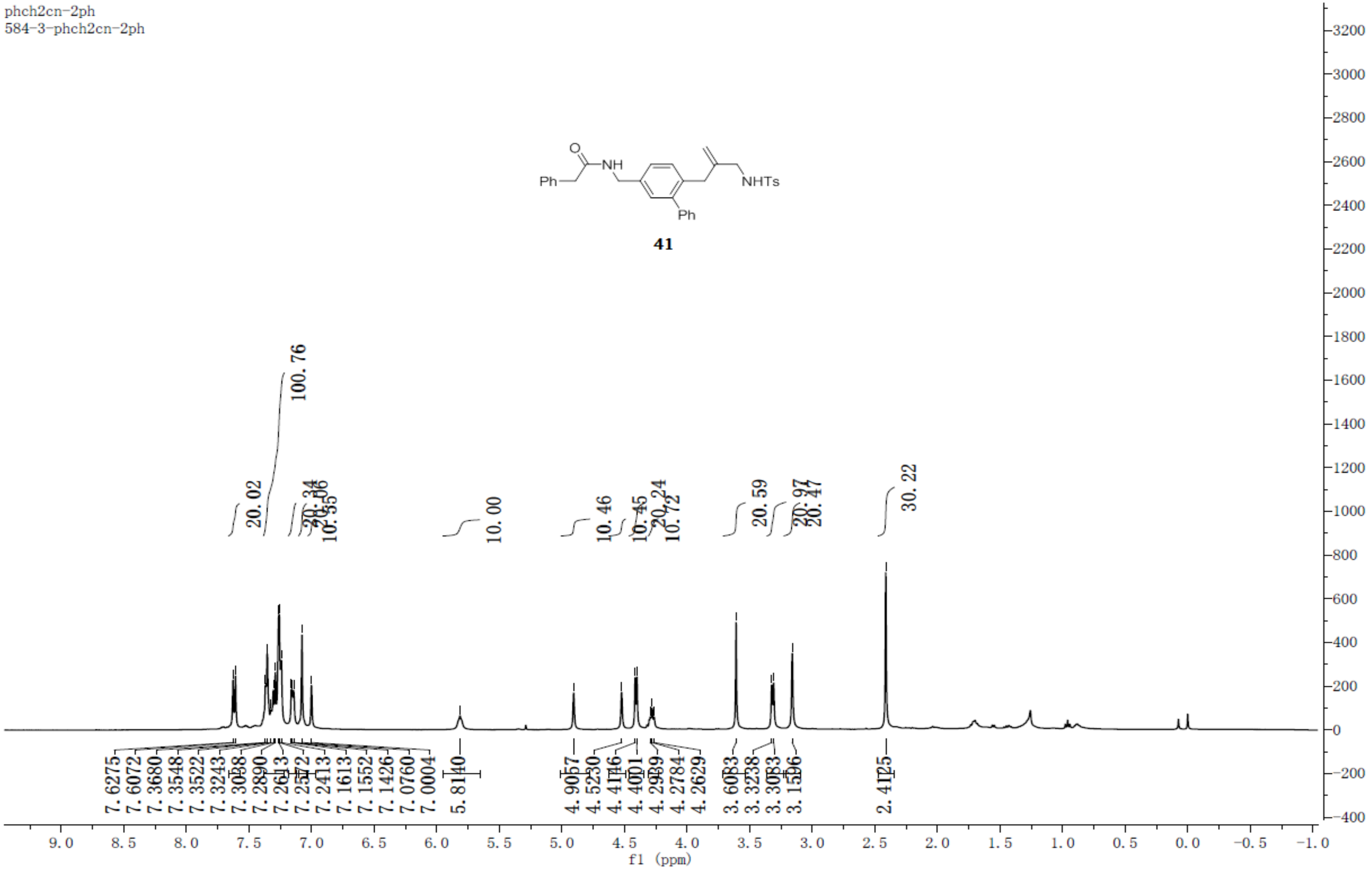

Supplementary Figure 79. ^{1}H NMR Spectrum of substrate 41

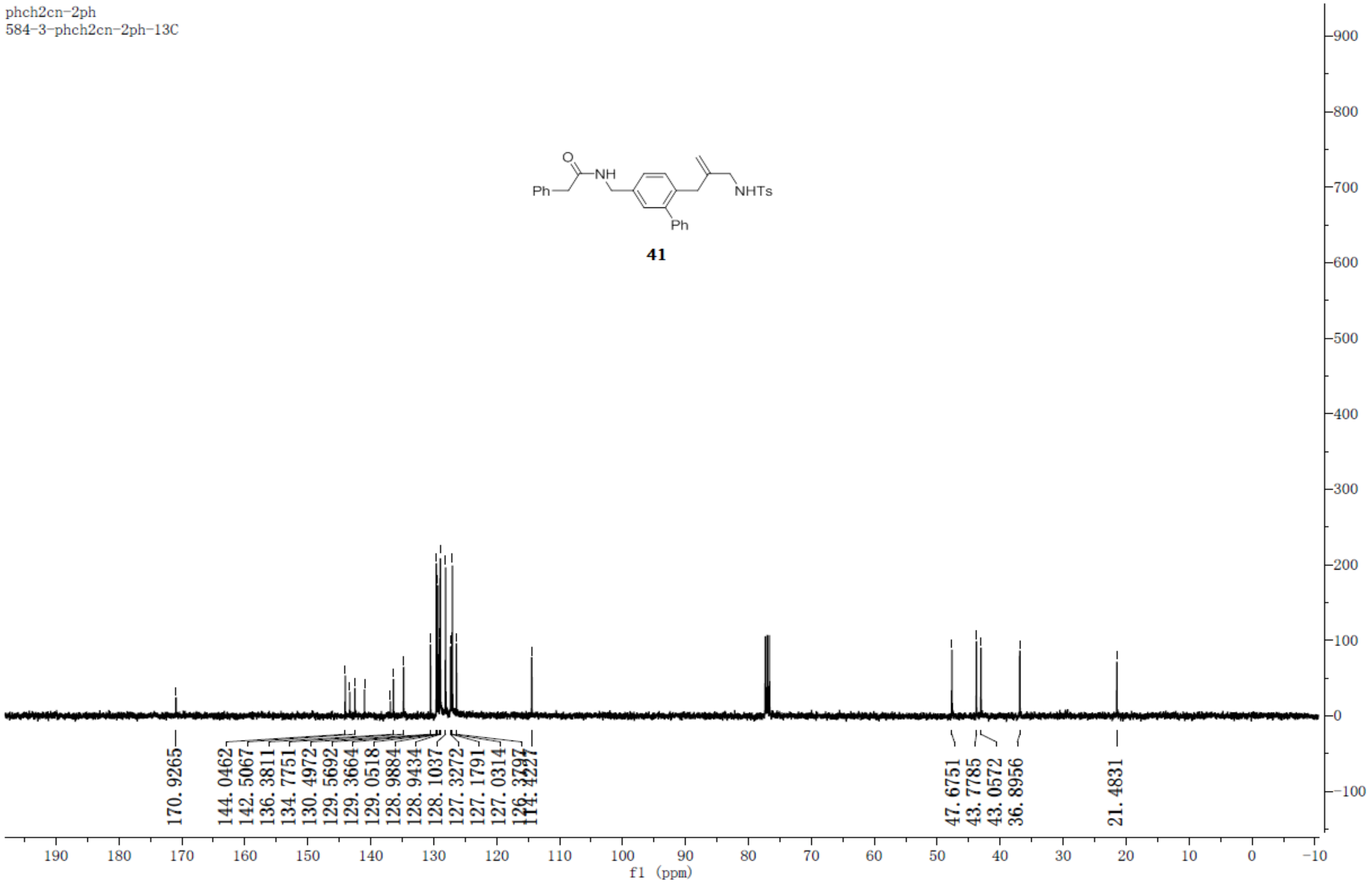

Supplementary Figure 80. ^{13}C NMR Spectrum of substrate 41

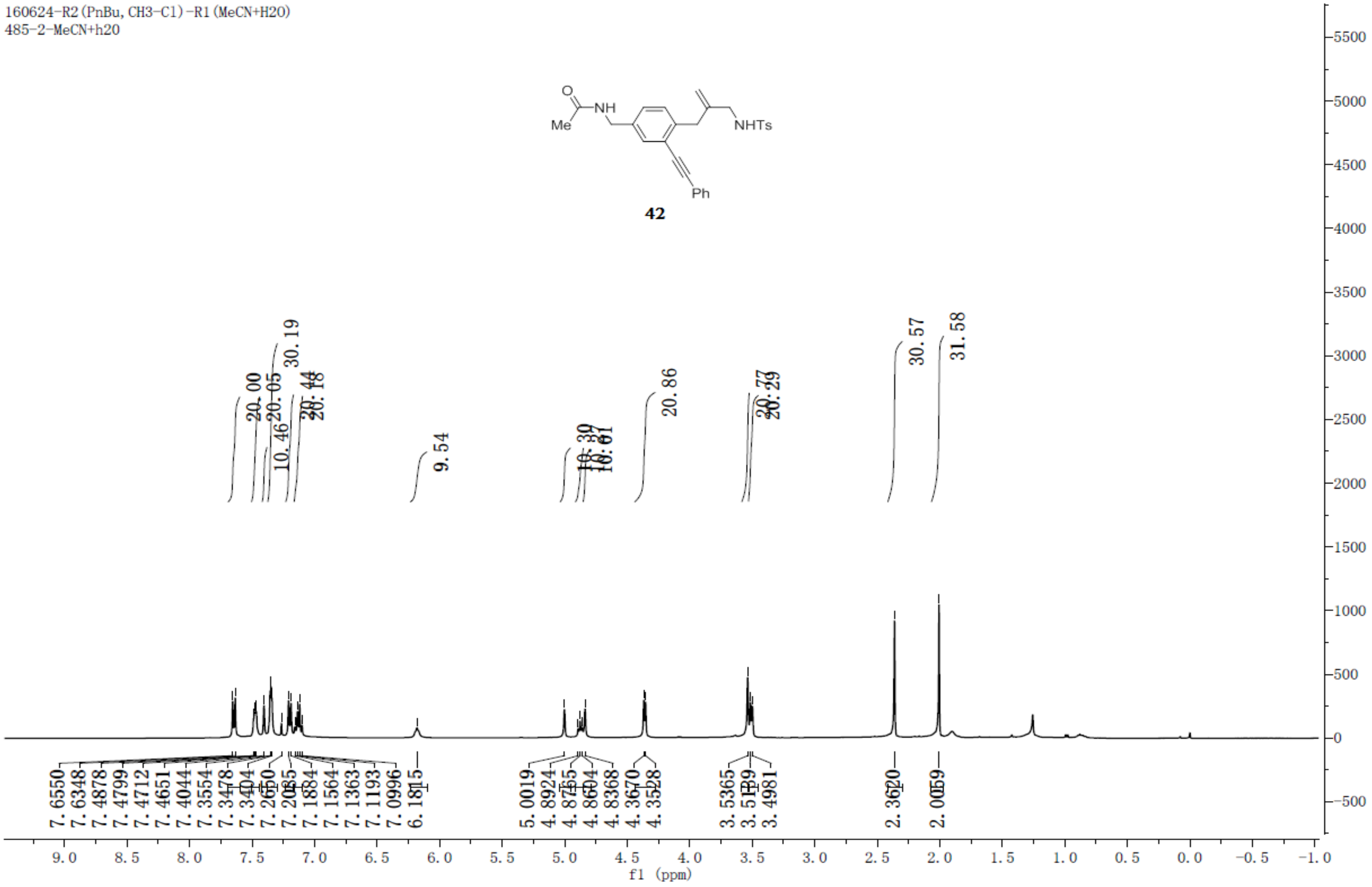

Supplementary Figure 81. ^{1}H NMR Spectrum of substrate 42

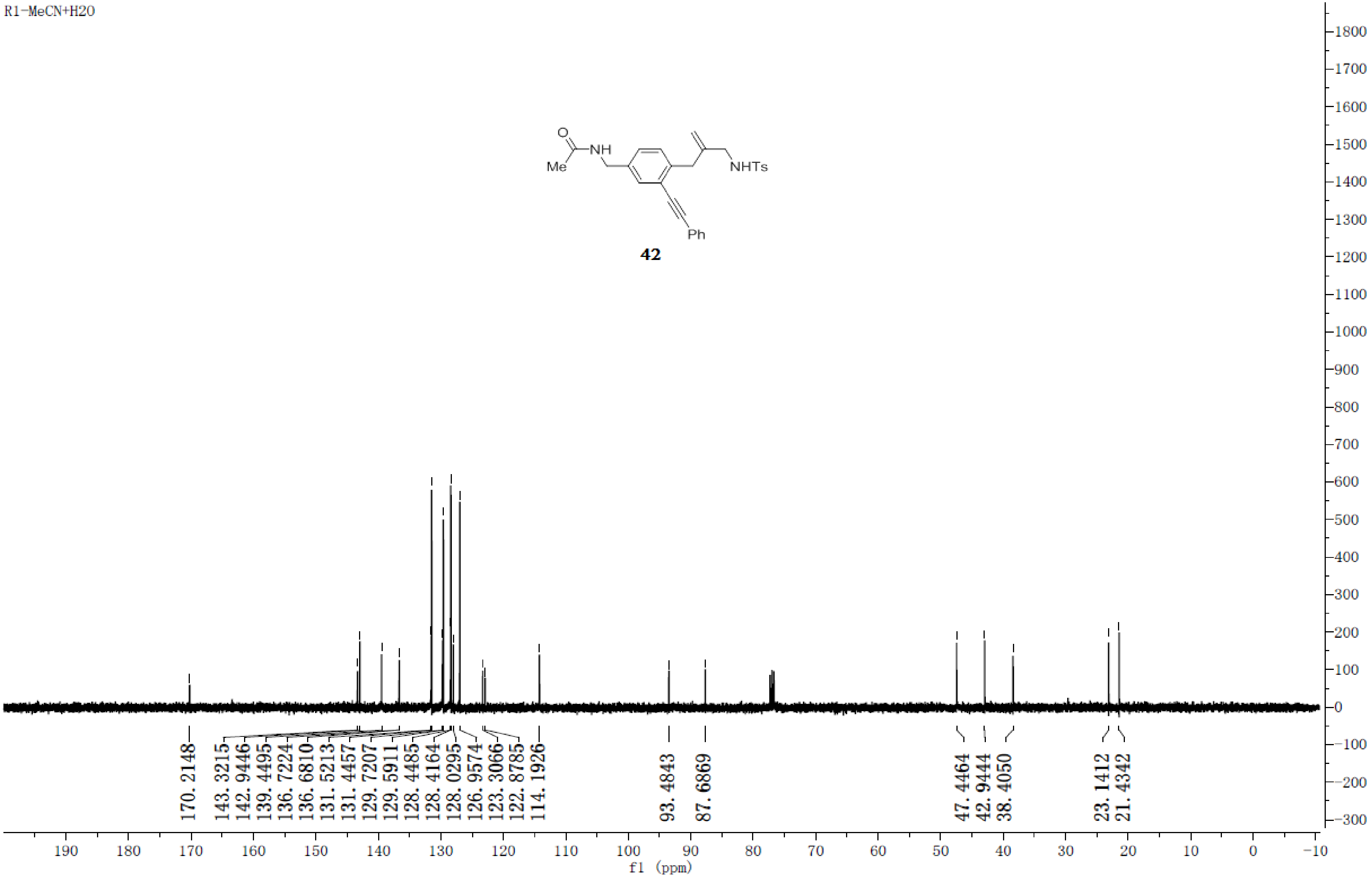

Supplementary Figure 82. ^{13}C NMR Spectrum of substrate 42

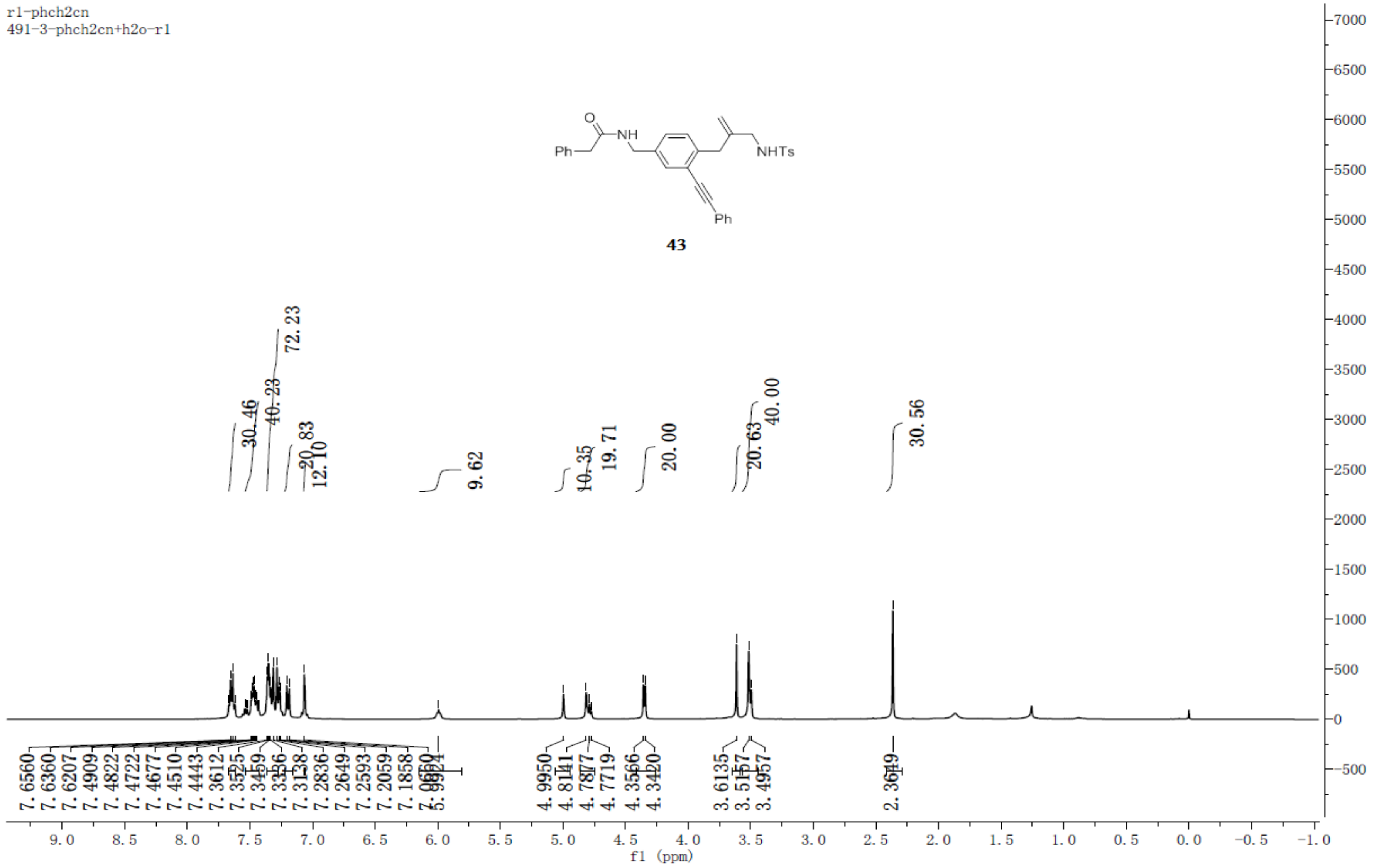

Supplementary Figure 83. ^{1}H NMR Spectrum of substrate 43

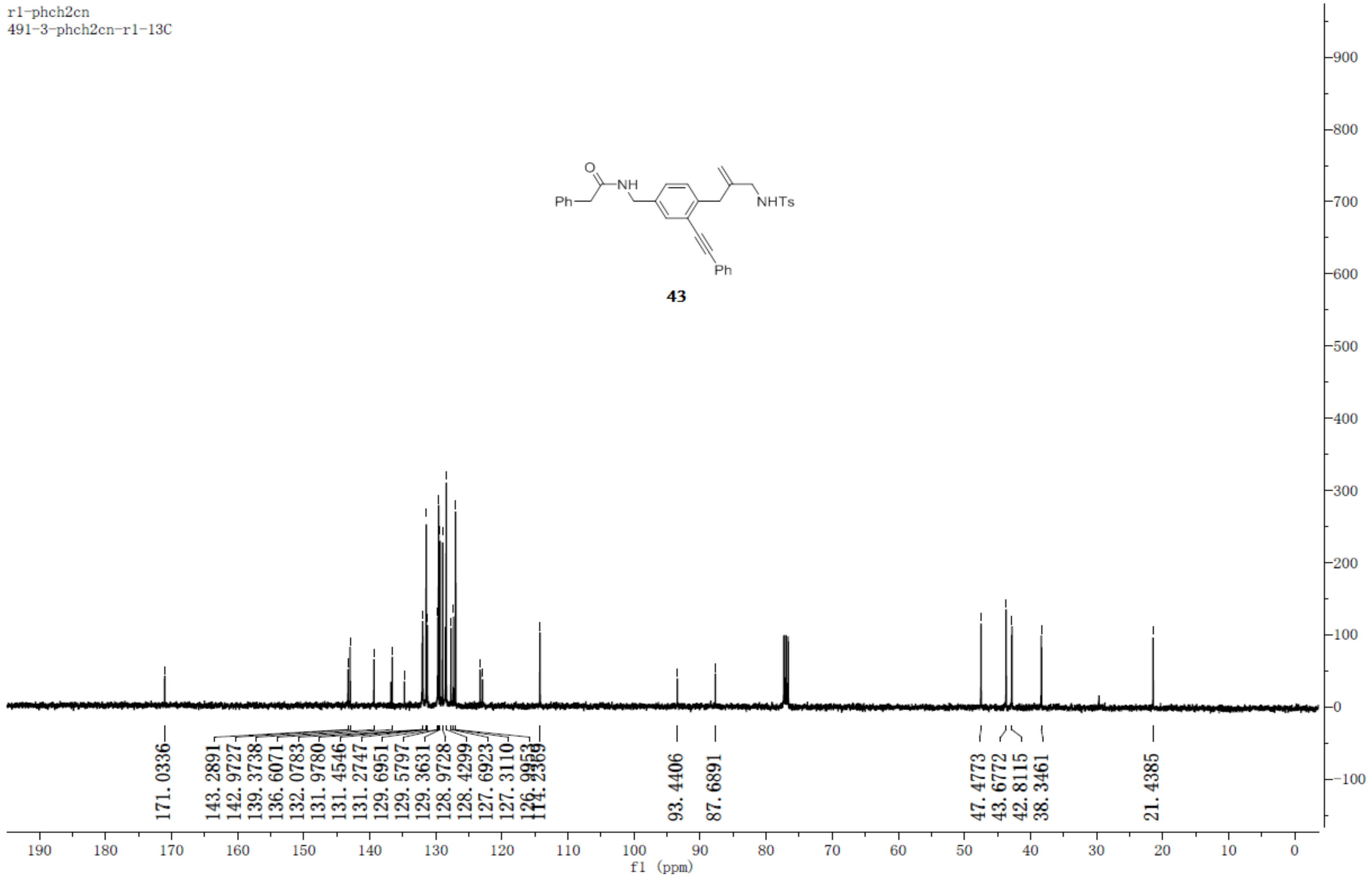

Supplementary Figure 84. ^{13}C NMR Spectrum of substrate 43

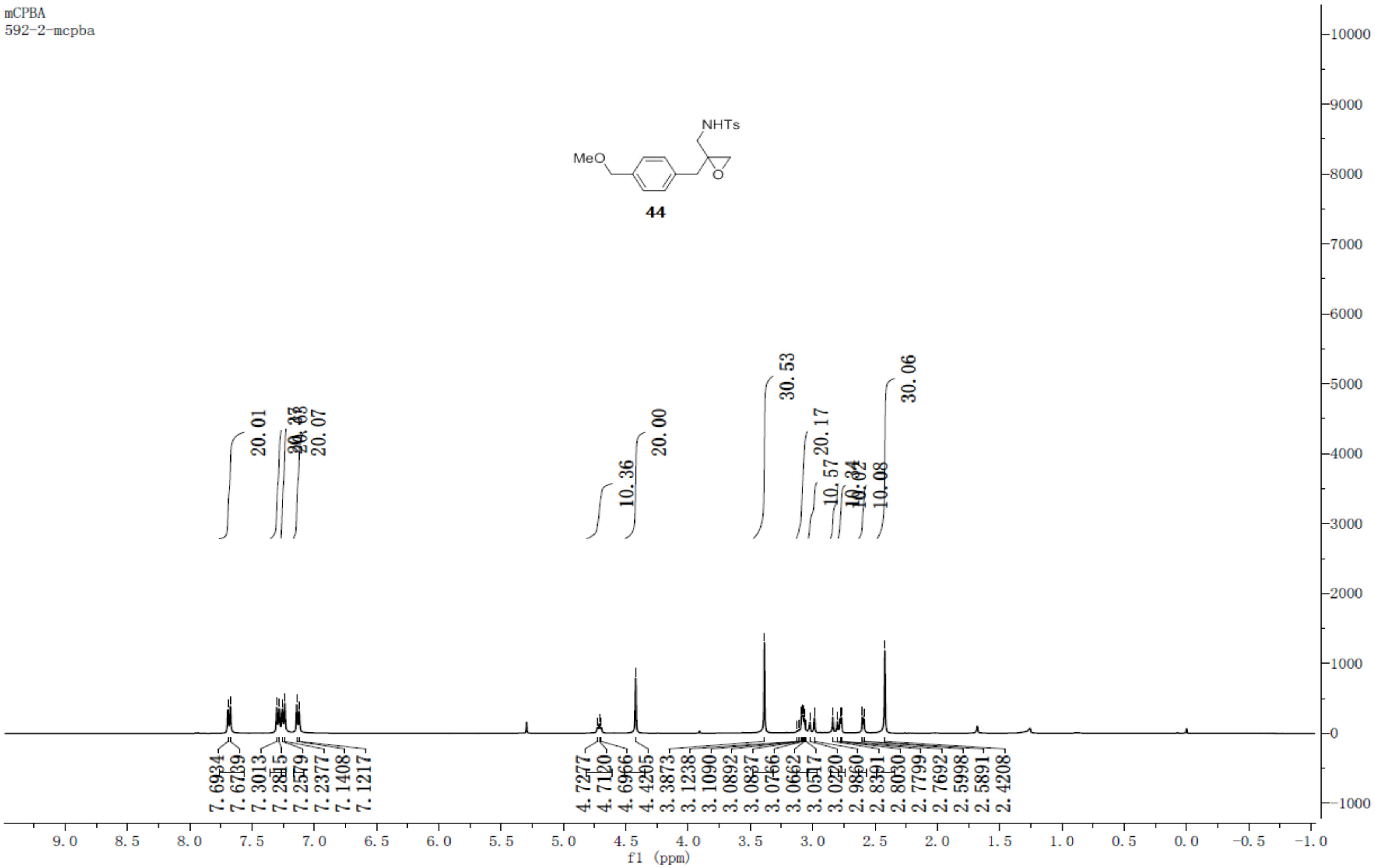

Supplementary Figure 85. ^{1}H NMR Spectrum of substrate 44

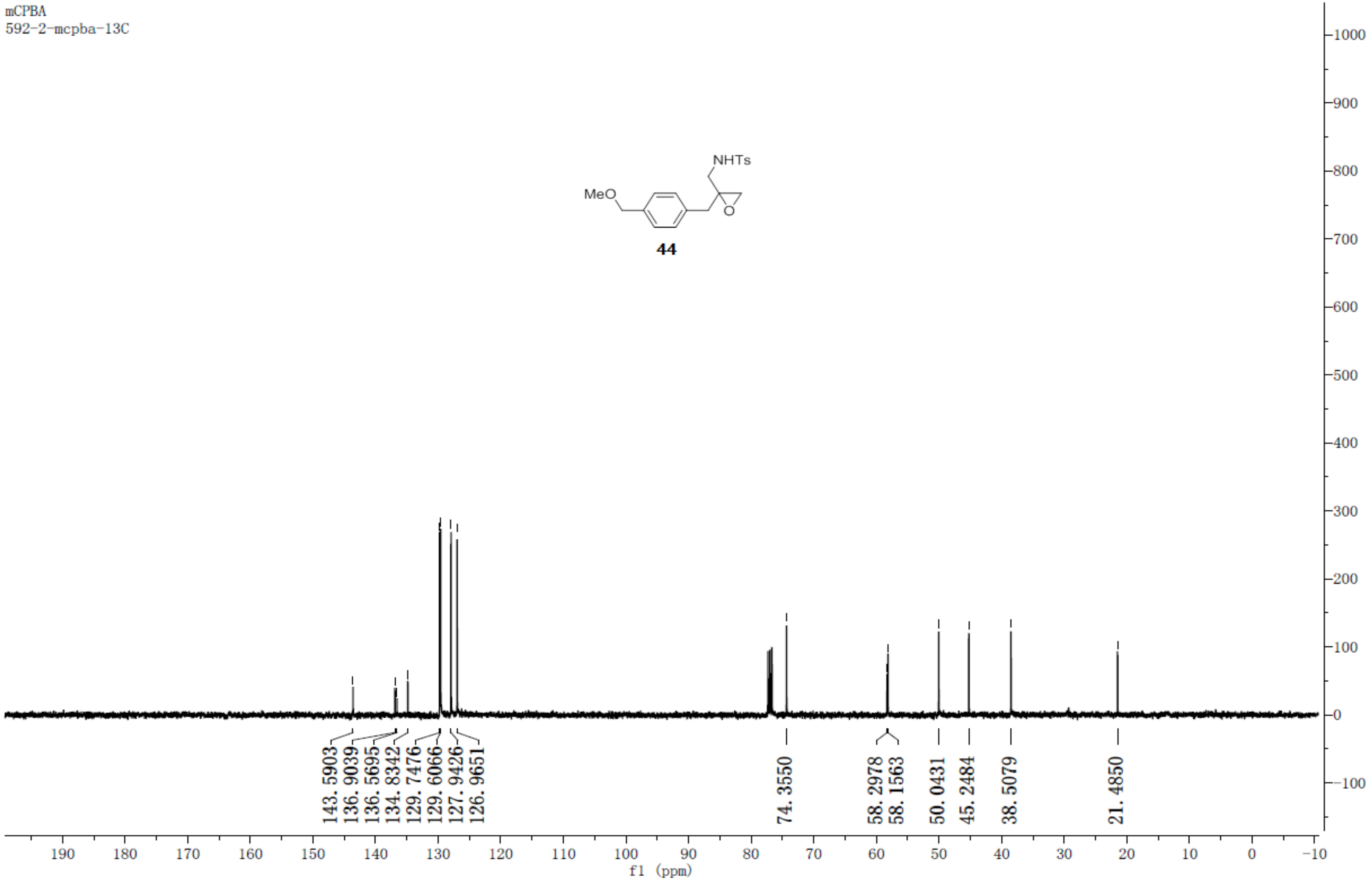

Supplementary Figure 86. ^{13}C NMR Spectrum of substrate 44

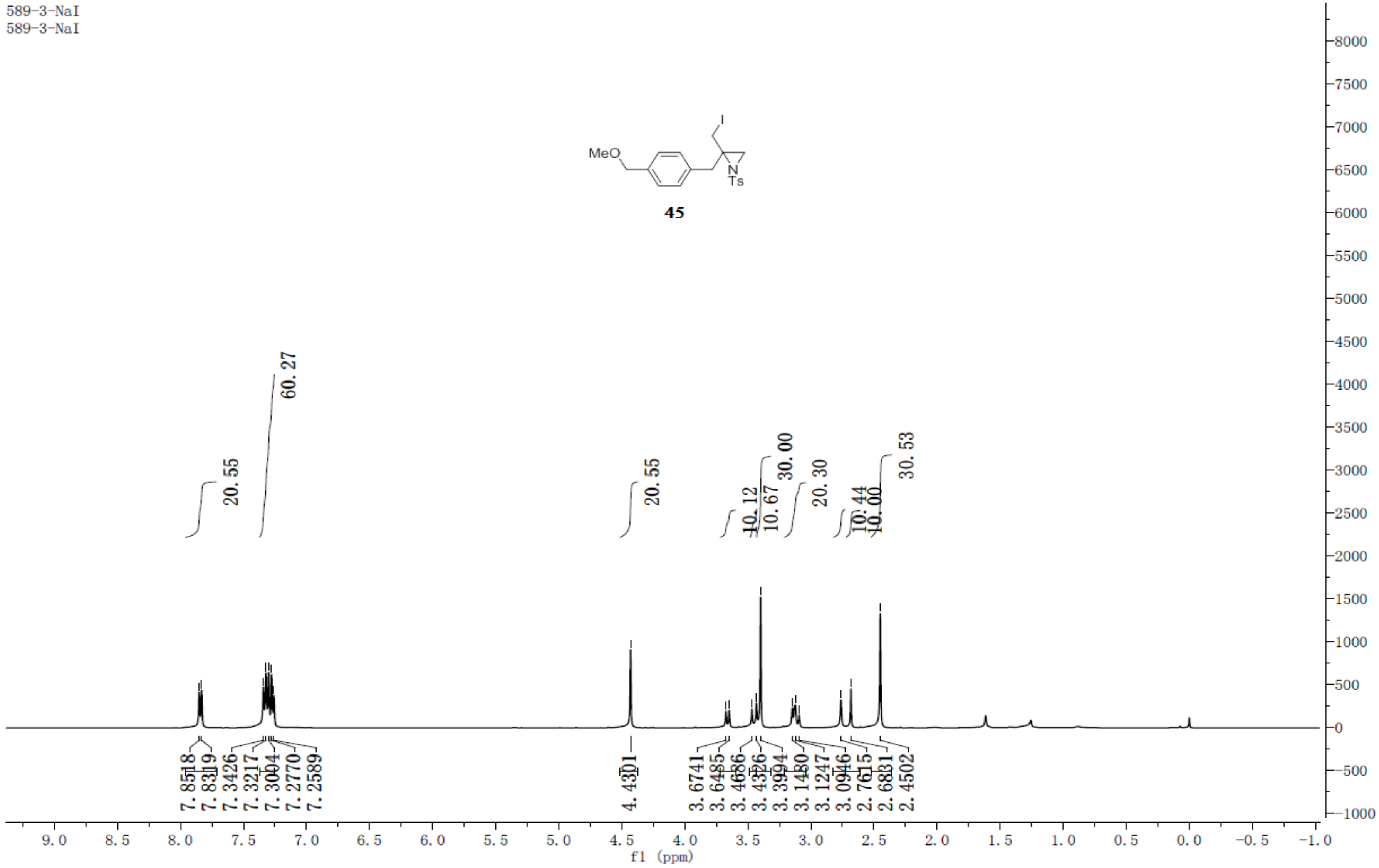

Supplementary Figure 87. ^{1}H NMR Spectrum of substrate 45

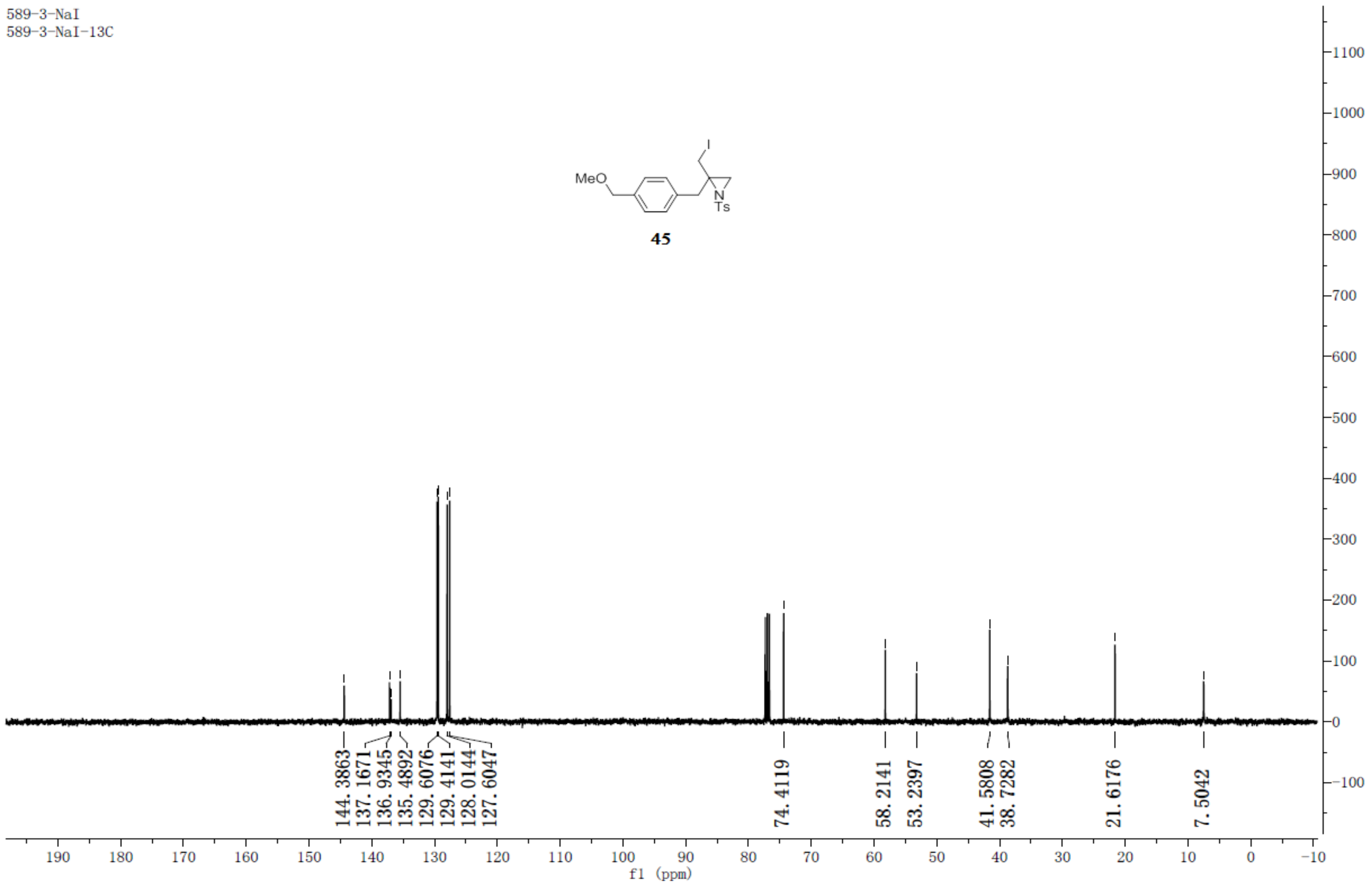

Supplementary Figure 88. ^{13}C NMR Spectrum of substrate 45

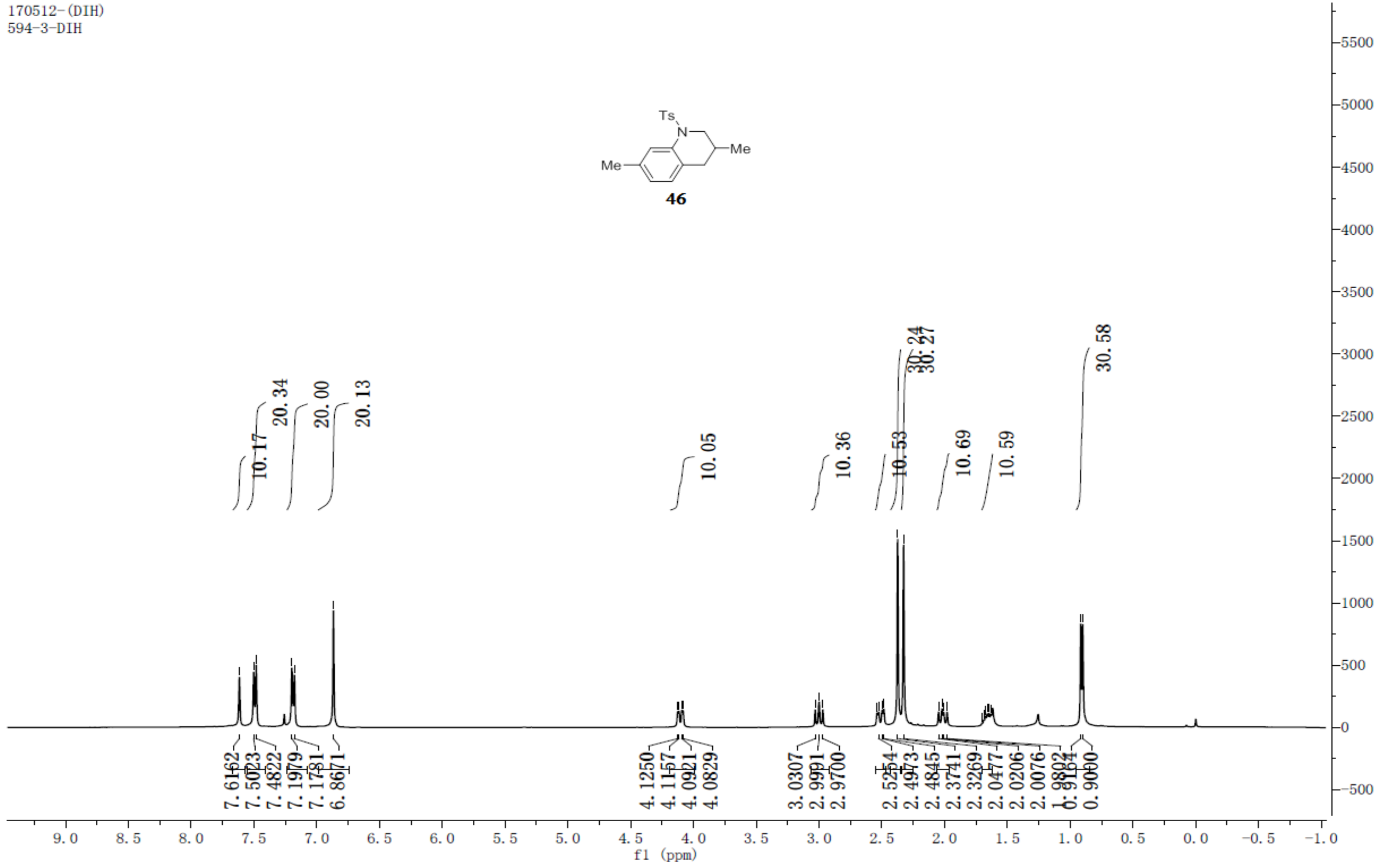

Supplementary Figure 89. ^{1}H NMR Spectrum of substrate 46

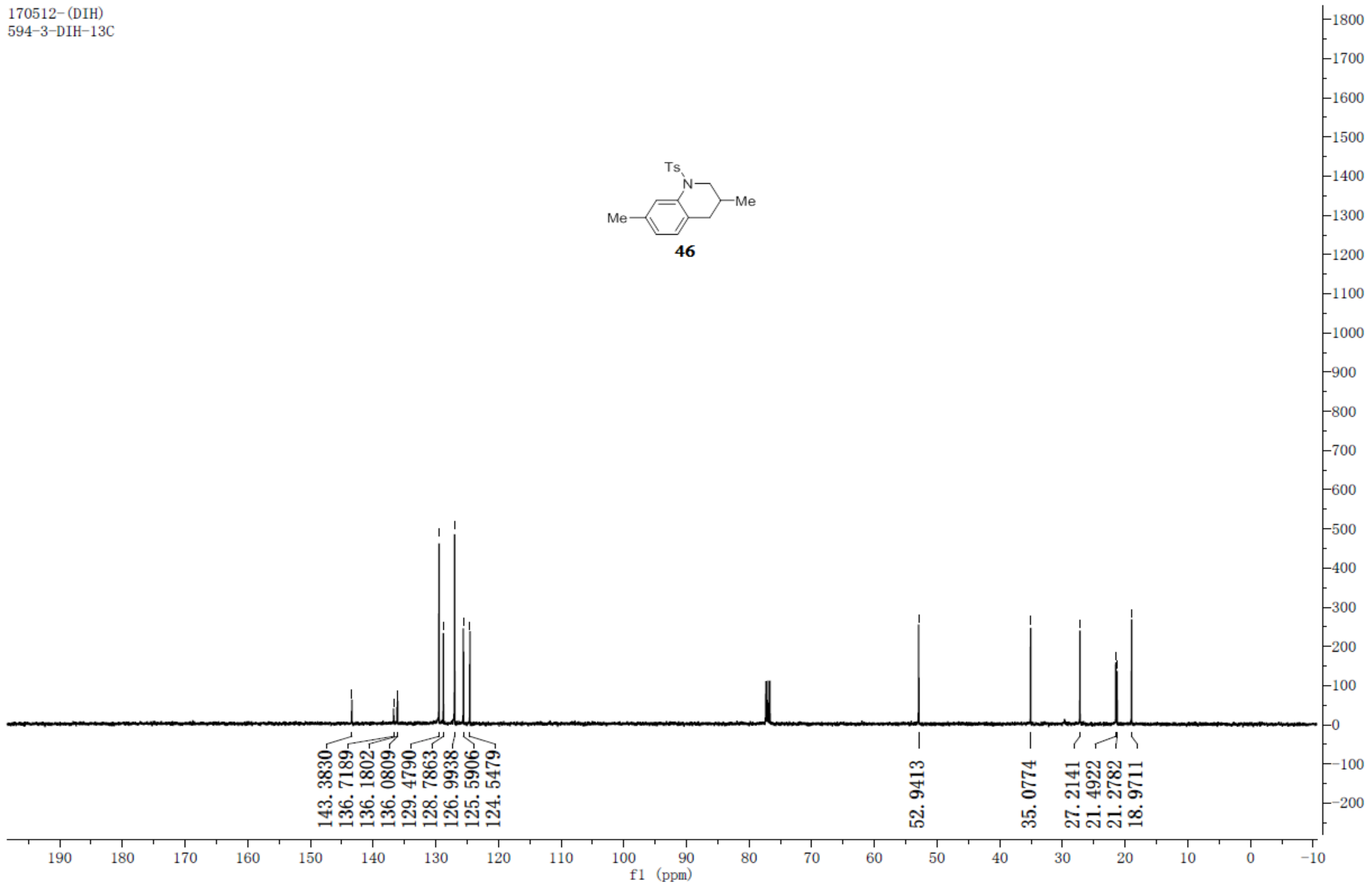

Supplementary Figure 90. 13C NMR Spectrum of substrate 46

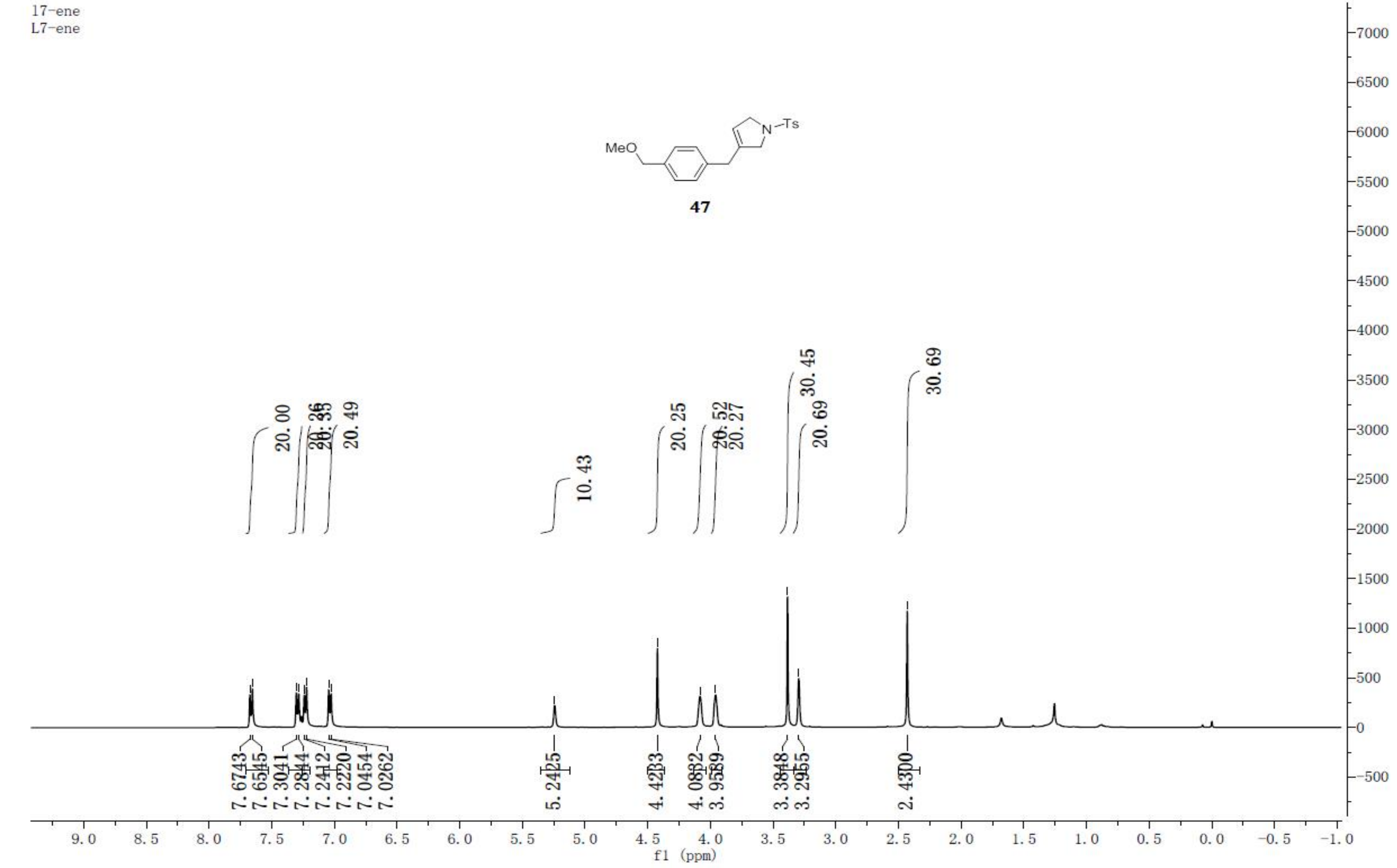

Supplementary Figure 91. ^{1}H NMR Spectrum of substrate 47

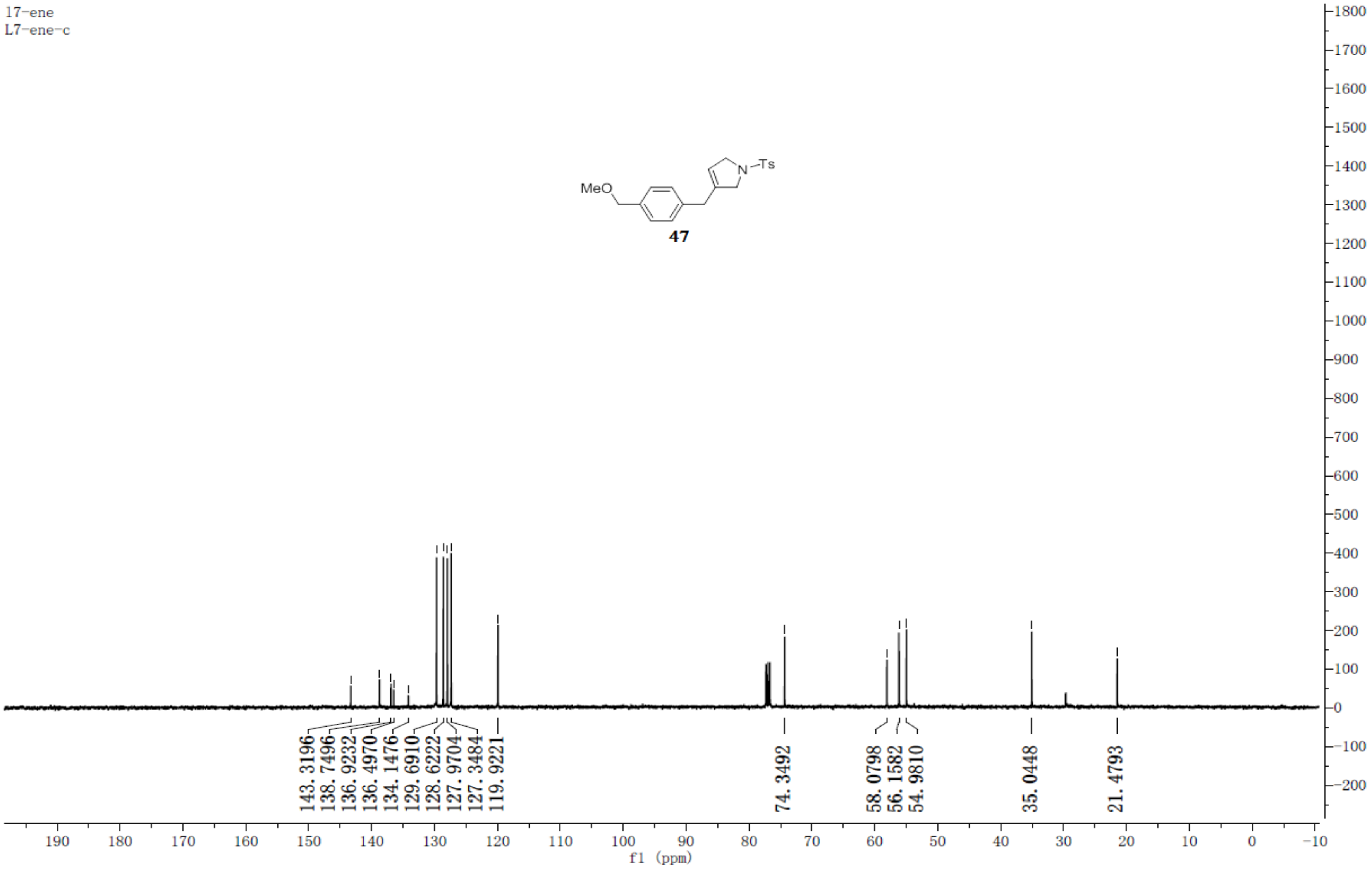

Supplementary Figure 92. ^{13}C NMR Spectrum of substrate 47

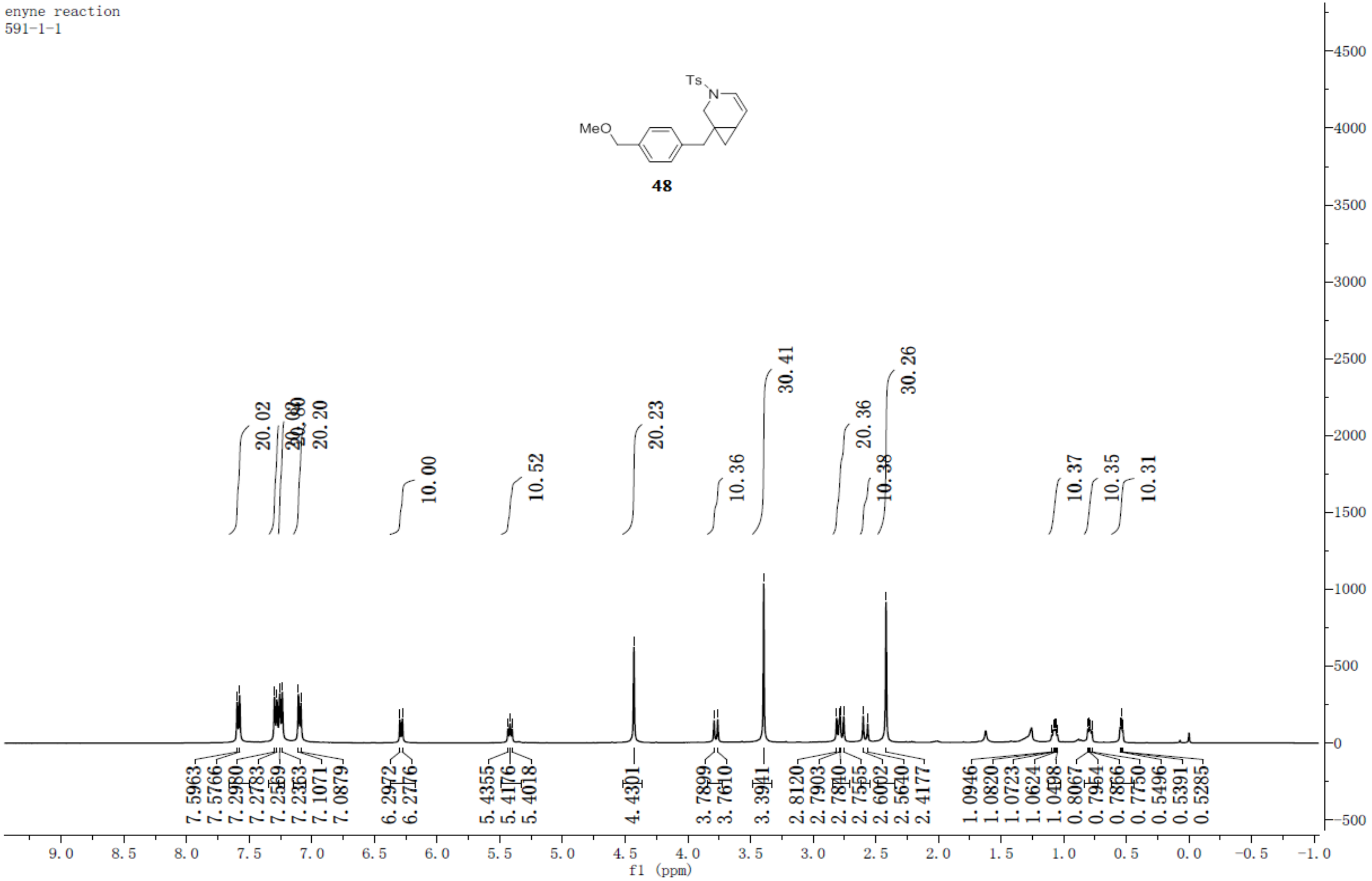

Supplementary Figure 93. ^{1}H NMR Spectrum of substrate 48

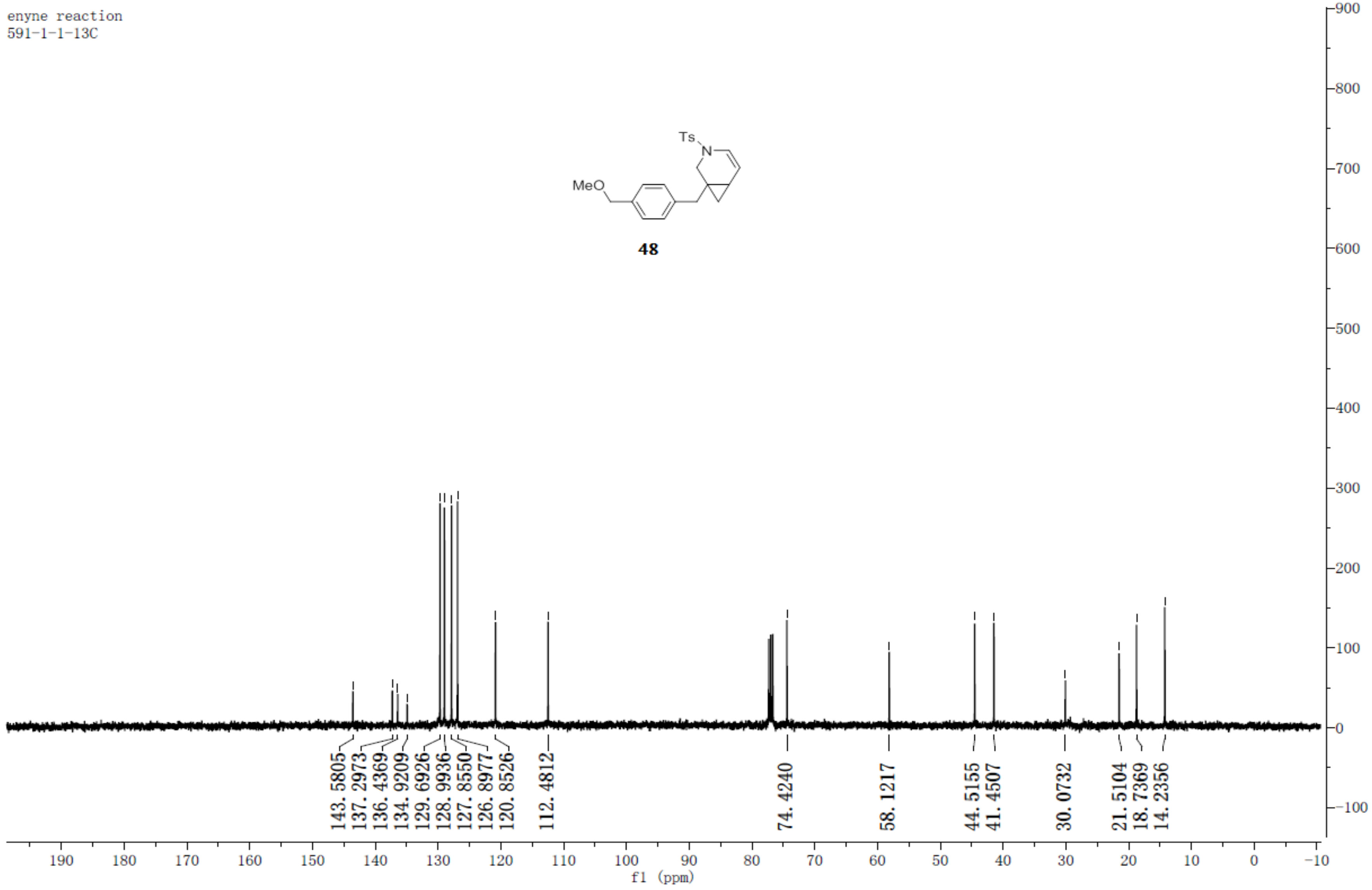

Supplementary Figure 94. ^{13}C NMR Spectrum of substrate 48

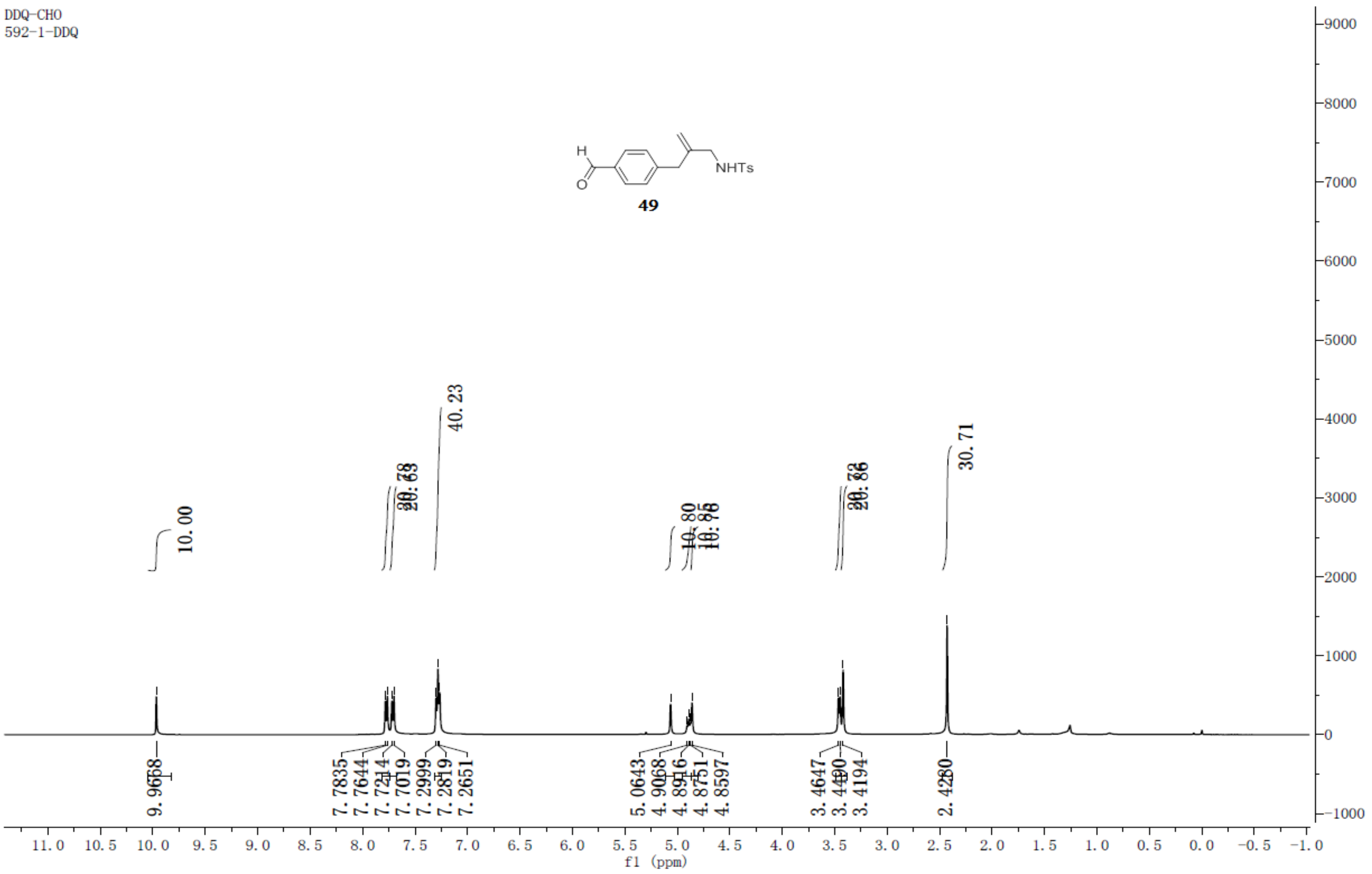

Supplementary Figure 95. ^{1}H NMR Spectrum of substrate 49

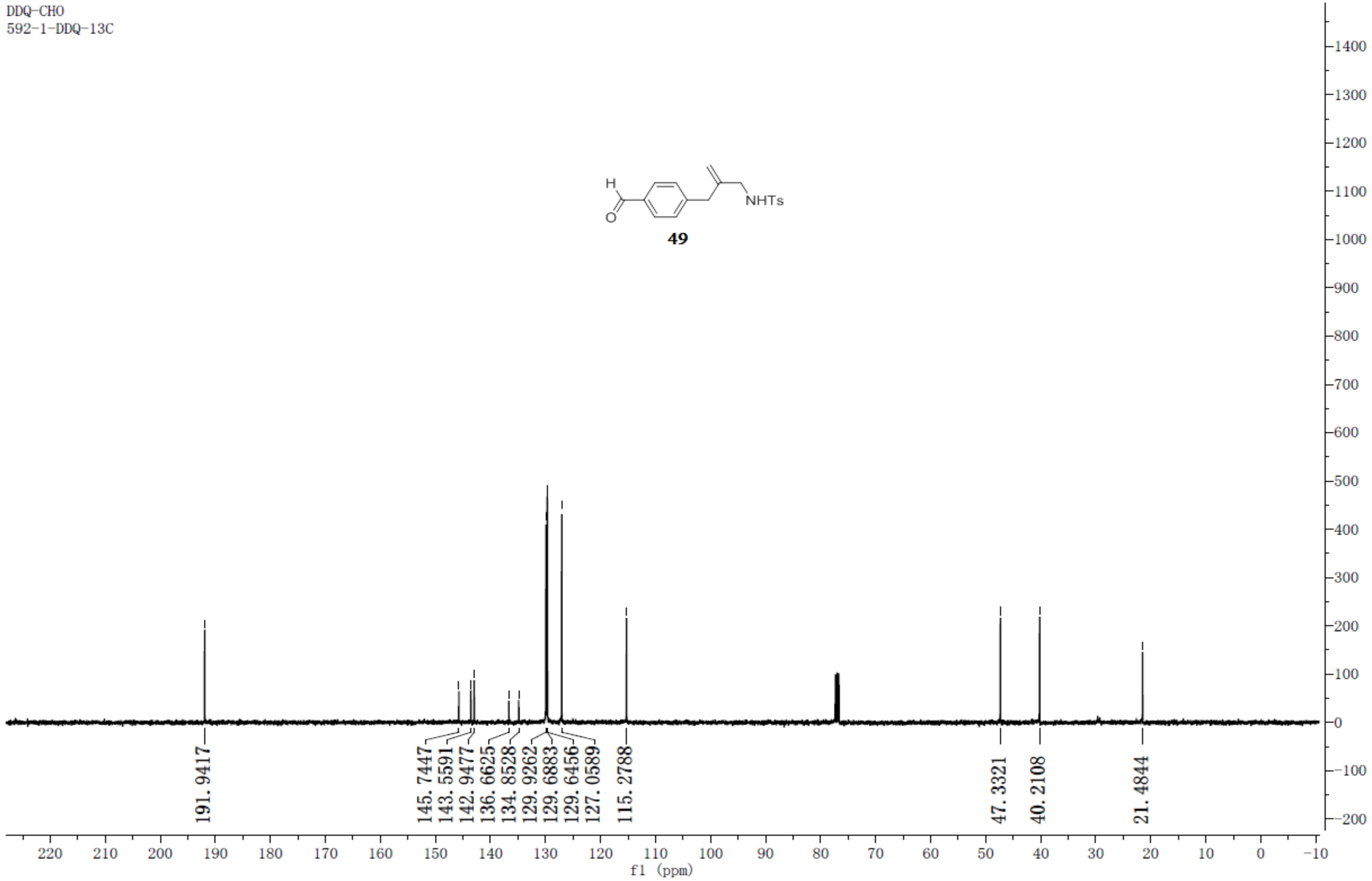

Supplementary Figure 96. ^{13}C NMR Spectrum of substrate 49

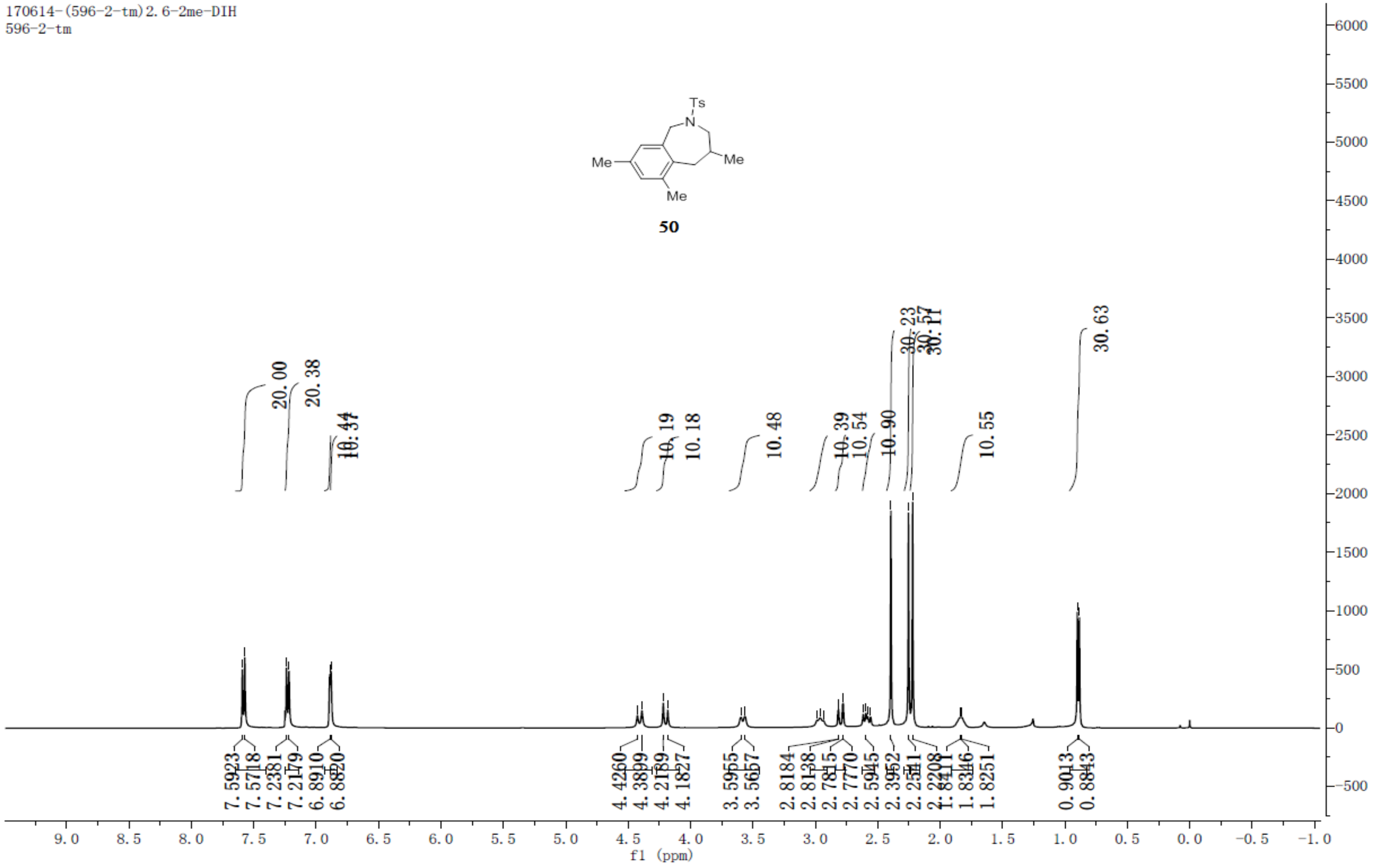

Supplementary Figure 97. ^{1}H NMR Spectrum of substrate 50

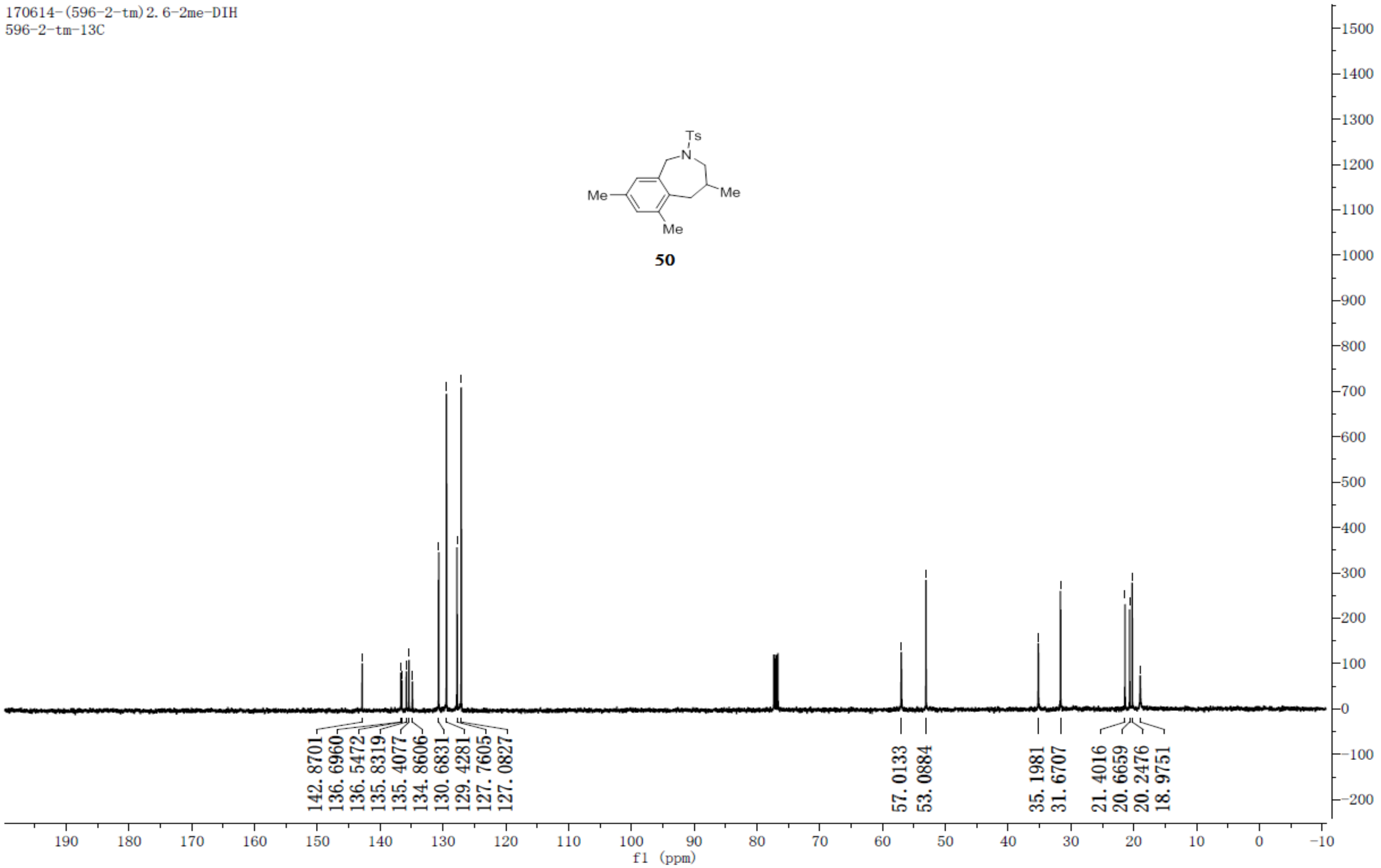

Supplementary Figure 98. ^{13}C NMR Spectrum of substrate 50

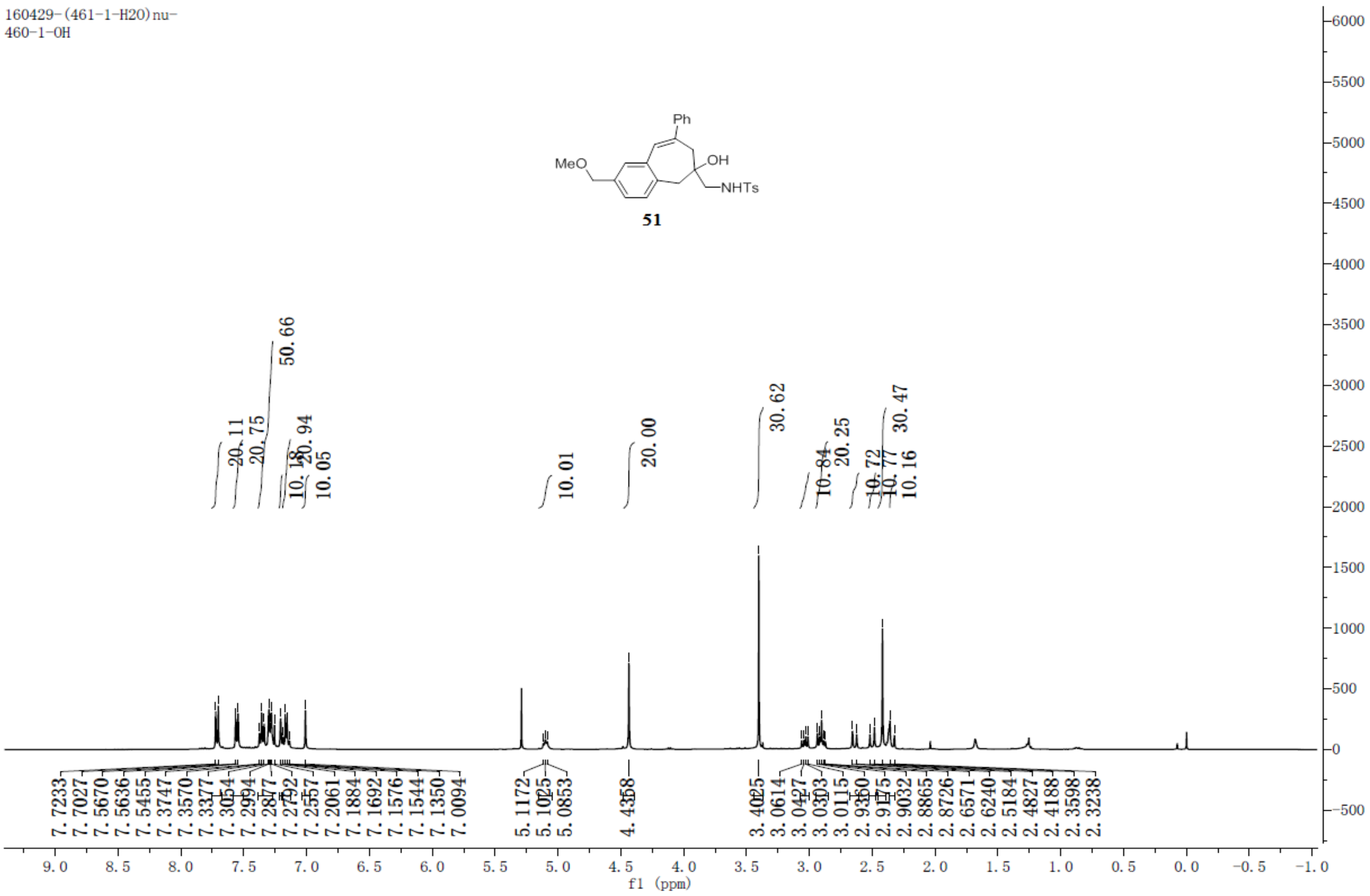

Supplementary Figure 99. ^{1}H NMR Spectrum of substrate 51

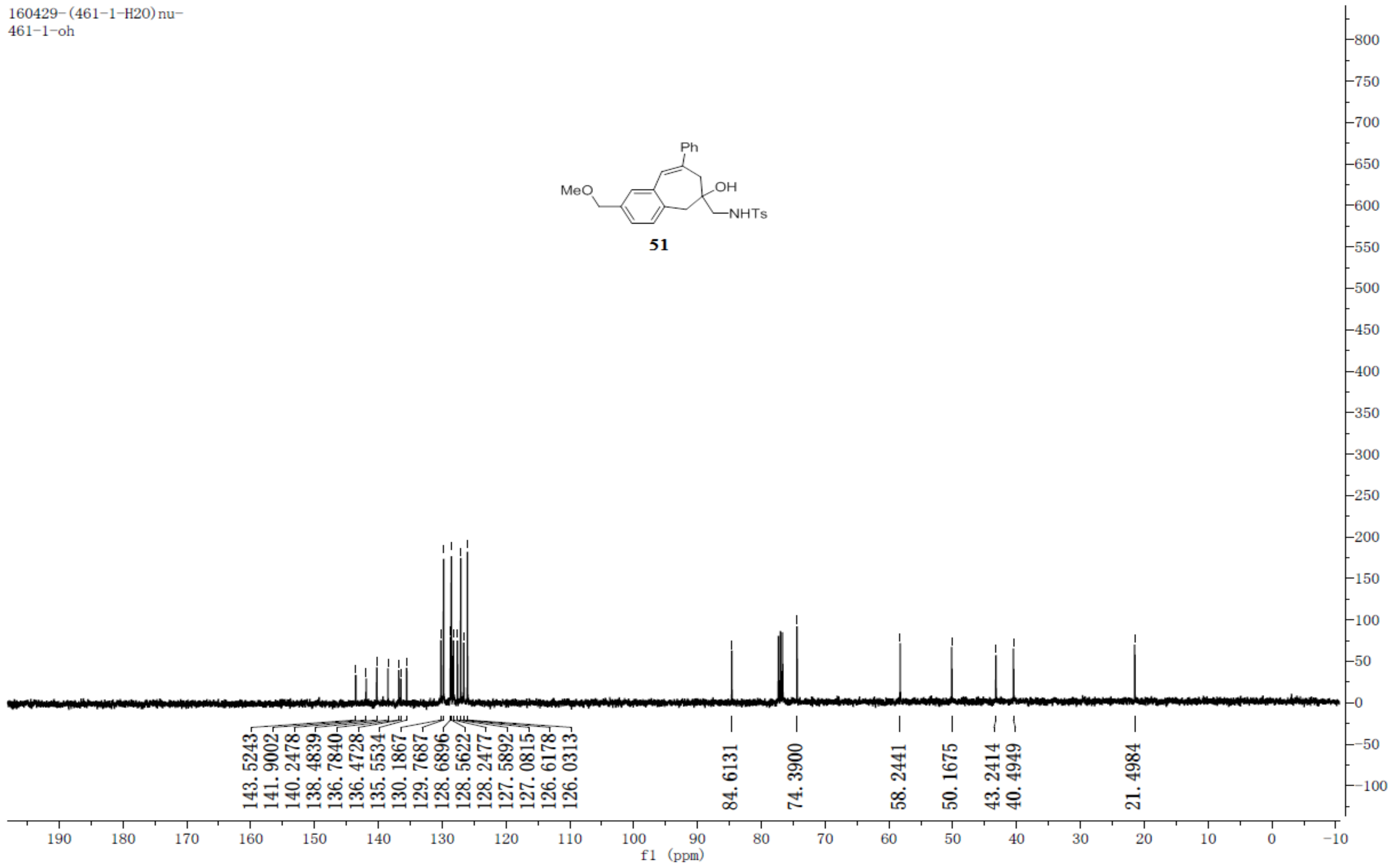

Supplementary Figure 100. ^{13}C NMR Spectrum of substrate 51

Supplementary References

1. Russo, F., Wångsell, F., Sävmarker, J., Jacobsson, M. & Larhed, M. Synthesis and evaluation of a new class of tertiary alcohol based BACE-1 inhibitors. *Tetrahedron* **65**, 10047-10059 (2009).
2. Minakata, S., Morino, Y., Oderaotoshi, Y. & Komatsu, M. Practical and Convenient Synthesis of N-Heterocycles: Stereoselective Cyclization of N-Alkenylamides with *t*-BuOI under Neutral Conditions. *Org. Lett.* **8**, 3335–3337 (2006).
3. Lindsay, V. N. G., Viart, H. M.-F. & Sarpong, R. Stereodivergent Intramolecular C(sp^3)−H Functionalization of Azavinyl Carbenes: Synthesis of Saturated Heterocycles and Fused N‑ Heterotricycles. *J. Am. Chem. Soc.* **137**, 8368−8371 (2015).
4. Johan Verendel, J., Zhou, T., Li, J.-Q., Paptchikhine, A., Lebedev, O. & Andersson, P. G. Highly Flexible Synthesis of Chiral Azacycles via Iridium-Catalyzed Hydrogenation. *J. Am. Chem. Soc.* **132**, 8880–8881 (2010).
5. Fürstner, A., Szillat, H. & Stelzer. F. Novel Rearrangements of Enynes Catalyzed by $PtCl_2$. *J. Am. Chem. Soc.* **122**, 6785-6786 (2000).
6. Sakakura, A., Sakuma, M. & Ishihara K. Chiral Lewis Base-Assisted Brønsted Acid (LBBA)-Catalyzed Enantioselective Cyclization of 2-Geranylphenols. *Org. Lett.* **13**, 3130–3133 (2011).
7. Nieto-Oberhuber, C., Pérez-Galán, P., Herrero-Gómez, E., Lau-terbach, T., Rodríguez, C., López, S., Bour, C., Rosellón, A., Cárdenas, D. J. & Echavarren, A. M. Gold(I)-Catalyzed Intra-molecular [4+2] Cycloadditions of Arylalkynes or 1,3-Enynes with Alkenes. *J. Am. Chem. Soc.* **130**, 269-279 (2008).
8. Toullec, P. Y., Genin, E., Leseurre, L., Genêt, J.-P. & Michelet, V. Room-Temperature AuI-Catalyzed C-C Bond Formation through a Tandem Friedel–Crafts-Type Addition/Carbocyclization Reaction. *Angew. Chem. Int. Ed.* **45**, 7427-7430 (2006).
